# Supplementary material for: Spatial deconvolution of HER2-positive breast cancer delineates tumor-associated cell type interactions
Source: Nat Commun. 2021 Oct 14;12:6012. doi: 10.1038/s41467-021-26271-2 (PMC8516894; doi:10.1038/s41467-021-26271-2)
Supplement: Supplementary file 1 — Supplementary Information [file 41467_2021_26271_MOESM1_ESM.pdf]

Supplementary Information for:

Spatial deconvolution of HER2-positive Breast cancer delineates tumor-associated cell type interactions

Alma Andersson[1†], Ludvig Larsson[1†], Linnea Stenbeck[1†], Fredrik Salmén[1,2], Anna Ehinger[3,4], Sunny Z Wu[5,6], Ghamdan Al-Eryani[5,6], Daniel Roden[5,6], Alex Swarbrick[5,6], Åke Borg[4], Jonas Frisén[7], Camilla Engblom[7], Joakim Lundeberg[1*]

1 Science for Life Laboratory, Division of Gene Technology, KTH Royal Institute of Technology, Stockholm, Sweden

2 Hubrecht Institute-KNAW (Royal Netherlands Academy of Arts and Sciences) and University Medical Center Utrecht, Cancer Genomics Netherlands, Utrecht, the Netherlands

3 Department of Genetics and Pathology, Laboratory Medicine Region Skåne, Lund, Sweden

4 Department of Clinical Sciences Lund, Division of Oncology, Lund University, Lund, Sweden

5 The Kinghorn Cancer Centre, Garvan Institute of Medical Research, Sydney, Australia

6 St Vincent's Clinical School, Faculty of Medicine, Sydney, Australia

7 Department of Cell and Molecular Biology, Karolinska Institutet, Stockholm, Sweden

† - Equal contribution

* - Corresponding author (joakim.lundeberg@scilifelab.se)

This PDF file includes:

Supplementary Figure 1-26

Supplementary Table 1-3

Supplementary Note 1-3

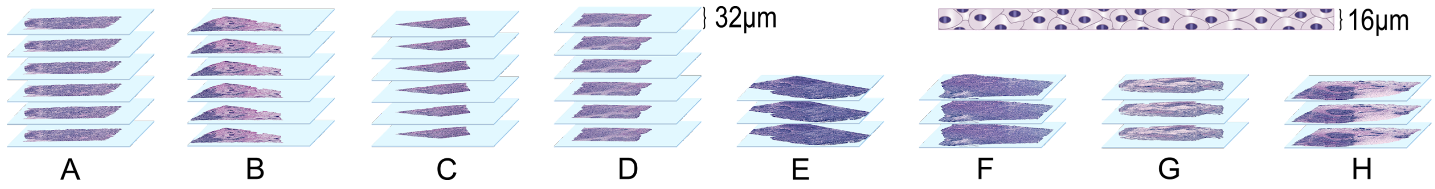

Supplementary Figure 1 | Experimental setup

From patient A-D, six cryosections were collected, separated by a distance of 32 μm. From patient E-H, three consecutive cryosections were collected. Each section was taken with a thickness of 16 um and placed on an ST-array.

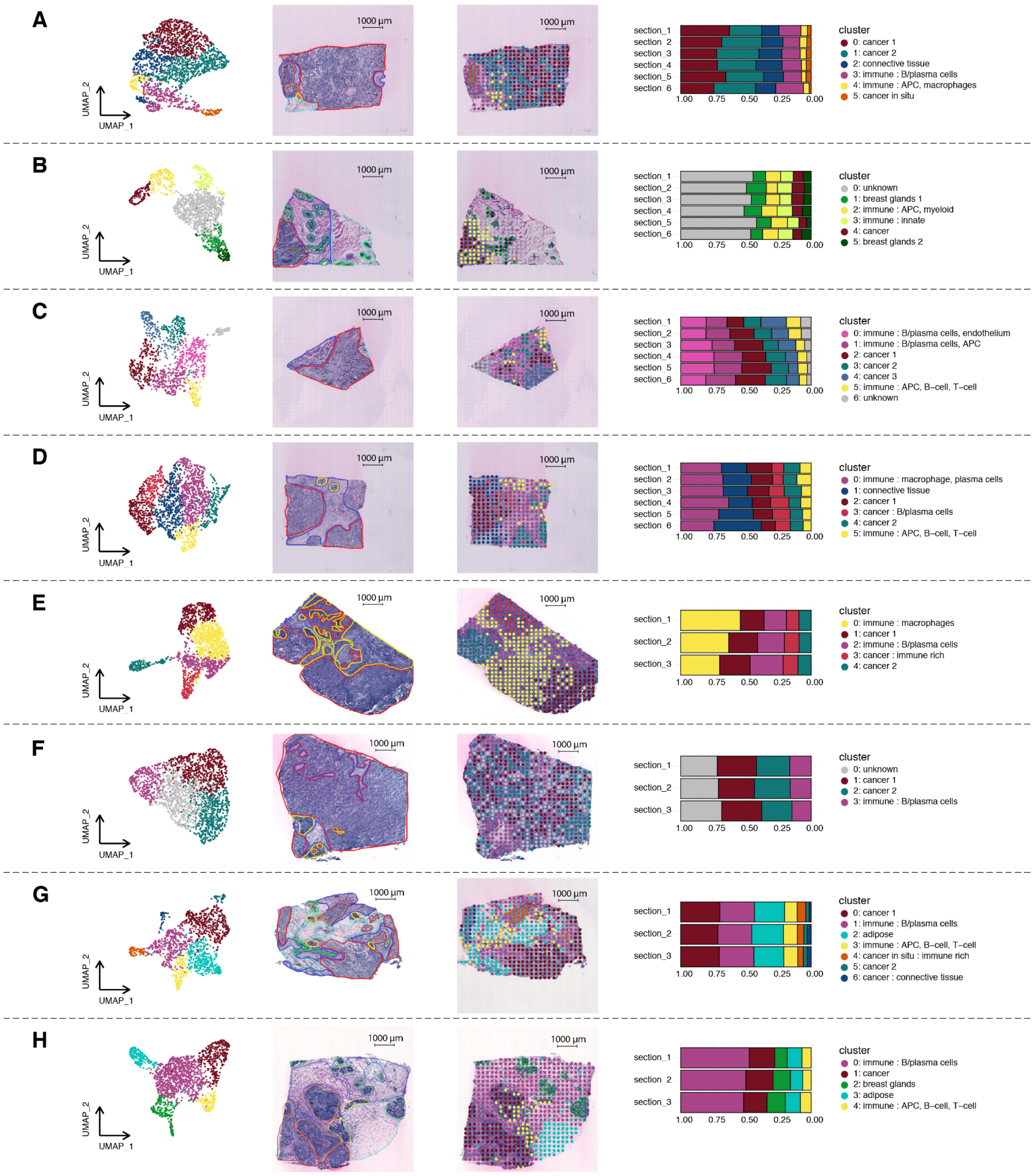

Supplementary Figure 2 | Expression-based clustering overview

Column 1. UMAP visualization of spots colored by cluster identity. **Column 2**. Annotations made by a pathologist into six distinct categories: Invasive Cancer (red), Adipose tissue (cyan), Connective tissue (blue), Breast glands (green), in situ cancer (orange) and immune infiltrates (yellow). **Column 3.** Spatial visualization of clusters, spots are colored by cluster identity. **Column 4.** Proportions of spots belonging to each cluster across replicate sections

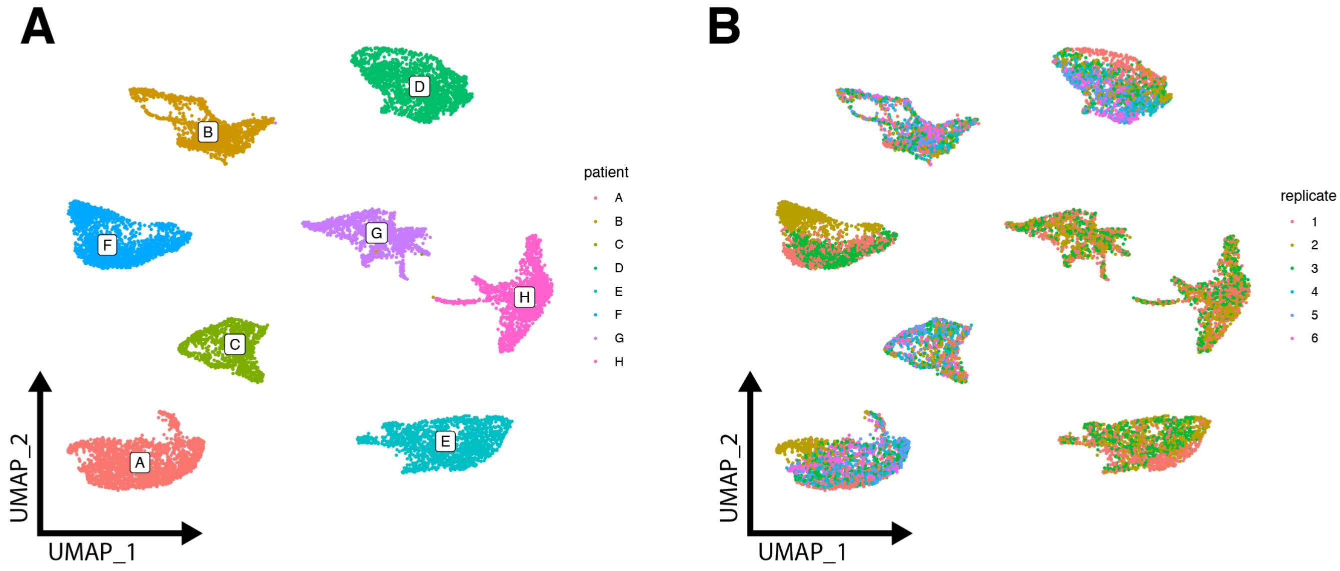

Supplementary Figure 3 | UMAP-plot

A. UMAP visualization of all spots colored by patient id. **B.** UMAP visualization of all spots colored by replicate id.

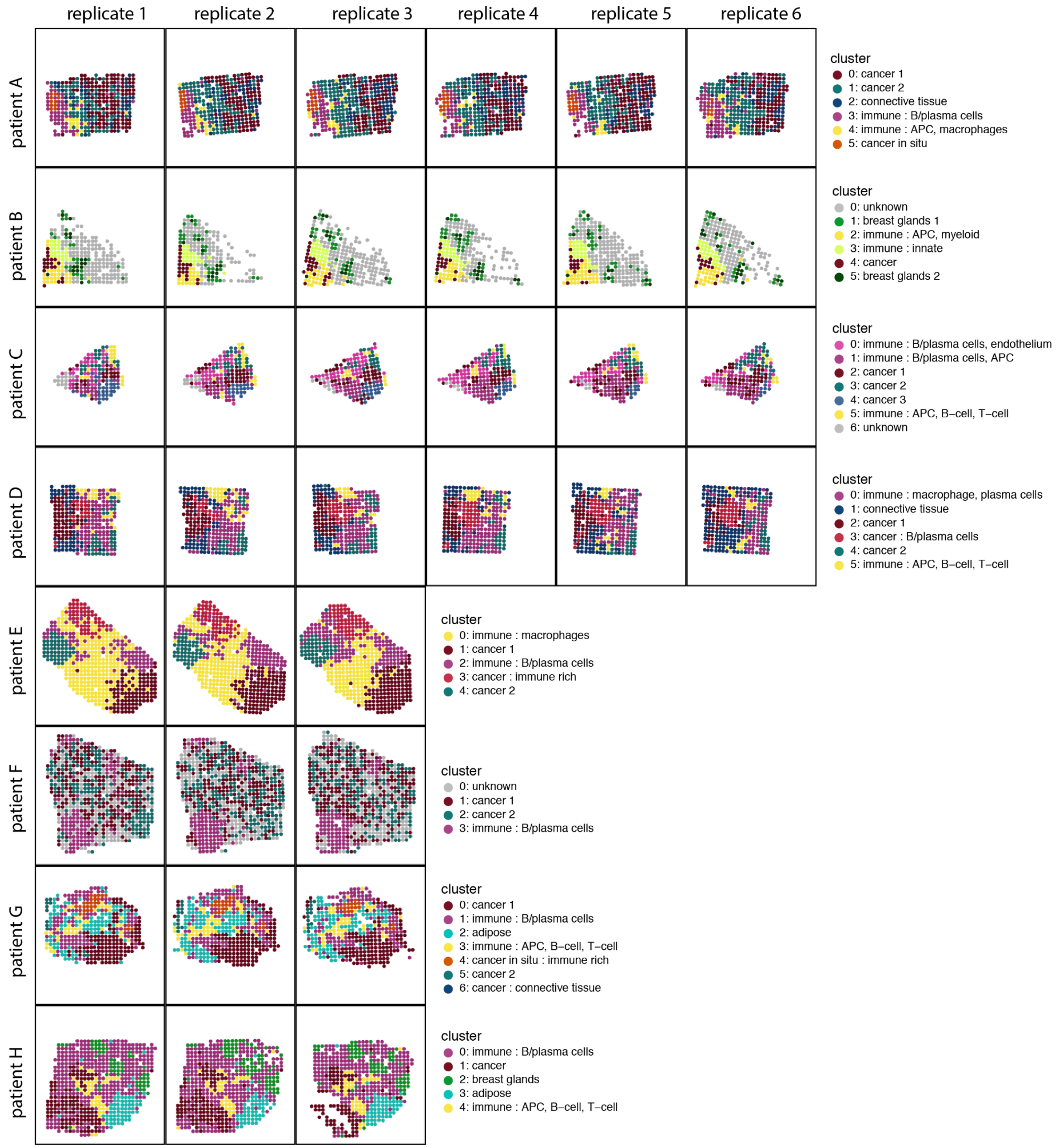

Supplementary Figure 4 | Spatial arrangement of all expression-based clusters

Spatial visualization of clusters across all replicates for each patient (A-H). Spots with the same colors belong to the same clusters. Clusters are not shared between patients. APC is short for antigen presenting cells.

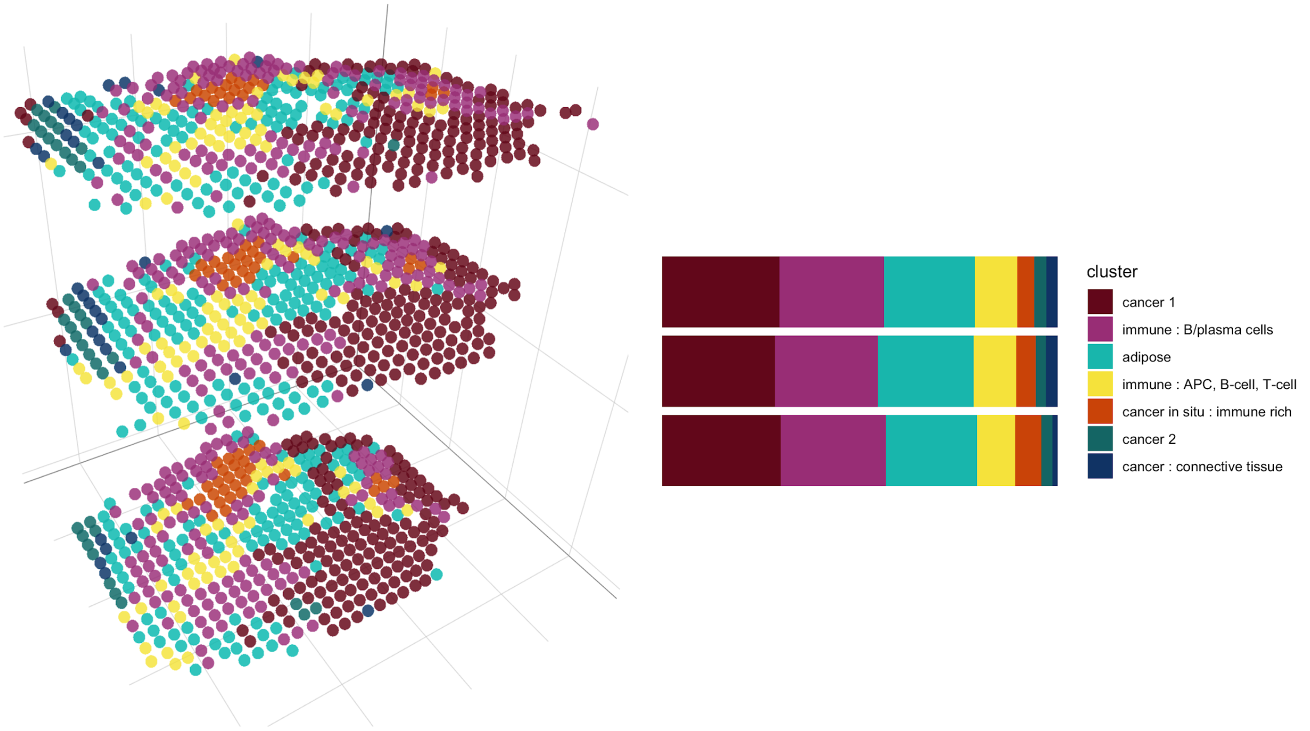

Supplementary Figure 5 | 3D visualization of Patient G

The three replicates taken from Patient G, visualizing the expression based clusters' distribution in all three dimensions. Distances in the XY-plane and Z-axis are, for ease of visualization, not depicted in the same scale. APC is short for antigen presenting cells.

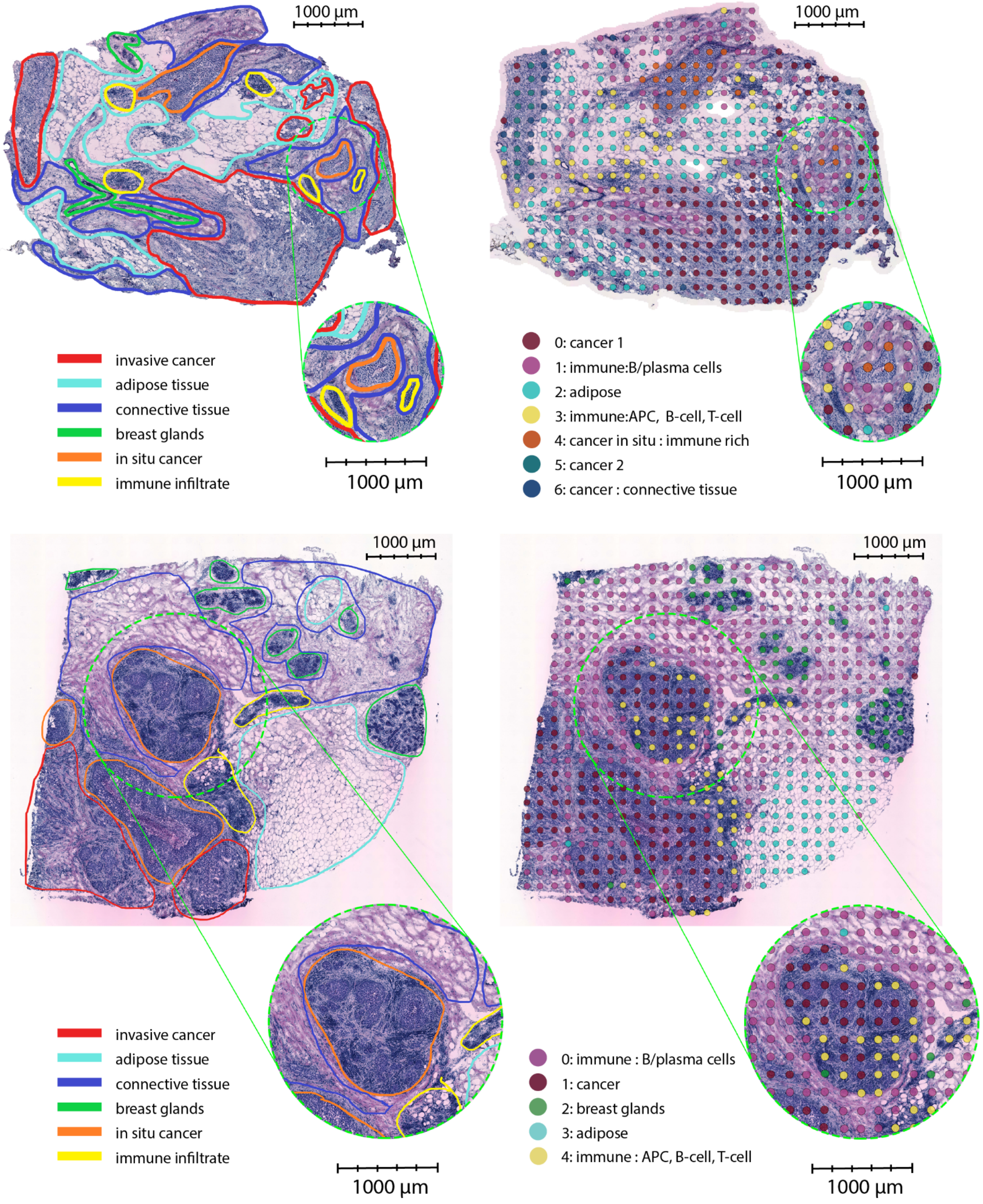

Supplementary Figure 6 | Added scale bar

(Top row) A region in patient G consisting of only three spots and aligning with the in situ cancer regions is assigned to a different cluster than its spatial neighbors (orange), the same cluster as the rest of the in situ cancer spots are found in. (Bottom row) A region with in situ cancer in patient H is populated by two different clusters (dark purple and yellow). APC is short for antigen presenting cell.

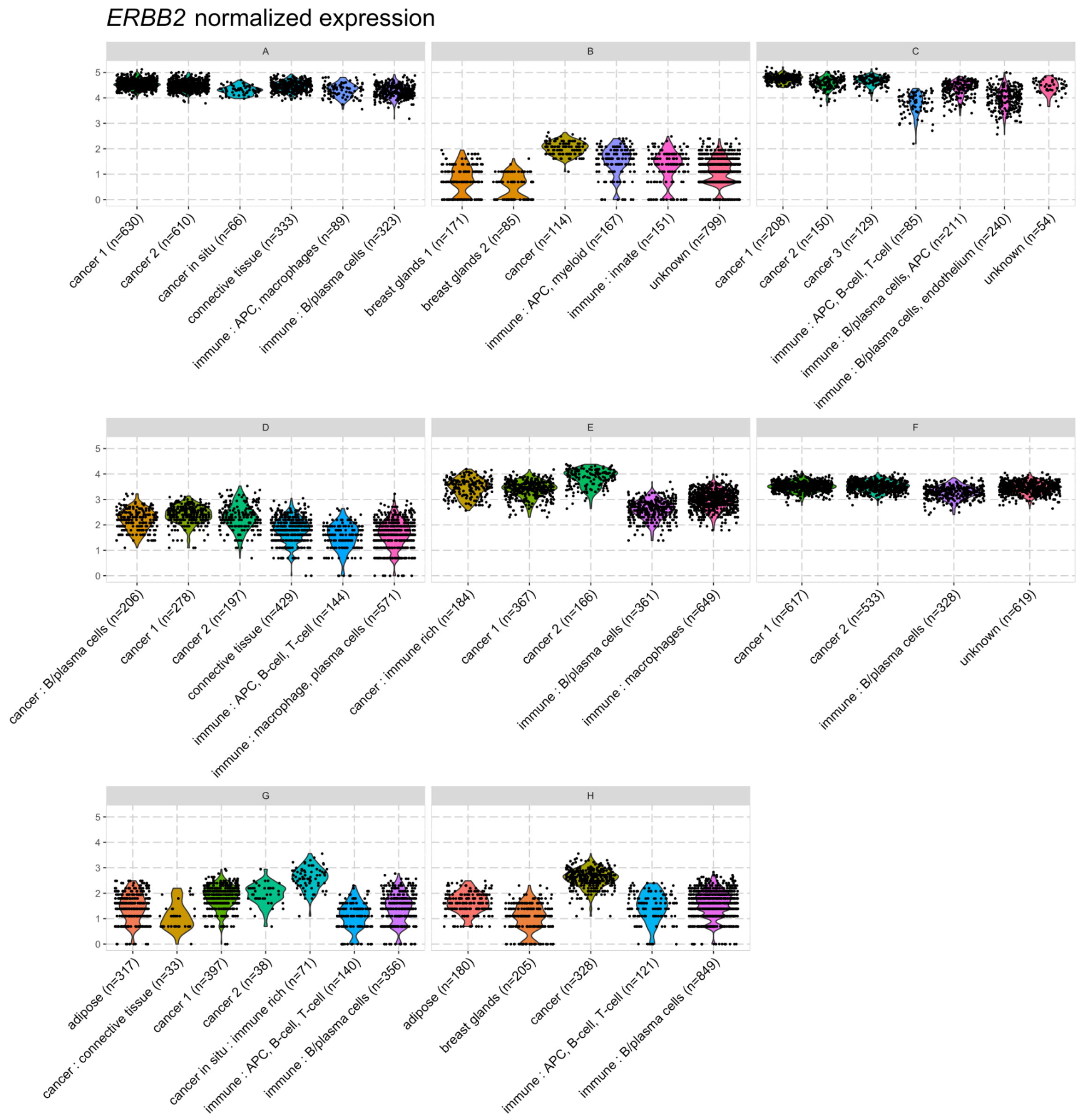

Supplementary Figure 7 | Normalized HER2 gene expression across clusters

Normalized, within each patient, expression of *ERBB2* (encoding the HER2-receptor). Each box represents the aggregated set of expression values for a given cluster taken over all replicates from the same patient. As can be seen, patient A exhibits high levels of *ERBB2* expression in all clusters. This can be explained by the fact that the tissue samples (from patient A) have a high degree of invasive and in situ cancer, hence cancer cells are present — although often in conjunction with other cell types — in most parts of the tissue. The cancer cells constitute a “cancer background” signal that is accounted for in the contrastive differential expression analysis but emerges when looking at individual transcripts like *ERBB2*. APC is short for antigen presenting cell.

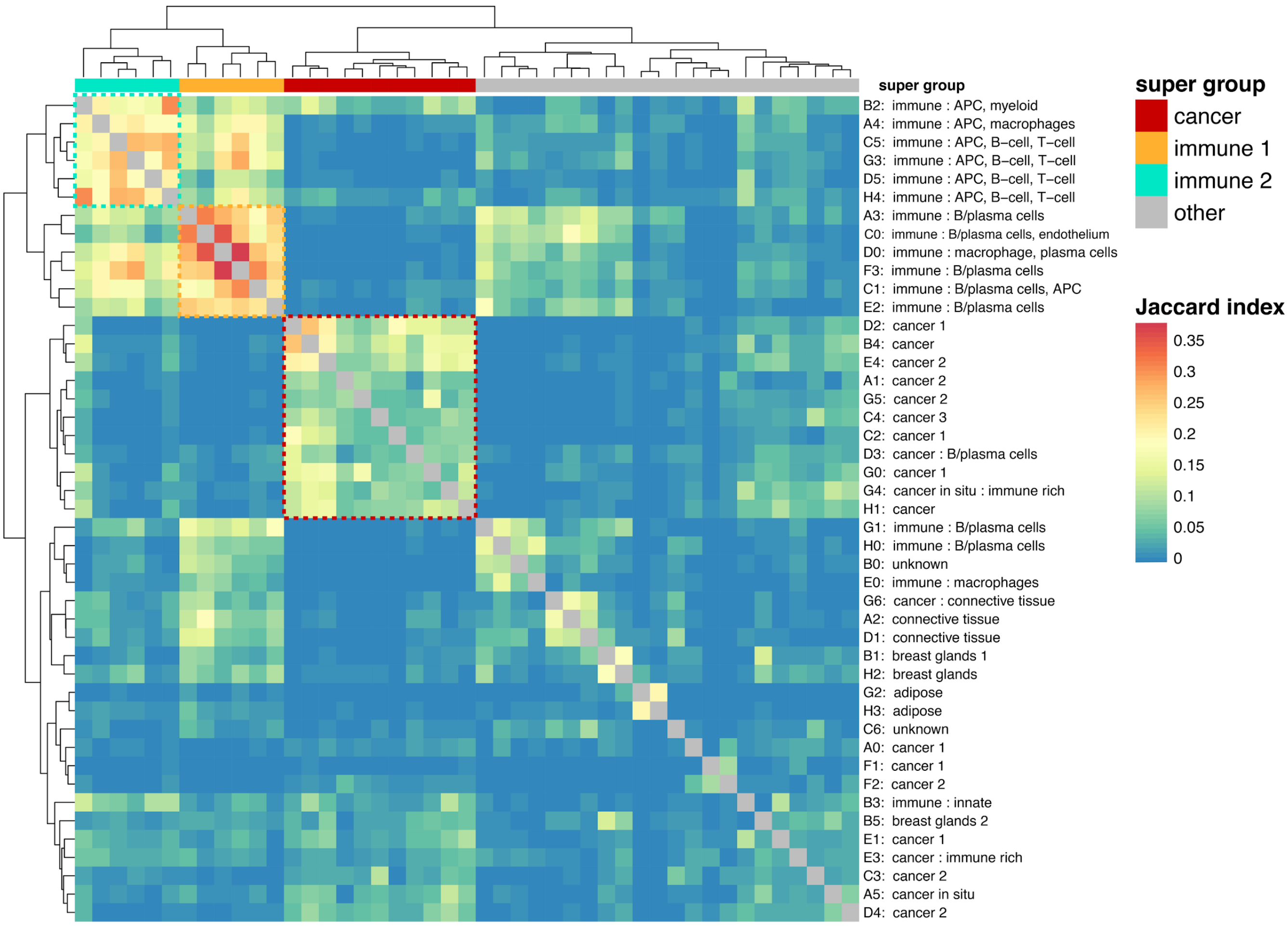

Supplementary Figure 8 | Clusters-of-clusters plot

Heatmap colored by Jaccard index values calculated across cluster geneset pairs. The three cluster supergroups are highlighted by dashed boxes, each defined by their upregulation of core signature genes. Group 1: cancer, group 2 and 3: immune related. APC is short for antigen presenting cell.

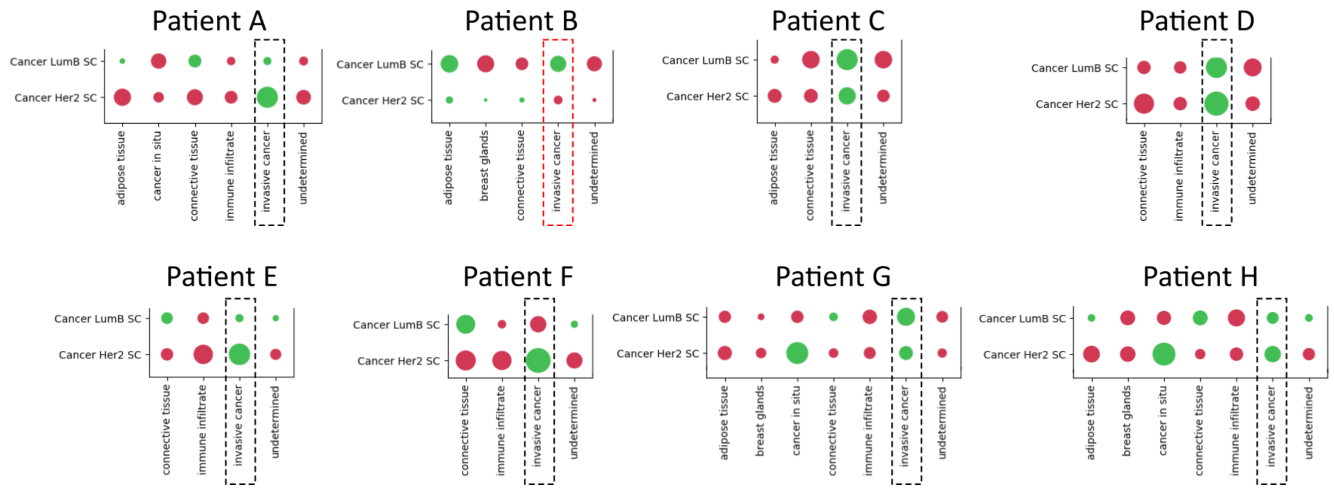

Supplementary Figure 9 | Cancer cell type enrichment across all patients

Enrichment/depletion plots of HER2 and Luminal B (LumB) associated cancer cell types in respective patients in the regions defined by the pathologist. Dashed boxes indicate regions of invasive cancer. Patient B (red dashed box) is the only patient that exhibits depletion (red) of HER2 and enrichment (green) of LumB associated cancer cell types. SC stands for single cell.

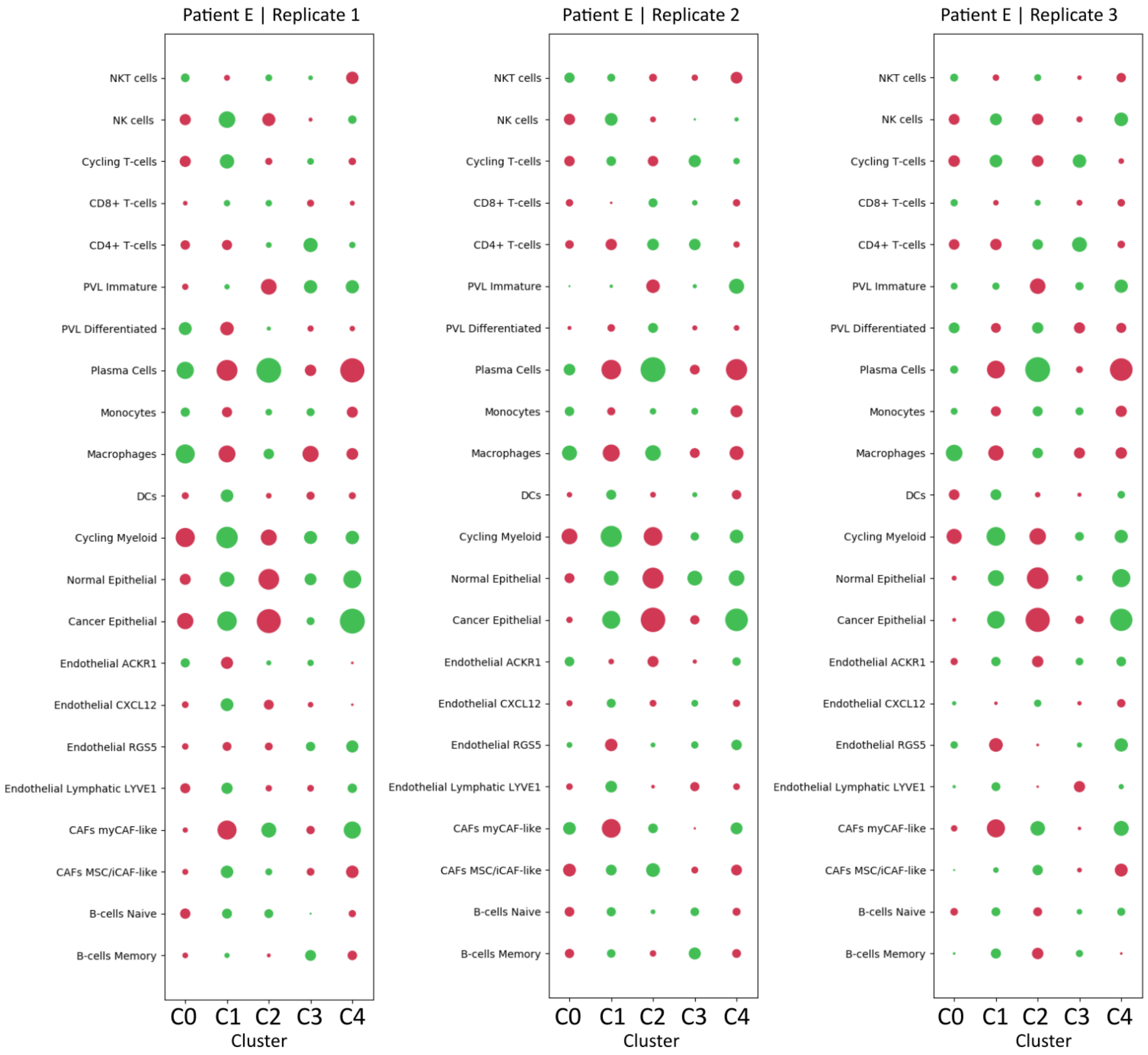

Supplementary Figure 10 | Enrichment in expression-based clusters, Patient E, minor tier

Patient E, enrichment of minor tier cell types within expression based clusters. Red is indicative of depletion, green of enrichment. Markersize is indicative of the extent of the effect (depletion or enrichment). Abbreviations are: PVL - perivascular, DCs - dendritic cells, CAFs - cancer associated fibroblasts (my = myofibroblastic, i = inflammatory), NK - natural killer cells, NKT - natural killer T-cell, MSC - mesenchymal stem cell.

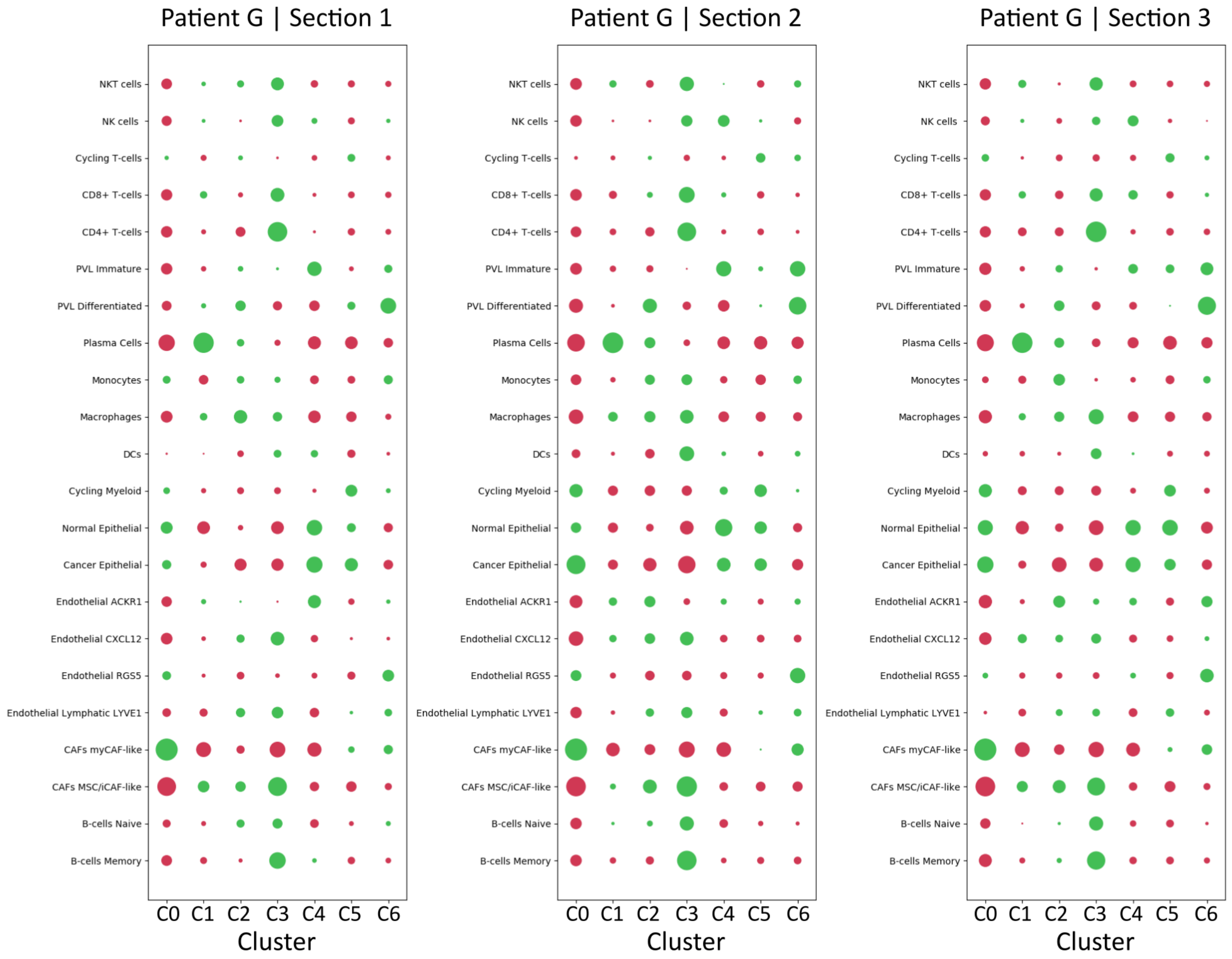

Supplementary Figure 11 | Enrichment in expression-based clusters, Patient G, minor tier

Patient G, enrichment of minor tier cell types within expression based clusters. Red is indicative of depletion, green of enrichment. Markersize is indicative of the extent of the effect (depletion or enrichment). Abbreviations are: PVL - perivascular, DCs - dendritic cells, CAFs - cancer associated fibroblasts (my = myofibroblastic, i = inflammatory), NK - natural killer cells, NKT - natural killer T-cell,MSC - mesenchymal stem cell.

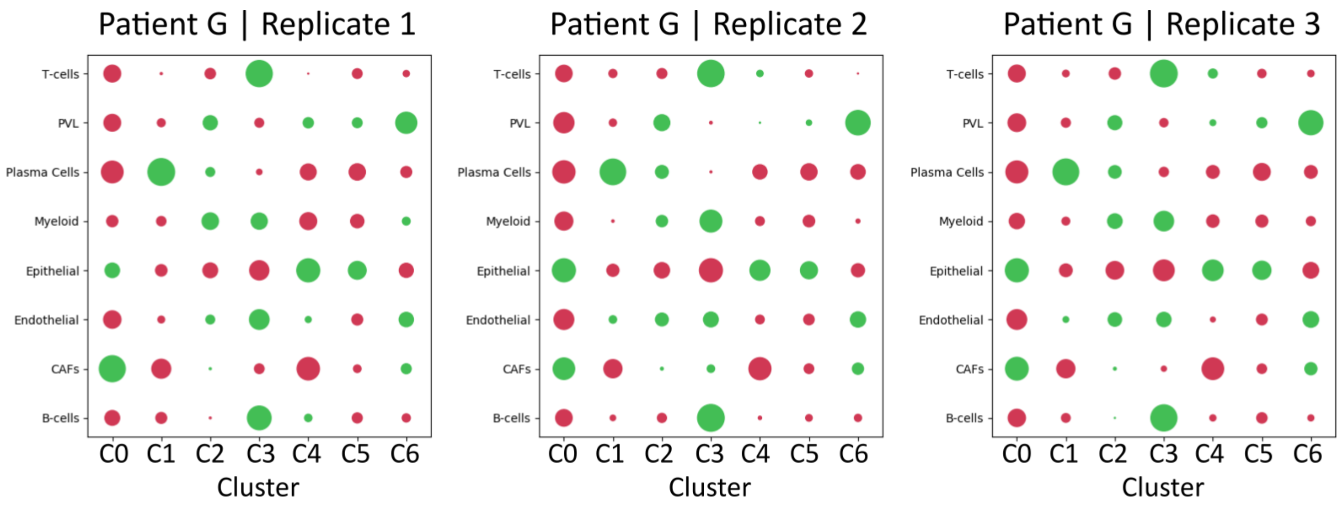

Supplementary Figure 12 | Enrichment in expression-based clusters, Patient G, major tier

Patient G, enrichment of major tier cell types within expression based clusters. Red is indicative of depletion, green of enrichment. Markersize is indicative of the extent of the effect (depletion or enrichment). Abbreviations are: PVL - perivascular, and CAFs - cancer associated fibroblasts.

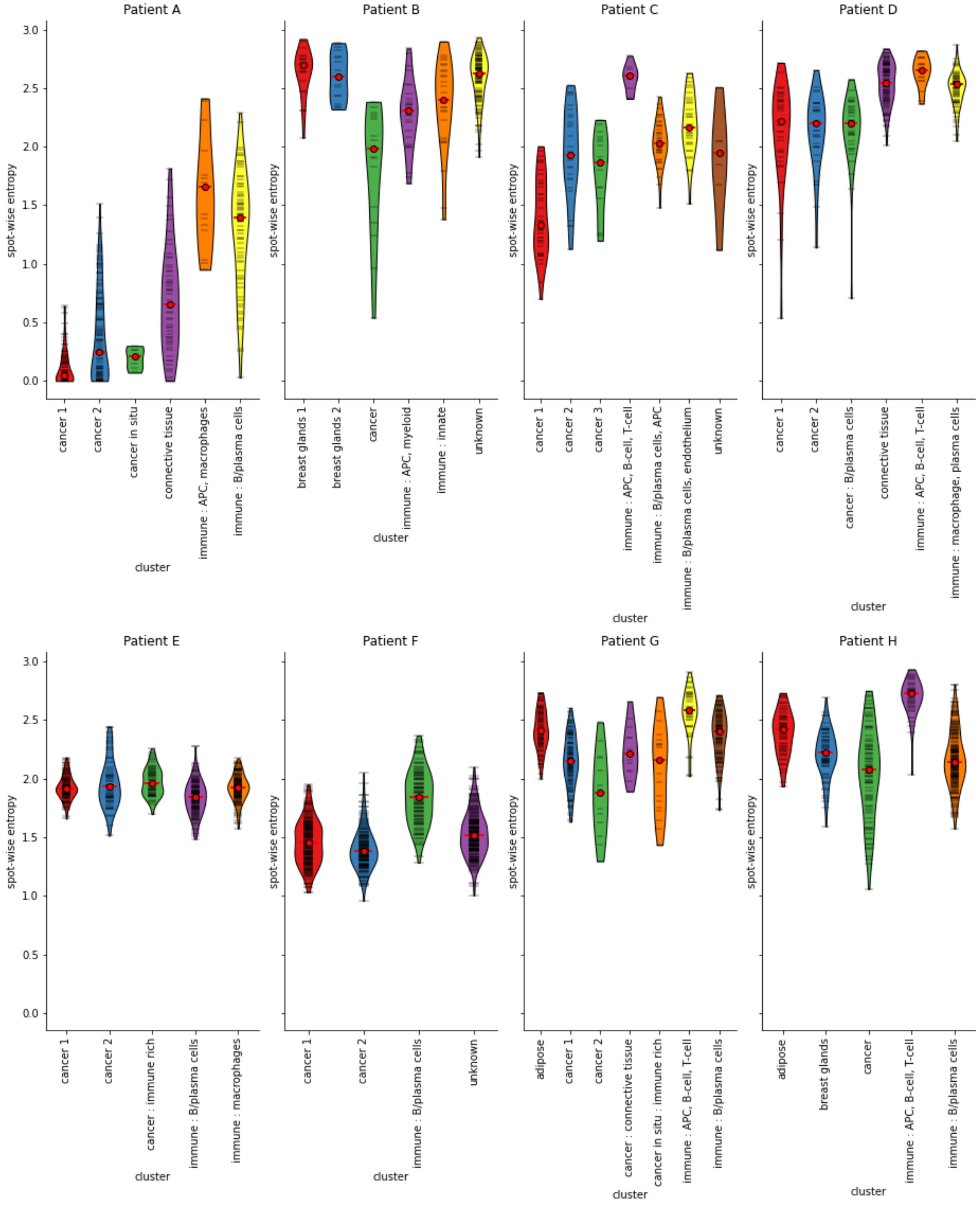

Supplementary Figure 13 | Assessing spatial diversity by entropy

Distribution of entropy values within each patient and cluster. The plots are similar to violin plots, but also indicate each observation with a horizontal black line; medians are given by a red circle. Colors are not shared between patients. APC stands for antigen presenting cells.

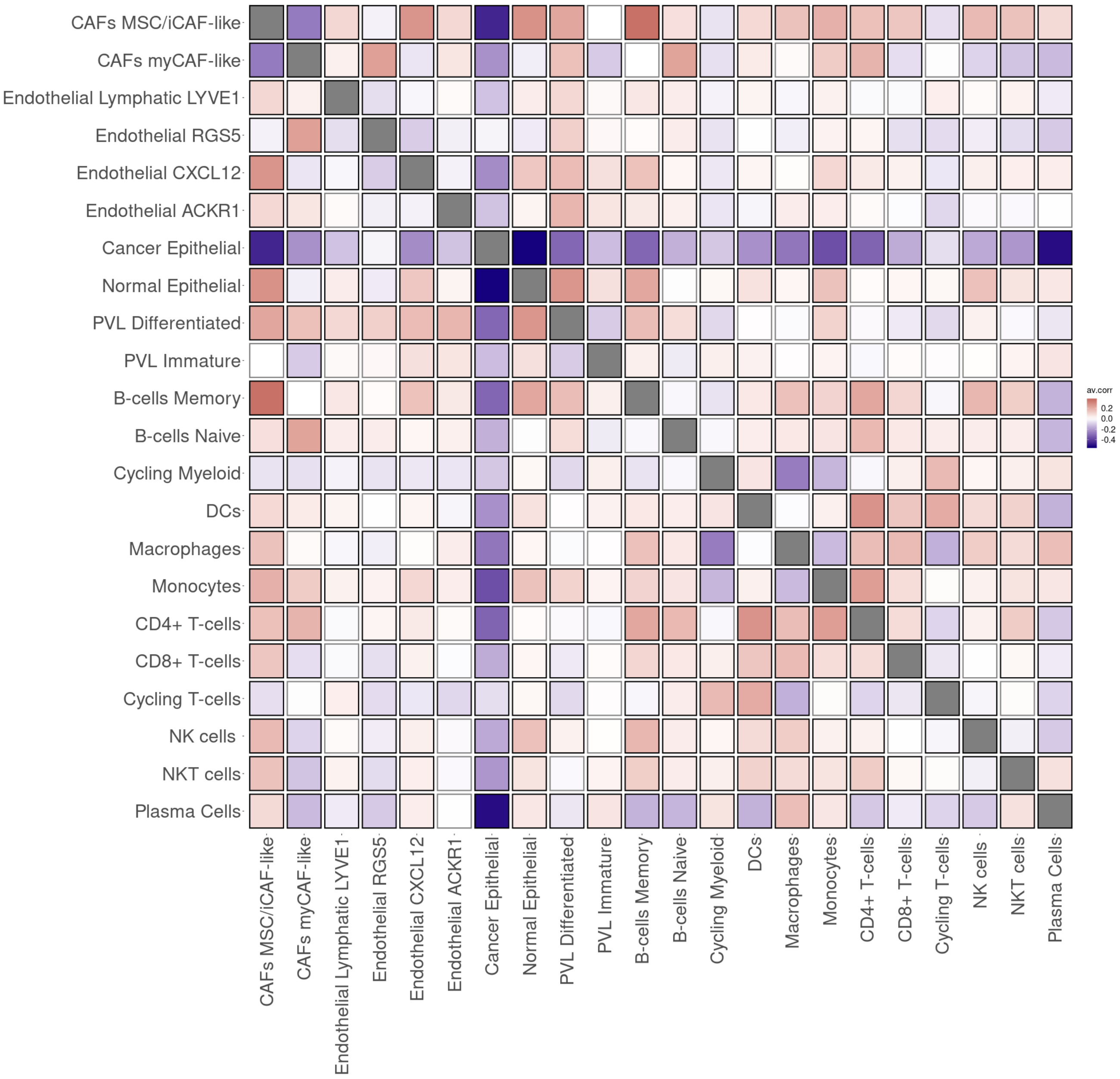

Supplementary Figure 14 | Correlation plot - All patients, minor tier

Correlation (Pearson) plot of cell type proportions across the spots, red is indicative of high spatial colocalization, blue is indicative of low spatial colocalization. The correlation values are computed over all sections, using proportion values from the minor tier mapping. Abbreviations are: PVL - perivascular, DCs - dendritic cells, CAFs - cancer associated fibroblasts (my = myofibroblastic, i = inflammatory), NK - natural killer cells, NKT - Natural T-killer cell, MSC - mesenchymal stem cell.

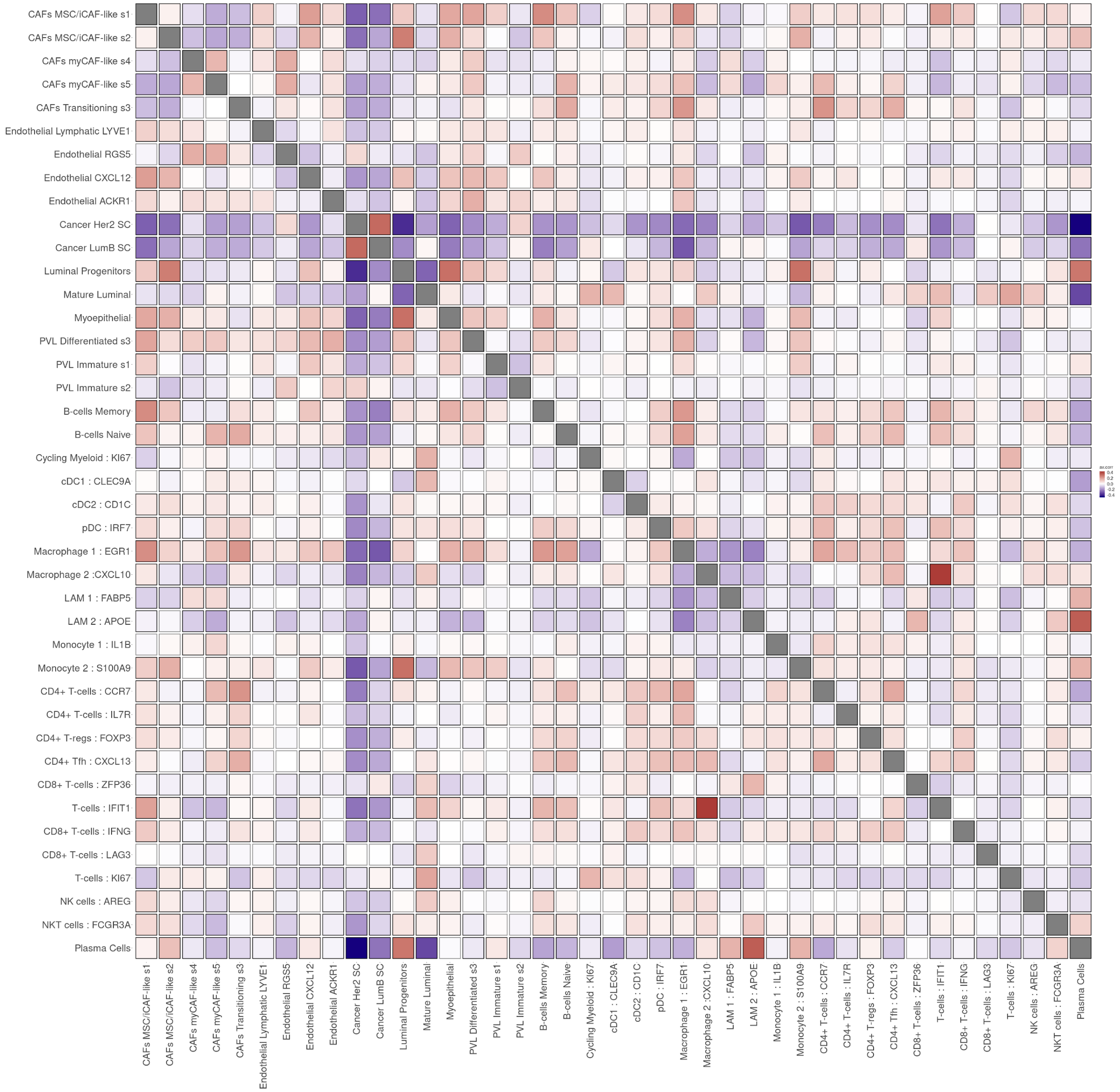

Supplementary Figure 15 | Correlation plot - All patients, subset tier

Correlation (Pearson) plot of cell type proportions across the spots, red is indicative of high spatial colocalization, blue is indicative of low spatial colocalization. The correlation values are computed over all sections, using proportion values from the subset tier mapping. Abbreviations are: PVL - perivascular, DCs - dendritic cells (c = conventional, p = plasmacytoid), CAFs - cancer associated fibroblasts (my = myofibroblastic, i = inflammatory), NK - natural killer cells, NKT - Natural T-killer cell,MSC - mesenchymal stem cell.

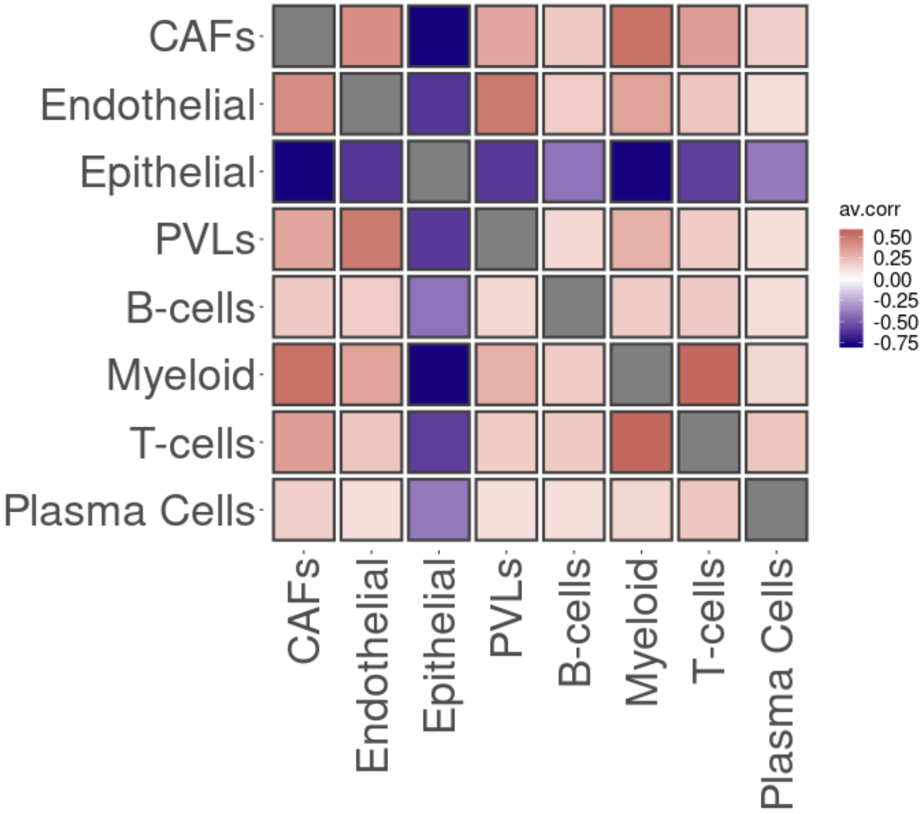

Supplementary Figure 16 | Correlation plot - Patient A, major tier

Correlation (Pearson) plot of cell type proportions across the spots, red is indicative of high spatial colocalization, blue is indicative of low spatial colocalization. The correlation values are computed over all six replicates of patient A, for the major tier. Abbreviations are: PVL - perivascular, and CAFs - cancer associated fibroblasts.

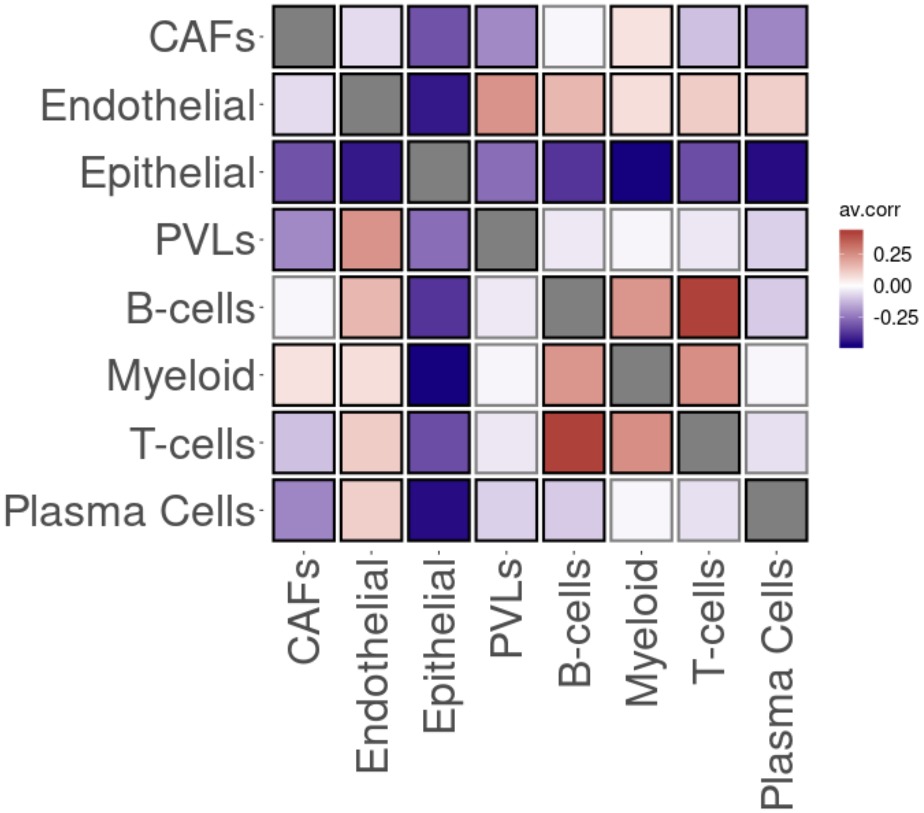

Supplementary Figure 17 | Correlation plot - patient G, major tier

Correlation (Pearson) plot of cell type proportions across the spots, red is indicative of high spatial colocalization, blue is indicative of low spatial colocalization. The correlation values are computed over all three replicates of patient G, for the major tier. Abbreviations are: PVL - perivascular, and CAFs - cancer associated fibroblasts.

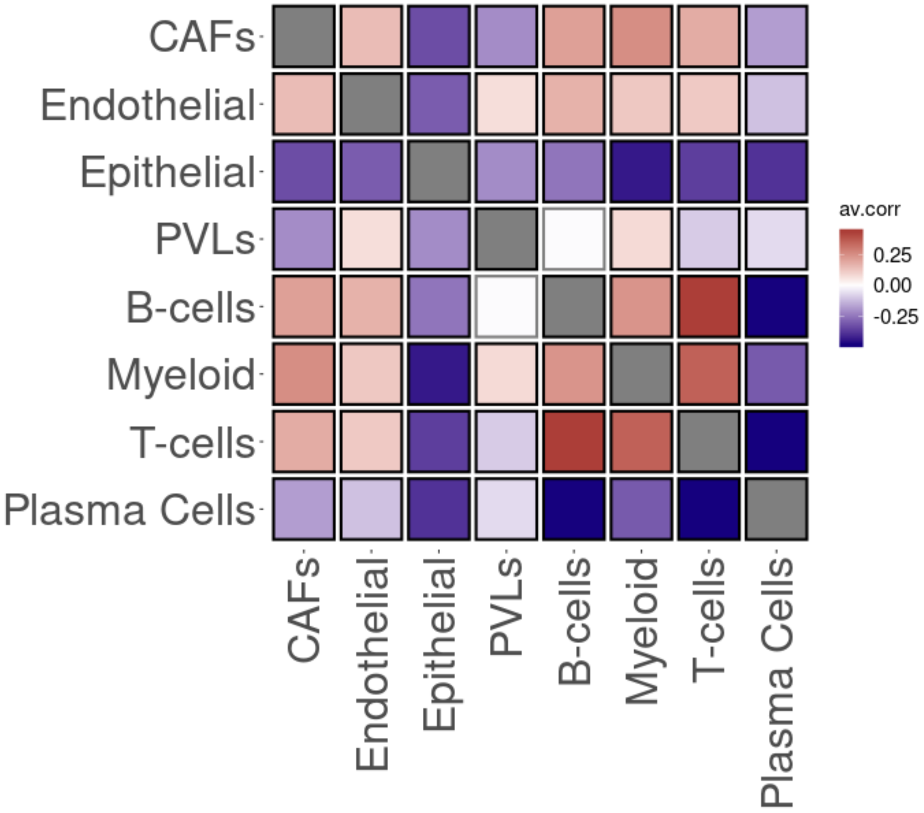

Supplementary Figure 18 | Correlation plot - Patient H, major tier

Correlation (Pearson) plot of cell type proportions across the spots, red is indicative of high spatial colocalization, blue is indicative of low spatial colocalization. The correlation values are computed over all three replicates of patient H, for the major tier. Abbreviations are: PVL - perivascular, and CAFs - cancer associated fibroblasts.

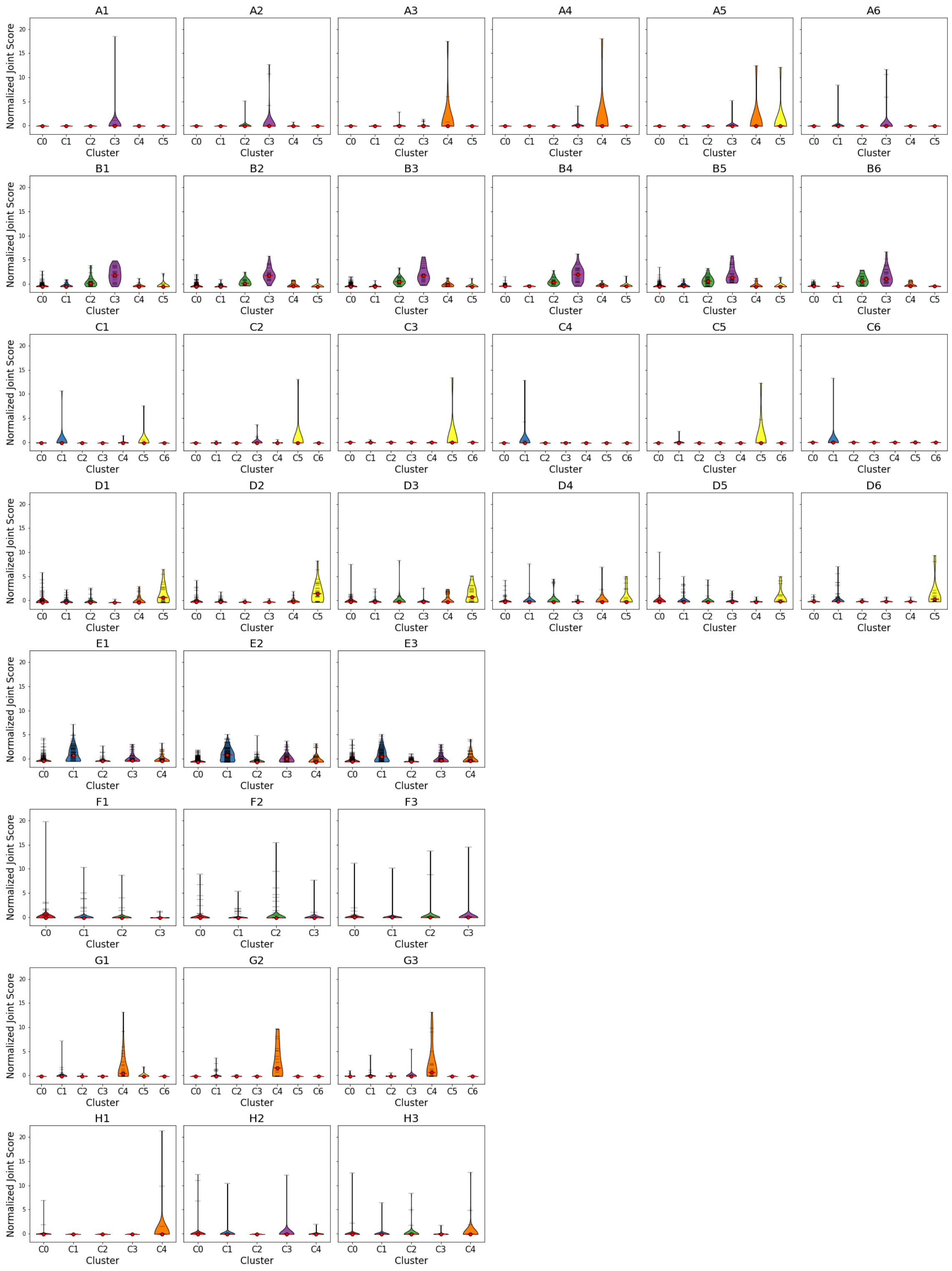

Supplementary Figure 19 | Joint Mø and T-cell subset scores across samples and clusters.

Distribution of joint proportion scores — of Macrophage 2:CXCL10 and T-cells:IFIT1 subsets — for each cluster and every patient sample. The plots are similar to violin plots, but also indicate each individual measure with a horizontal black line; medians are given by a red circle. Colors are not shared between patients.

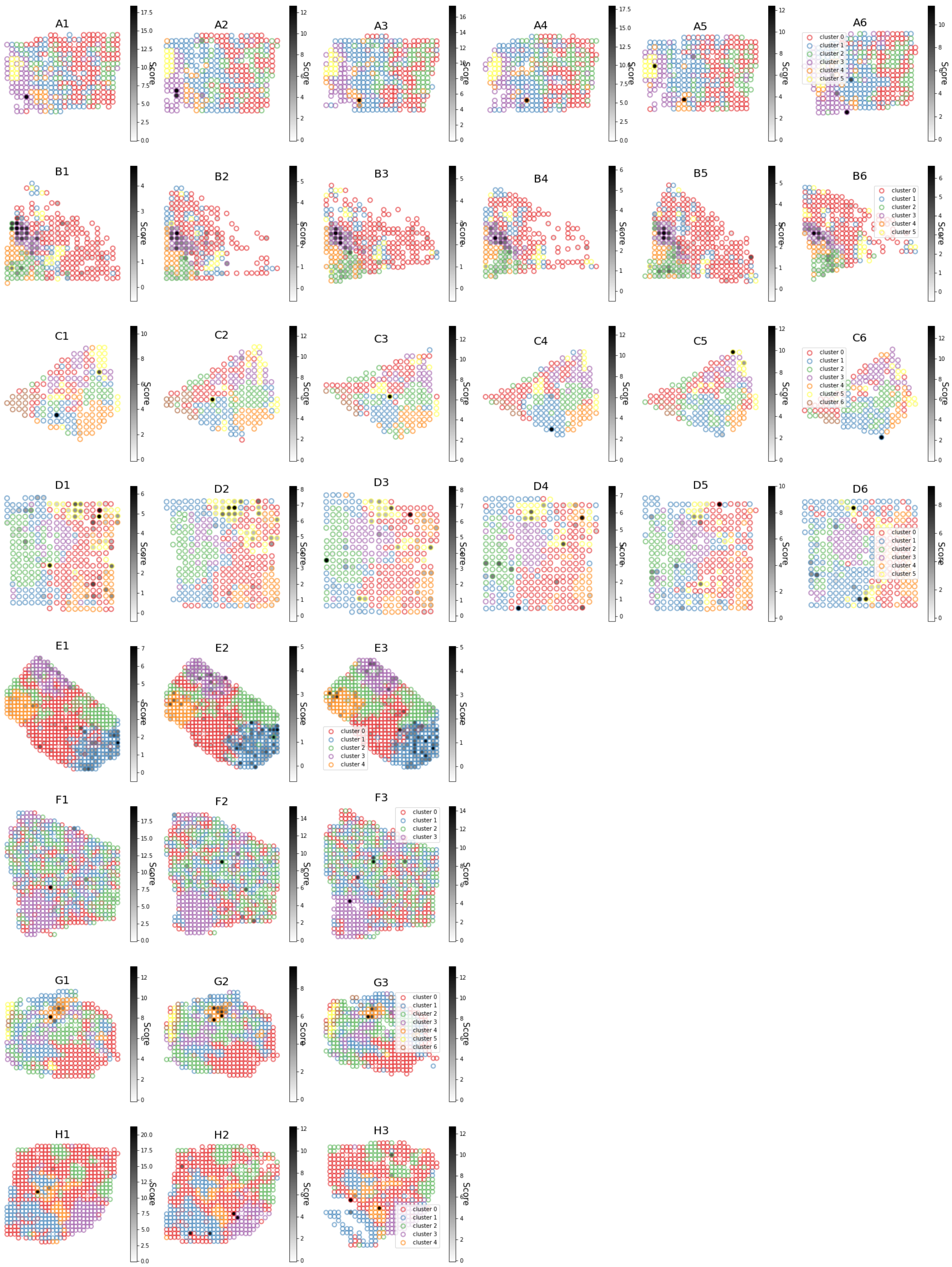

Supplementary Figure 20 | Spatial distribution of joint Macrophage 2 : CXCL10 and T-cell : IFIT1 scores

Similarly to previous spatial plots, spots are indicated by circular markers. Cluster identities are given by the edgecolor, these colors are shared with Supplementary Figure 19. The facecolor intensity of each marker is proportional to the normalized joint scores, darker values represent high scores.

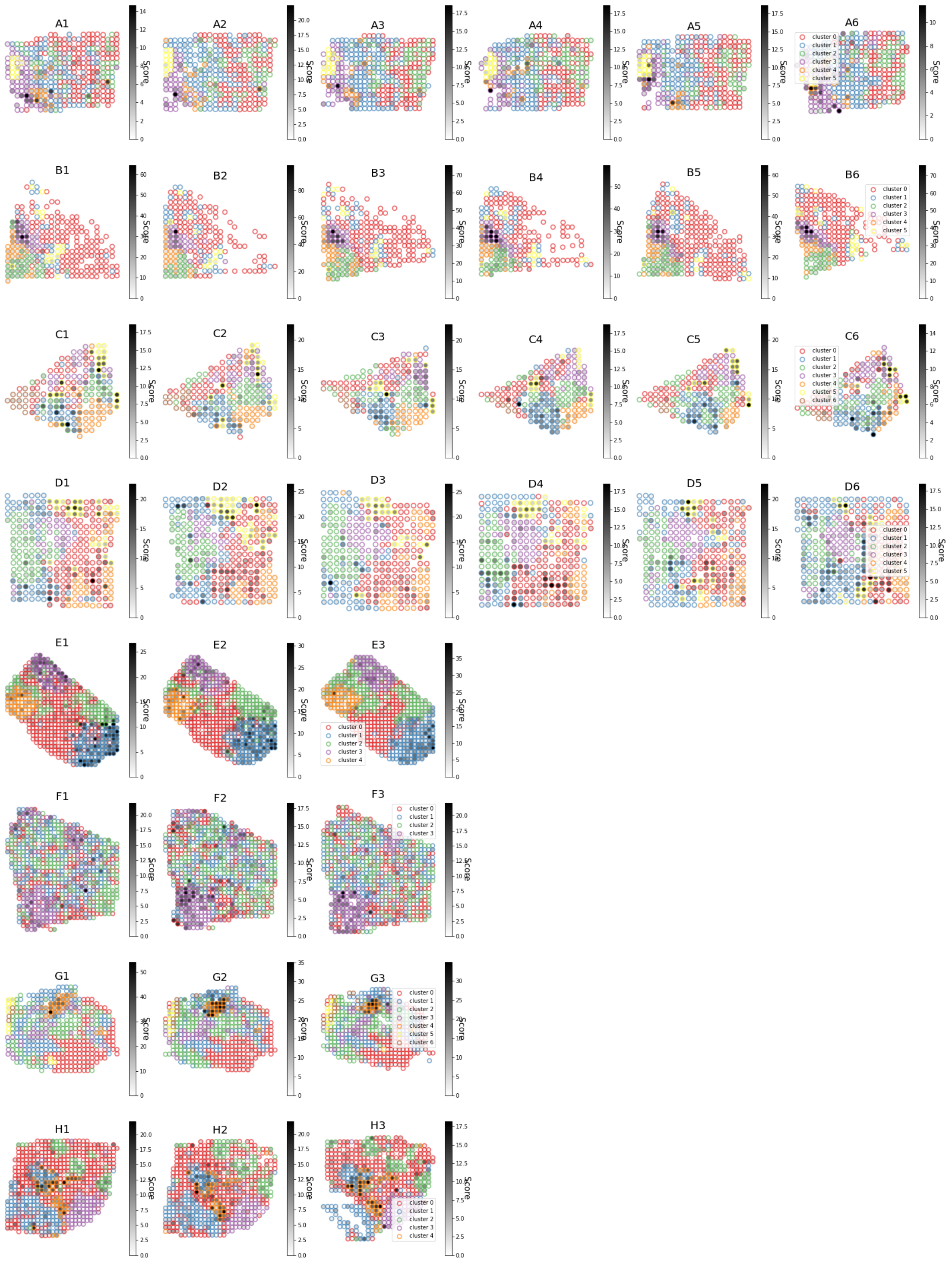

Supplementary Figure 21 | Spotwise enrichment of type I interferon signaling pathway

Similar to Supplementary Figure 20 but where facecolor intensities are proportional to the enrichment of a type I interferon signaling pathway (GO:0060337) score, computed in the same way as for Figure 4E.

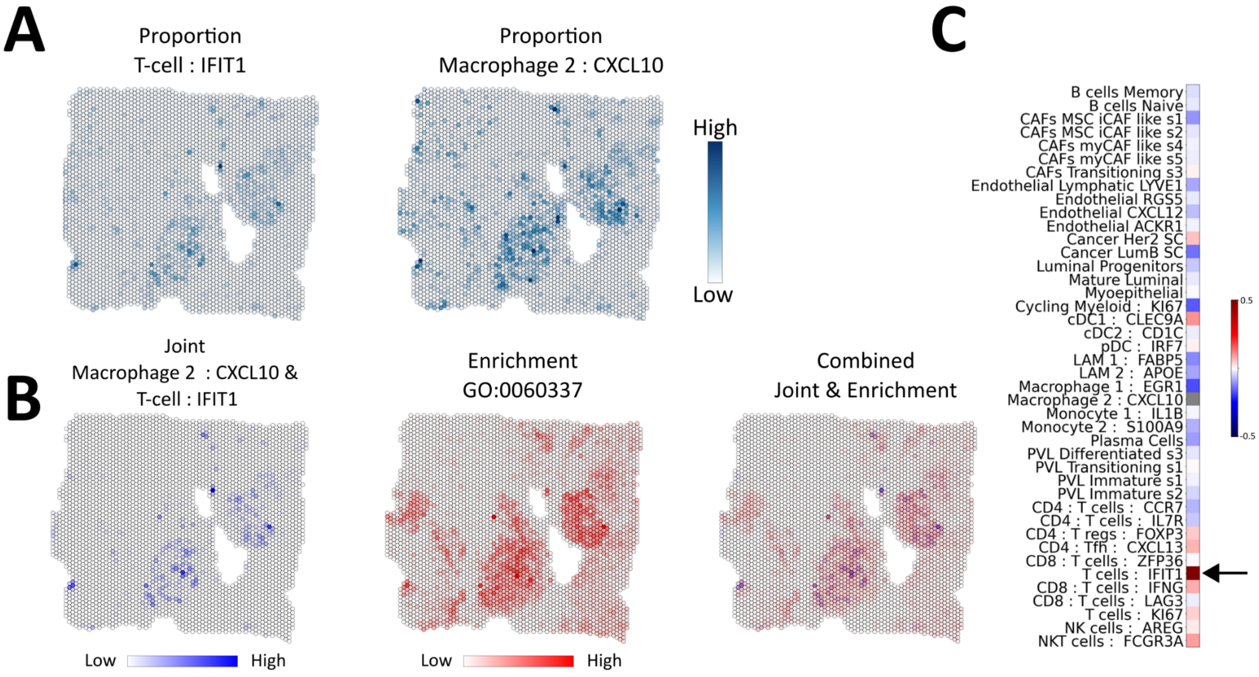

Supplementary Figure 22 | T-cell, Macrophage and type I interferon response colocalization in external HER2-positive breast cancer data

A. Proportion values of T-cell:IFIT1 and Macrophage 2:CXCL10 (Mø2:CXCL10) cell states, estimated by *stereoscope*. Values are scaled internally within each cell type (all values are divided by the max-value) for visualization purposes. **B.** (left) Spot-wise product between T-cell:IFIT1 and Mø2:CXCL10 cell states, (middle) spot-wise enrichment of the pathway “type I interferon signaling pathway” (GO:0060337), (right) combined joint product values and enrichment score; blue channel represents the joint product values and red channel the enrichment score. **C.** Pearson correlation between Mø2:CXCL10 and all other cell types at the minor tier, the black arrow indicates T-cell:IFIT1. Abbreviations are: PVL - perivascular, DCs - dendritic cells (c = conventional, p = plasmacytoid), CAFs - cancer associated fibroblasts (my = myofibroblastic, i = inflammatory), NK - natural killer cells, NKT - Natural T-killer cell,MSC - mesenchymal stem cell, LAM - lipid associated macrophages.

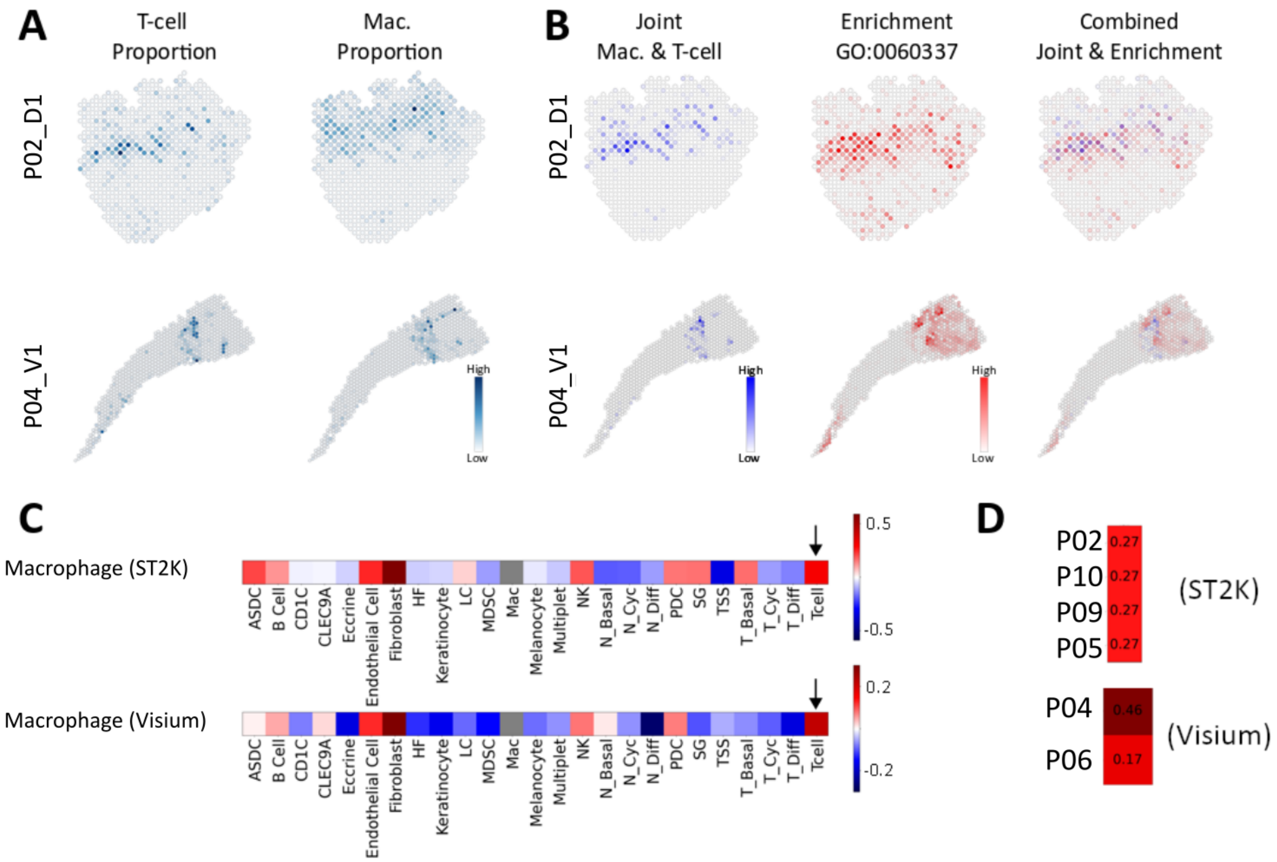

Supplementary Figure 23 | T-cell, Mø and type I interferon response colocalization in external squamous cell carcinoma

A-C are equivalent to Supplementary Figure 22A-C but using the SCC single cell and spatial data, with T-cell and Mø cell types rather than finer cell states. **D.** Pearson correlation values estimated for the two Visium (P04 and P06) specimens and the four ST2K specimens (P02,P10,P09 and P05), each specimen having two replicates. Abbreviations are: ASDC - AXL+ dendritic cells, CD1C - CD1C+ dendritic cells (DC1), LC - Langerhan Cells, MDSC - myeloid derived suppressor cells , Mac - macrophages, NK - natural killer cell, N_basal - normal basal, N_Cyc - , N_Diff - normal differentiating , PDC- plasmacytoid dendritic cells , SG -sebaceous gland related cells , TSS - tumor specific keratinocyte , T_Basal - tumor basal , T_Cyc - tumor cycling , T-Diff - tumor differentiating, HF - hair follicle related cells , CLEC9A - CLEC9A+ dendritic cells (DC2).

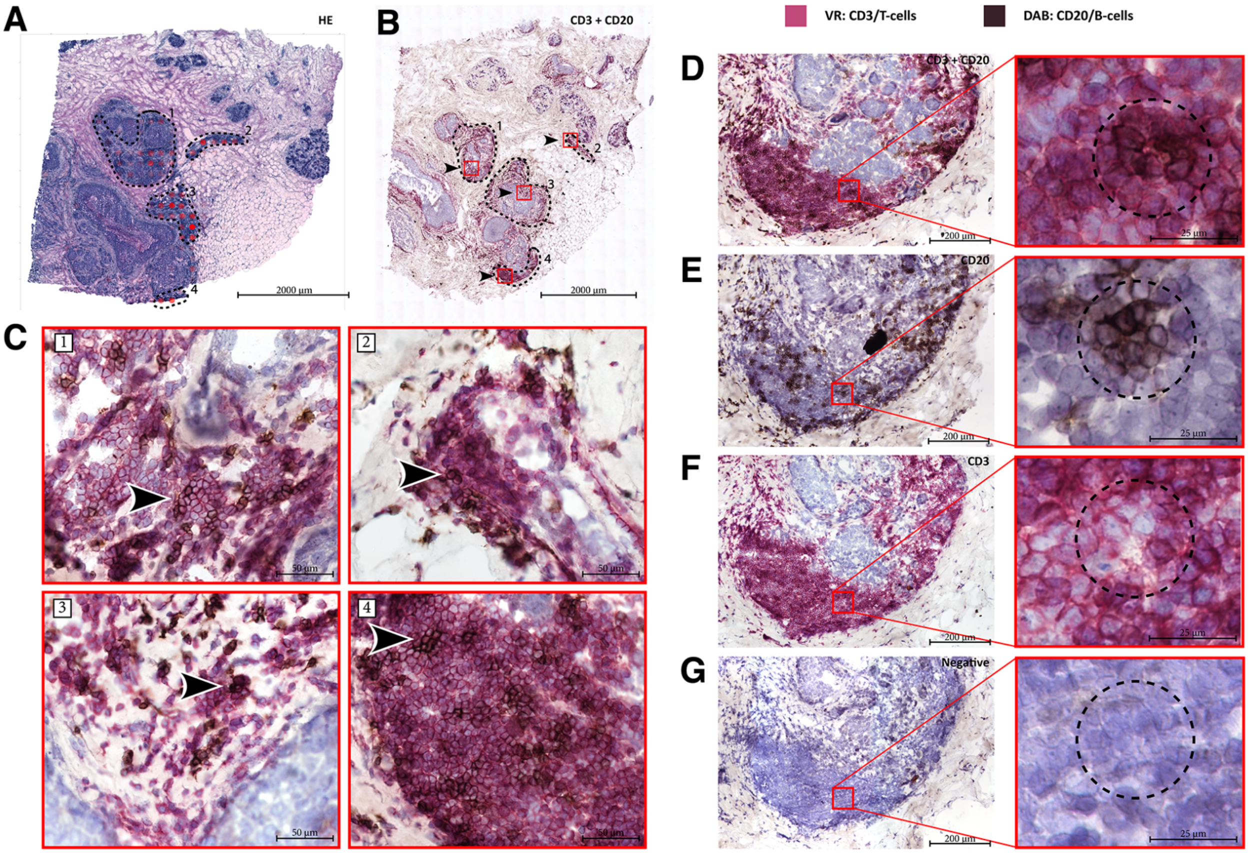

Supplementary Figure 24 | IHC validation of TL-like structures

IHC validation was performed on four consecutive sections from patient H with one section being used for dual staining, two sections for single staining, and one as negative control (not included). **A.** B and T-cell colocalization signal, derived from the single cell mapping, overlaid on the HE-image corresponding to the deconvolved ST-data. Each area containing elevated colocalization signals is enclosed by a dashed black line. **B.** Dual IHC staining with the T-cell marker CD3 (Vector Red (VR)/pink) and B-cell marker CD20 (DAB/brown), performed on a different section from the same tissue specimen, with the equivalent histological regions marked by a dashed line. **C.** 20X magnification of areas marked in B, TL-like structures are indicated by arrows **D.** TL-like structure from the dual-stained section of CD3 and CD20. **E.** The same TL-like structure from a consecutive section with respect to the dual-stain section in B, exclusively stained with CD20. **F.** The same TL-like structure from a consecutive section to the dual-stain section in B with a single stain of CD3. **G.** The same area to that of the TL-like structure from a consecutive section to the dual-stain section in B with no primary antibodies, used as a negative control.

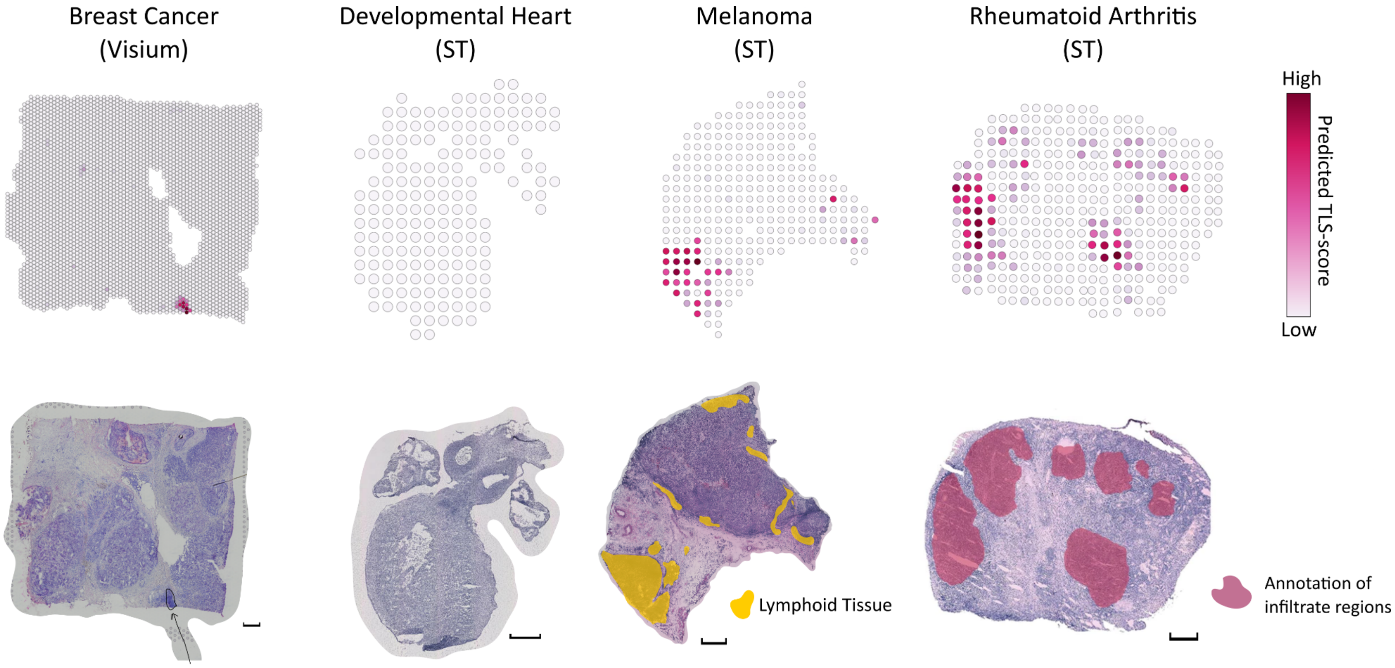

Supplementary Figure 25 | Prediction of TL-like structures in external data sets

(Top row) Predicted TLS-score of tissues from two distinct platforms, Visium and Spatial Transcriptomics (ST), as well as different tissues (breast cancer, developmental heart, melanoma and rheumatoid arthritis). (Bottom row) HE-images of the corresponding tissue. The HE-image and annotations used in the original melanoma sample publication were shared with us by the authors.[1] The annotated image of the rheumatoid arthritis (RA) sample is an adaptation of Figure 1A from Carlberg et.al, used under a creative commons license (CC BY).[2] Our pathologist has annotated the Visium sample, with an arrow indicating a likely TLS site (the only candidate identified) . A cutoff of 0.0 was used when visualizing the TLS-score predictions. Scale bars indicate 500µm, and are associated with the image of each section.

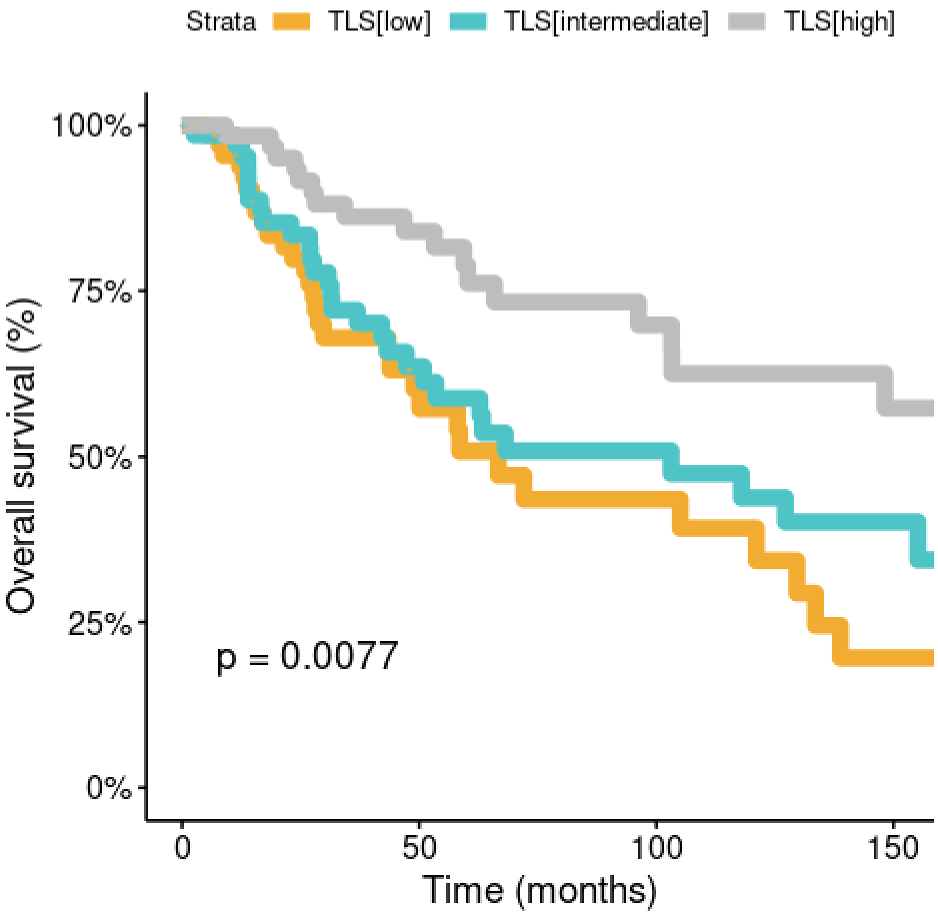

Supplementary Figure 26 | Kaplan-Meier Plot based on TLS-score prediction of TCGA SKCM data

Kaplan-Meier plot for the metastatic tumors in the SKCM (Skin Cutaneous Melanoma) dataset). The tumors are stratified by TLS-score obtained after applying our model to the normalized bulk RNA-seq data. The orange curve represents the high strata (fifth quantile), green curve the intermediate (third quantile) strata and gray the low (first quantile) strata. The p-value stems from a two-sided log-rank test where the null hypothesis that all patients have the same survival outcome.

Supplementary Table 1 | Intrapatient cluster comparison of ERBB2-expression

Results from a two-sided Mann-Whitney U Test, comparing normalized ERBB2 expression between clusters within the same patient.

Cluster comparison	patient	p-val	p_{adj}-val	p_{adj}-val < 0.05
cancer 1 vs cancer 2	A	1.46E-09	2.78E-09	Yes
cancer 1 vs cancer in situ	A	1.25E-19	5.94E-19	Yes
cancer 2 vs cancer in situ	A	3.13E-12	8.48E-12	Yes
cancer 3 vs cancer 2	C	5.96E-06	8.71E-06	Yes
cancer 3 vs cancer 1	C	2.26E-05	3.07E-05	Yes
cancer 2 vs cancer 1	C	1.52E-18	5.76E-18	Yes
cancer : B/plasma cells vs cancer 1	D	4.82E-07	7.64E-07	Yes
cancer : B/plasma cells vs cancer 2	D	5.00E-02	5.94E-02	No
cancer 1 vs cancer 2	D	1.89E-02	2.39E-02	Yes
cancer : immune rich vs cancer 2	E	3.54E-20	2.24E-19	Yes
cancer : immune rich vs cancer 1	E	5.35E-02	5.98E-02	No
cancer 2 vs cancer 1	E	6.24E-35	1.19E-33	Yes
cancer 1 vs cancer 2	F	5.46E-01	5.46E-01	No
cancer in situ : immune rich vs cancer 1	G	2.33E-26	2.21E-25	Yes
cancer in situ : immune rich vs cancer 2	G	4.33E-10	9.14E-10	Yes
cancer in situ : immune rich vs cancer : connective tissue	G	3.88E-15	1.23E-14	Yes
cancer 1 vs cancer 2	G	1.20E-01	1.27E-01	No
cancer 1 vs cancer : connective tissue	G	2.51E-11	5.96E-11	Yes
cancer 2 vs cancer : connective tissue	G	2.05E-08	3.54E-08	Yes

Supplementary Table 2 | Receptor Status

ER and PgR receptor status for all patients (A-H) used during the tumor classification. Only Patient B (bold) has positive PgR status.

Patient	ER	PgR
A	-	-
B	-	+
C	-	-
D	-	-
E	-	-
F	-	-
G	-	-
H	-	-

Supplementary Table 3| Probes and primers

Probes and primers that were used in the protocol. The sequences replacing "XXXXXX" for the Illumina PCR index primers are listed as Index 1-12.

Name	Sequence	Manufacturer
Ligation adapter, B′-5rApp-3ddC-DNA	[rApp]AGATCGGAAGAGCACACGTCTGAACTCCAGTCAC[ddC]	IDT
Second RT primer (aRNA primer)	GTGACTGGAGTTCAGACGTGTGCTCTTCCGA	IDT
PCR InPE 1.0	AATGATACGGCGACCACCGAGATCTACACTCTTTCCCTACACGACGCTCTTCCGATCT	IDT
PCR InPE 2.0	GTGACTGGAGTTCAGACGTGTGCTCTTCCGATCT	IDT
Illumina PCR index primers	CAAGCAGAAGACGGCATACGAGATXXXXXXGTGACTGGAGTTC	IDT
Index 1	CGTGAT	N/A
Index 2	ACATCG	N/A
Index 3	GCCTAA	N/A
Index 4	TGGTCA	N/A
Index 5	CACTGT	N/A
Index 6	ATTGGC	N/A
Index 7	GATCTG	N/A
Index 8	TCAAGT	N/A
Index 9	CTGATC	N/A
Index 10	AAGCTA	N/A
Index 11	GTAGCC	N/A
Index 12	TACAAG	N/A
Cy3 anti-A-short probe	(Cy3)AGATCGGAAGAGCGTCGTGT	IDT
Cy3 anti-frame probe	(Cy3)GGTACAGAAGCGCGATAGCAG	IDT

Supplementary Note 1 | Interferon response in the presence of Mø and T-cell interactions

While patient G was used in the main text to exemplify how a joint presence of certain Mø (Mø2:CXL10) and T-cell (T-cells:IFIT1) subsets spatially aligns with an interferon response, we were keen to investigate whether this was an isolated event or could be observed among more of our samples. To answer the above question, we used a joint spot-wise score (Methods) based on the cell type proportion values; the spot-wise scores were stratified by cluster identity (Supplementary Figure 19). Several clusters seemed to be enriched for this signal, for example: pBc3, pDc5, pEc1, pGc4 (the in situ cancer cluster discussed in the main text). Of note, all the four aforementioned clusters had at least one type I interferon related pathway listed among the set of enriched pathways associated with them. We also visualize the joint scores spatially in Supplementary Figure 20, where it is clear how the elevated signals align well with the spatial regions demarcating each cluster.

The presence of a type I pathways support our hypothesis that this colocalization is partially related to a type I interferon stimuli/response, but to add support to these claims we generated spatial spot-wise enrichment plots - similar to and using the same GO-term as in Figure 4E - which are show in Supplementary Figure 21. What can be deduced from these plots is that a high joint presence of the two cell type subsets very often aligns spatially with the presence of a type I interferon pathway, but that the opposite is not always true. This is to some extent expected since type I interferon signals and responses are by no means restricted to these two cell types. Furthermore, we cannot pinpoint the likeliest source of the type I interferon response inducing stimuli (e.g., IFNα or IFNβ) due to low detection of the type I interferon transcripts in this dataset

To further validate our findings, we asked whether the trifold colocalization between Mø, T-cells and a type I interferon response could be discerned in two additional external data sets. First, we investigated the "*Human Breast Cancer (Block A Section 1)*" data set from 10x Genomics^{TM} website, subtyped as HER2-positive; we will refer to this sample as Visium10x. We mapped the single cell data set from Wu et al. used in the main text onto the Visium10x sample using *stereoscope* (Methods), to obtain proportion estimates for each cell type. We found that the cell states T-cell:IFIT1 and Mø:CXCL10 shared similar distributions (Supplementary Figure 22A). Analogous to our analysis in

the main text, we highlighted regions where the two cell states (T-cells:IFIT1 and Mø2:CXCL10) overlap by multiplying their proportion values in each spot; again, these regions aligned with spots enriched for a type I interferon response pathway (GO:0060337) (Supplementary Figure 22B). As in the main data set, the Pearson correlation between Mø2:CXCL10 and T-cell:IFIT showed a similarly increased signal. (Supplementary Figure 22C).

These results are encouraging as they show how the same colocalization patterns between the cell states and the interferon response signal are present in a HER2-positive breast cancer data set from: (i) an external lab (i.e., not produced by any of the authors of this manuscript) and (ii) on a different platform than what we are using (Spatial Transcriptomics 1k vs Visium). To investigate whether this phenotype could be observed in another cancer type we sought to use a second pair of data sets independent from ours. Thus, we downloaded spatial transcriptomics data (Spatial Transcriptomics 2K and Visium, n = 12 sections) and single cell data collected from Squamous Cell Carcinoma (SCC) tissue samples.[3] We were particularly interested in this data set as the authors explicitly speak of an interaction between T-cells and Mø (expressing CXCL9/10/11), and also note the presence of a type I interferon response in their data, but do not link these to each other. To be noted, no Mø subset characterized by elevated levels of CXCL9/10/11 was explicitly defined by the authors in this data set, nor any T-cell subset with an interferon related expression profile. However, a large proportion of the Mø cells expressed CXCL9/10/11 (see Ji.et.al Figure 4F)[3], and an interferon enriched T-cell subset could be present but with too few members to constitute their own cluster. *stereoscope* was used to map the SCC single cell data onto the spatial transcriptomics data (Methods). Supplementary Figure 23A shows T-cell and Mø cell types proportion values, where an overlap was observed. We performed identical analyses as for the Visium10x sample, computing the product between Mø and T-cells to expose regions where with elevated abundance of both types, to then compared the joint Mø and T-cell enrichment with the interferon related pathway enrichment of the type I interferon response related pathway (Supplementary Figure 23B). We also computed Pearson correlation (Methods) between Mø and all remaining cell types, where positive signals with the T-cells indicate a tendency to colocalize (Supplementary Figure 23C). Colocalization between T-cells and Mø, of varying strength, were present among all patients in the data set (Supplementary Figure 23D).

This second analysis does not only show reproducibility with a different spatial platform and a different experimental batch, but also across disease, which we consider as strong support for a mechanism involving certain T-cell and Mø

states together with a type I interferon response. It also confirms that the signal is not an artifact of the single cell data set that we are using in the main analysis. However, we can yet only speculate about the mechanism of this interaction, and further - more specific and targeted - studies are required to delineate this.

Supplementary Note 2 | Prediction of TL-like structures in external data

Having established good performance for our predictive model across techniques when applied to breast cancer data we wanted to assess its behavior when presented with other types of tissue. Thus we predicted TLS-score in samples from three different tissues: rheumatoid arthritis (RA), melanoma and (as a negative control) developmental heart (Supplementary Figure 25). Regions with high TLS-score in RA tissue overlapped with the areas annotated as immune infiltrates in the original publication, where they also discuss the likely presence of ectopic lymphoid structures (in this context, synonymous to TLS).[2] A similar trend was observed in the melanoma sample, where high TLS-scores were predicted in regions annotated as immune infiltrates.[1] In the healthy developmental heart, no signals above zero were observed for the predicted TLS-score, suggesting absence of TLSs, as expected in this tissue.[4,5]

Supplementary Note 3 | Reproducing previous studies of overall survival and presence of TLSs

In a previous study presented by Cabrita et.al it was shown how TLS-signal strength could be related to survival outcome among patients diagnosed with metastatic melanoma.[6] We thus reasoned that if our signature was generalizable and characteristic of TL-like structures it should be able to reproduce these results at least to some extent. After accessing the relevant data (SKCM data set from TCGA), we normalized the expression data, applied our model and stratified the samples into three tiers (high, intermediate and low) based on their TLS-score (Methods). Similar to Cabrita el al. we saw a clear separation of the “high” stratum from the “low” and “intermediate” strata, and obtained a (log-rank) p-value of 0.0077 when testing whether the samples come from populations with identical survival curves or not (Supplementary Figure 26). The generated Kaplan-Meier plot is highly similar in character to what Cabrita et.al. present in their Figure 3B, not included here due to copyright restrictions.

Supplementary References

1. Thrane, K., Eriksson, H., Maaskola, J., Hansson, J. & Lundeberg, J. Spatially Resolved Transcriptomics Enables Dissection of Genetic Heterogeneity in Stage III Cutaneous Malignant Melanoma. *Cancer Res.* **78**, 5970–5979 (2018).
2. Carlberg, K. *et al.* Exploring inflammatory signatures in arthritic joint biopsies with Spatial Transcriptomics. *Sci. Rep.* **9**, 18975 (2019).
3. Ji, A. L. *et al.* Multimodal Analysis of Composition and Spatial Architecture in Human Squamous Cell Carcinoma. *Cell* **182**, 497–514.e22 (2020).
4. Asp, M. *et al.* A Spatiotemporal Organ-Wide Gene Expression and Cell Atlas of the Developing Human Heart. *Cell* **179**, 1647–1660.e19 (2019).
5. Colbeck, E. J., Ager, A., Gallimore, A. & Jones, G. W. Tertiary Lymphoid Structures in Cancer: Drivers of Antitumor Immunity, Immunosuppression, or Bystander Sentinels in Disease? *Front. Immunol.* **8**, 1830 (2017).
6. Cabrita, R. *et al.* Tertiary lymphoid structures improve immunotherapy and survival in melanoma. *Nature* **577**, 561–565 (2020).
