# Supplementary material for: Spatial deconvolution of HER2-positive breast cancer delineates tumor-associated cell type interactions
Source: Nat Commun. 2021 Oct 14;12:6012. doi: 10.1038/s41467-021-26271-2 (PMC8516894; doi:10.1038/s41467-021-26271-2)
Supplement: Supplementary file 3 — Description of Additional Supplementary Files [file 41467_2021_26271_MOESM3_ESM.pdf]

Description of Additional Supplementary Files

File Name: Supplementary Data 1
Description: marker genes for each of the expression based clusters (within each patient). Statistical test: two-sided Mann-Whitney U test. Multiple testing correction method: Bonferroni.

File Name: Supplementary Data 2
Description: Cluster annotations together with their motivations.

File Name: Supplementary Data 3
Description: Enrichment of expression-based clusters and the regions defined by the pathologist. Each sheet gives information for one section, the specific section is indicated by the sheet name.

File Name: Supplementary Data 4
Description: Enriched pathways for each of the expression based clusters. Each sheet holds information for one patient, as indicated by the sheet name. P-values are derived from a hypergeometric test and the method for multiple testing correction was gSCS.

File Name: Supplementary Data 5
Description: Coefficient values for the genes included in the linear model fitted to predict TLS-score. The genes included in the TLS-signature (total 171 genes) are also listed in a separate sheet together with their associated coefficient values.

File Name: Supplementary Data 6
Description: genes included in each of the respective core signatures (2 immune associated and 1 tumor associated)

File Name: Supplementary Data 7
Description: Visualization of proportion estimates across all tiers and samples. Tier and sample are indicated by the title of each page.

File Name: Supplementary Data 8
Description: Proportion estimates across all tiers and samples. Sheet naming follows the convention: [tier]_[patient][replicate].

File Name: Supplementary Data 9
Description: enrichment/depletion results for all cell types across all tiers. Annotation type, tier, and patient id are indicated by the page titles.

File Name: Supplementary Data 10
Description: Correlation matrices across all three tiers and sections. Tier and section are indicated by the page titles.

File Name: Supplementary Data 11
Description: The list of genes excluded in parts of the analysis due to their aberrant behavior, i.e., forming ring-like patterns in several capture areas.

File Name: Supplementary Data 12

Description: The pathways obtained when subjecting the TLS-signature to functional enrichment analysis (Database: GO:BP).

File Name: Supplementary Data 13
Description: The set of genes used in the stereoscope analysis.
