# Supplementary material for: Spatial deconvolution of HER2-positive breast cancer delineates tumor-associated cell type interactions
Source: Nat Commun. 2021 Oct 14;12:6012. doi: 10.1038/s41467-021-26271-2 (PMC8516894; doi:10.1038/s41467-021-26271-2)
Supplement: Supplementary file 10 — Supplementary Data 7 [file 41467_2021_26271_MOESM10_ESM.pdf]

major_A3

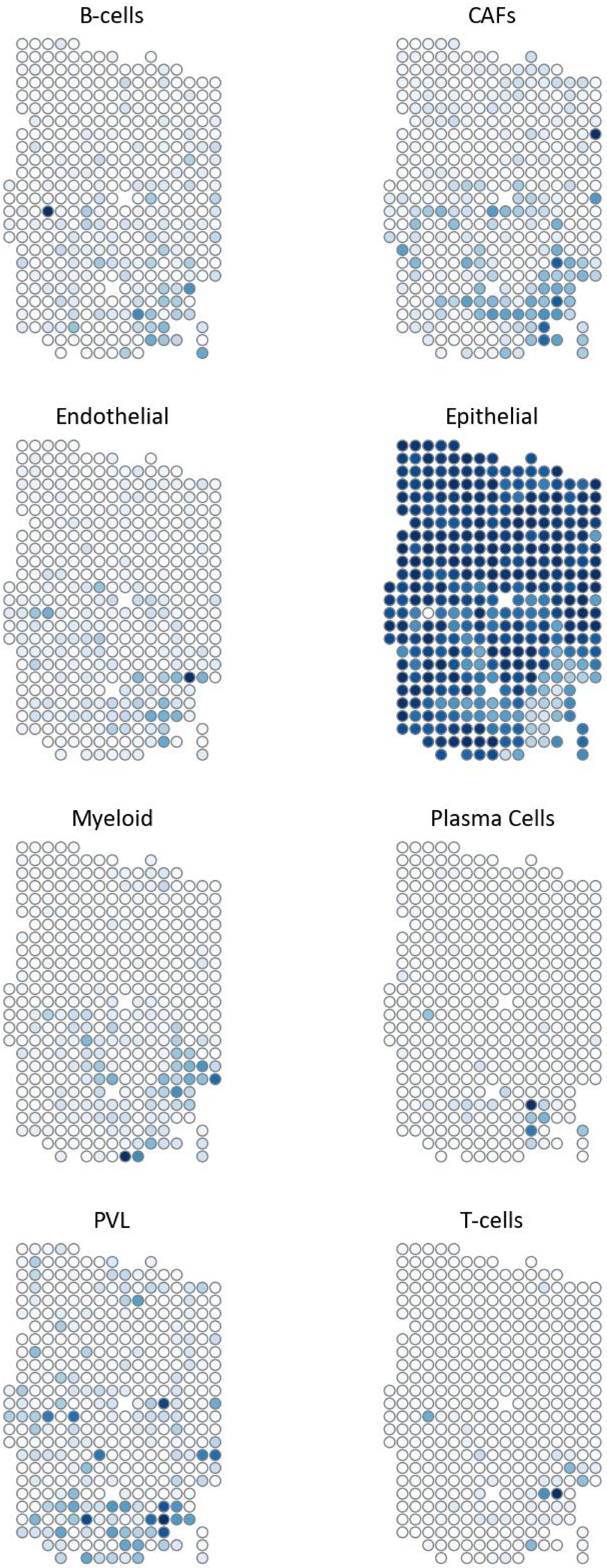
B-cells
CAFs
Endothelial
Epithelial
Myeloid
Plasma Cells
PVL
T-cells

major_A6

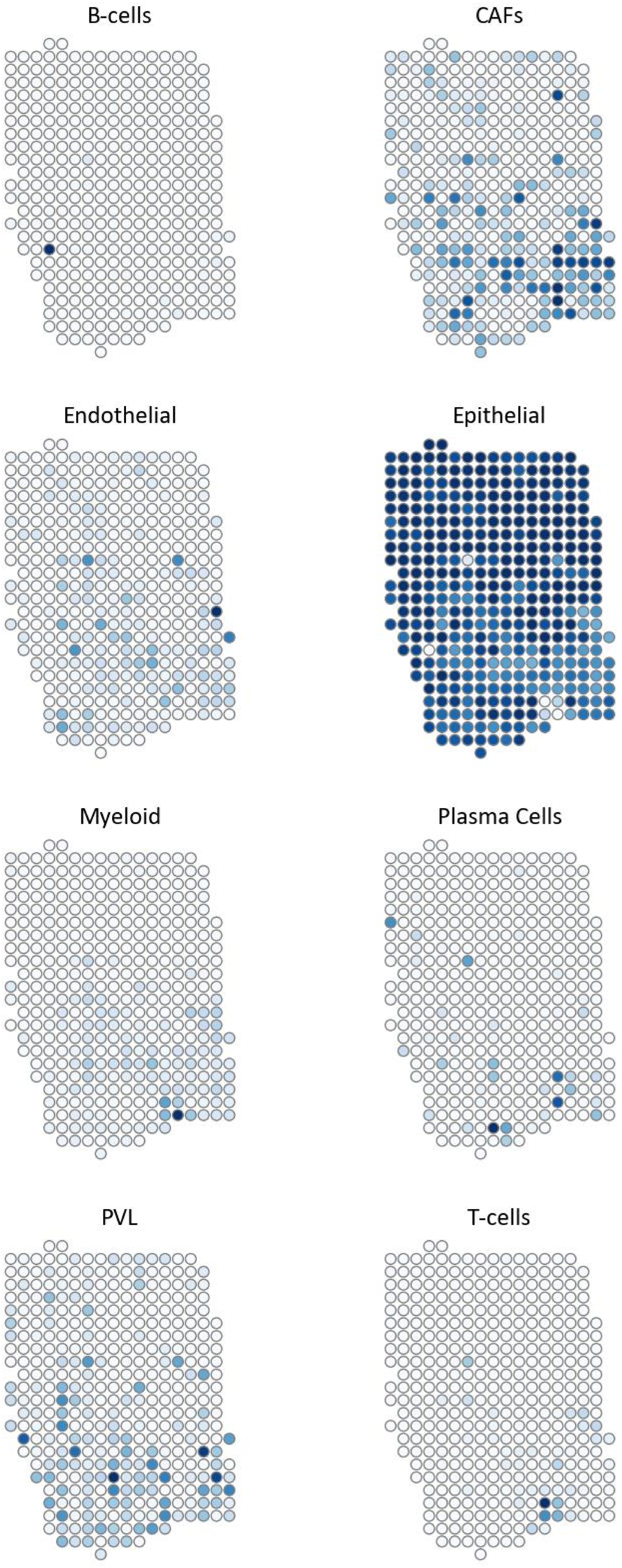

B-cells
CAFs
Endothelial
Epithelial
Myeloid
Plasma Cells
PVL
T-cells

major_G3

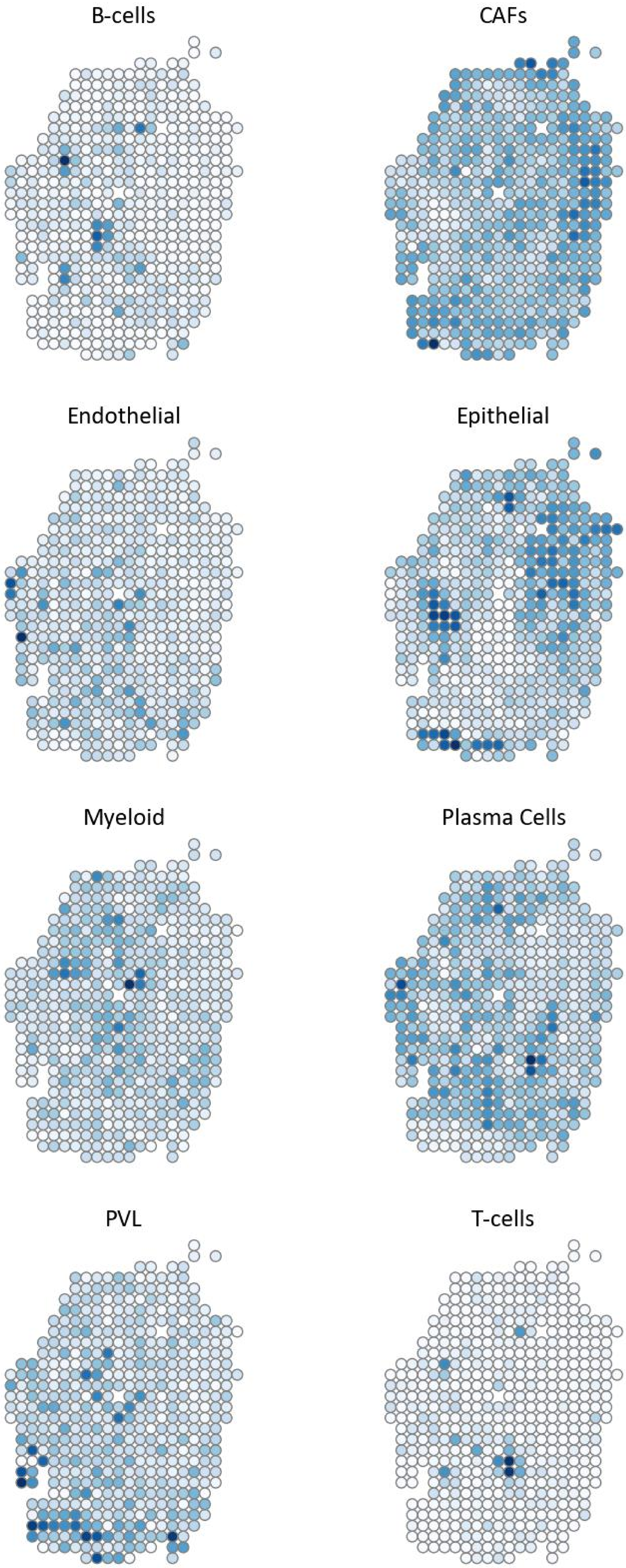
B-cells
CAFs
Endothelial
Epithelial
Myeloid
Plasma Cells
PVL
T-cells

major_D2

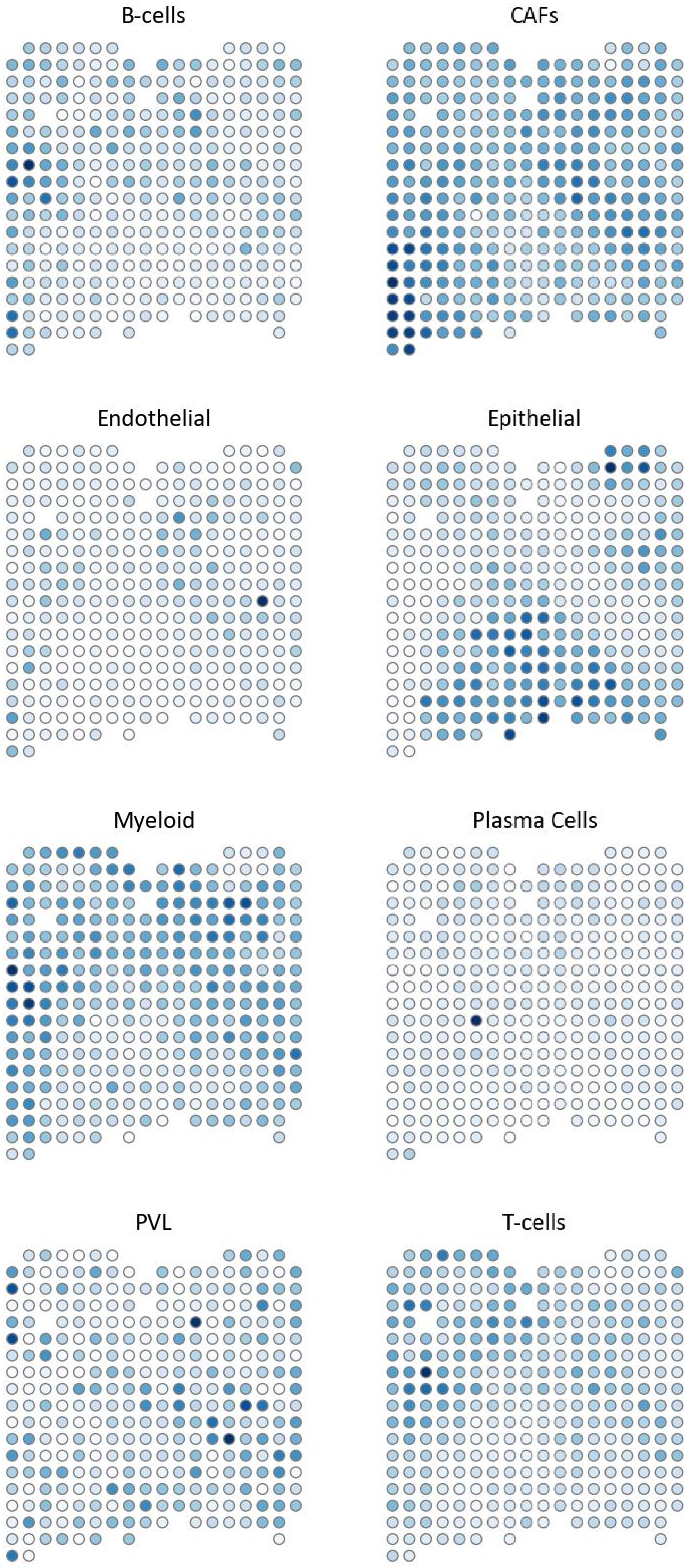
B-cells
CAFs
Endothelial
Epithelial
Myeloid
Plasma Cells
PVL
T-cells

major_D4

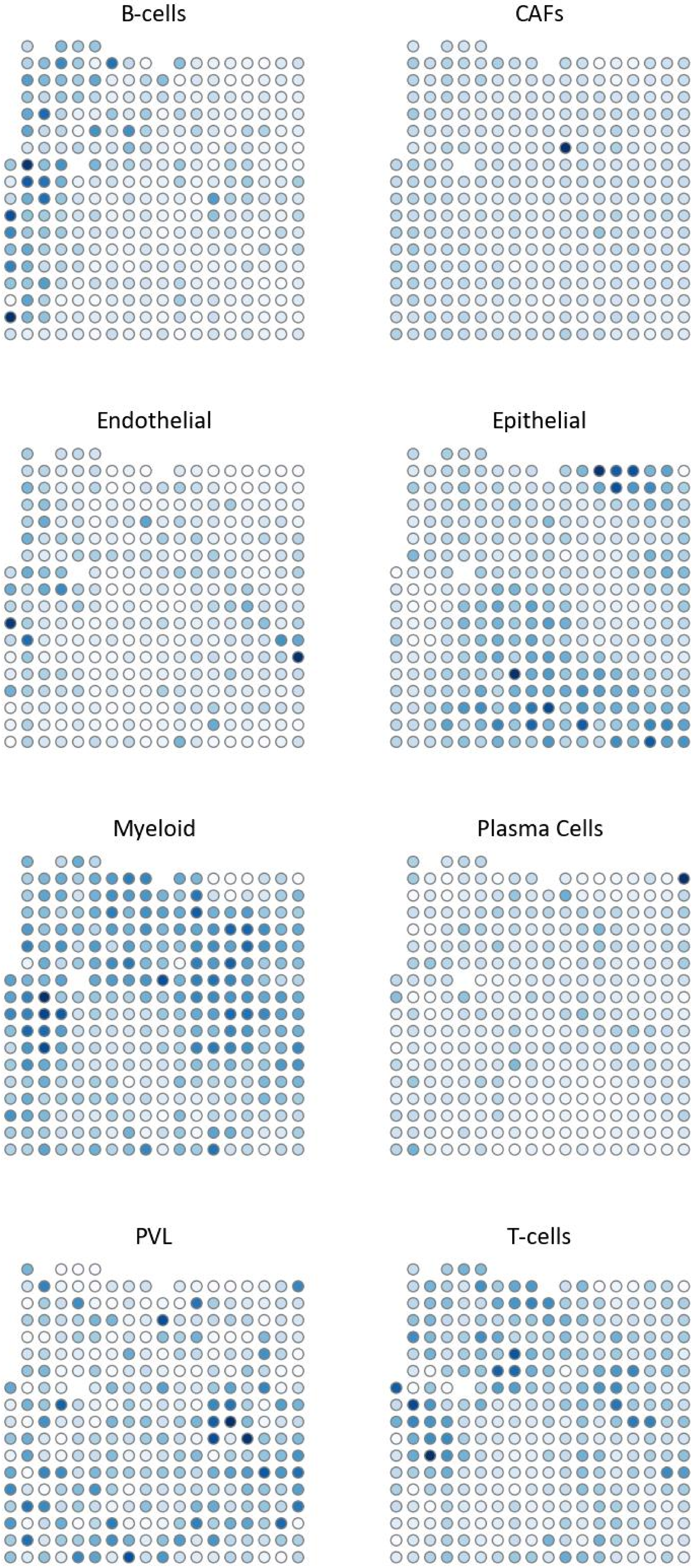
B-cells
CAFs
Endothelial
Epithelial
Myeloid
Plasma Cells
PVL
T-cells

major_C5

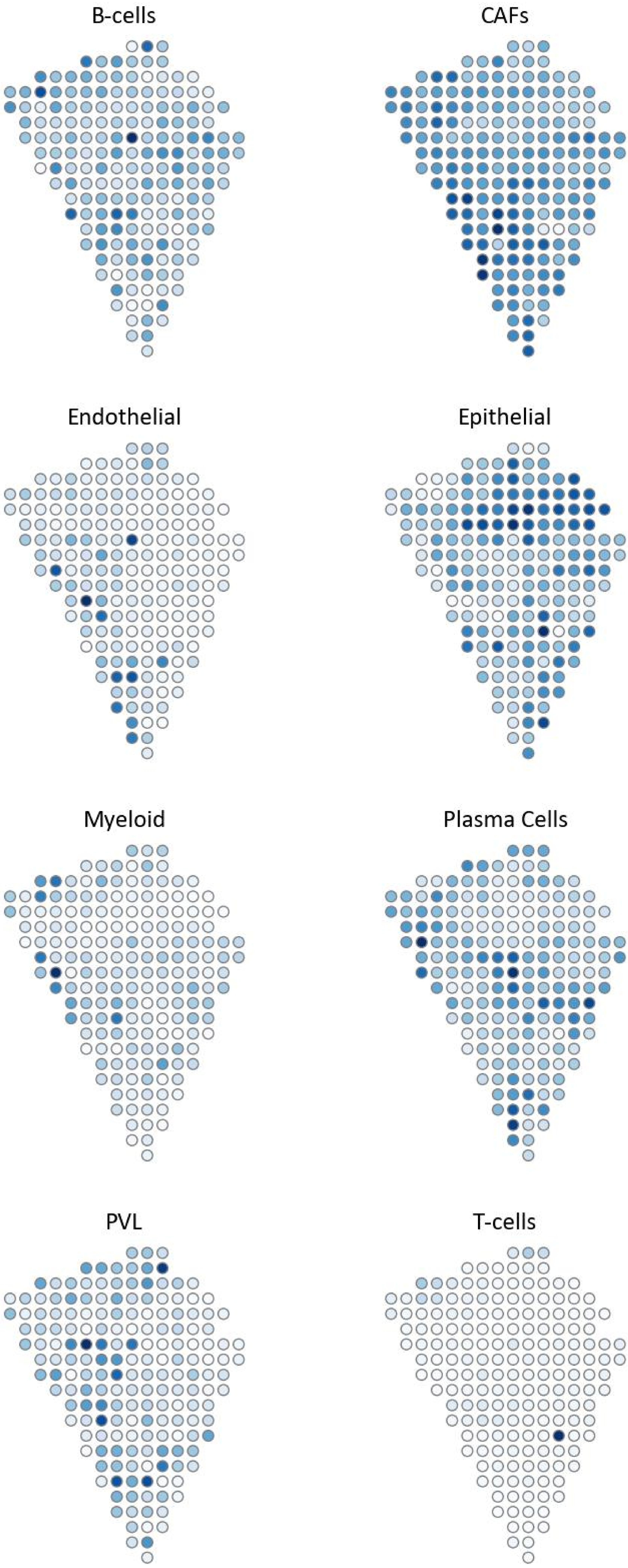
B-cells
CAFs
Endothelial
Epithelial
Myeloid
Plasma Cells
PVL
T-cells

major_E3

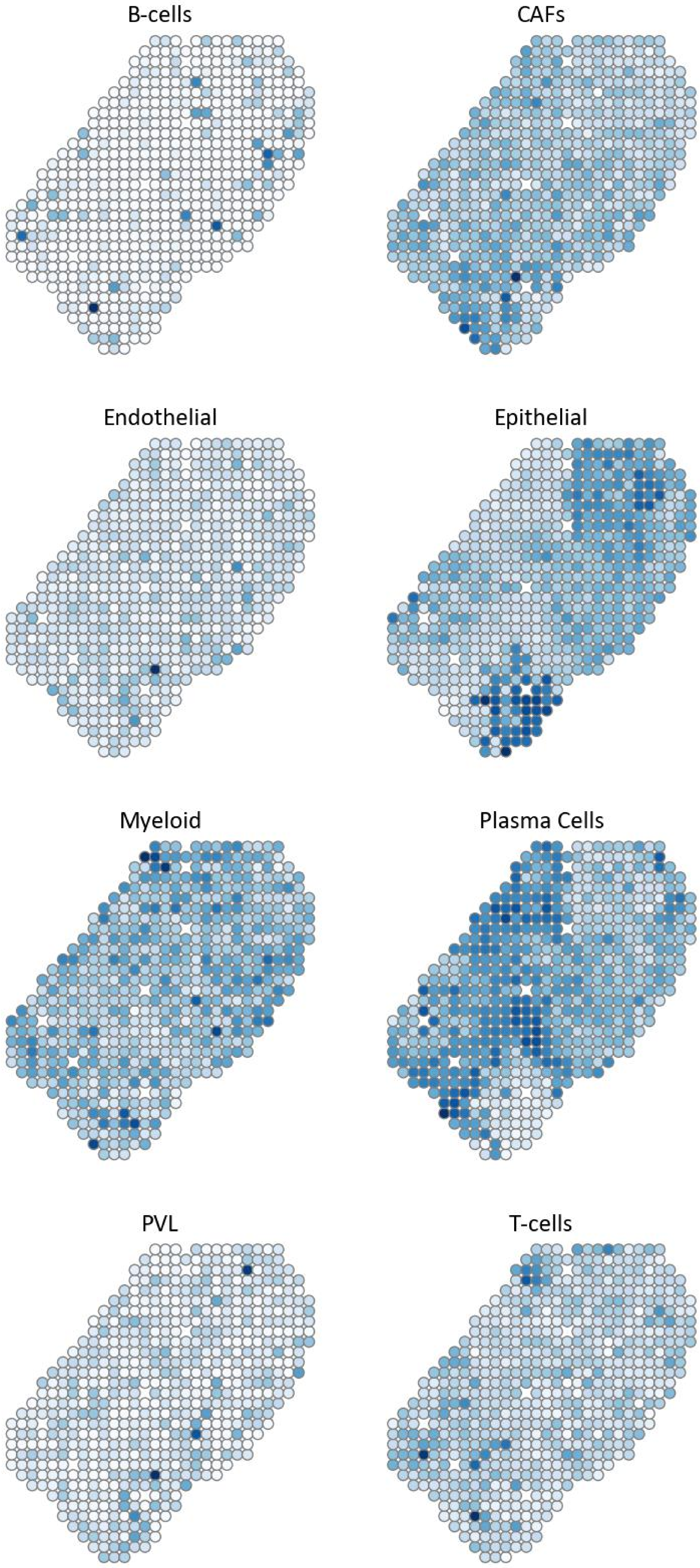

B-cells
CAFs
Endothelial
Epithelial
Myeloid
Plasma Cells
PVL
T-cells

major_G1

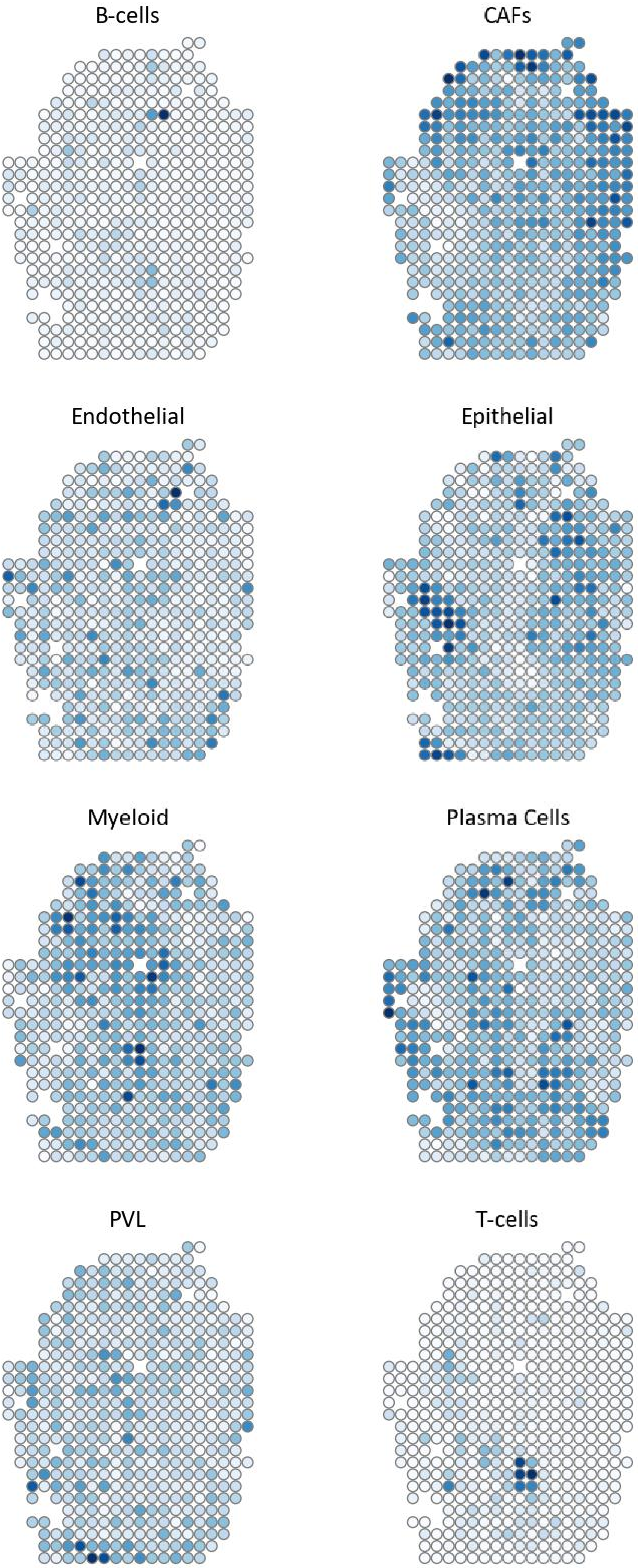
B-cells
CAFs
Endothelial
Epithelial
Myeloid
Plasma Cells
PVL
T-cells

major_F1

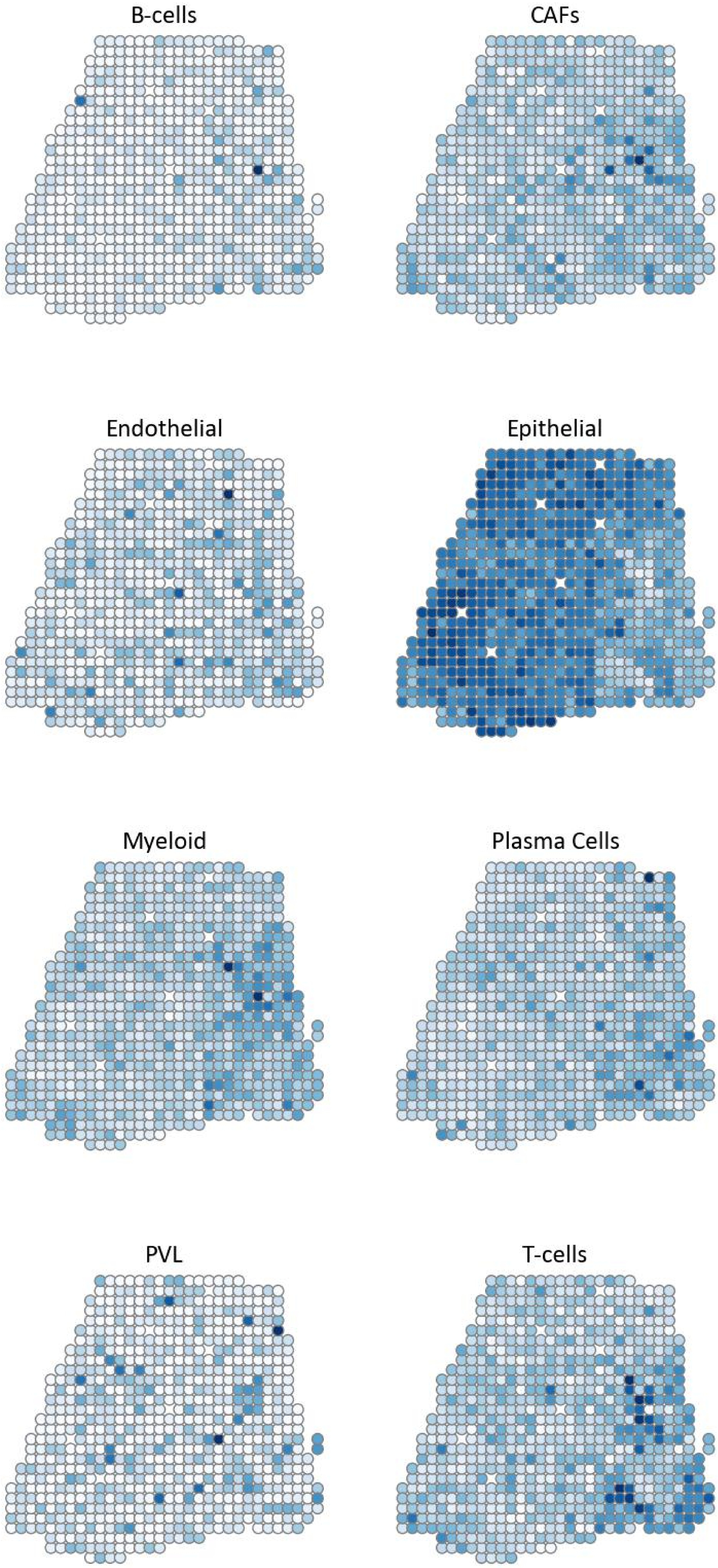
B-cells
CAFs
Endothelial
Epithelial
Myeloid
Plasma Cells
PVL
T-cells

major_H2

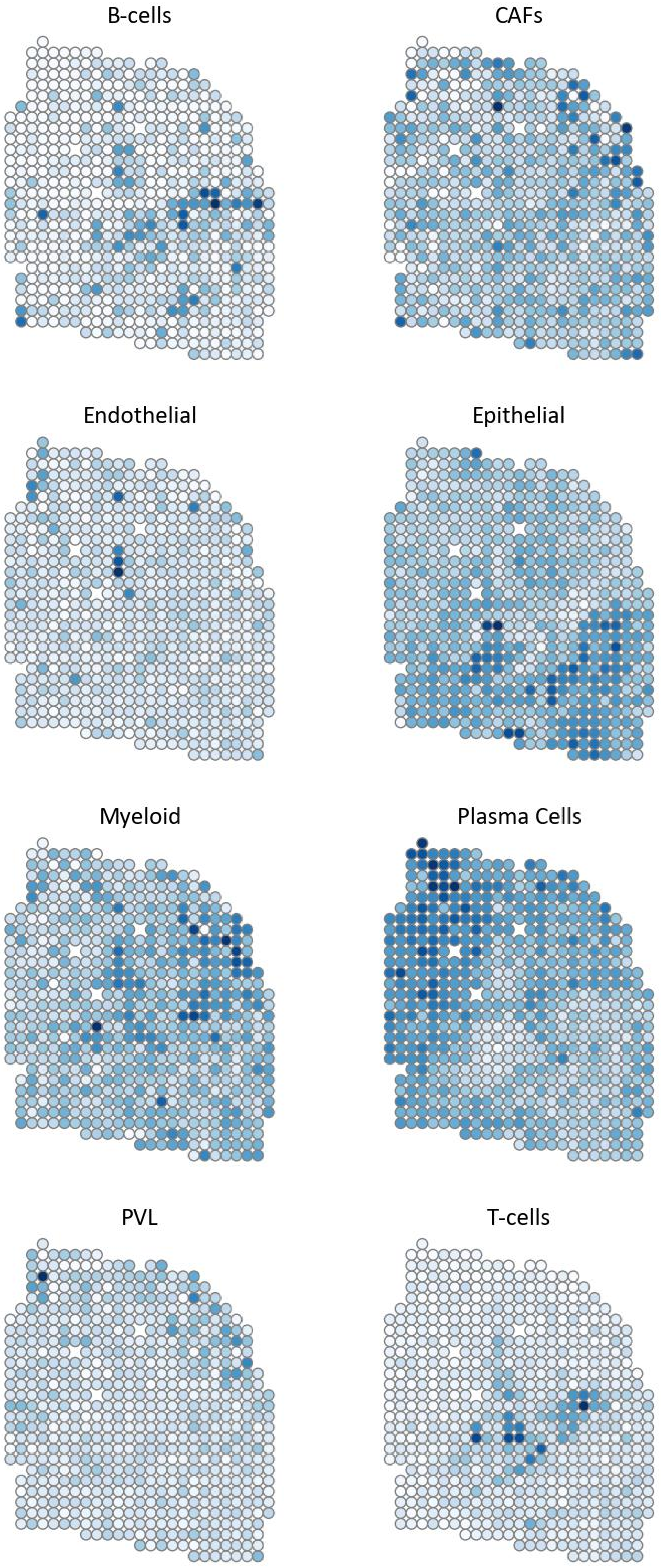
B-cells
CAFs
Endothelial
Epithelial
Myeloid
Plasma Cells
PVL
T-cells

major_B1

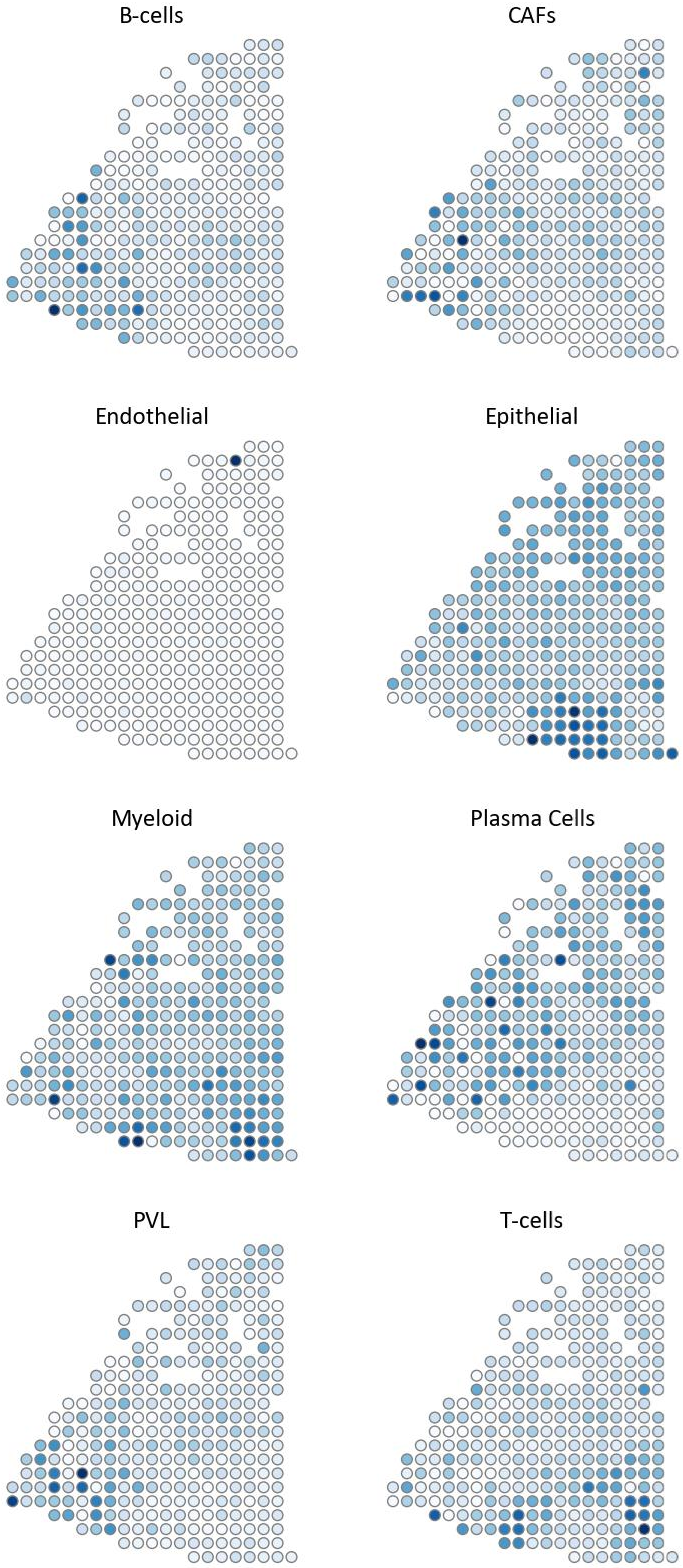
B-cells
CAFs
Endothelial
Epithelial
Myeloid
Plasma Cells
PVL
T-cells

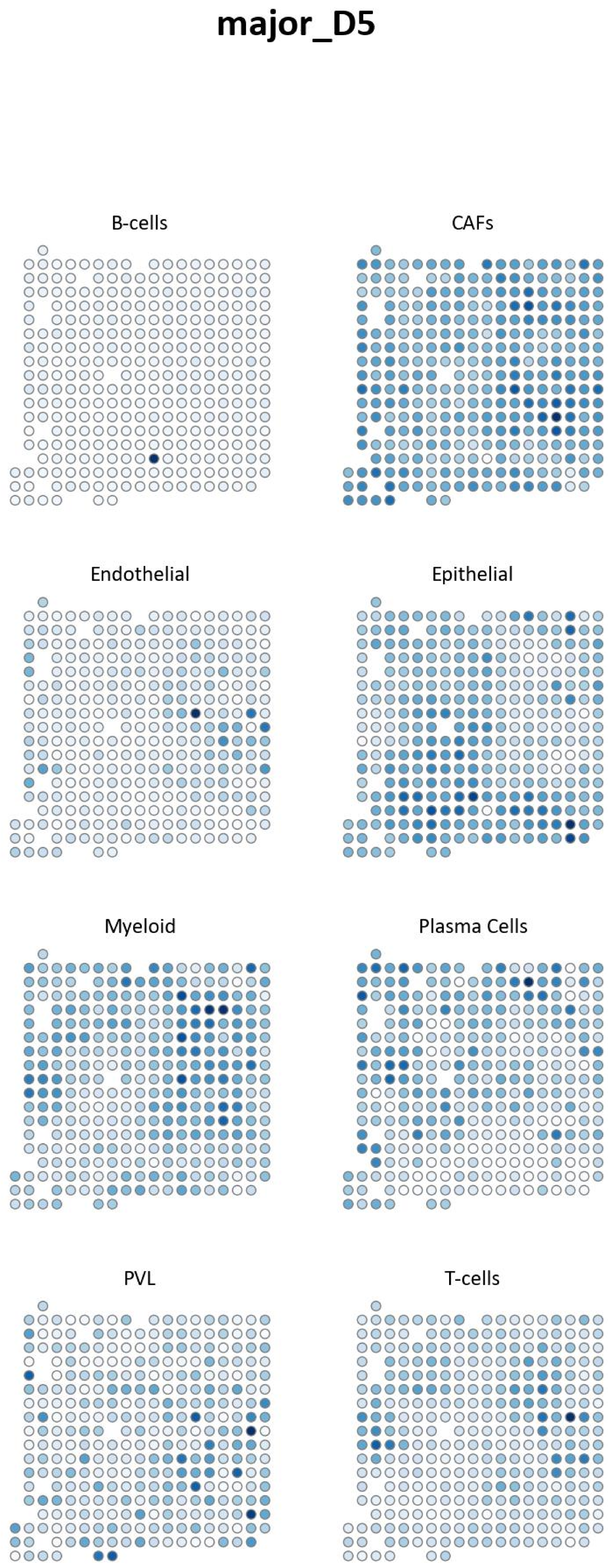

major_D5
B-cells
CAFs
Endothelial
Epithelial
Myeloid
Plasma Cells
PVL
T-cells

major_B5

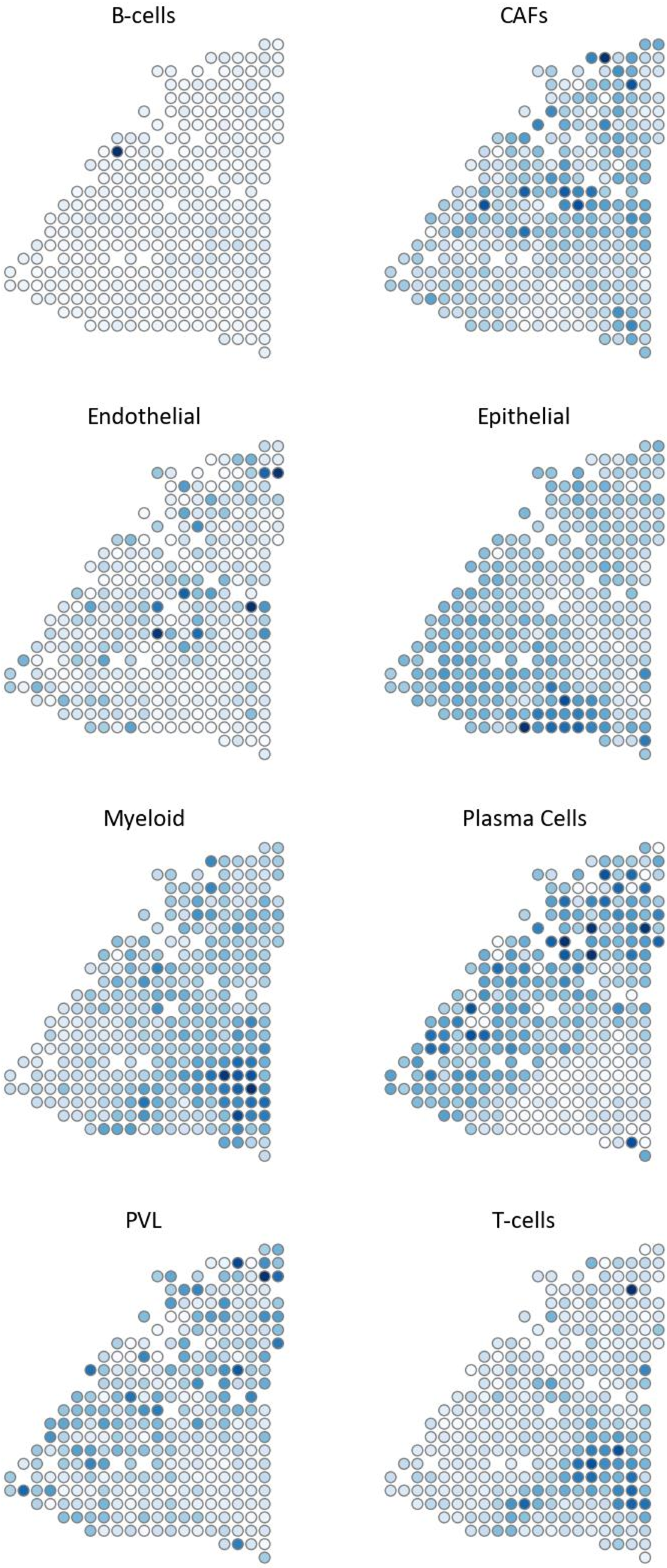
B-cells
CAFs
Endothelial
Epithelial
Myeloid
Plasma Cells
PVL
T-cells

major_B6

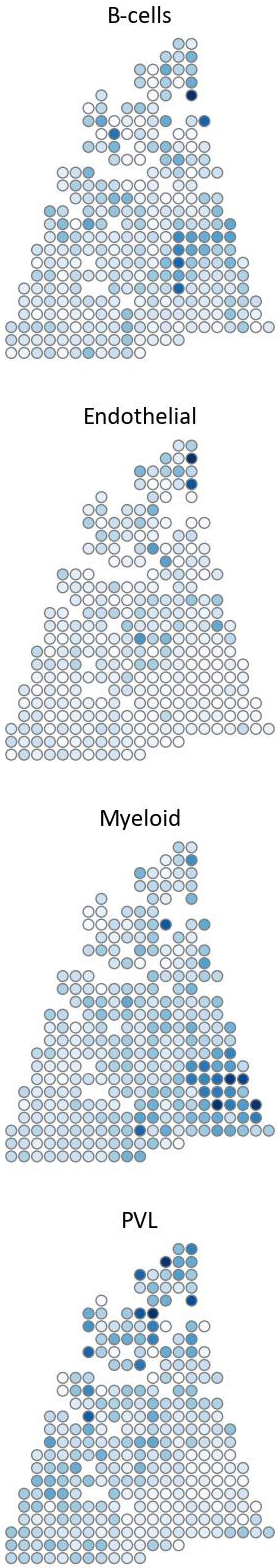
B-cells
CAFs
Endothelial
Epithelial
Myeloid
Plasma Cells
PVL
T-cells

major_A2

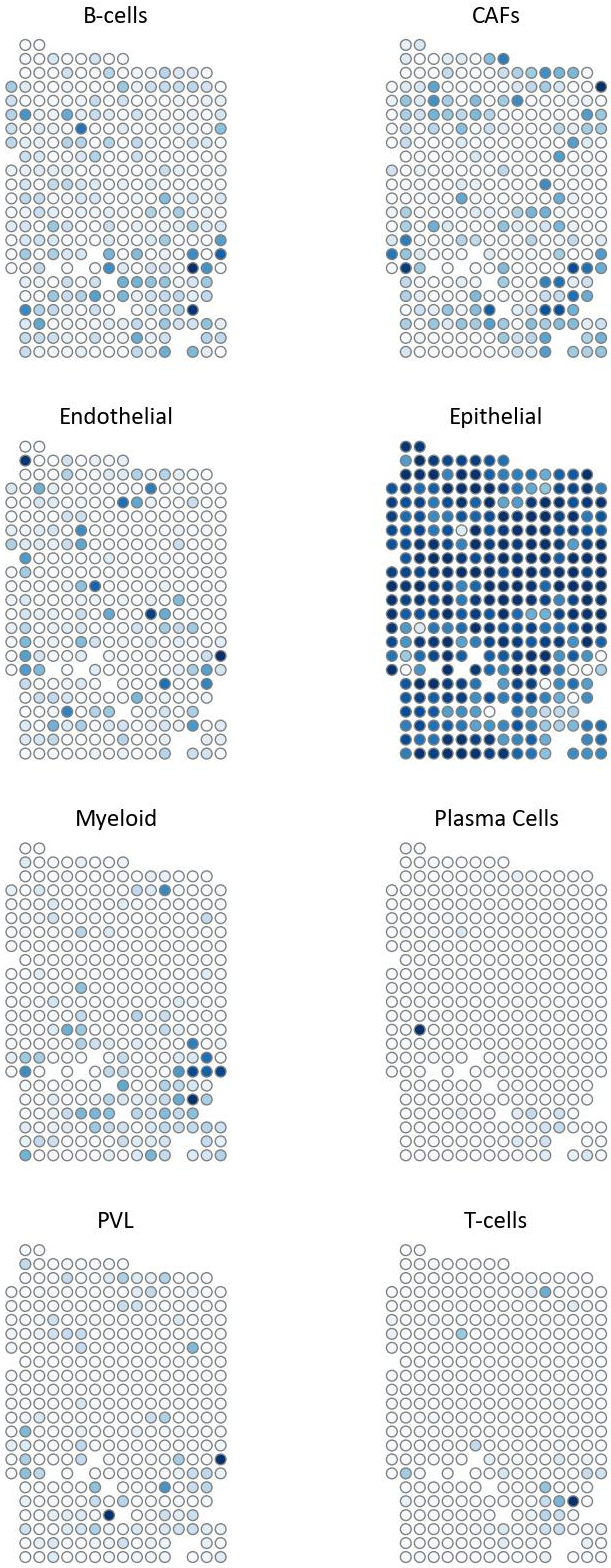

B-cells
CAFs
Endothelial
Epithelial
Myeloid
Plasma Cells
PVL
T-cells

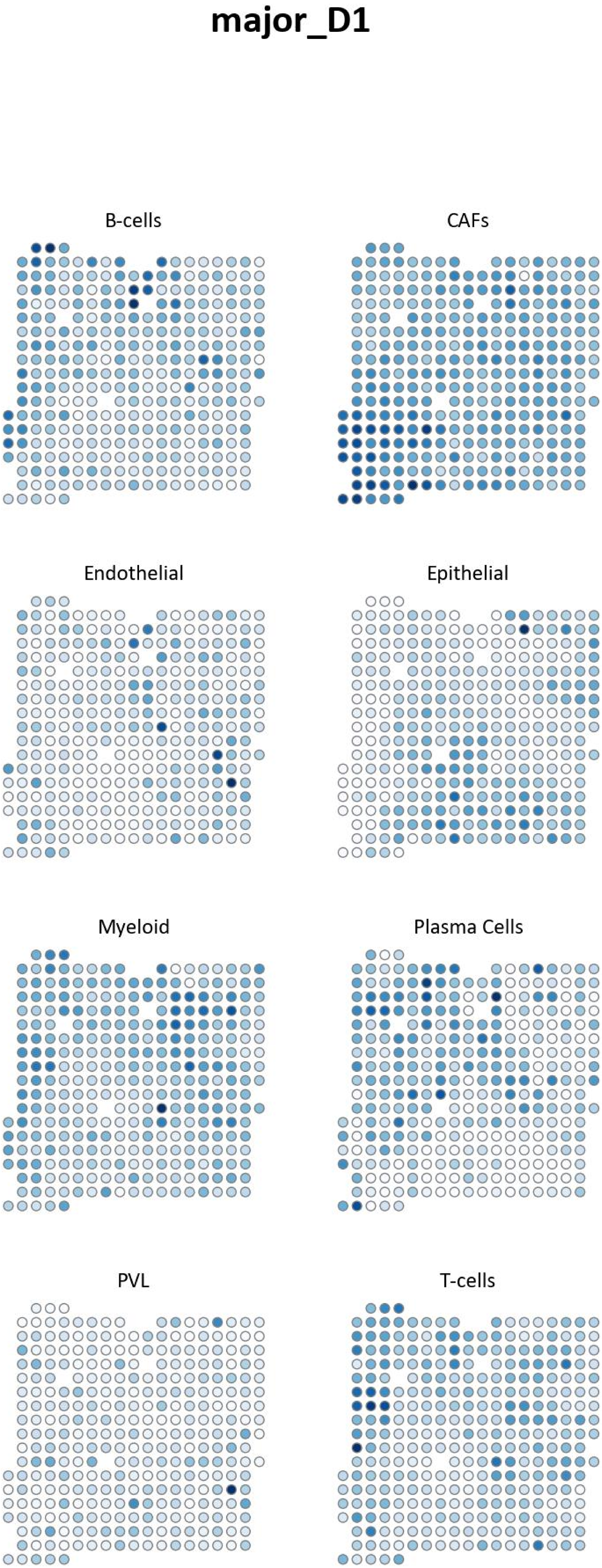
major_D1
B-cells
CAFs
Endothelial
Epithelial
Myeloid
Plasma Cells
PVL
T-cells

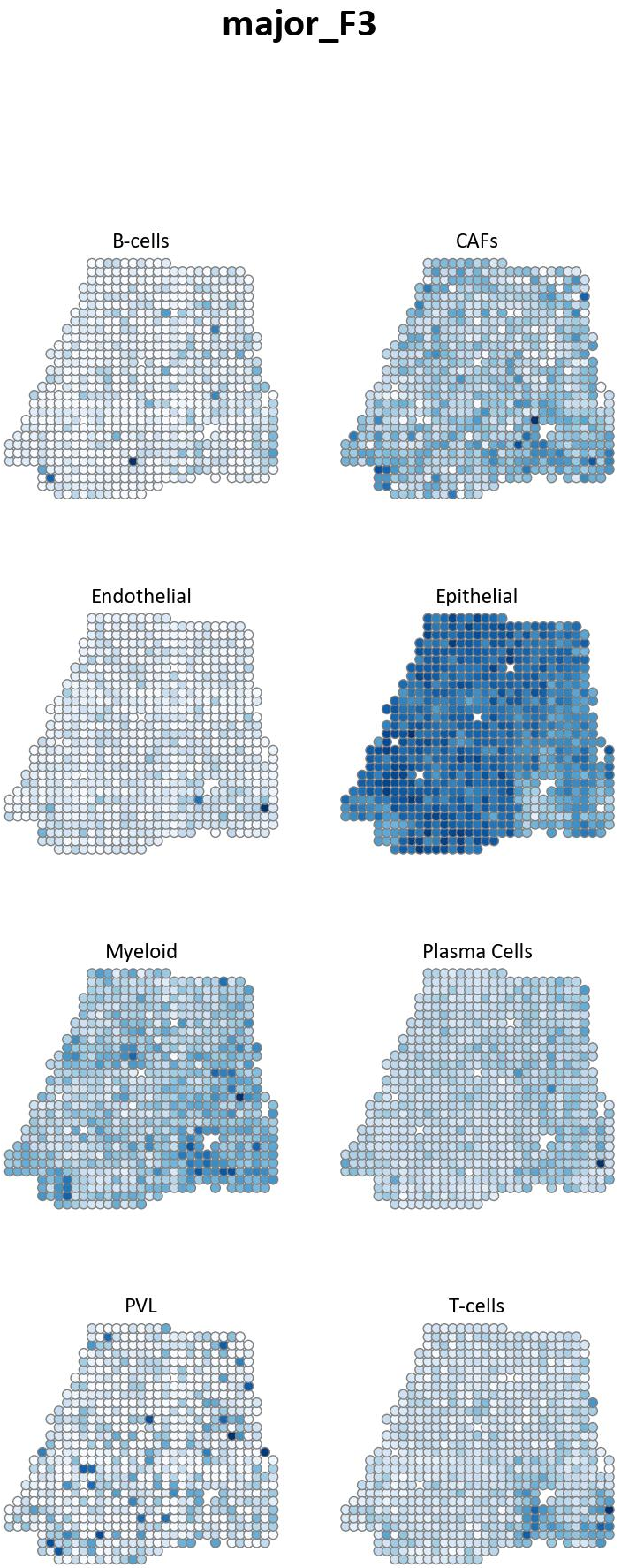
major_F3
B-cells
CAFs
Endothelial
Epithelial
Myeloid
Plasma Cells
PVL
T-cells

major_A1

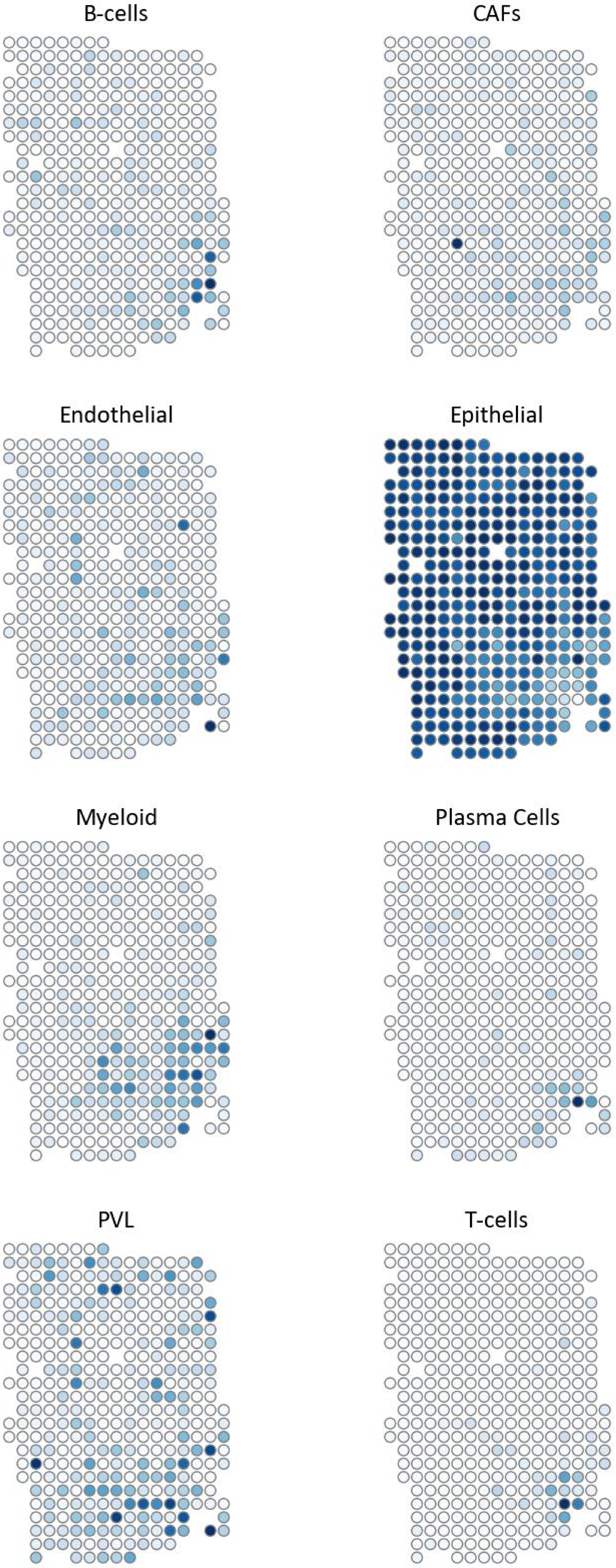
B-cells
CAFs
Endothelial
Epithelial
Myeloid
Plasma Cells
PVL
T-cells

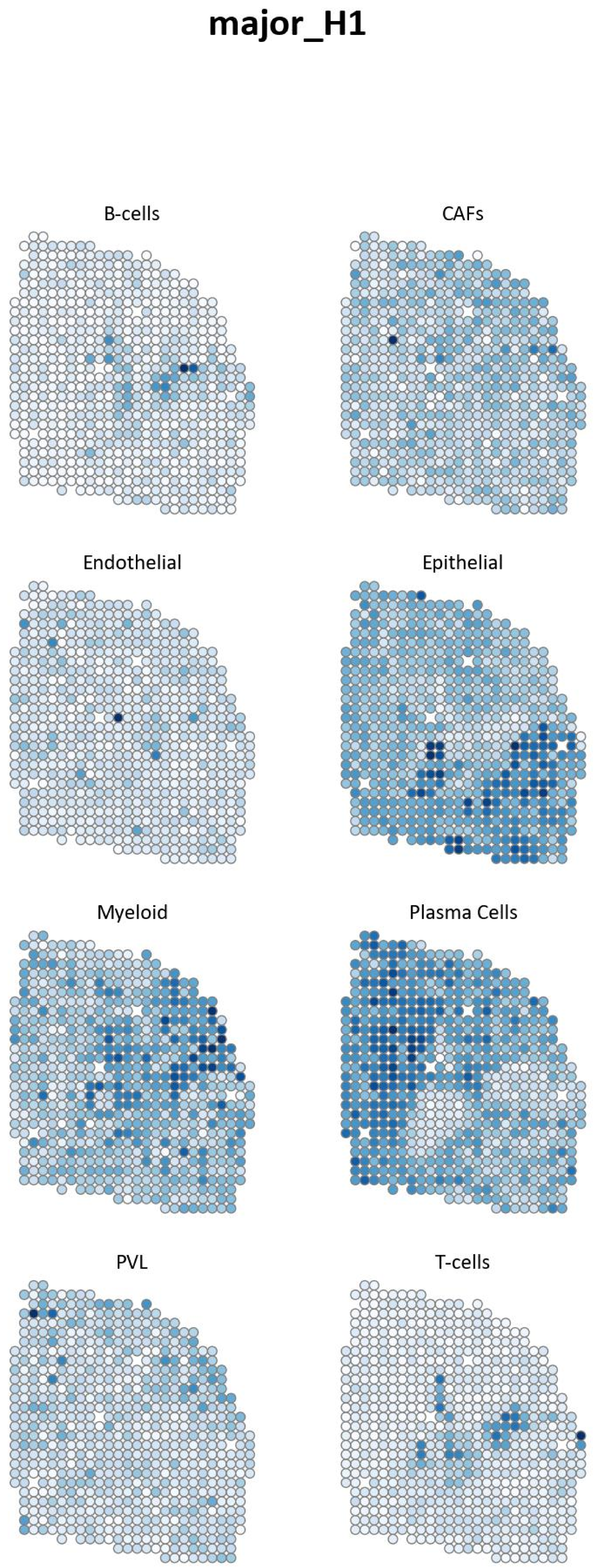
major_H1
B-cells
CAFs
Endothelial
Epithelial
Myeloid
Plasma Cells
PVL
T-cells

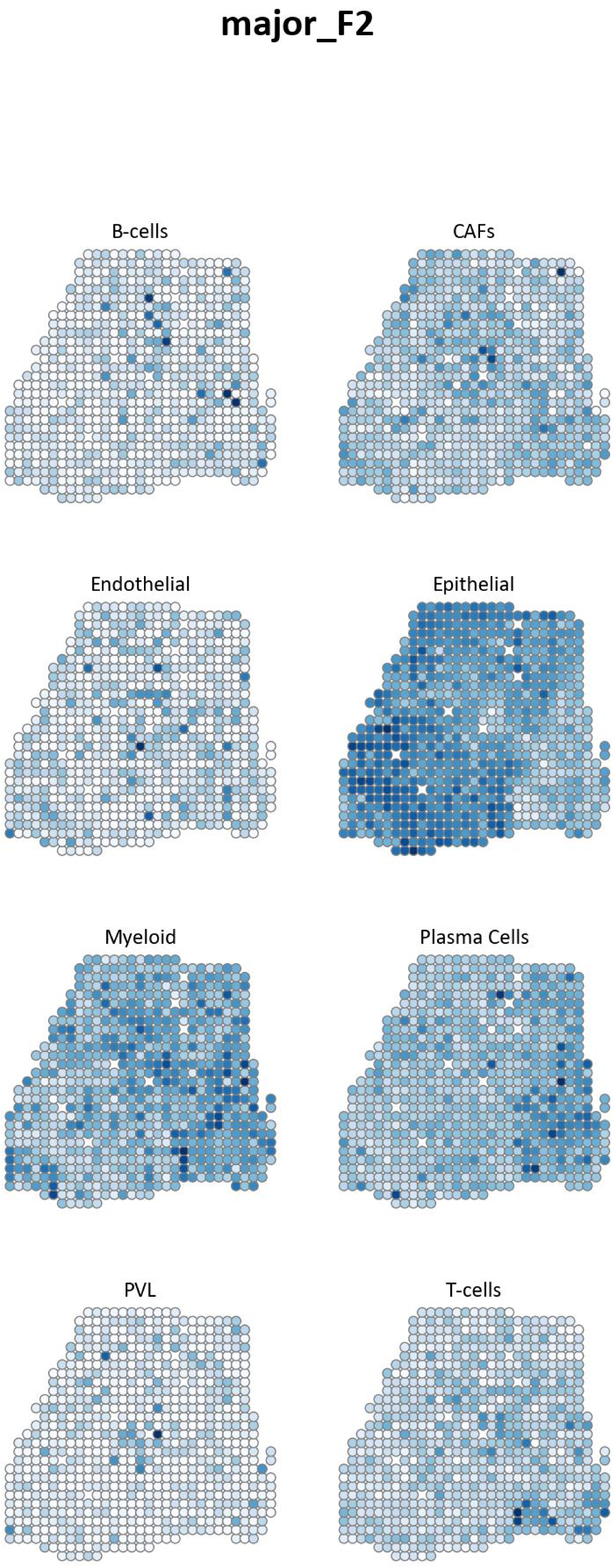
major_F2
B-cells
CAFs
Endothelial
Epithelial
Myeloid
Plasma Cells
PVL
T-cells

major_E2

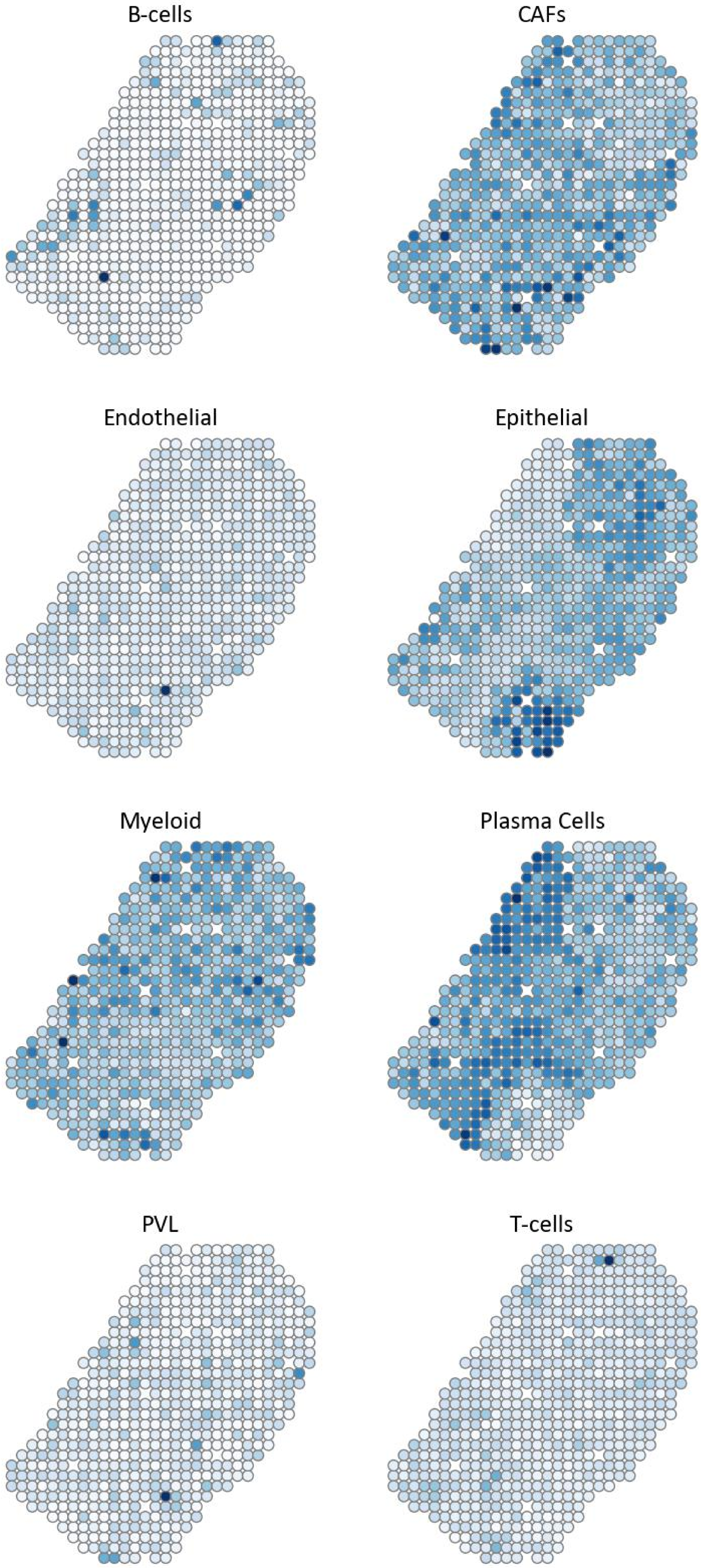
B-cells
CAFs
Endothelial
Epithelial
Myeloid
Plasma Cells
PVL
T-cells

major_C4

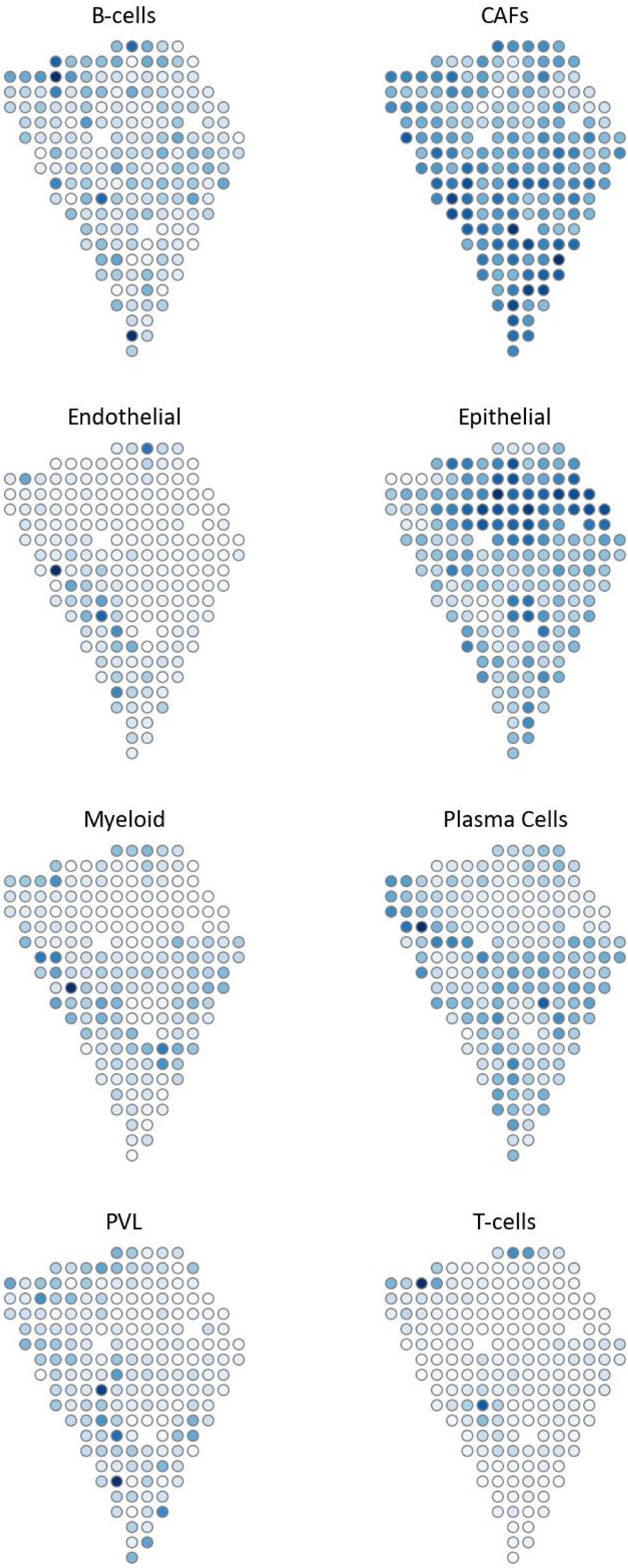
B-cells
CAFs
Endothelial
Epithelial
Myeloid
Plasma Cells
PVL
T-cells

major_D6

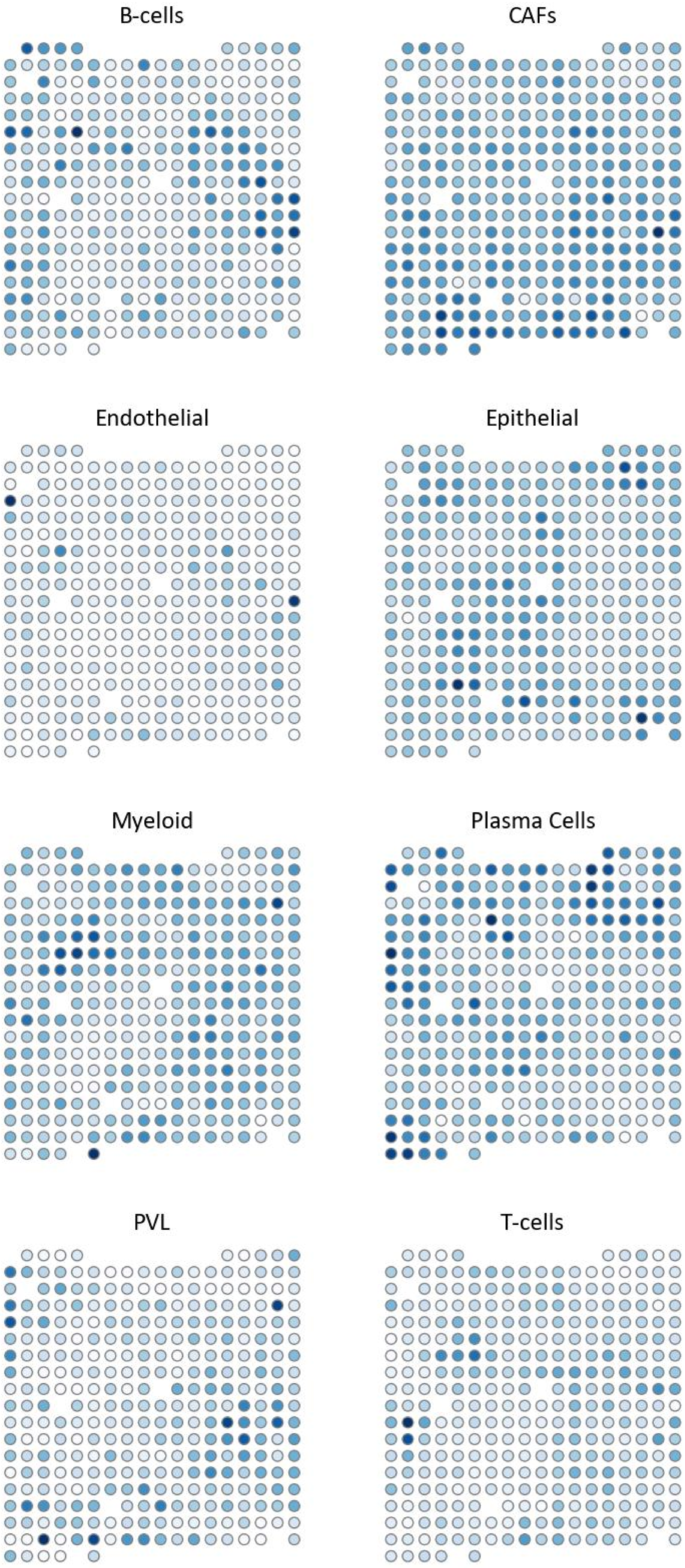

B-cells
CAFs
Endothelial
Epithelial
Myeloid
Plasma Cells
PVL
T-cells

major_C3

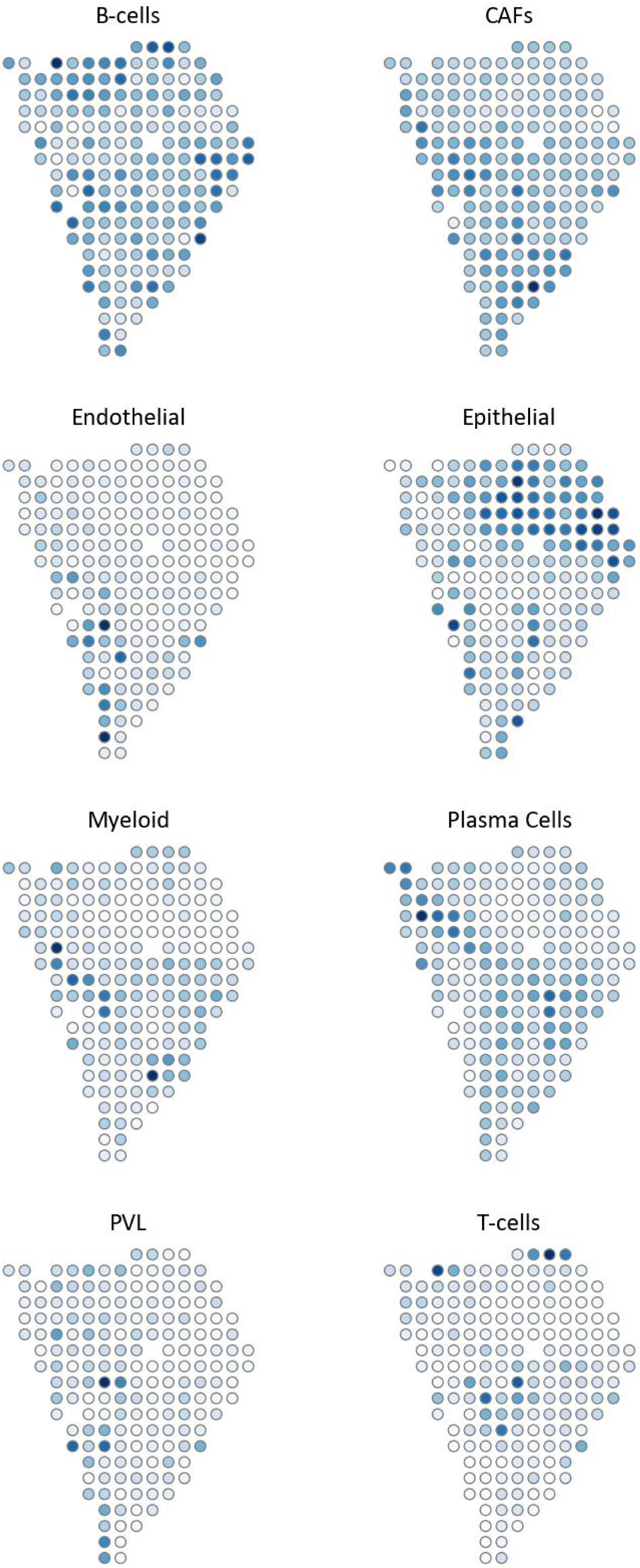

B-cells
CAFs
Endothelial
Epithelial
Myeloid
Plasma Cells
PVL
T-cells

major_A4

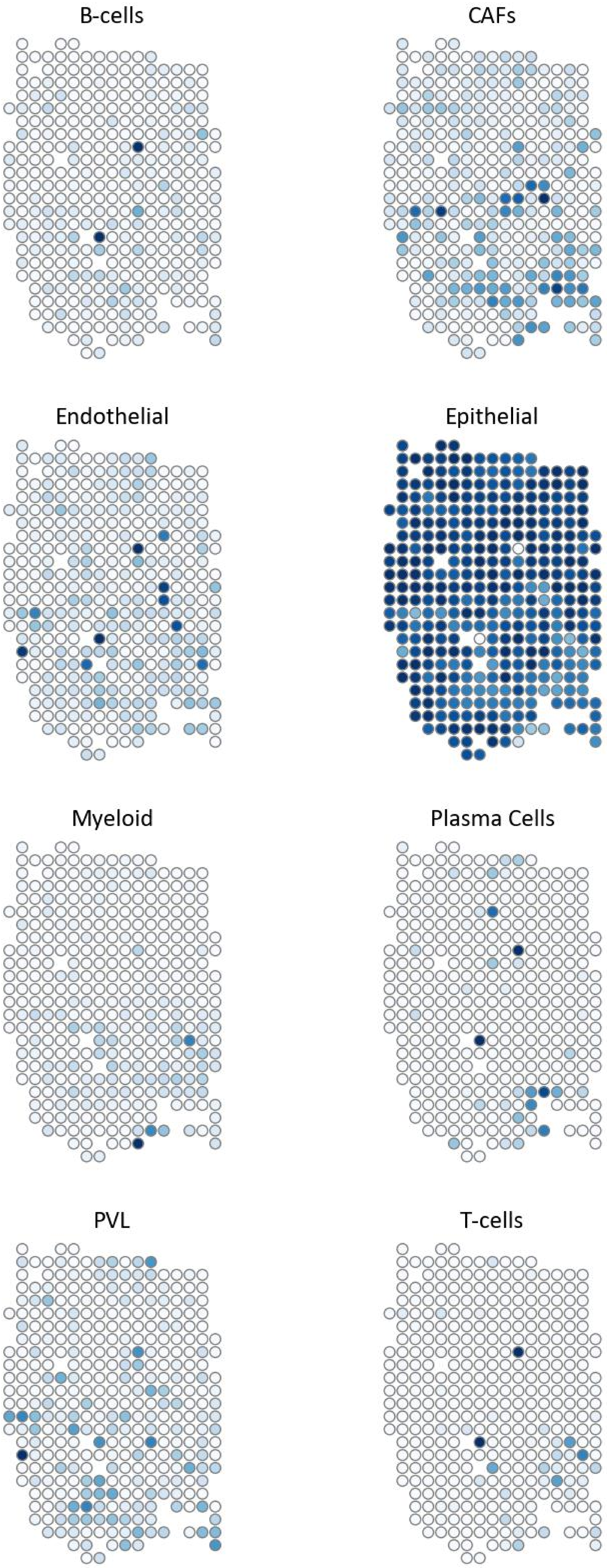

B-cells
CAFs
Endothelial
Epithelial
Myeloid
Plasma Cells
PVL
T-cells

major_B2

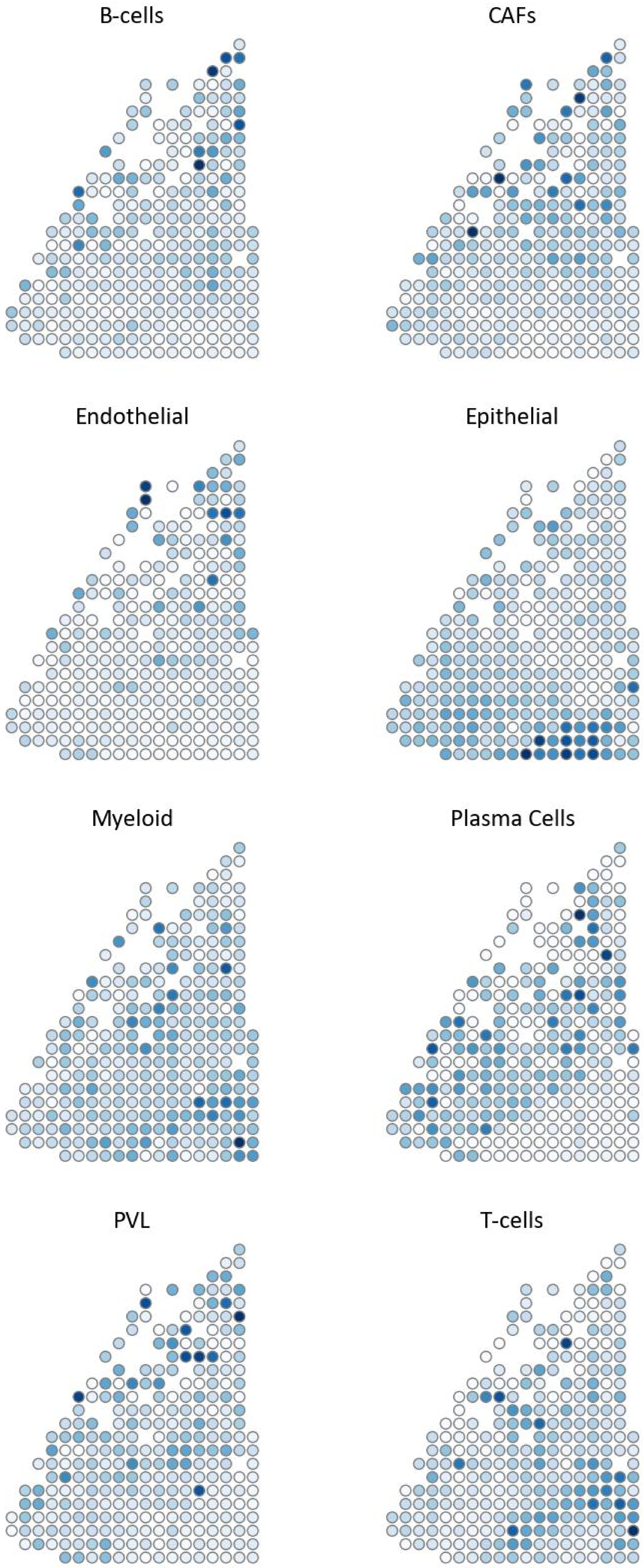

B-cells
CAFs
Endothelial
Epithelial
Myeloid
Plasma Cells
PVL
T-cells

major_E1

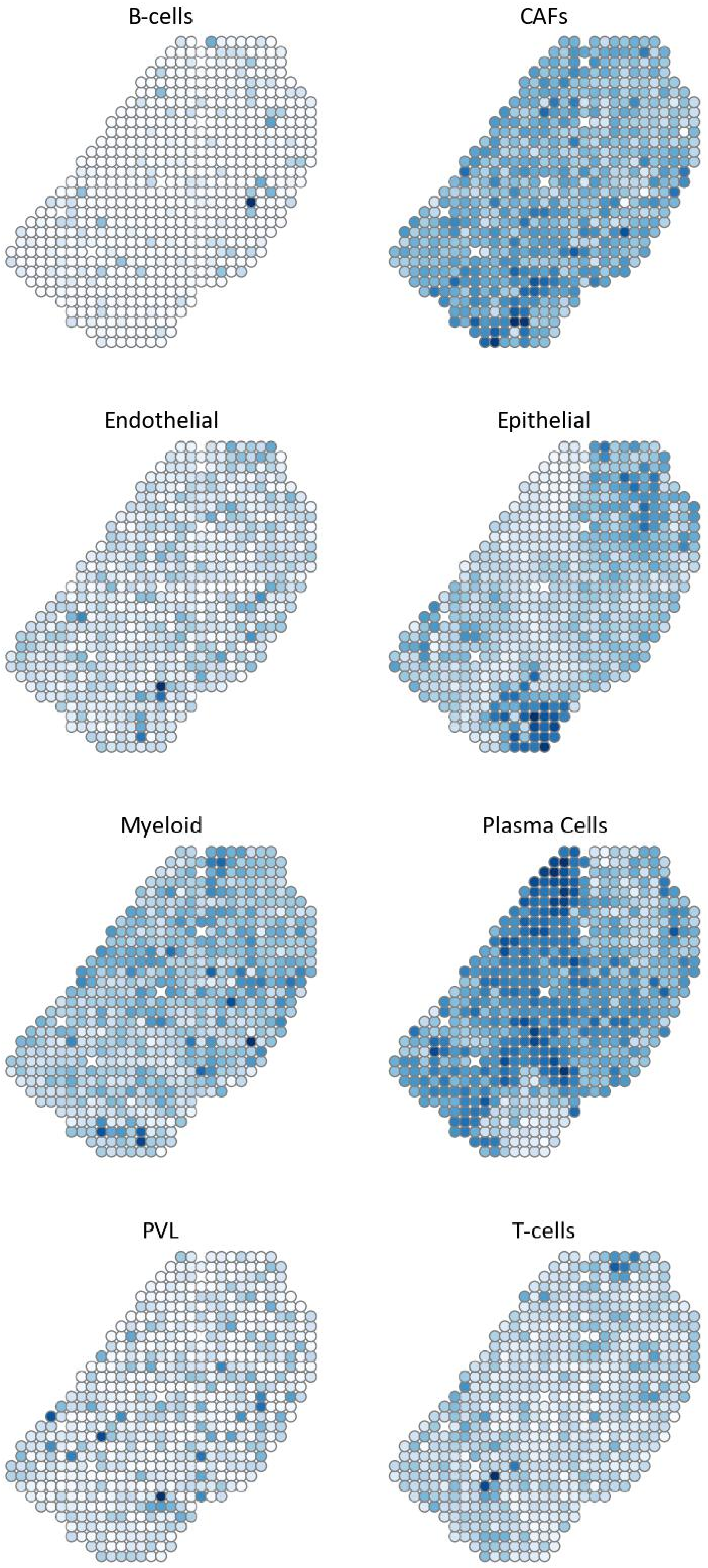
B-cells
CAFs
Endothelial
Epithelial
Myeloid
Plasma Cells
PVL
T-cells

major_B3

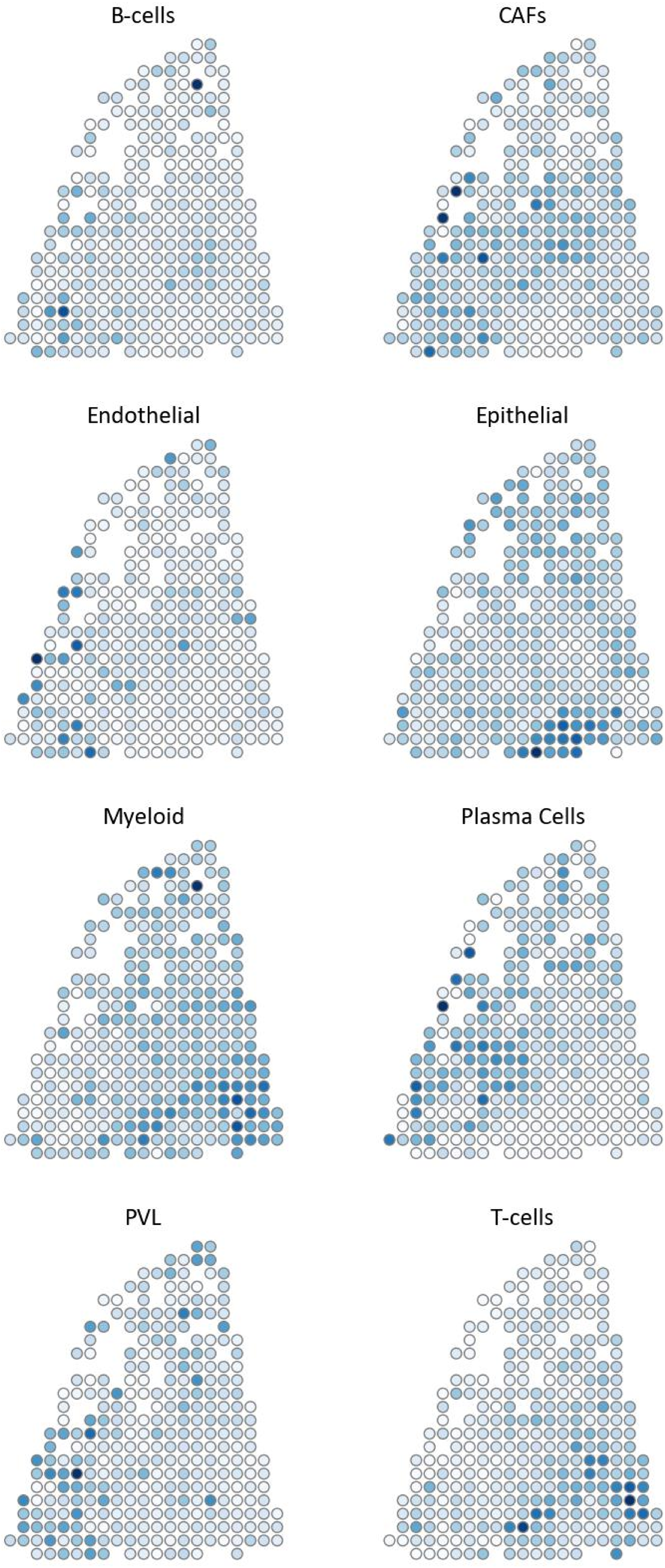

B-cells
CAFs
Endothelial
Epithelial
Myeloid
Plasma Cells
PVL
T-cells

major_B4

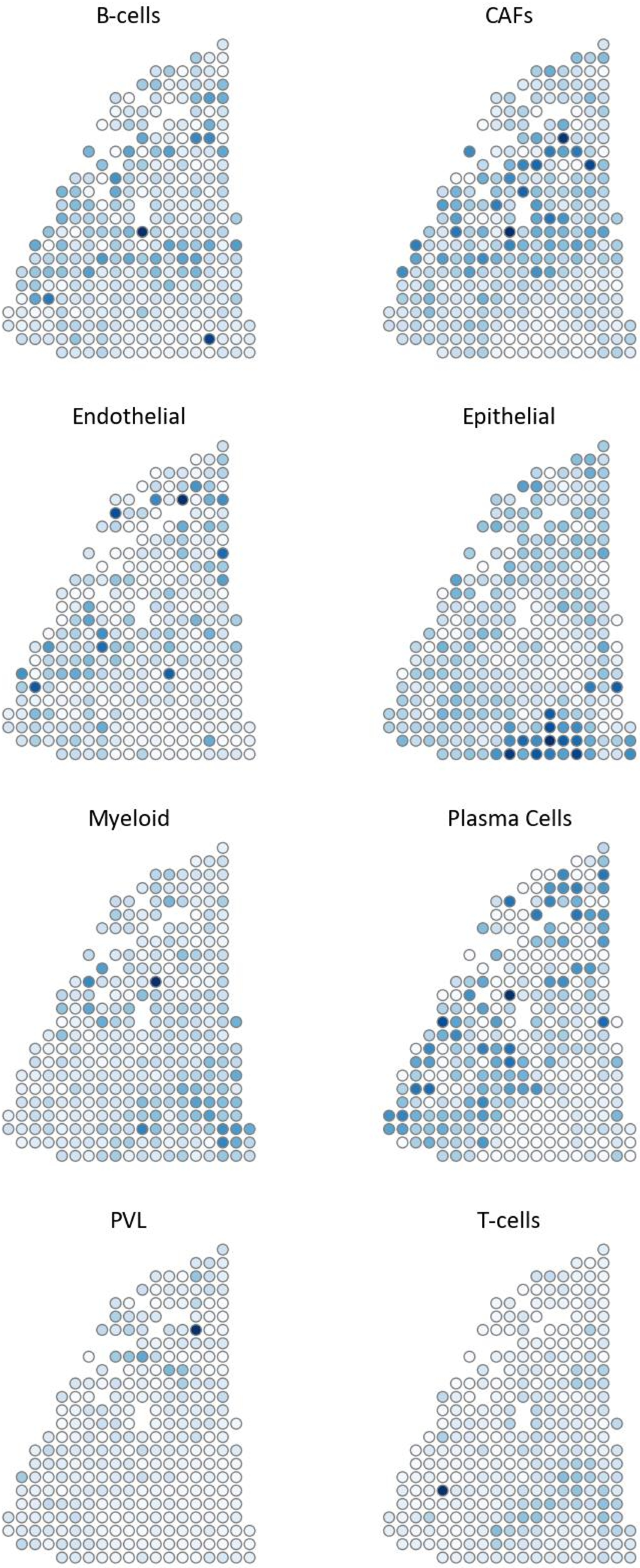
B-cells
CAFs
Endothelial
Epithelial
Myeloid
Plasma Cells
PVL
T-cells

major_D3

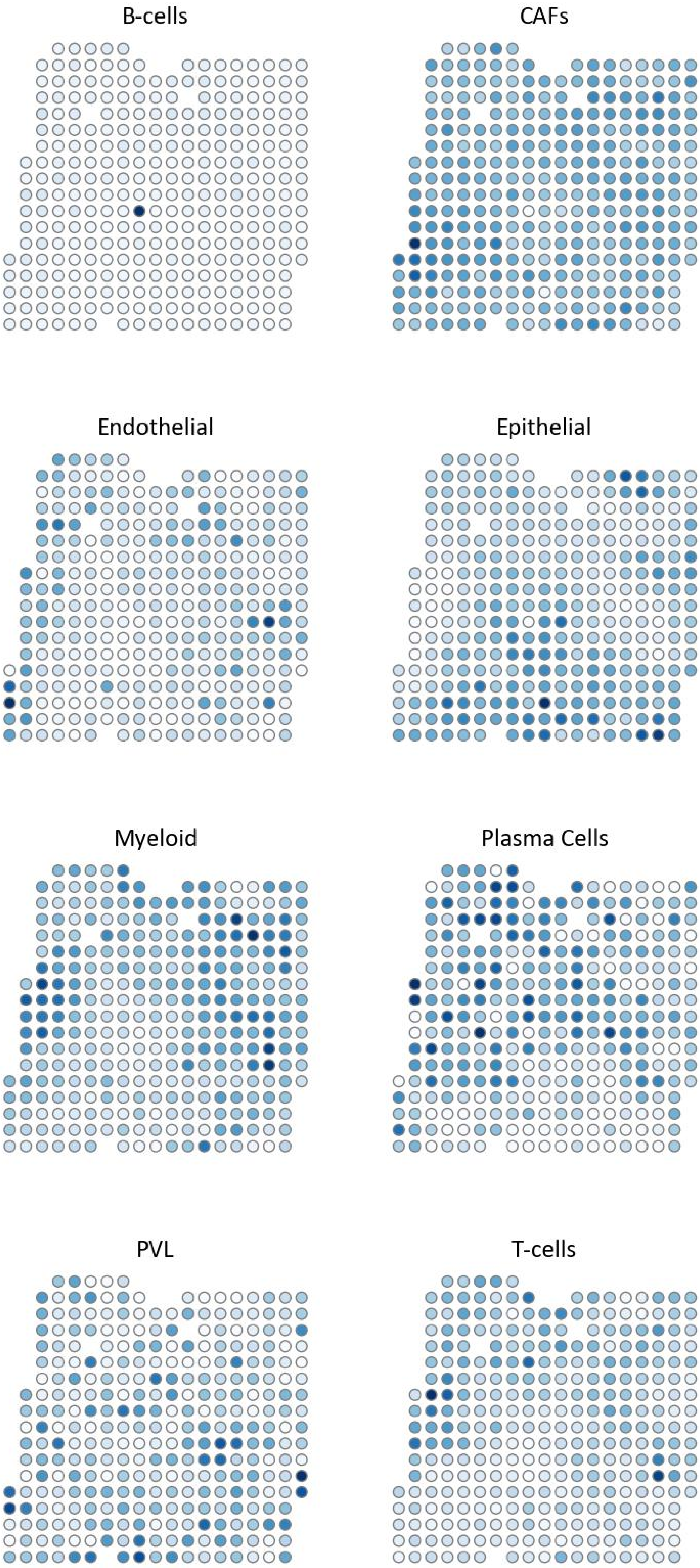
B-cells
CAFs
Endothelial
Epithelial
Myeloid
Plasma Cells
PVL
T-cells

major_C2

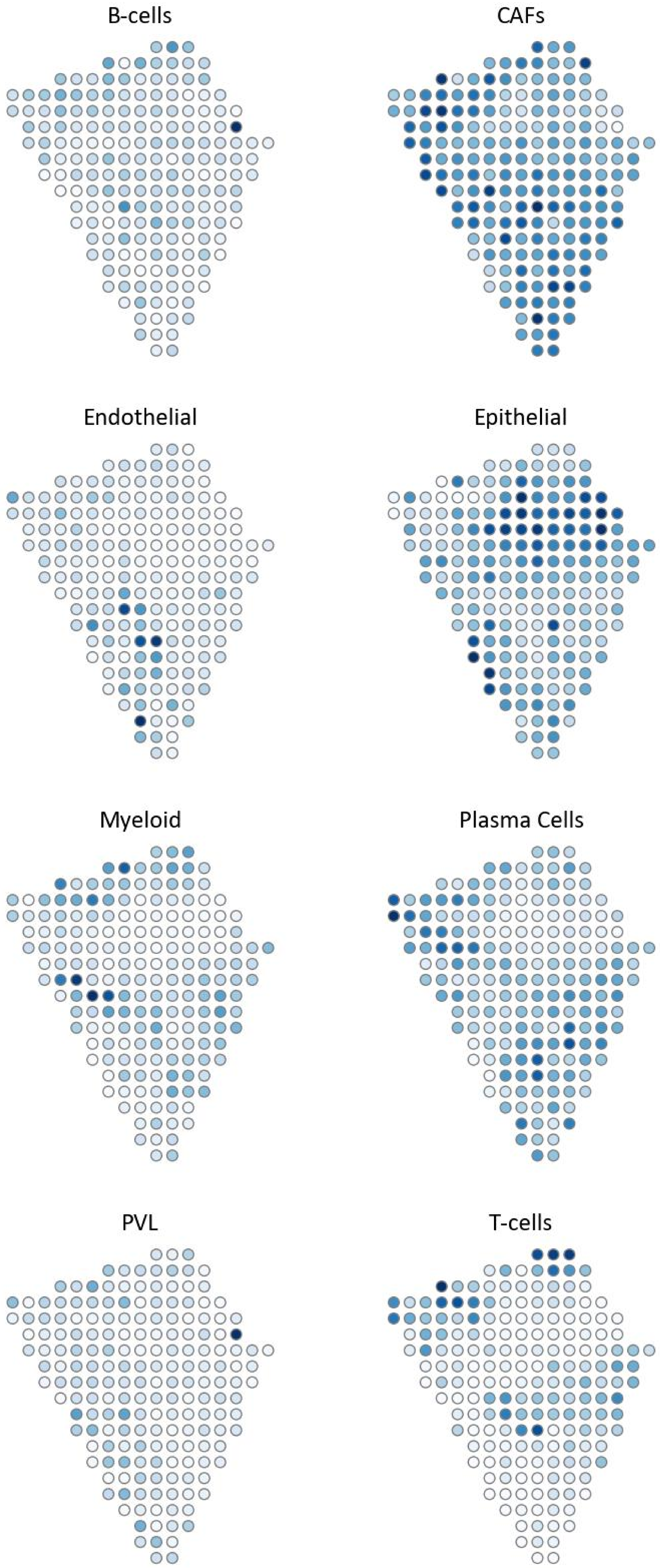

B-cells
CAFs
Endothelial
Epithelial
Myeloid
Plasma Cells
PVL
T-cells

major_C6

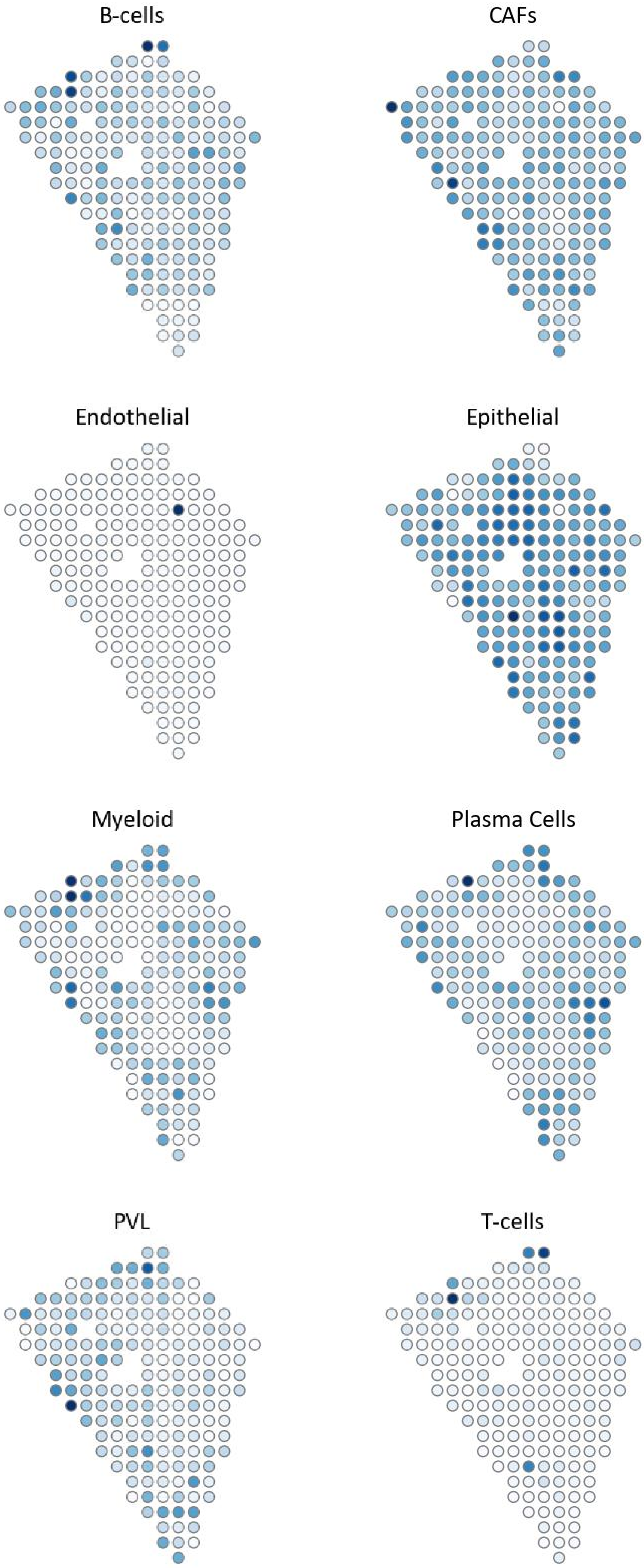
B-cells
CAFs
Endothelial
Epithelial
Myeloid
Plasma Cells
PVL
T-cells

major_A5

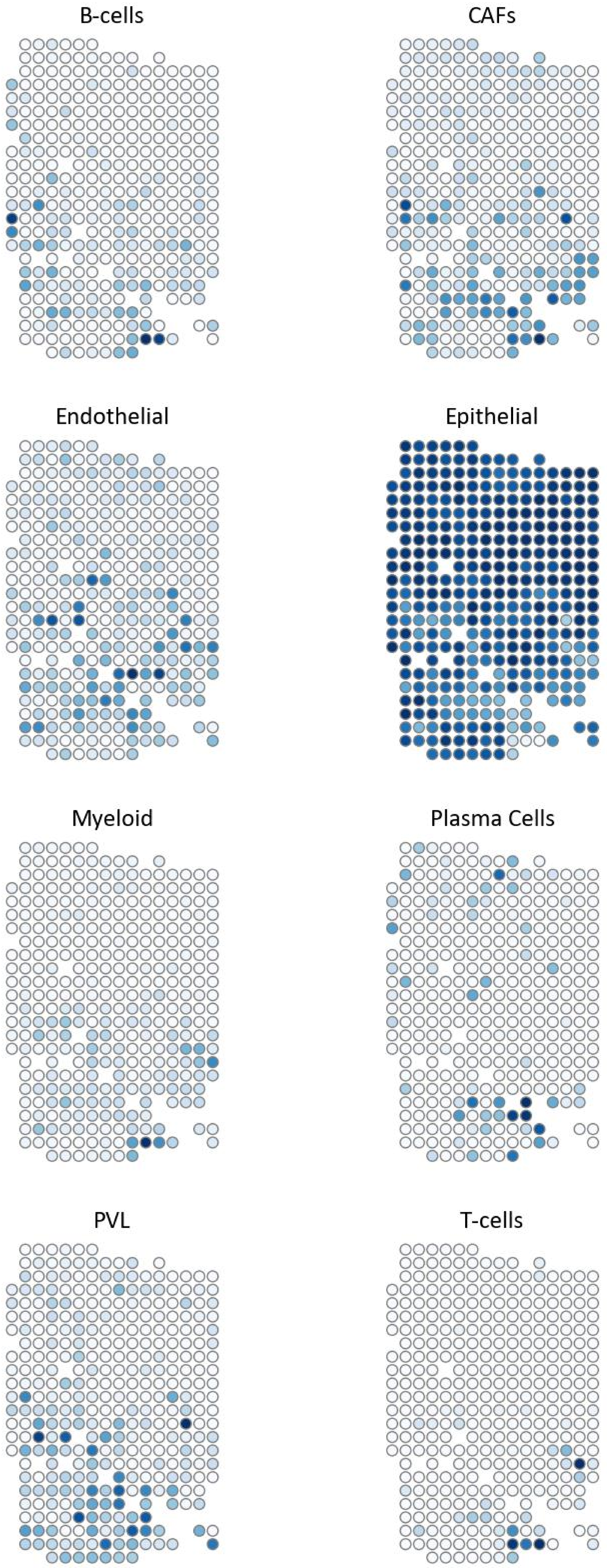

B-cells
CAFs
Endothelial
Epithelial
Myeloid
Plasma Cells
PVL
T-cells

major_H3

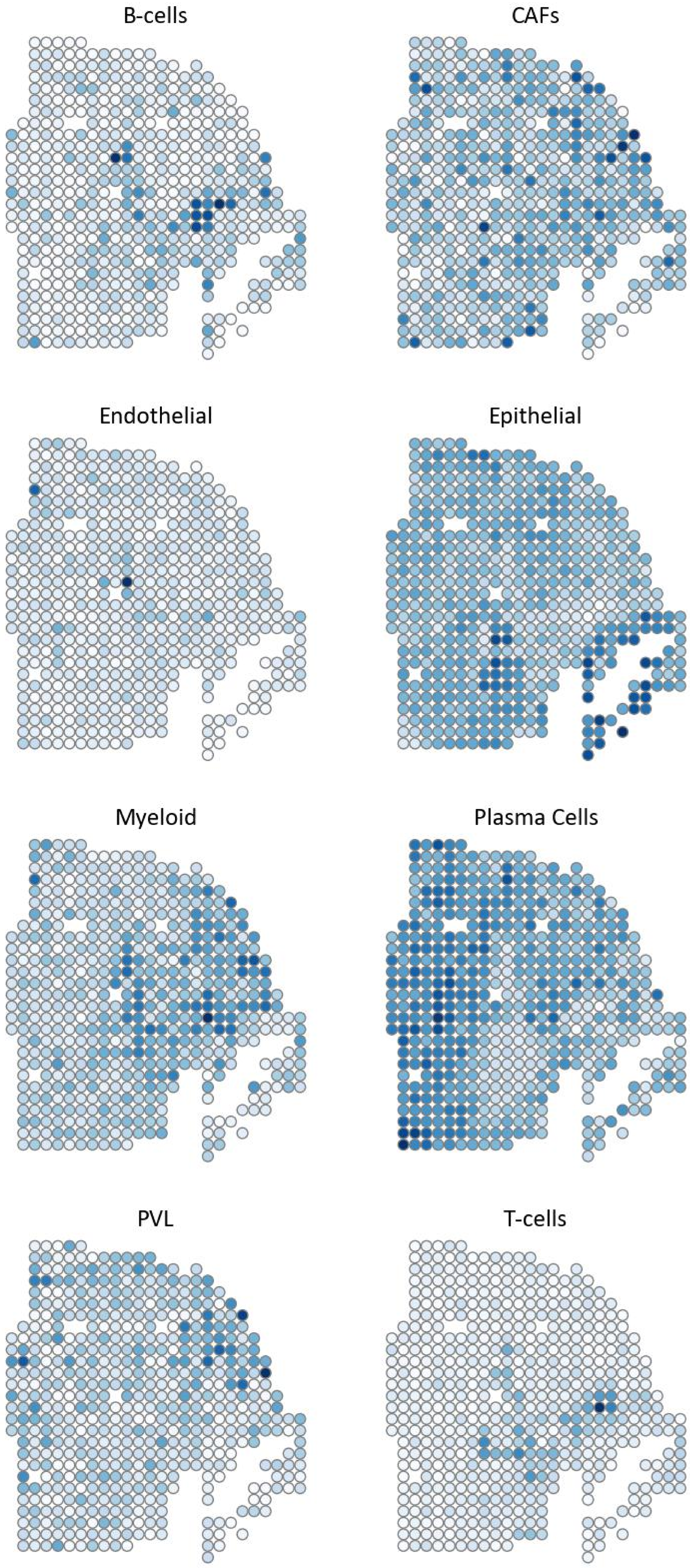

B-cells
CAFs
Endothelial
Epithelial
Myeloid
Plasma Cells
PVL
T-cells

major_C1

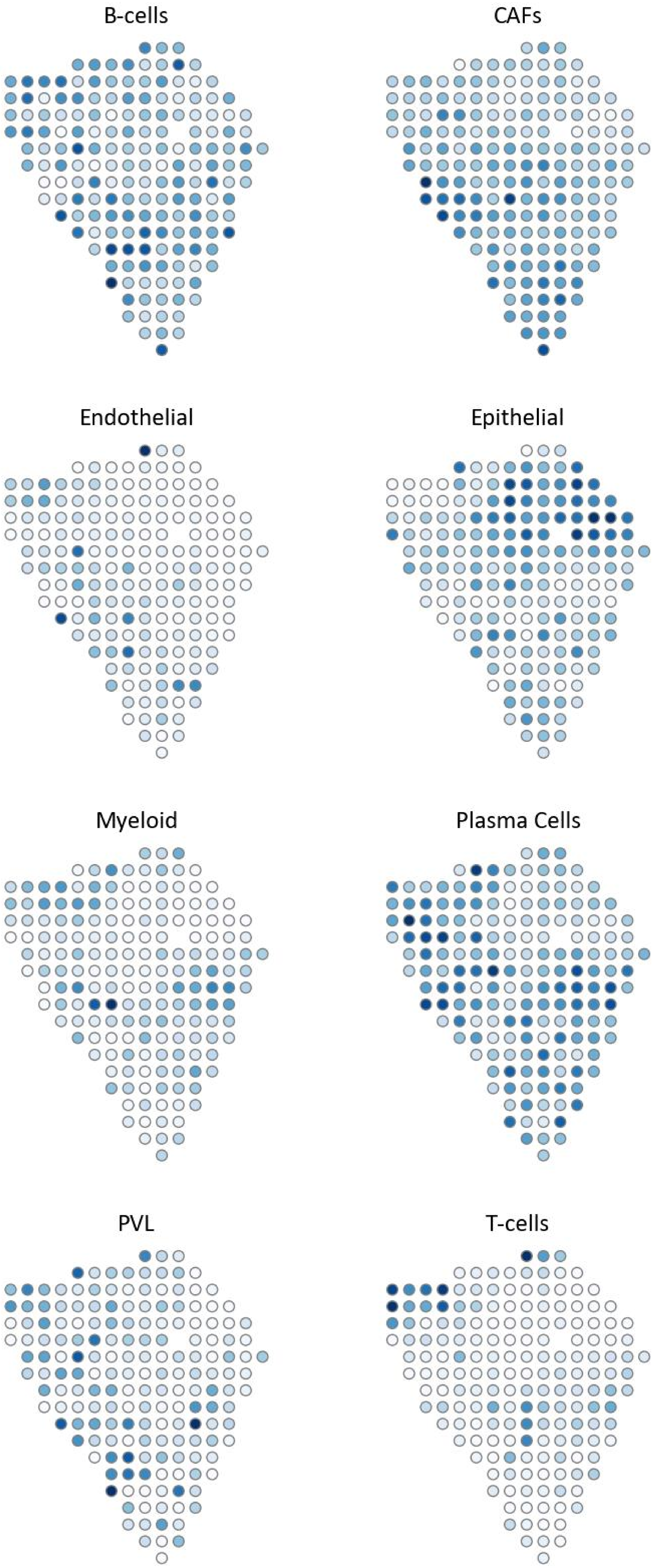
B-cells
CAFs
Endothelial
Epithelial
Myeloid
Plasma Cells
PVL
T-cells

major_G2

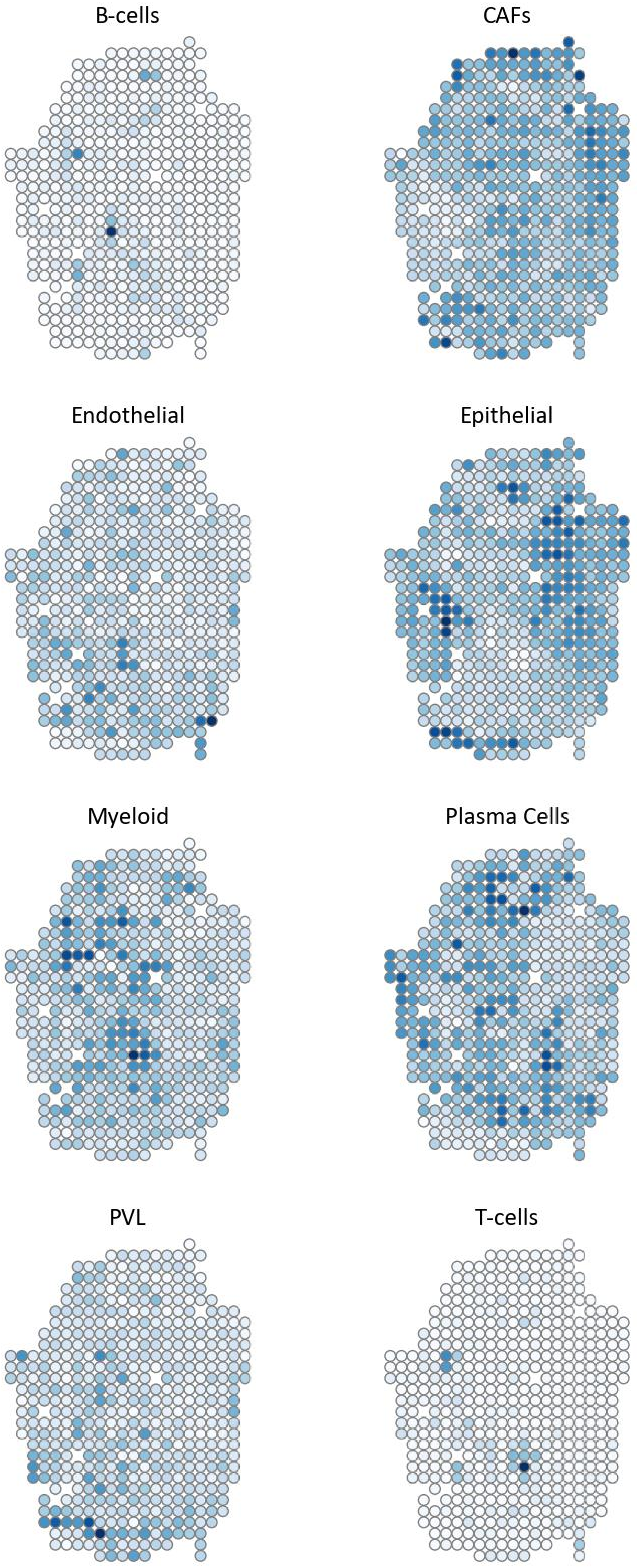
B-cells
CAFs
Endothelial
Epithelial
Myeloid
Plasma Cells
PVL
T-cells

minor_E2

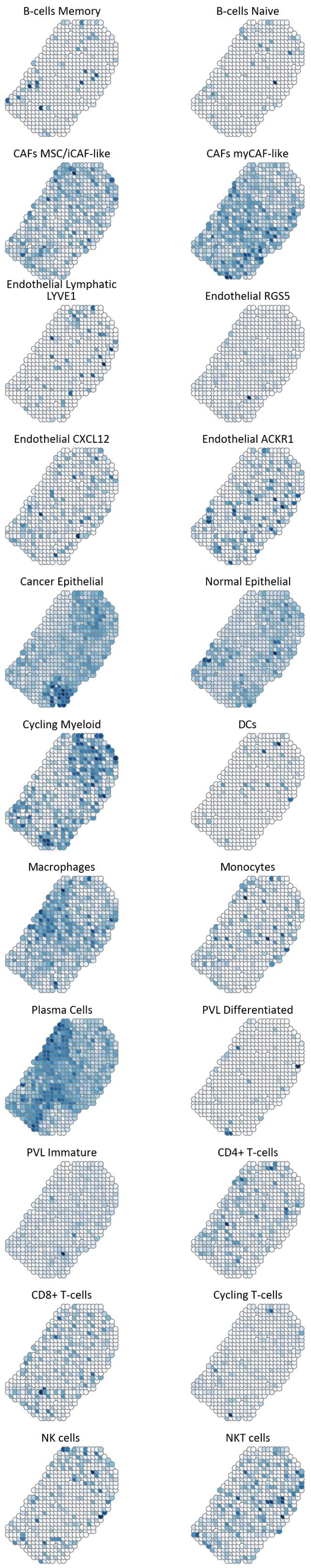

B-cells Memory
B-cells Naive
CAFs MSC/iCAF-like
CAFs myCAF-like
Endothelial Lymphatic LYVE1
Endothelial RGS5
Endothelial CXCL12
Endothelial ACKR1
Cancer Epithelial
Normal Epithelial
Cycling Myeloid
DCs
Macrophages
Monocytes
Plasma Cells
PVL Differentiated
PVL Immature
CD4+ T-cells
CD8+ T-cells
Cycling T-cells
NK cells
NKT cells

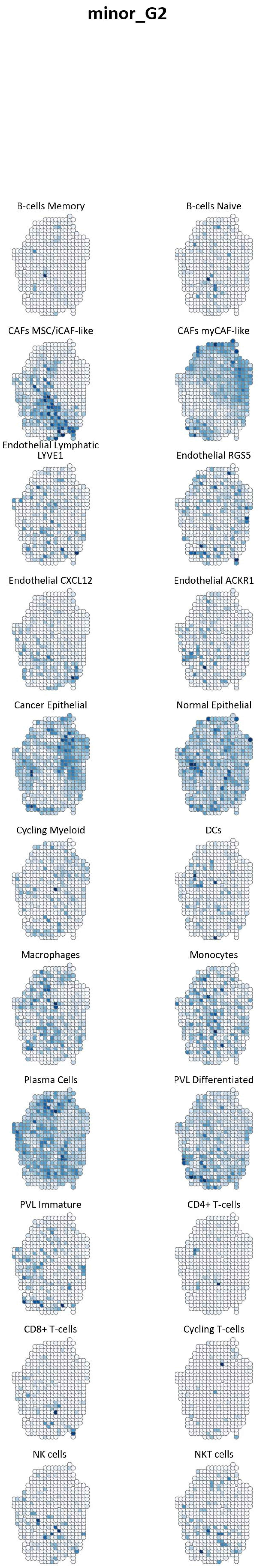
minor_G2
B-cells Memory
B-cells Naive
CAFs MSC/iCAF-like
CAFs myCAF-like
Endothelial Lymphatic LYVE1
Endothelial RGS5
Endothelial CXCL12
Endothelial ACKR1
Cancer Epithelial
Normal Epithelial
Cycling Myeloid
DCs
Macrophages
Monocytes
Plasma Cells
PVL Differentiated
PVL Immature
CD4+ T-cells
CD8+ T-cells
Cycling T-cells
NK cells
NKT cells

minor_C4

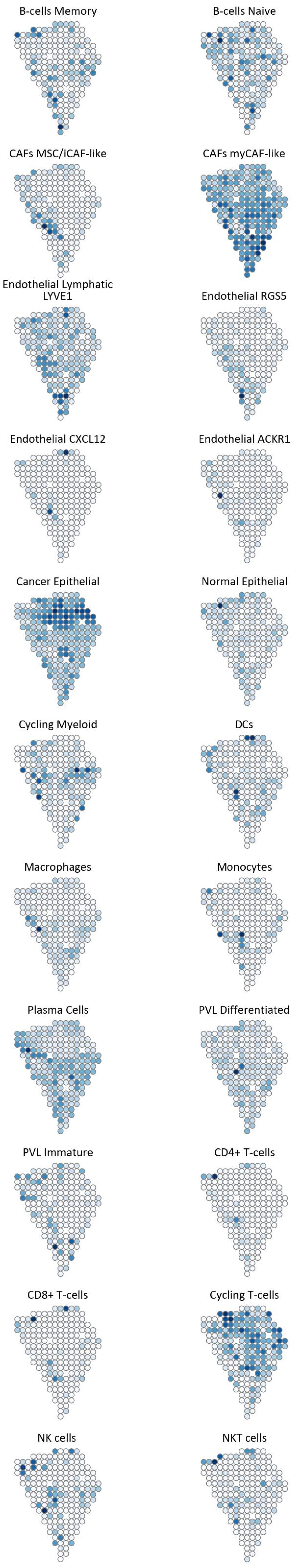
B-cells Memory
B-cells Naive
CAFs MSC/iCAF-like
CAFs myCAF-like
Endothelial Lymphatic LYVE1
Endothelial RGS5
Endothelial CXCL12
Endothelial ACKR1
Cancer Epithelial
Normal Epithelial
Cycling Myeloid
DCs
Macrophages
Monocytes
Plasma Cells
PVL Differentiated
PVL Immature
CD4+ T-cells
CD8+ T-cells
Cycling T-cells
NK cells
NKT cells

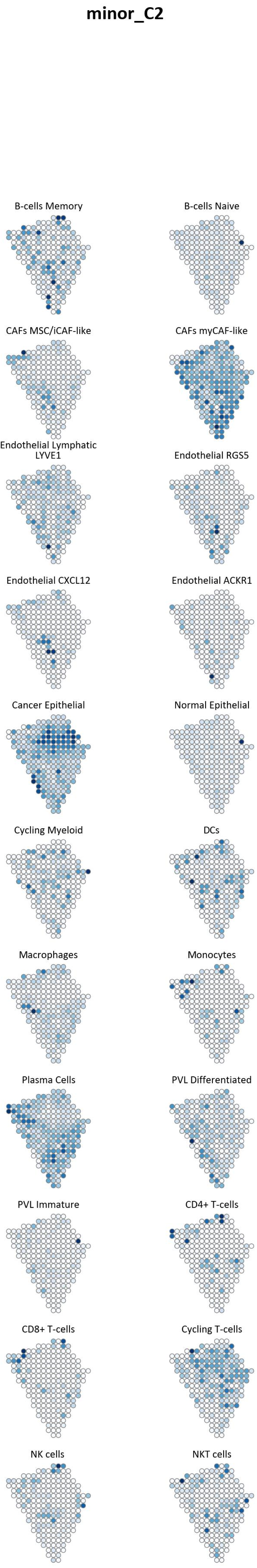
minor_C2
B-cells Memory
B-cells Naive
CAFs MSC/iCAF-like
CAFs myCAF-like
Endothelial Lymphatic LYVE1
Endothelial RGS5
Endothelial CXCL12
Endothelial ACKR1
Cancer Epithelial
Normal Epithelial
Cycling Myeloid
DCs
Macrophages
Monocytes
Plasma Cells
PVL Differentiated
PVL Immature
CD4+ T-cells
CD8+ T-cells
Cycling T-cells
NK cells
NKT cells

minor_C5

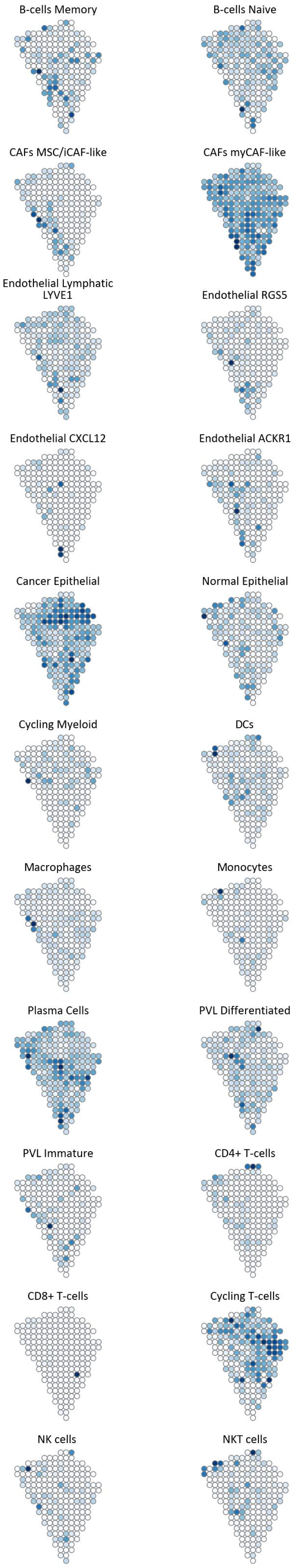
B-cells Memory
B-cells Naive
CAFs MSC/iCAF-like
CAFs myCAF-like
Endothelial Lymphatic LYVE1
Endothelial RGS5
Endothelial CXCL12
Endothelial ACKR1
Cancer Epithelial
Normal Epithelial
Cycling Myeloid
DCs
Macrophages
Monocytes
Plasma Cells
PVL Differentiated
PVL Immature
CD4+ T-cells
CD8+ T-cells
Cycling T-cells
NK cells
NKT cells

minor_E1

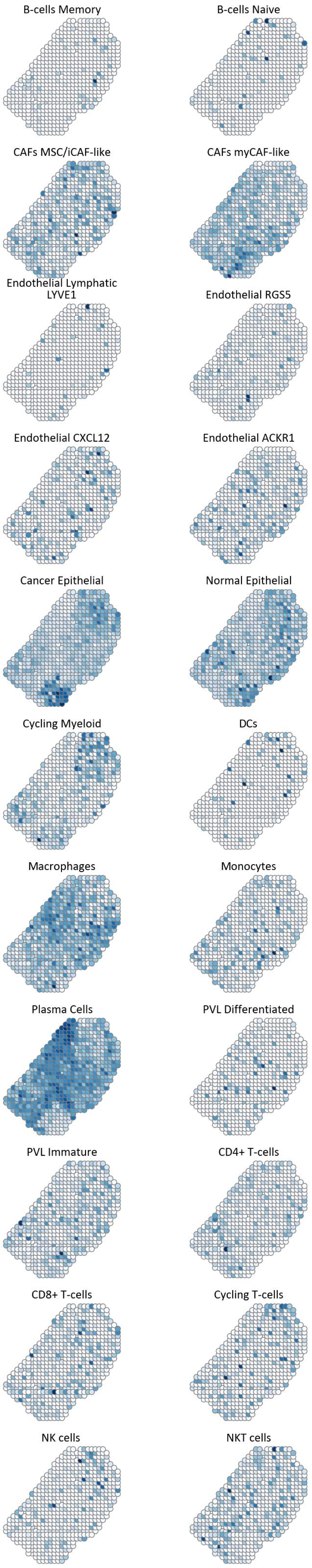
B-cells Memory
B-cells Naive
CAFs MSC/iCAF-like
CAFs myCAF-like
Endothelial Lymphatic LYVE1
Endothelial RGS5
Endothelial CXCL12
Endothelial ACKR1
Cancer Epithelial
Normal Epithelial
Cycling Myeloid
DCs
Macrophages
Monocytes
Plasma Cells
PVL Differentiated
PVL Immature
CD4+ T-cells
CD8+ T-cells
Cycling T-cells
NK cells
NKT cells

minor_B4

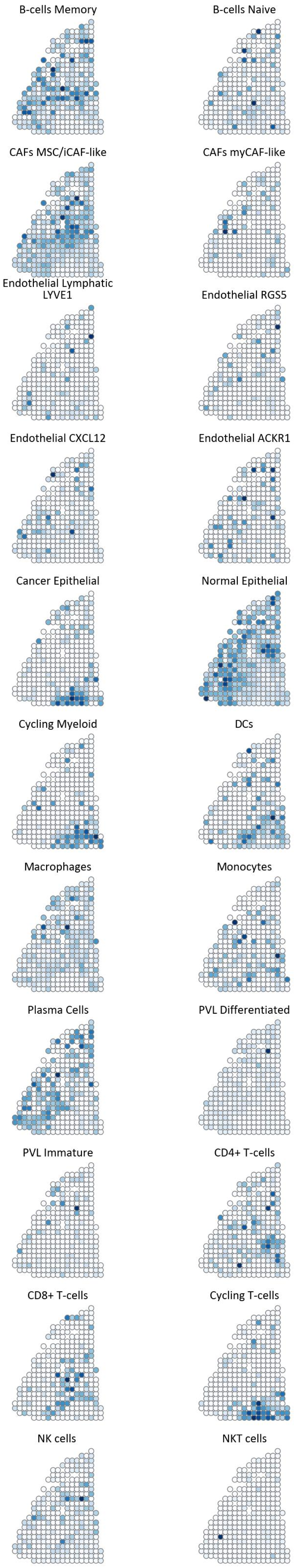
B-cells Memory
B-cells Naive
CAFs MSC/iCAF-like
CAFs myCAF-like
Endothelial Lymphatic LYVE1
Endothelial RGS5
Endothelial CXCL12
Endothelial ACKR1
Cancer Epithelial
Normal Epithelial
Cycling Myeloid
DCs
Macrophages
Monocytes
Plasma Cells
PVL Differentiated
PVL Immature
CD4+ T-cells
CD8+ T-cells
Cycling T-cells
NK cells
NKT cells

minor_A2

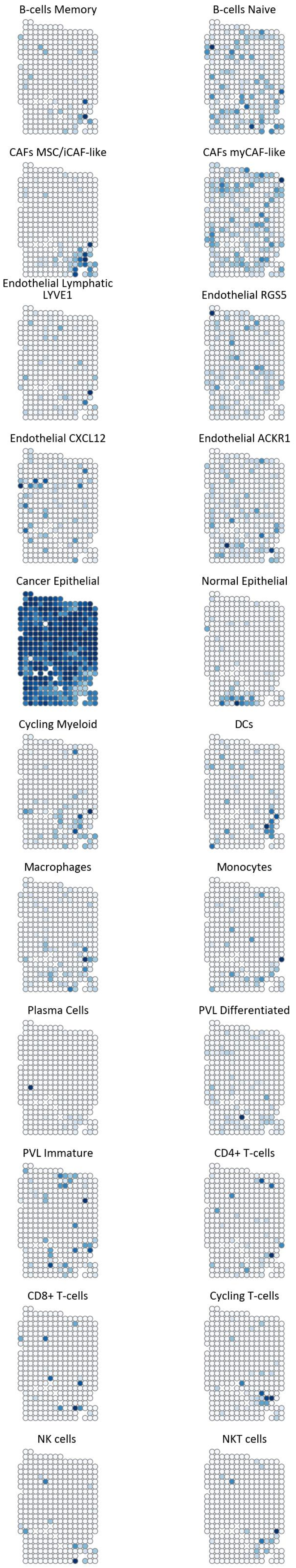

B-cells Memory
B-cells Naive
CAFs MSC/iCAF-like
CAFs myCAF-like
Endothelial Lymphatic LYVE1
Endothelial RGS5
Endothelial CXCL12
Endothelial ACKR1
Cancer Epithelial
Normal Epithelial
Cycling Myeloid
DCs
Macrophages
Monocytes
Plasma Cells
PVL Differentiated
PVL Immature
CD4+ T-cells
CD8+ T-cells
Cycling T-cells
NK cells
NKT cells

minor_A4

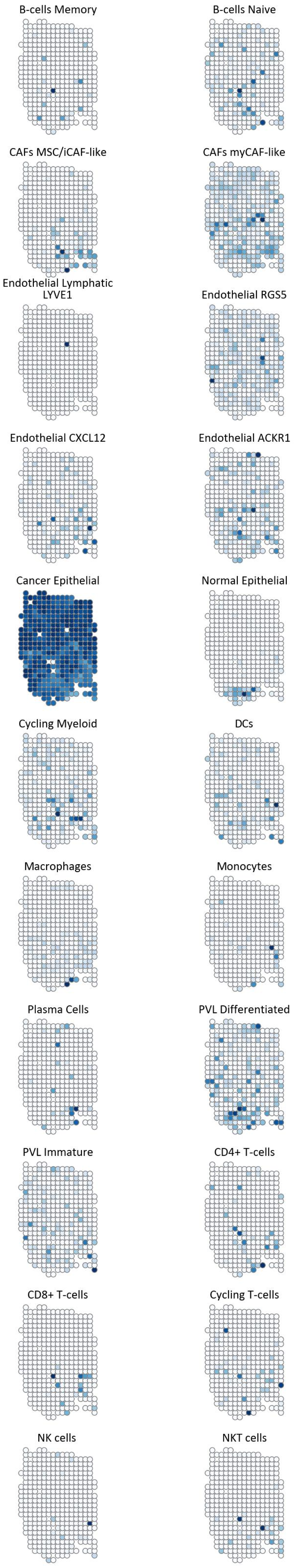

B-cells Memory
B-cells Naive
CAFs MSC/iCAF-like
CAFs myCAF-like
Endothelial Lymphatic LYVE1
Endothelial RGS5
Endothelial CXCL12
Endothelial ACKR1
Cancer Epithelial
Normal Epithelial
Cycling Myeloid
DCs
Macrophages
Monocytes
Plasma Cells
PVL Differentiated
PVL Immature
CD4+ T-cells
CD8+ T-cells
Cycling T-cells
NK cells
NKT cells

minor_A1

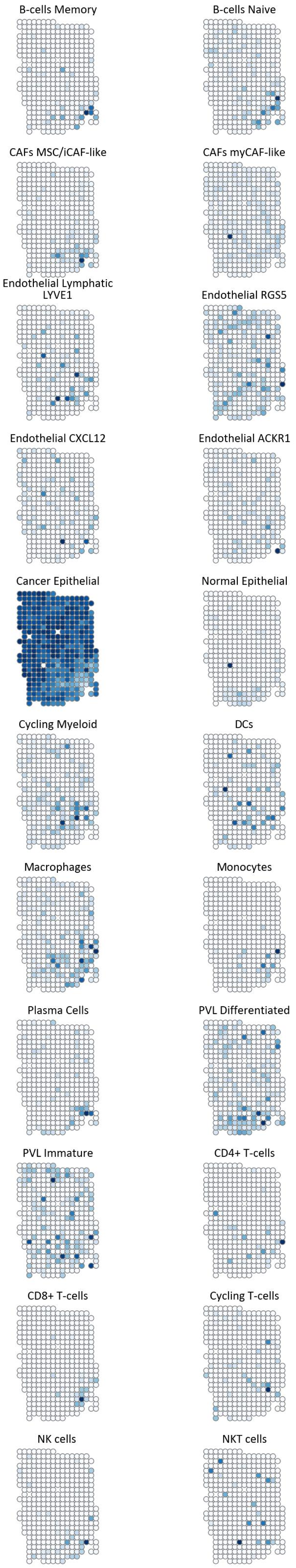
B-cells Memory
B-cells Naive
CAFs MSC/iCAF-like
CAFs myCAF-like
Endothelial Lymphatic LYVE1
Endothelial RGS5
Endothelial CXCL12
Endothelial ACKR1
Cancer Epithelial
Normal Epithelial
Cycling Myeloid
DCs
Macrophages
Monocytes
Plasma Cells
PVL Differentiated
PVL Immature
CD4+ T-cells
CD8+ T-cells
Cycling T-cells
NK cells
NKT cells

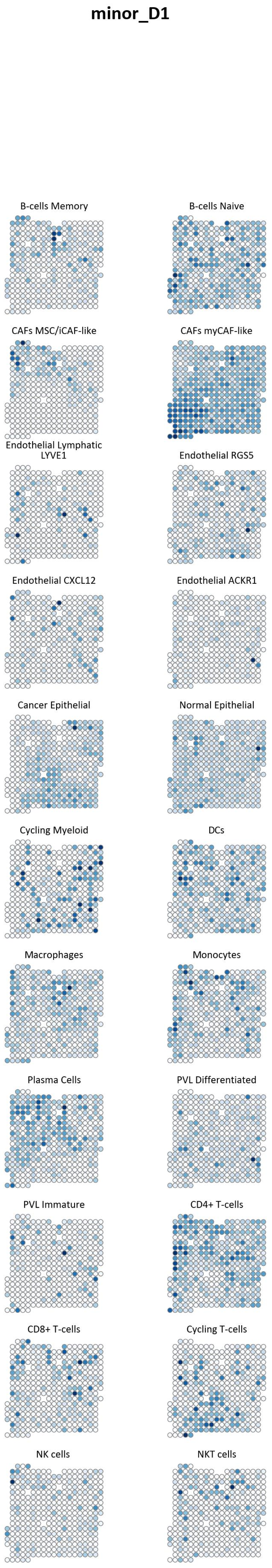
minor_D1
B-cells Memory
B-cells Naive
CAFs MSC/iCAF-like
CAFs myCAF-like
Endothelial Lymphatic LYVE1
Endothelial RGS5
Endothelial CXCL12
Endothelial ACKR1
Cancer Epithelial
Normal Epithelial
Cycling Myeloid
DCs
Macrophages
Monocytes
Plasma Cells
PVL Differentiated
PVL Immature
CD4+ T-cells
CD8+ T-cells
Cycling T-cells
NK cells
NKT cells

minor_C1

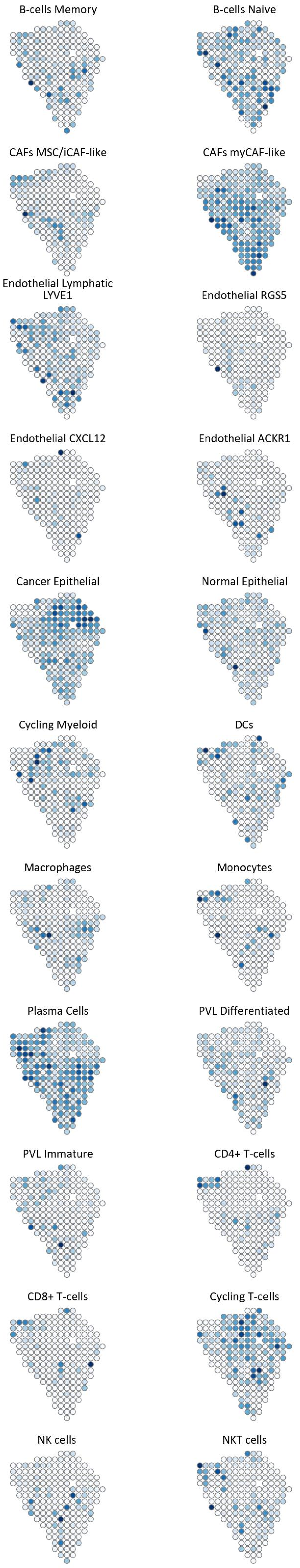
B-cells Memory
B-cells Naive
CAFs MSC/iCAF-like
CAFs myCAF-like
Endothelial Lymphatic LYVE1
Endothelial RGS5
Endothelial CXCL12
Endothelial ACKR1
Cancer Epithelial
Normal Epithelial
Cycling Myeloid
DCs
Macrophages
Monocytes
Plasma Cells
PVL Differentiated
PVL Immature
CD4+ T-cells
CD8+ T-cells
Cycling T-cells
NK cells
NKT cells

minor_D6

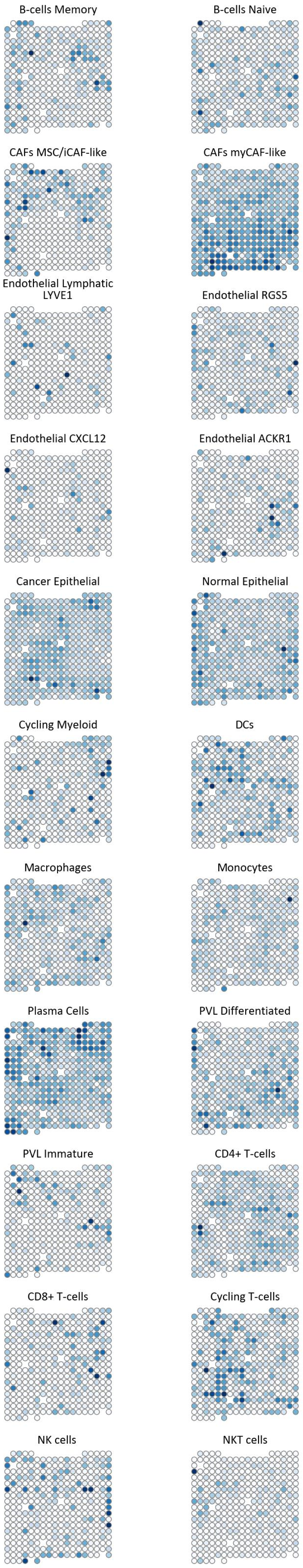
B-cells Memory
B-cells Naive
CAFs MSC/iCAF-like
CAFs myCAF-like
Endothelial Lymphatic LYVE1
Endothelial RGS5
Endothelial CXCL12
Endothelial ACKR1
Cancer Epithelial
Normal Epithelial
Cycling Myeloid
DCs
Macrophages
Monocytes
Plasma Cells
PVL Differentiated
PVL Immature
CD4+ T-cells
CD8+ T-cells
Cycling T-cells
NK cells
NKT cells

minor_A5

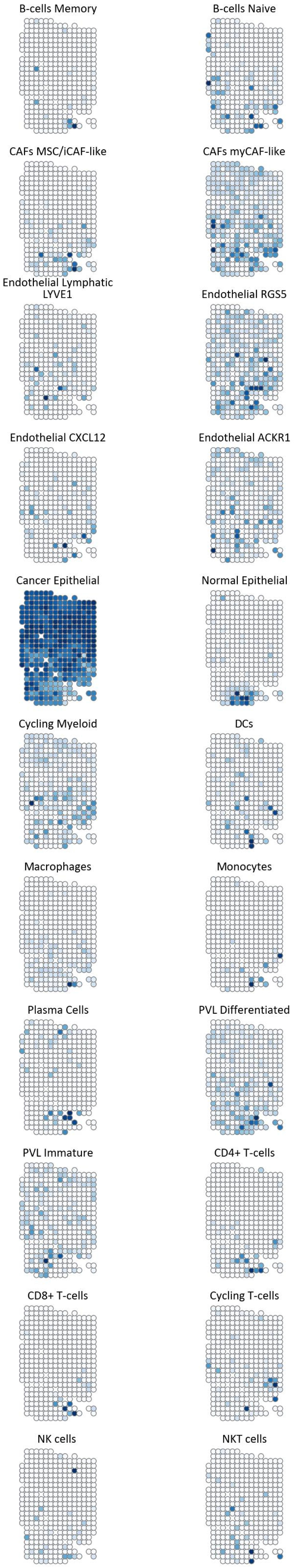
B-cells Memory
B-cells Naive
CAFs MSC/iCAF-like
CAFs myCAF-like
Endothelial Lymphatic LYVE1
Endothelial RGS5
Endothelial CXCL12
Endothelial ACKR1
Cancer Epithelial
Normal Epithelial
Cycling Myeloid
DCs
Macrophages
Monocytes
Plasma Cells
PVL Differentiated
PVL Immature
CD4+ T-cells
CD8+ T-cells
Cycling T-cells
NK cells
NKT cells

minor_H2

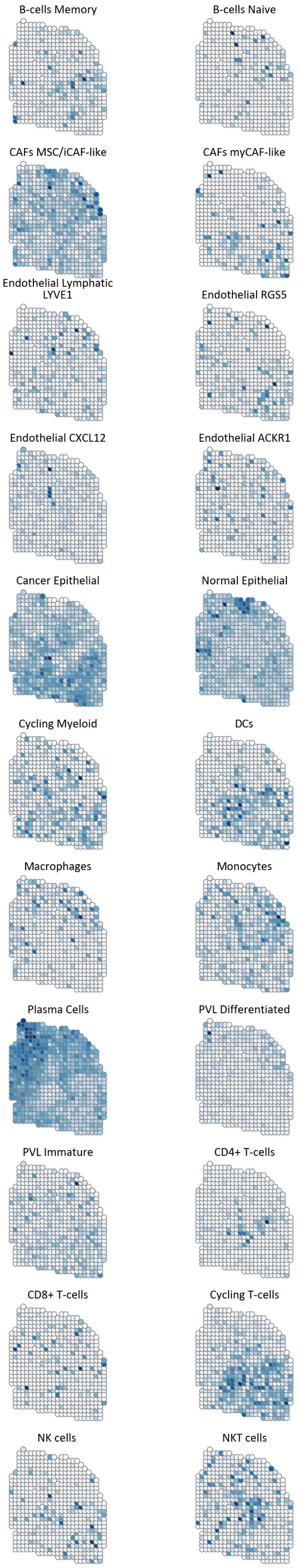

B-cells Memory
B-cells Naive
CAFs MSC/iCAF-like
CAFs myCAF-like
Endothelial Lymphatic LYVE1
Endothelial RGS5
Endothelial CXCL12
Endothelial ACKR1
Cancer Epithelial
Normal Epithelial
Cycling Myeloid
DCs
Macrophages
Monocytes
Plasma Cells
PVL Differentiated
PVL Immature
CD4+ T-cells
CD8+ T-cells
Cycling T-cells
NK cells
NKT cells

minor_D2

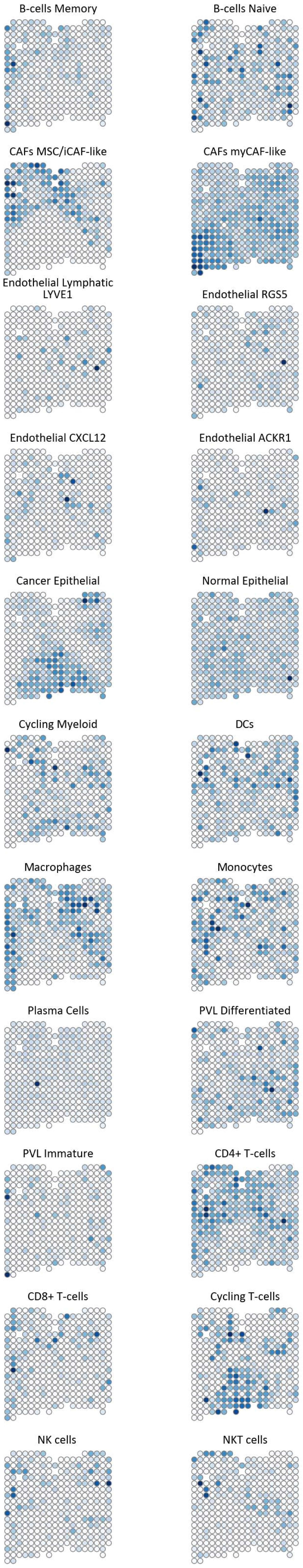
B-cells Memory
B-cells Naive
CAFs MSC/iCAF-like
CAFs myCAF-like
Endothelial Lymphatic LYVE1
Endothelial RGS5
Endothelial CXCL12
Endothelial ACKR1
Cancer Epithelial
Normal Epithelial
Cycling Myeloid
DCs
Macrophages
Monocytes
Plasma Cells
PVL Differentiated
PVL Immature
CD4+ T-cells
CD8+ T-cells
Cycling T-cells
NK cells
NKT cells

minor_H1

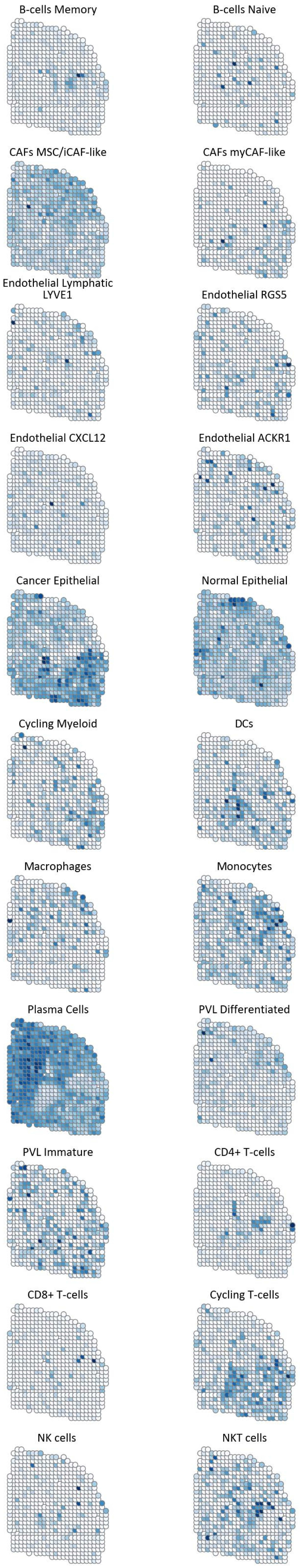
B-cells Memory
B-cells Naive
CAFs MSC/iCAF-like
CAFs myCAF-like
Endothelial Lymphatic LYVE1
Endothelial RGS5
Endothelial CXCL12
Endothelial ACKR1
Cancer Epithelial
Normal Epithelial
Cycling Myeloid
DCs
Macrophages
Monocytes
Plasma Cells
PVL Differentiated
PVL Immature
CD4+ T-cells
CD8+ T-cells
Cycling T-cells
NK cells
NKT cells

minor_F1

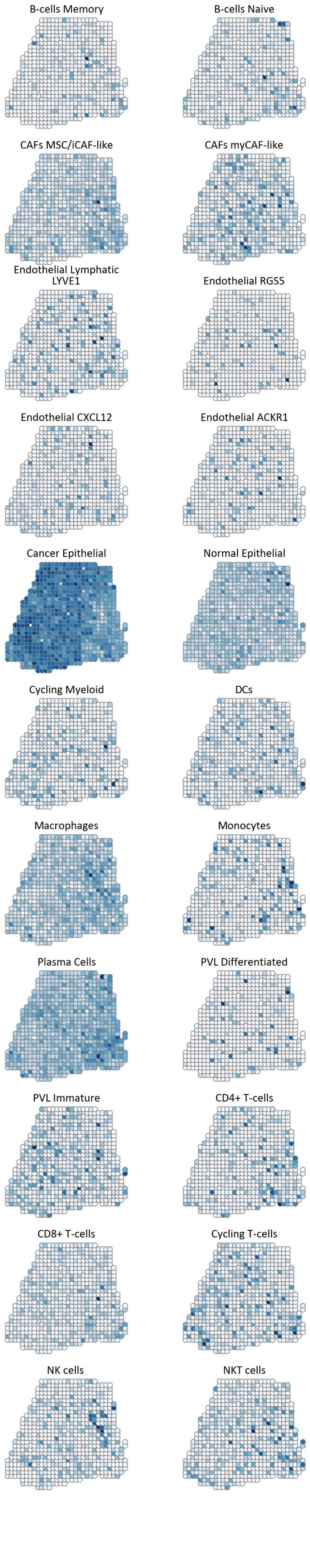
B-cells Memory
B-cells Naive
CAFs MSC/iCAF-like
CAFs myCAF-like
Endothelial Lymphatic LYVE1
Endothelial RGS5
Endothelial CXCL12
Endothelial ACKR1
Cancer Epithelial
Normal Epithelial
Cycling Myeloid
DCs
Macrophages
Monocytes
Plasma Cells
PVL Differentiated
PVL Immature
CD4+ T-cells
CD8+ T-cells
Cycling T-cells
NK cells
NKT cells

minor_B1

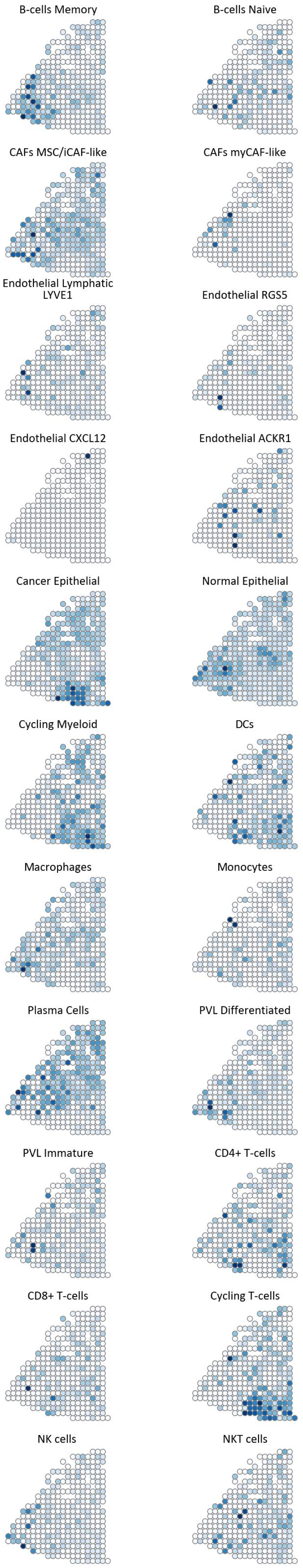
B-cells Memory
B-cells Naive
CAFs MSC/iCAF-like
CAFs myCAF-like
Endothelial Lymphatic LYVE1
Endothelial RGS5
Endothelial CXCL12
Endothelial ACKR1
Cancer Epithelial
Normal Epithelial
Cycling Myeloid
DCs
Macrophages
Monocytes
Plasma Cells
PVL Differentiated
PVL Immature
CD4+ T-cells
CD8+ T-cells
Cycling T-cells
NK cells
NKT cells

minor_G1

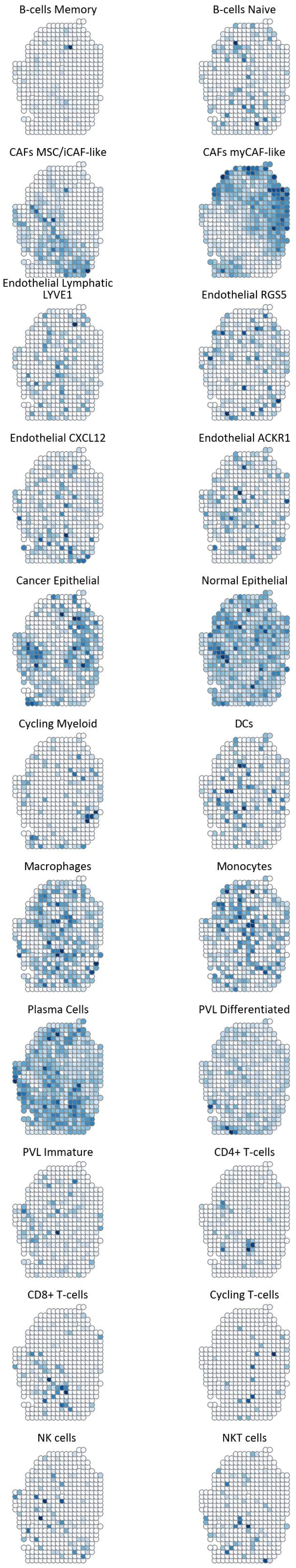
B-cells Memory
B-cells Naive
CAFs MSC/iCAF-like
CAFs myCAF-like
Endothelial Lymphatic LYVE1
Endothelial RGS5
Endothelial CXCL12
Endothelial ACKR1
Cancer Epithelial
Normal Epithelial
Cycling Myeloid
DCs
Macrophages
Monocytes
Plasma Cells
PVL Differentiated
PVL Immature
CD4+ T-cells
CD8+ T-cells
Cycling T-cells
NK cells
NKT cells

minor_D3

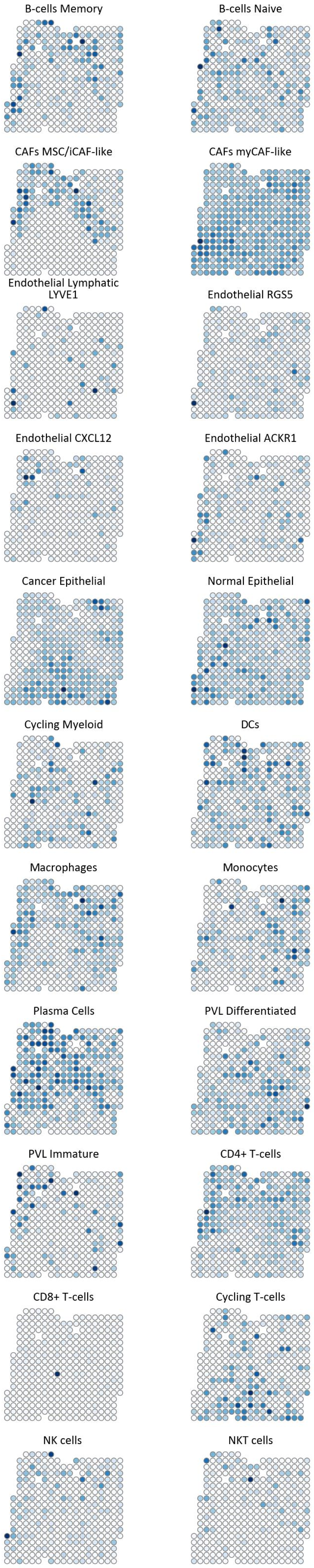
B-cells Memory
B-cells Naive
CAFs MSC/iCAF-like
CAFs myCAF-like
Endothelial Lymphatic LYVE1
Endothelial RGS5
Endothelial CXCL12
Endothelial ACKR1
Cancer Epithelial
Normal Epithelial
Cycling Myeloid
DCs
Macrophages
Monocytes
Plasma Cells
PVL Differentiated
PVL Immature
CD4+ T-cells
CD8+ T-cells
Cycling T-cells
NK cells
NKT cells

minor_C6

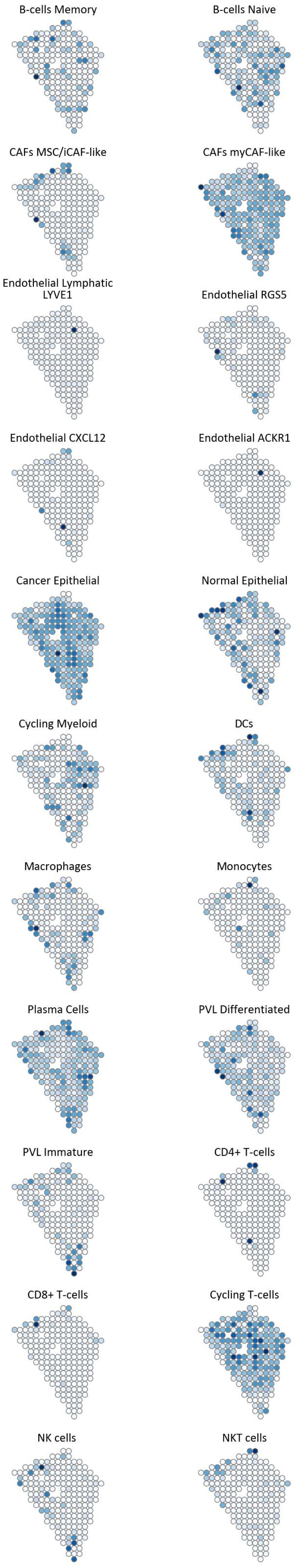
B-cells Memory
B-cells Naive
CAFs MSC/iCAF-like
CAFs myCAF-like
Endothelial Lymphatic LYVE1
Endothelial RGS5
Endothelial CXCL12
Endothelial ACKR1
Cancer Epithelial
Normal Epithelial
Cycling Myeloid
DCs
Macrophages
Monocytes
Plasma Cells
PVL Differentiated
PVL Immature
CD4+ T-cells
CD8+ T-cells
Cycling T-cells
NK cells
NKT cells

minor_B5

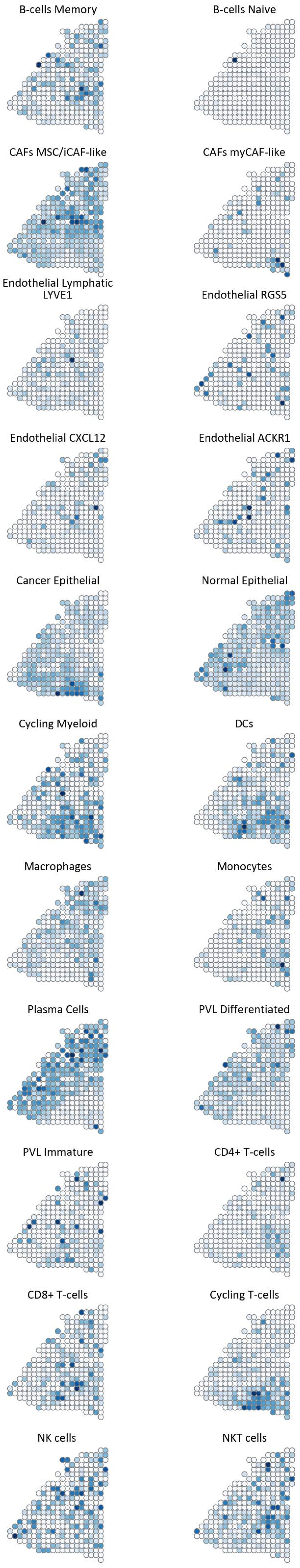
B-cells Memory
B-cells Naive
CAFs MSC/iCAF-like
CAFs myCAF-like
Endothelial Lymphatic LYVE1
Endothelial RGS5
Endothelial CXCL12
Endothelial ACKR1
Cancer Epithelial
Normal Epithelial
Cycling Myeloid
DCs
Macrophages
Monocytes
Plasma Cells
PVL Differentiated
PVL Immature
CD4+ T-cells
CD8+ T-cells
Cycling T-cells
NK cells
NKT cells

minor_E3

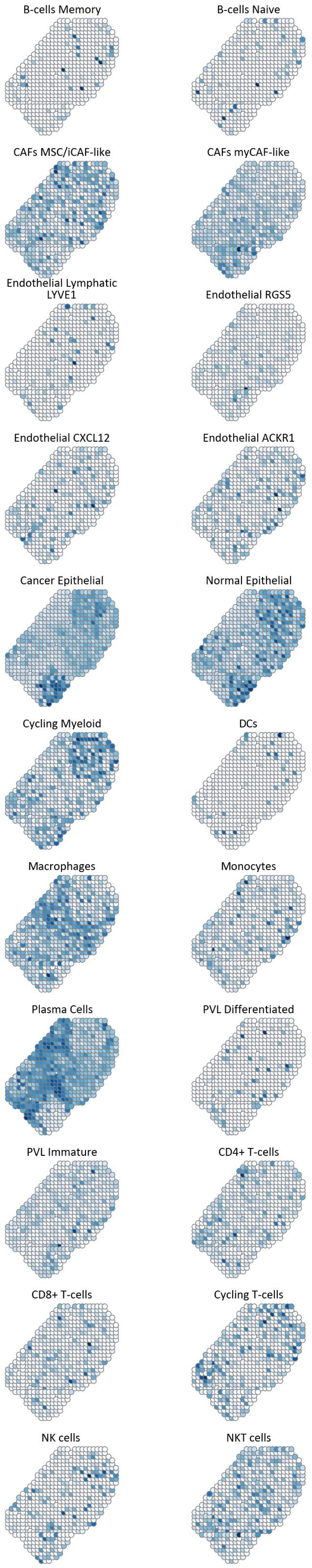
B-cells Memory
B-cells Naive
CAFs MSC/iCAF-like
CAFs myCAF-like
Endothelial Lymphatic LYVE1
Endothelial RGS5
Endothelial CXCL12
Endothelial ACKR1
Cancer Epithelial
Normal Epithelial
Cycling Myeloid
DCs
Macrophages
Monocytes
Plasma Cells
PVL Differentiated
PVL Immature
CD4+ T-cells
CD8+ T-cells
Cycling T-cells
NK cells
NKT cells

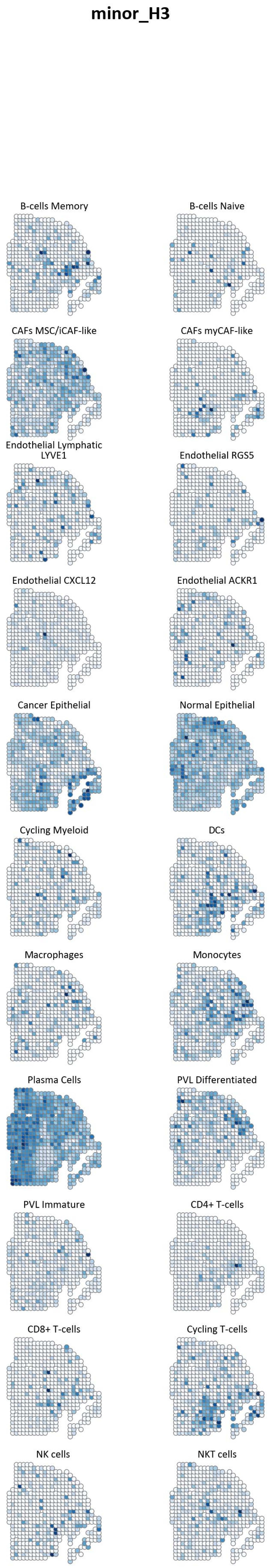
minor_H3
B-cells Memory
B-cells Naive
CAFs MSC/iCAF-like
CAFs myCAF-like
Endothelial Lymphatic LYVE1
Endothelial RGS5
Endothelial CXCL12
Endothelial ACKR1
Cancer Epithelial
Normal Epithelial
Cycling Myeloid
DCs
Macrophages
Monocytes
Plasma Cells
PVL Differentiated
PVL Immature
CD4+ T-cells
CD8+ T-cells
Cycling T-cells
NK cells
NKT cells

minor_D4

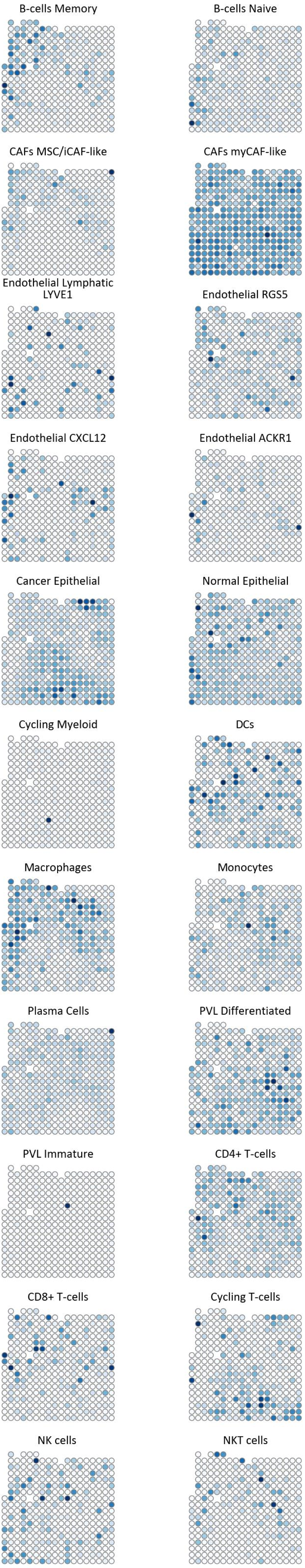
B-cells Memory
B-cells Naive
CAFs MSC/iCAF-like
CAFs myCAF-like
Endothelial Lymphatic LYVE1
Endothelial RGS5
Endothelial CXCL12
Endothelial ACKR1
Cancer Epithelial
Normal Epithelial
Cycling Myeloid
DCs
Macrophages
Monocytes
Plasma Cells
PVL Differentiated
PVL Immature
CD4+ T-cells
CD8+ T-cells
Cycling T-cells
NK cells
NKT cells

minor_B2

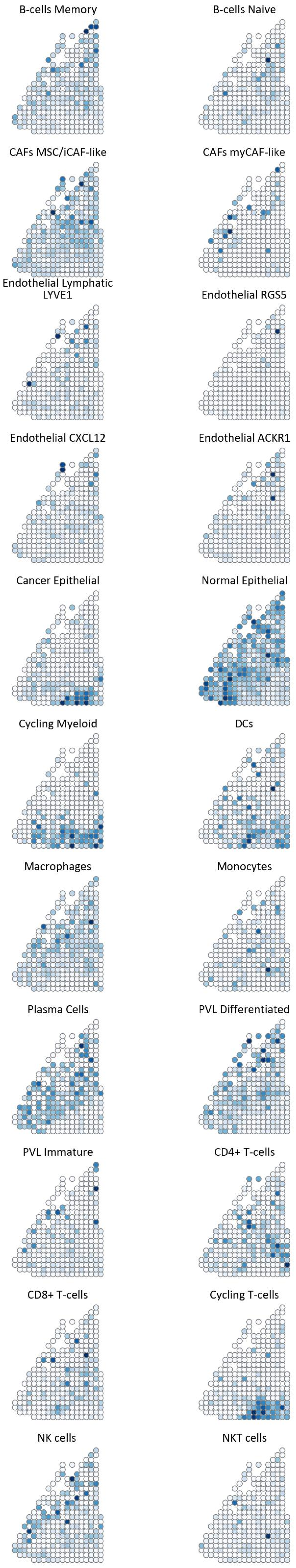
B-cells Memory
B-cells Naive
CAFs MSC/iCAF-like
CAFs myCAF-like
Endothelial Lymphatic LYVE1
Endothelial RGS5
Endothelial CXCL12
Endothelial ACKR1
Cancer Epithelial
Normal Epithelial
Cycling Myeloid
DCs
Macrophages
Monocytes
Plasma Cells
PVL Differentiated
PVL Immature
CD4+ T-cells
CD8+ T-cells
Cycling T-cells
NK cells
NKT cells

minor_A6

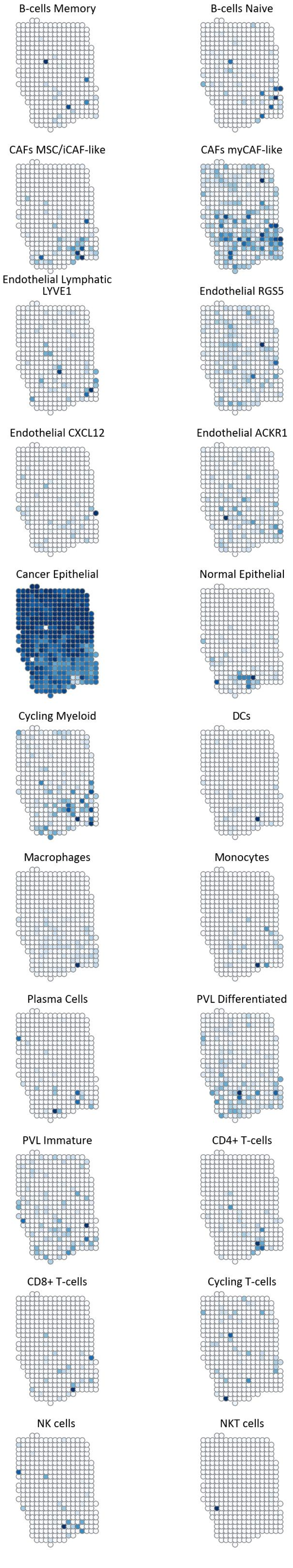
B-cells Memory
B-cells Naive
CAFs MSC/iCAF-like
CAFs myCAF-like
Endothelial Lymphatic LYVE1
Endothelial RGS5
Endothelial CXCL12
Endothelial ACKR1
Cancer Epithelial
Normal Epithelial
Cycling Myeloid
DCs
Macrophages
Monocytes
Plasma Cells
PVL Differentiated
PVL Immature
CD4+ T-cells
CD8+ T-cells
Cycling T-cells
NK cells
NKT cells

minor_F3

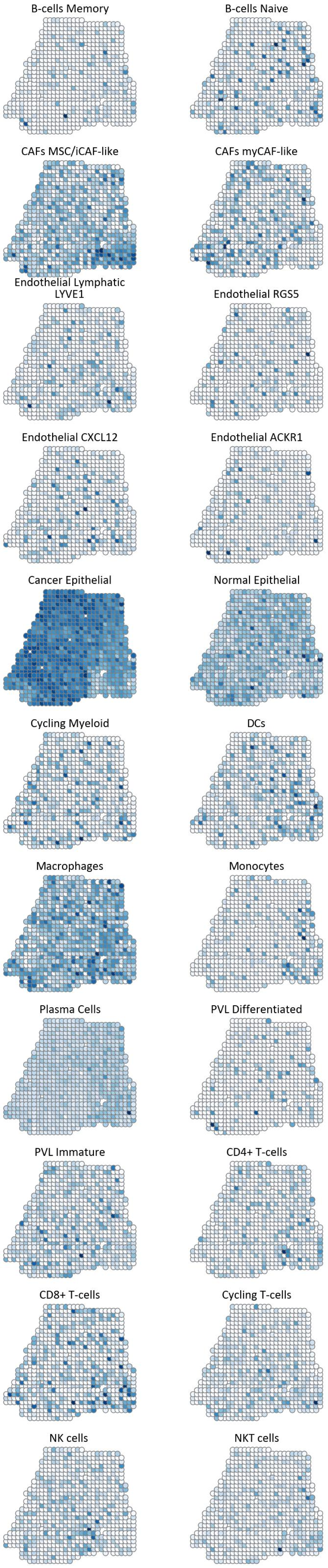

B-cells Memory
B-cells Naive
CAFs MSC/iCAF-like
CAFs myCAF-like
Endothelial Lymphatic LYVE1
Endothelial RGS5
Endothelial CXCL12
Endothelial ACKR1
Cancer Epithelial
Normal Epithelial
Cycling Myeloid
DCs
Macrophages
Monocytes
Plasma Cells
PVL Differentiated
PVL Immature
CD4+ T-cells
CD8+ T-cells
Cycling T-cells
NK cells
NKT cells

minor_A3

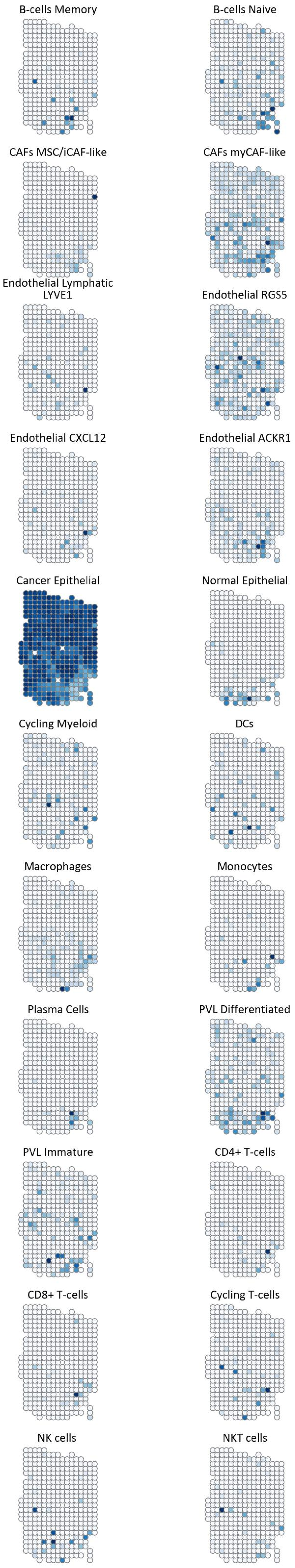
B-cells Memory
B-cells Naive
CAFs MSC/iCAF-like
CAFs myCAF-like
Endothelial Lymphatic LYVE1
Endothelial RGS5
Endothelial CXCL12
Endothelial ACKR1
Cancer Epithelial
Normal Epithelial
Cycling Myeloid
DCs
Macrophages
Monocytes
Plasma Cells
PVL Differentiated
PVL Immature
CD4+ T-cells
CD8+ T-cells
Cycling T-cells
NK cells
NKT cells

minor_D5

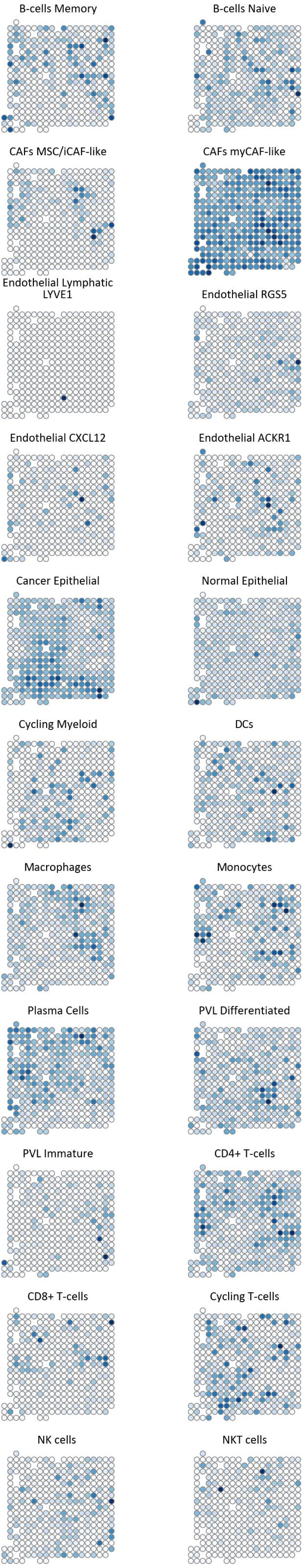

B-cells Memory
B-cells Naive
CAFs MSC/iCAF-like
CAFs myCAF-like
Endothelial Lymphatic LYVE1
Endothelial RGS5
Endothelial CXCL12
Endothelial ACKR1
Cancer Epithelial
Normal Epithelial
Cycling Myeloid
DCs
Macrophages
Monocytes
Plasma Cells
PVL Differentiated
PVL Immature
CD4+ T-cells
CD8+ T-cells
Cycling T-cells
NK cells
NKT cells

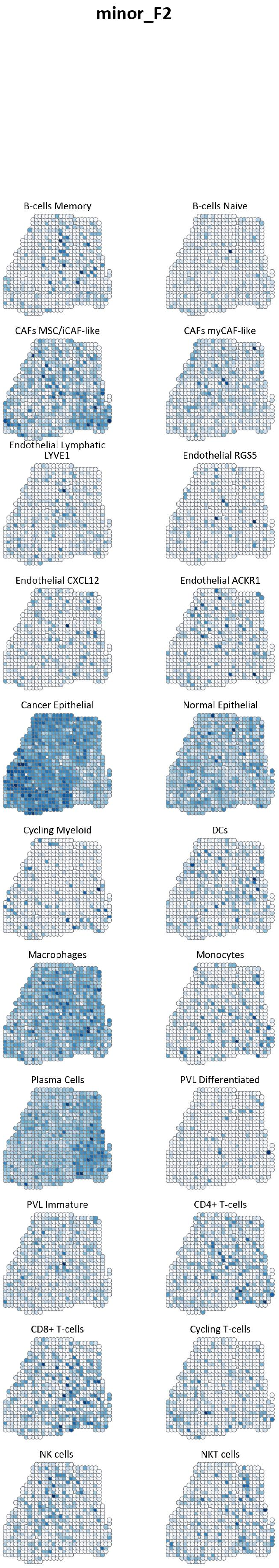
minor_F2
B-cells Memory
B-cells Naive
CAFs MSC/iCAF-like
CAFs myCAF-like
Endothelial Lymphatic LYVE1
Endothelial RGS5
Endothelial CXCL12
Endothelial ACKR1
Cancer Epithelial
Normal Epithelial
Cycling Myeloid
DCs
Macrophages
Monocytes
Plasma Cells
PVL Differentiated
PVL Immature
CD4+ T-cells
CD8+ T-cells
Cycling T-cells
NK cells
NKT cells

minor_G3

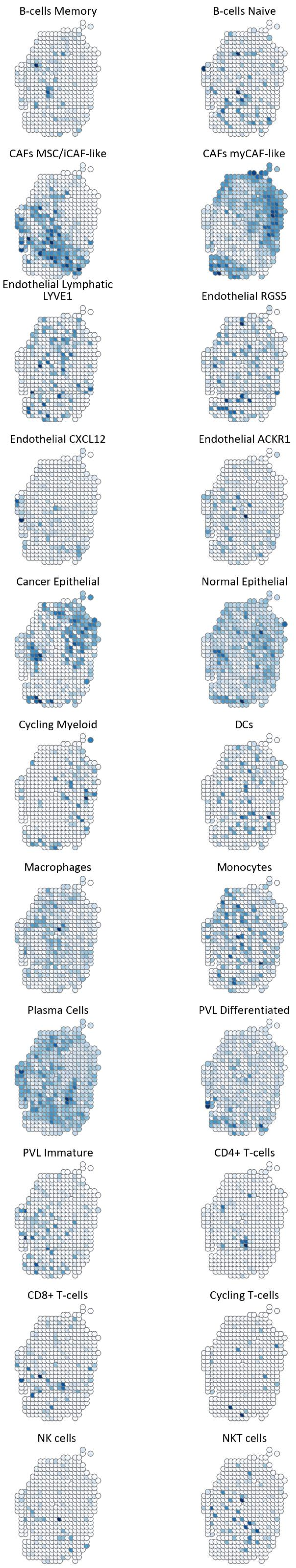
B-cells Memory
B-cells Naive
CAFs MSC/iCAF-like
CAFs myCAF-like
Endothelial Lymphatic LYVE1
Endothelial RGS5
Endothelial CXCL12
Endothelial ACKR1
Cancer Epithelial
Normal Epithelial
Cycling Myeloid
DCs
Macrophages
Monocytes
Plasma Cells
PVL Differentiated
PVL Immature
CD4+ T-cells
CD8+ T-cells
Cycling T-cells
NK cells
NKT cells

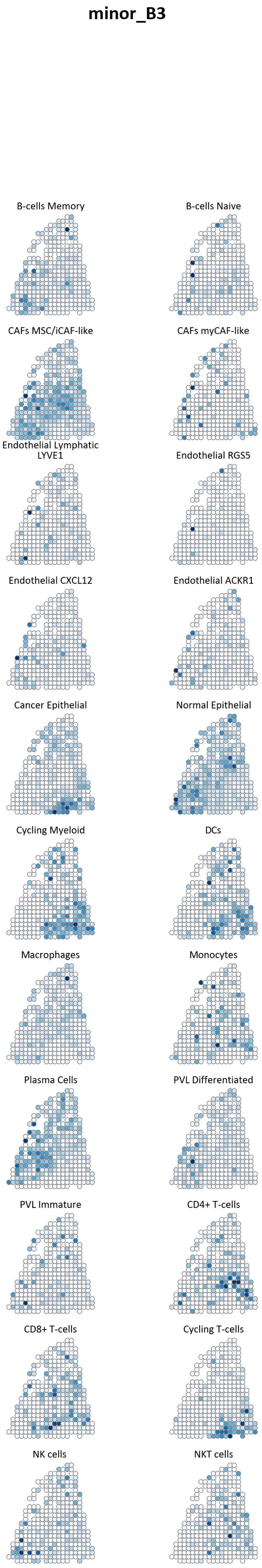
minor_B3
B-cells Memory
B-cells Naive
CAFs MSC/iCAF-like
CAFs myCAF-like
Endothelial Lymphatic LYVE1
Endothelial RGS5
Endothelial CXCL12
Endothelial ACKR1
Cancer Epithelial
Normal Epithelial
Cycling Myeloid
DCs
Macrophages
Monocytes
Plasma Cells
PVL Differentiated
PVL Immature
CD4+ T-cells
CD8+ T-cells
Cycling T-cells
NK cells
NKT cells

minor_B6

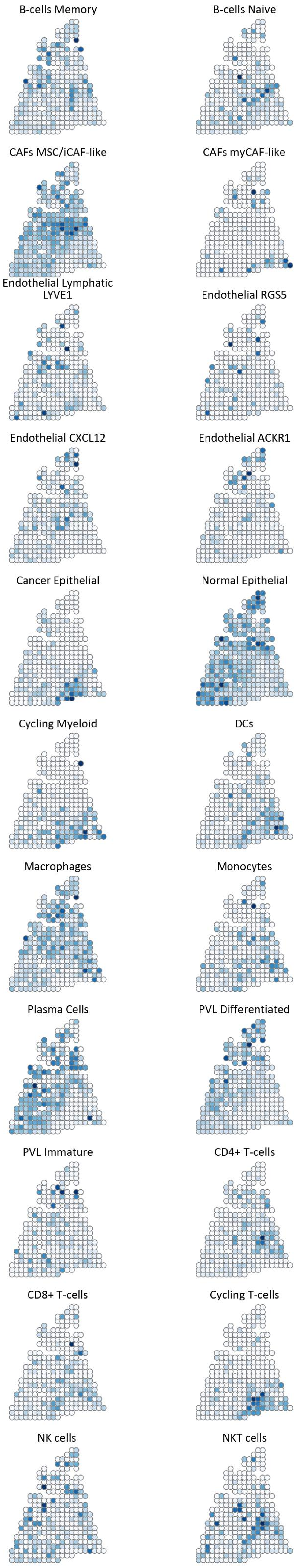
B-cells Memory
B-cells Naive
CAFs MSC/iCAF-like
CAFs myCAF-like
Endothelial Lymphatic LYVE1
Endothelial RGS5
Endothelial CXCL12
Endothelial ACKR1
Cancer Epithelial
Normal Epithelial
Cycling Myeloid
DCs
Macrophages
Monocytes
Plasma Cells
PVL Differentiated
PVL Immature
CD4+ T-cells
CD8+ T-cells
Cycling T-cells
NK cells
NKT cells

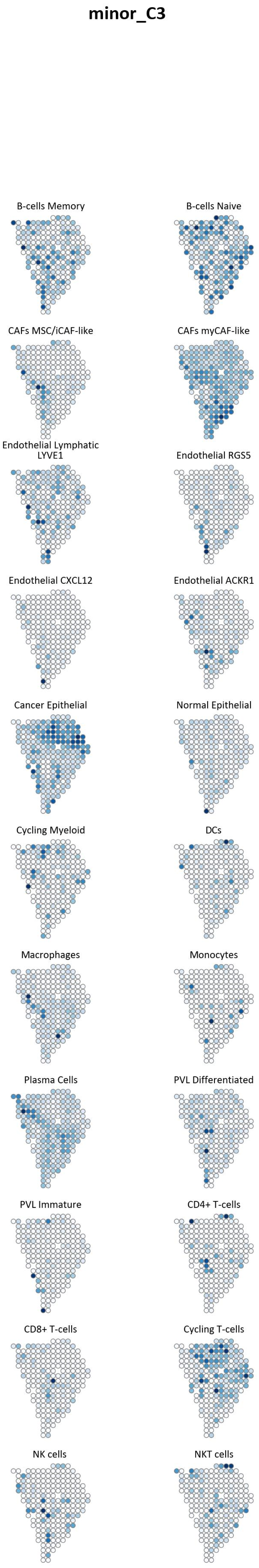
minor_C3
B-cells Memory
B-cells Naive
CAFs MSC/iCAF-like
CAFs myCAF-like
Endothelial Lymphatic LYVE1
Endothelial RGS5
Endothelial CXCL12
Endothelial ACKR1
Cancer Epithelial
Normal Epithelial
Cycling Myeloid
DCs
Macrophages
Monocytes
Plasma Cells
PVL Differentiated
PVL Immature
CD4+ T-cells
CD8+ T-cells
Cycling T-cells
NK cells
NKT cells

subset_E2

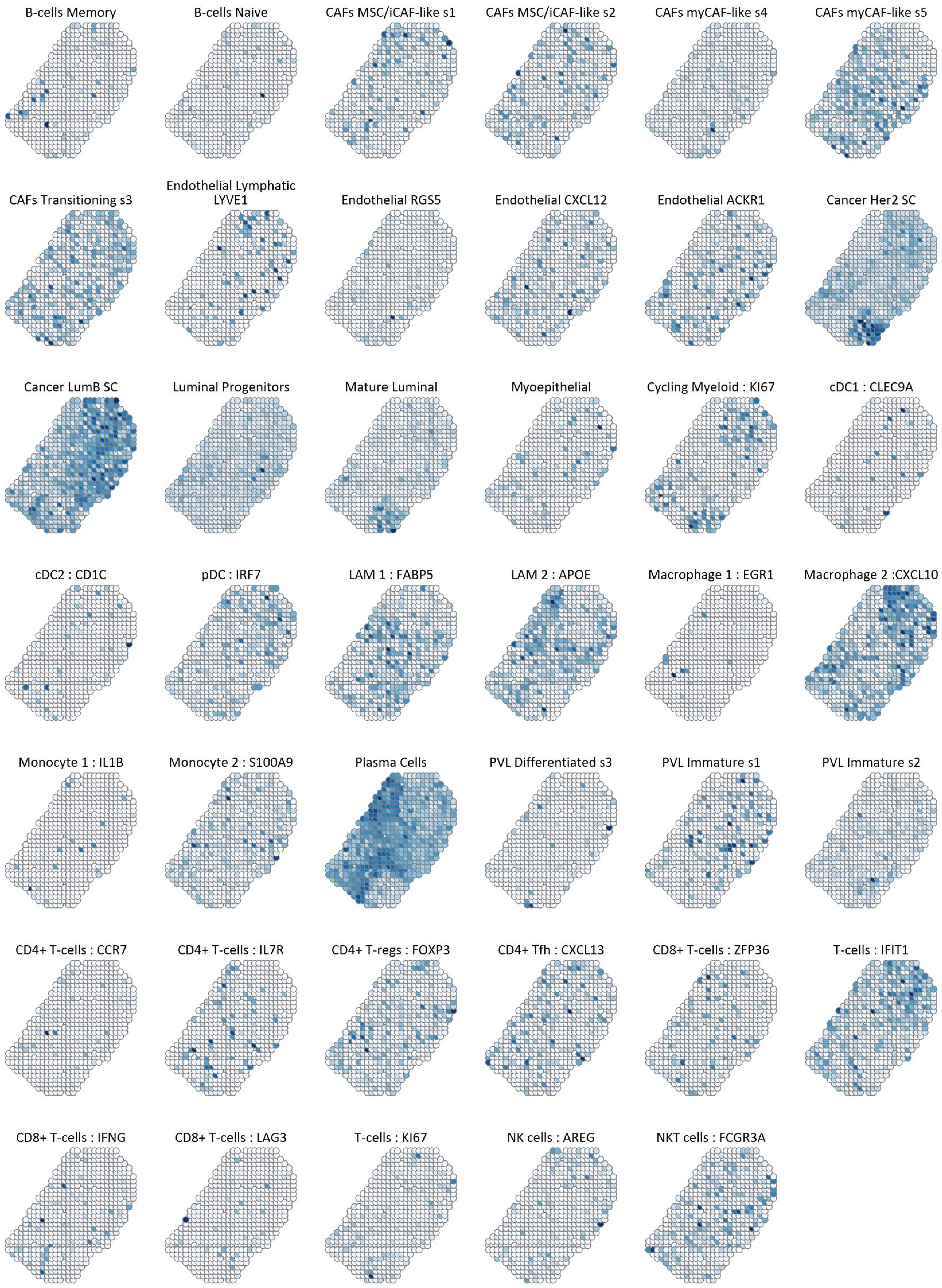
B-cells Memory
B-cells Naive
CAFs MSC/iCAF-like s1
CAFs MSC/iCAF-like s2
CAFs myCAF-like s4
CAFs myCAF-like s5
CAFs Transitioning s3
Endothelial Lymphatic LYVE1
Endothelial RGS5
Endothelial CXCL12
Endothelial ACKR1
Cancer Her2 SC
Cancer LumB SC
Luminal Progenitors
Mature Luminal
Myoepithelial
Cycling Myeloid : KI67
cDC1 : CLEC9A
cDC2 : CD1C
pDC : IRF7
LAM 1 : FABP5
LAM 2 : APOE
Macrophage 1 : EGR1
Macrophage 2 :CXCL10
Monocyte 1 : IL1B
Monocyte 2 : S100A9
Plasma Cells
PVL Differentiated s3
PVL Immature s1
PVL Immature s2
CD4+ T-cells : CCR7
CD4+ T-cells : IL7R
CD4+ T-regs : FOXP3
CD4+ Tfh : CXCL13
CD8+ T-cells : ZFP36
T-cells : IFIT1
CD8+ T-cells : IFNG
CD8+ T-cells : LAG3
T-cells : KI67
NK cells : AREG
NKT cells : FCGR3A

subset_G2

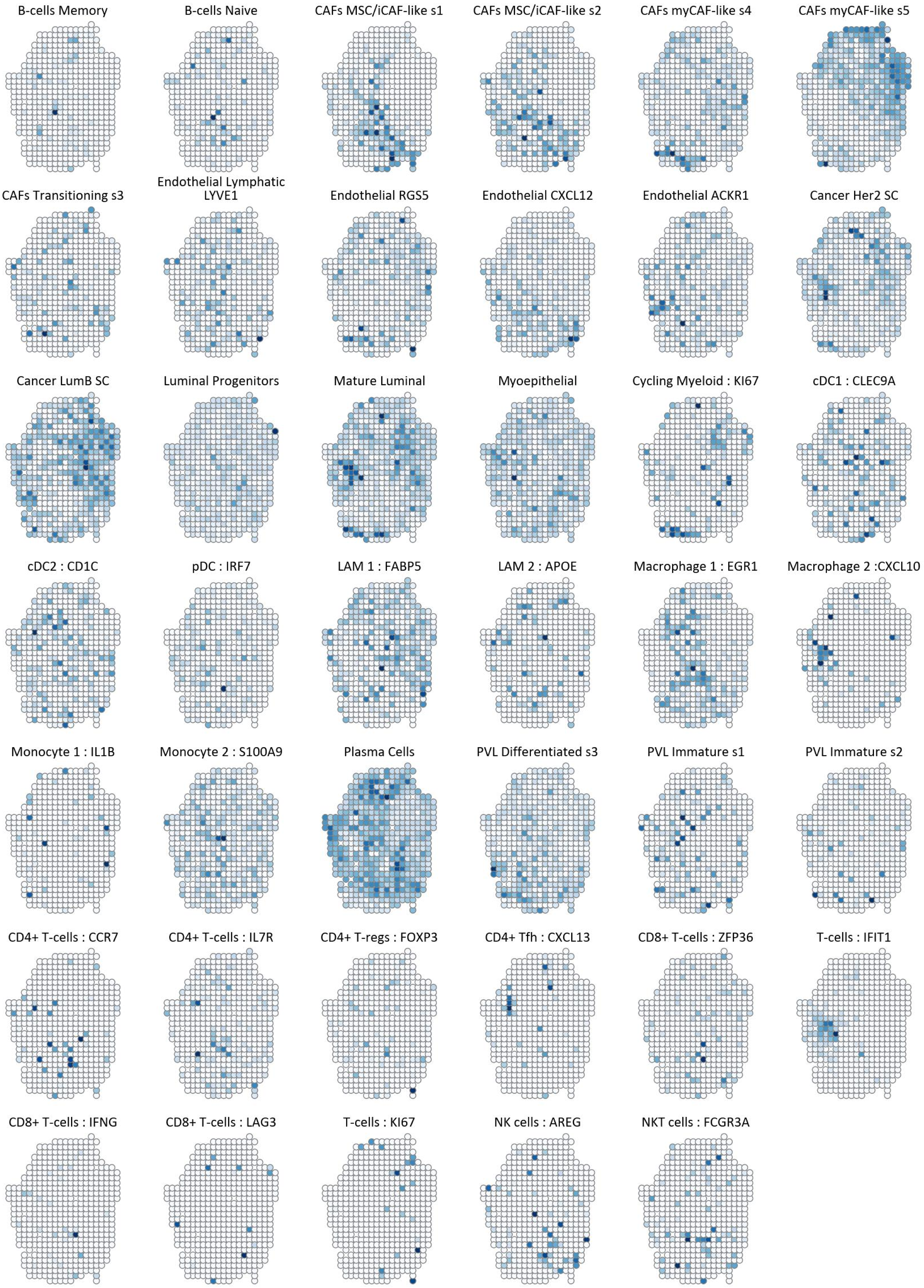
B-cells Memory
B-cells Naive
CAFs MSC/iCAF-like s1
CAFs MSC/iCAF-like s2
CAFs myCAF-like s4
CAFs myCAF-like s5
CAFs Transitioning s3
Endothelial Lymphatic LYVE1
Endothelial RGS5
Endothelial CXCL12
Endothelial ACKR1
Cancer Her2 SC
Cancer LumB SC
Luminal Progenitors
Mature Luminal
Myoepithelial
Cycling Myeloid : KI67
cDC1 : CLEC9A
cDC2 : CD1C
pDC : IRF7
LAM 1 : FABP5
LAM 2 : APOE
Macrophage 1 : EGR1
Macrophage 2 :CXCL10
Monocyte 1 : IL1B
Monocyte 2 : S100A9
Plasma Cells
PVL Differentiated s3
PVL Immature s1
PVL Immature s2
CD4+ T-cells : CCR7
CD4+ T-cells : IL7R
CD4+ T-regs : FOXP3
CD4+ Tfh : CXCL13
CD8+ T-cells : ZFP36
T-cells : IFIT1
CD8+ T-cells : IFNG
CD8+ T-cells : LAG3
T-cells : KI67
NK cells : AREG
NKT cells : FCGR3A

subset_C4

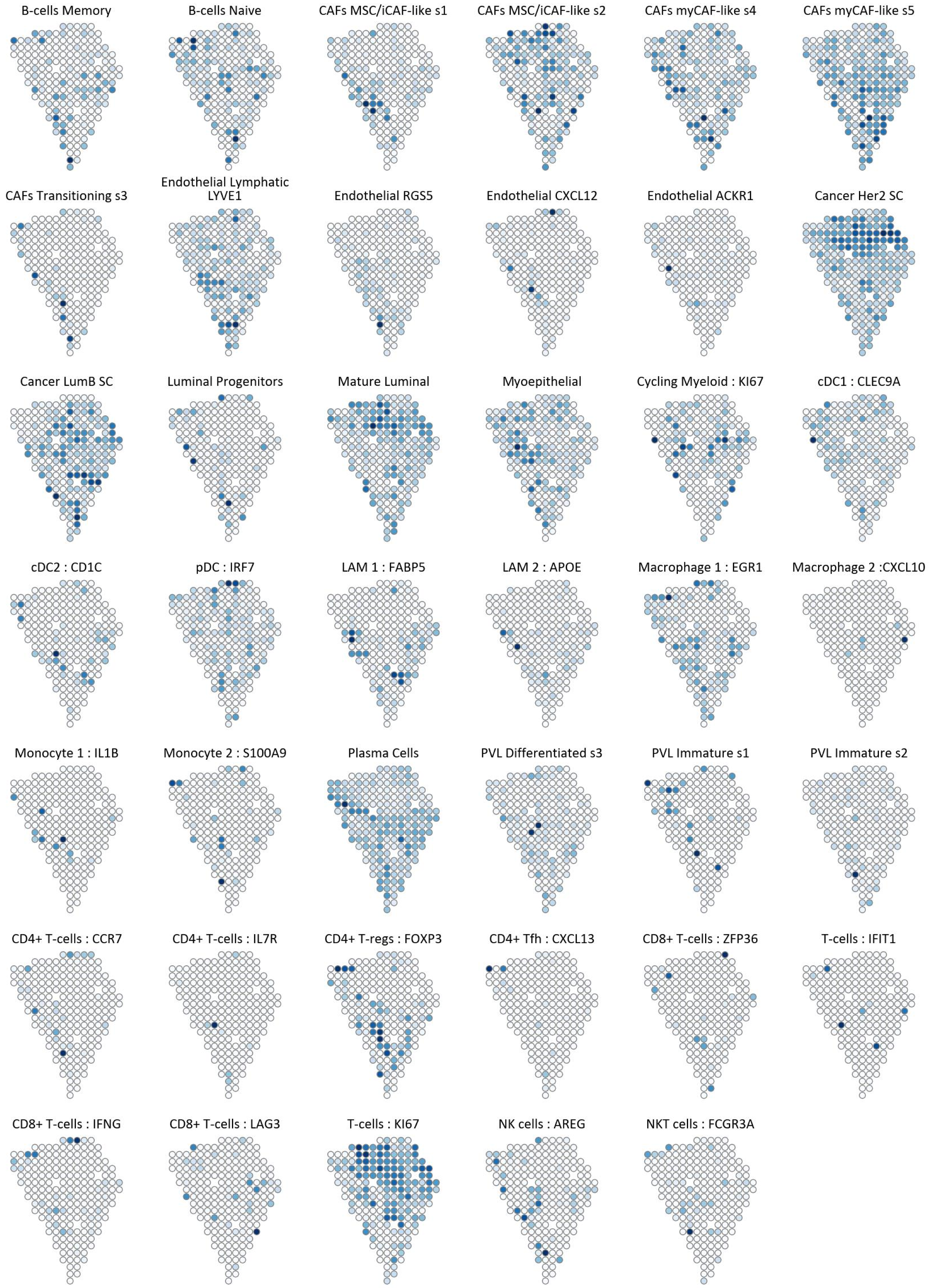

B-cells Memory
B-cells Naive
CAFs MSC/iCAF-like s1
CAFs MSC/iCAF-like s2
CAFs myCAF-like s4
CAFs myCAF-like s5
CAFs Transitioning s3
Endothelial Lymphatic LYVE1
Endothelial RGS5
Endothelial CXCL12
Endothelial ACKR1
Cancer Her2 SC
Cancer LumB SC
Luminal Progenitors
Mature Luminal
Myoepithelial
Cycling Myeloid : KI67
cDC1 : CLEC9A
cDC2 : CD1C
pDC : IRF7
LAM 1 : FABP5
LAM 2 : APOE
Macrophage 1 : EGR1
Macrophage 2 :CXCL10
Monocyte 1 : IL1B
Monocyte 2 : S100A9
Plasma Cells
PVL Differentiated s3
PVL Immature s1
PVL Immature s2
CD4+ T-cells : CCR7
CD4+ T-cells : IL7R
CD4+ T-regs : FOXP3
CD4+ Tfh : CXCL13
CD8+ T-cells : ZFP36
T-cells : IFIT1
CD8+ T-cells : IFNG
CD8+ T-cells : LAG3
T-cells : KI67
NK cells : AREG
NKT cells : FCGR3A

subset_C2

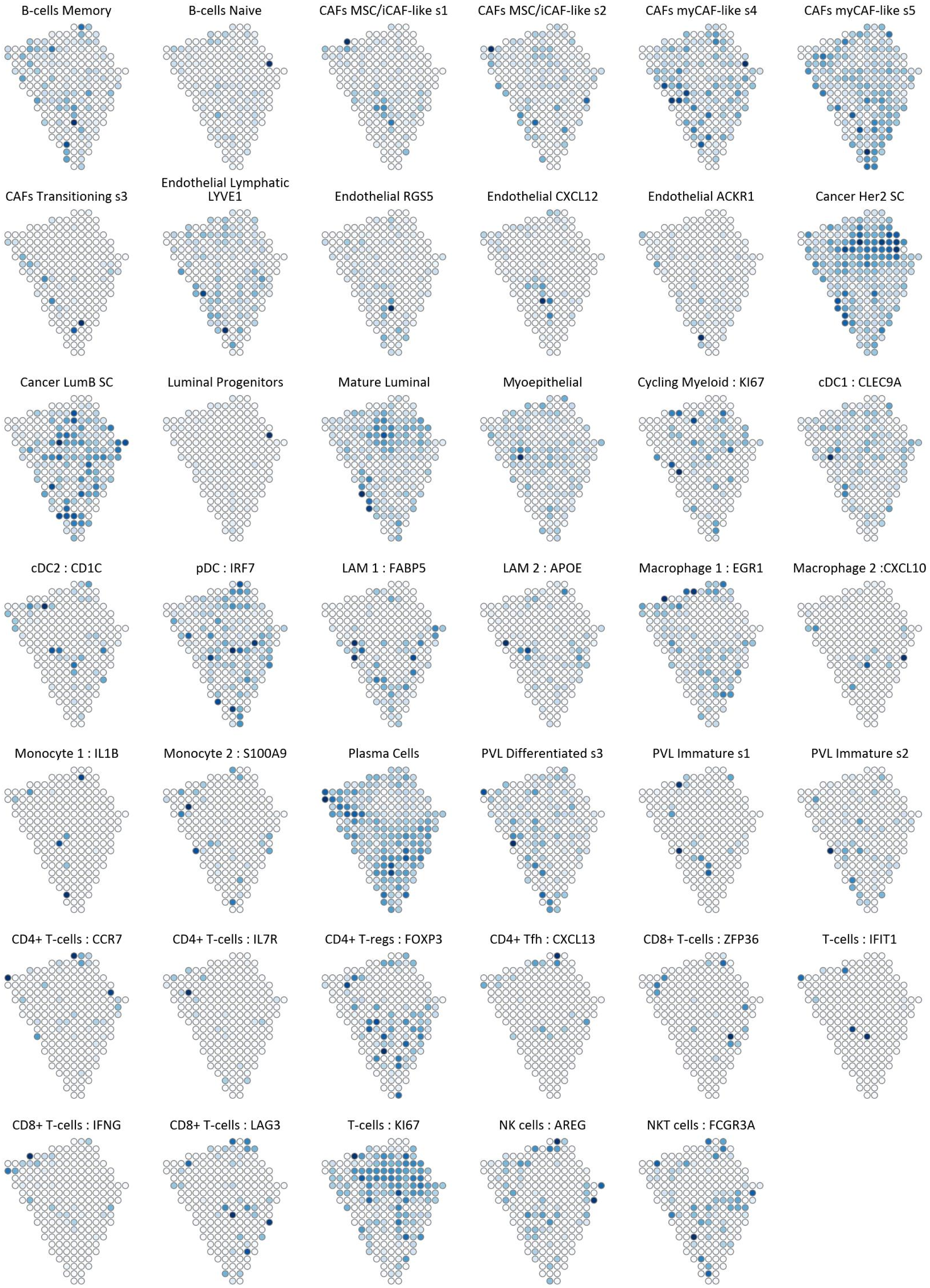
B-cells Memory
B-cells Naive
CAFs MSC/iCAF-like s1
CAFs MSC/iCAF-like s2
CAFs myCAF-like s4
CAFs myCAF-like s5
CAFs Transitioning s3
Endothelial Lymphatic LYVE1
Endothelial RGS5
Endothelial CXCL12
Endothelial ACKR1
Cancer Her2 SC
Cancer LumB SC
Luminal Progenitors
Mature Luminal
Myoepithelial
Cycling Myeloid : KI67
cDC1 : CLEC9A
cDC2 : CD1C
pDC : IRF7
LAM 1 : FABP5
LAM 2 : APOE
Macrophage 1 : EGR1
Macrophage 2 :CXCL10
Monocyte 1 : IL1B
Monocyte 2 : S100A9
Plasma Cells
PVL Differentiated s3
PVL Immature s1
PVL Immature s2
CD4+ T-cells : CCR7
CD4+ T-cells : IL7R
CD4+ T-regs : FOXP3
CD4+ Tfh : CXCL13
CD8+ T-cells : ZFP36
T-cells : IFIT1
CD8+ T-cells : IFNG
CD8+ T-cells : LAG3
T-cells : KI67
NK cells : AREG
NKT cells : FCGR3A

subset_C5

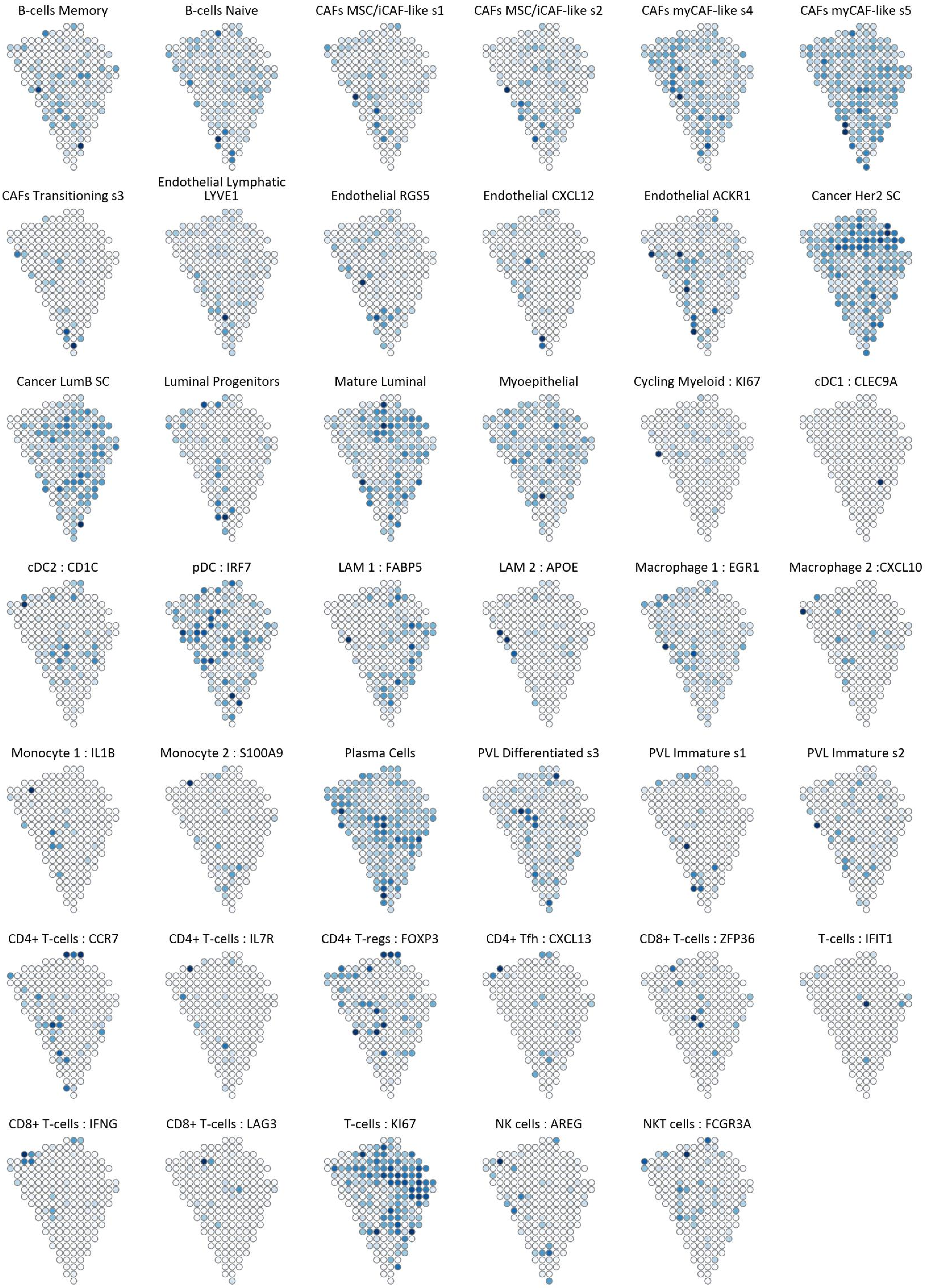

B-cells Memory
B-cells Naive
CAFs MSC/iCAF-like s1
CAFs MSC/iCAF-like s2
CAFs myCAF-like s4
CAFs myCAF-like s5
CAFs Transitioning s3
Endothelial Lymphatic LYVE1
Endothelial RGS5
Endothelial CXCL12
Endothelial ACKR1
Cancer Her2 SC
Cancer LumB SC
Luminal Progenitors
Mature Luminal
Myoepithelial
Cycling Myeloid : KI67
cDC1 : CLEC9A
cDC2 : CD1C
pDC : IRF7
LAM 1 : FABP5
LAM 2 : APOE
Macrophage 1 : EGR1
Macrophage 2 :CXCL10
Monocyte 1 : IL1B
Monocyte 2 : S100A9
Plasma Cells
PVL Differentiated s3
PVL Immature s1
PVL Immature s2
CD4+ T-cells : CCR7
CD4+ T-cells : IL7R
CD4+ T-regs : FOXP3
CD4+ Tfh : CXCL13
CD8+ T-cells : ZFP36
T-cells : IFIT1
CD8+ T-cells : IFNG
CD8+ T-cells : LAG3
T-cells : KI67
NK cells : AREG
NKT cells : FCGR3A

subset_E1

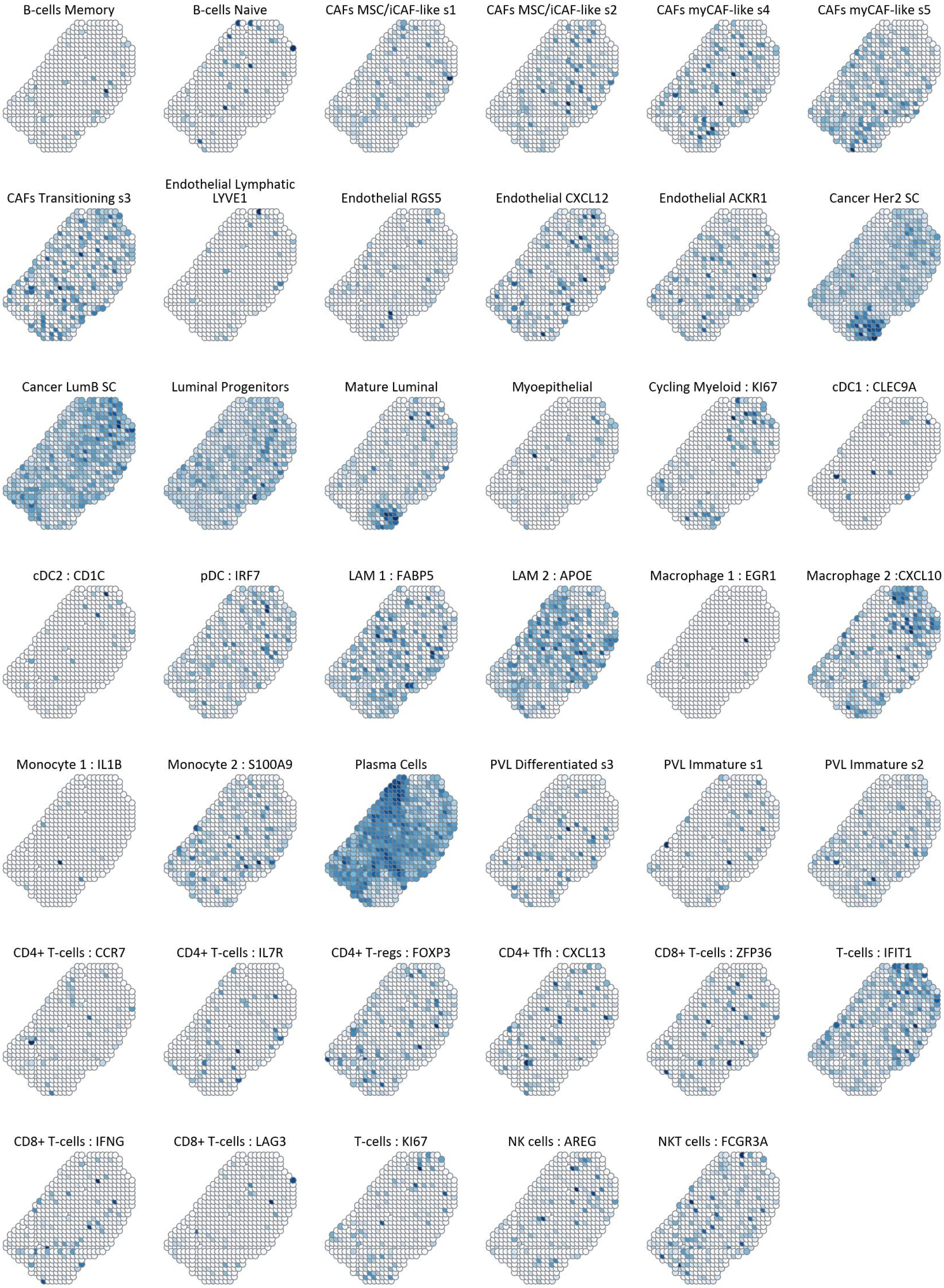
B-cells Memory
B-cells Naive
CAFs MSC/iCAF-like s1
CAFs MSC/iCAF-like s2
CAFs myCAF-like s4
CAFs myCAF-like s5
CAFs Transitioning s3
Endothelial Lymphatic LYVE1
Endothelial RGS5
Endothelial CXCL12
Endothelial ACKR1
Cancer Her2 SC
Cancer LumB SC
Luminal Progenitors
Mature Luminal
Myoepithelial
Cycling Myeloid : KI67
cDC1 : CLEC9A
cDC2 : CD1C
pDC : IRF7
LAM 1 : FABP5
LAM 2 : APOE
Macrophage 1 : EGR1
Macrophage 2 :CXCL10
Monocyte 1 : IL1B
Monocyte 2 : S100A9
Plasma Cells
PVL Differentiated s3
PVL Immature s1
PVL Immature s2
CD4+ T-cells : CCR7
CD4+ T-cells : IL7R
CD4+ T-regs : FOXP3
CD4+ Tfh : CXCL13
CD8+ T-cells : ZFP36
T-cells : IFIT1
CD8+ T-cells : IFNG
CD8+ T-cells : LAG3
T-cells : KI67
NK cells : AREG
NKT cells : FCGR3A

subset_B4

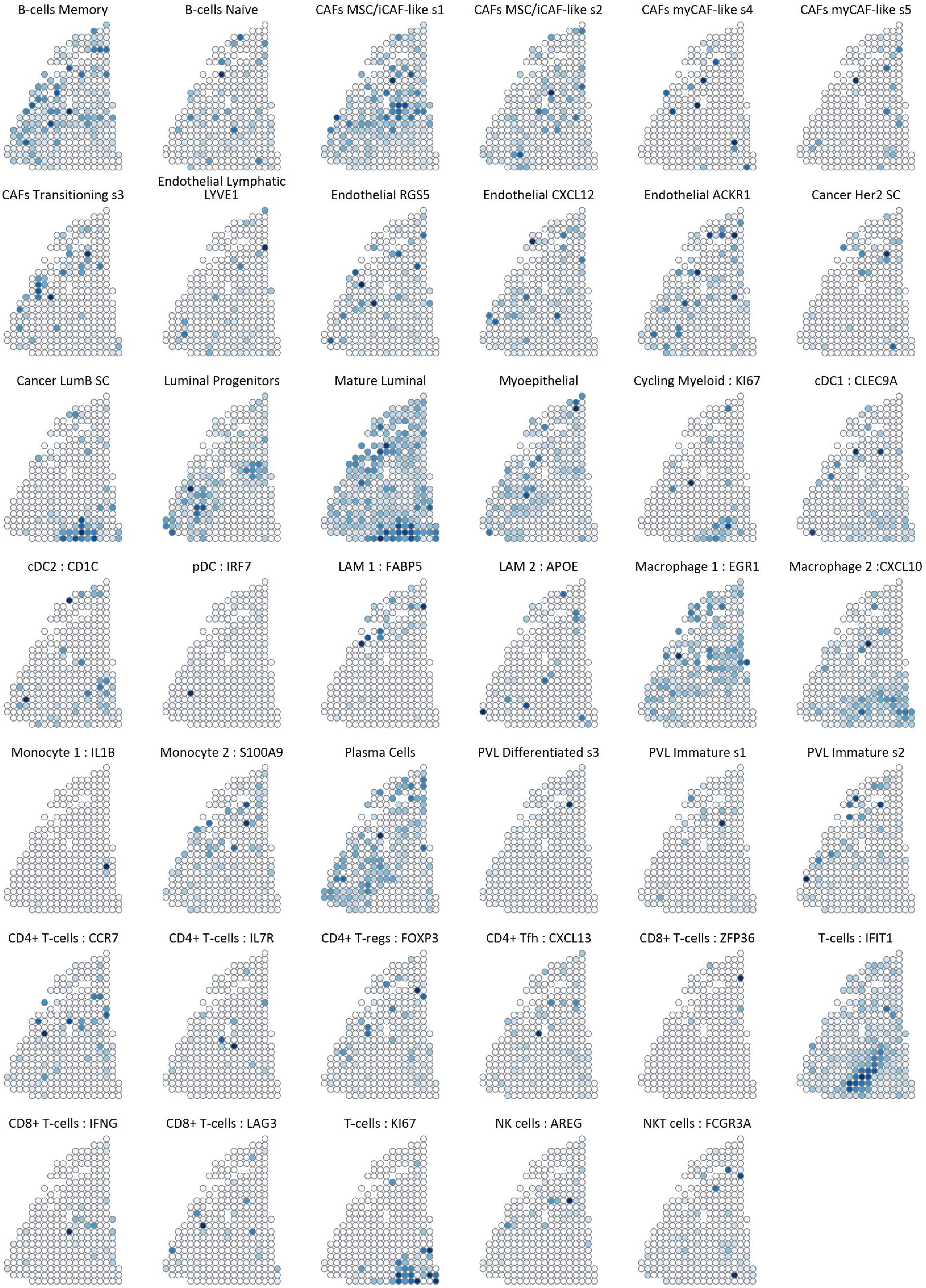

B-cells Memory
B-cells Naive
CAFs MSC/iCAF-like s1
CAFs MSC/iCAF-like s2
CAFs myCAF-like s4
CAFs myCAF-like s5
CAFs Transitioning s3
Endothelial Lymphatic LYVE1
Endothelial RGS5
Endothelial CXCL12
Endothelial ACKR1
Cancer Her2 SC
Cancer LumB SC
Luminal Progenitors
Mature Luminal
Myoepithelial
Cycling Myeloid : KI67
cDC1 : CLEC9A
cDC2 : CD1C
pDC : IRF7
LAM 1 : FABP5
LAM 2 : APOE
Macrophage 1 : EGR1
Macrophage 2 :CXCL10
Monocyte 1 : IL1B
Monocyte 2 : S100A9
Plasma Cells
PVL Differentiated s3
PVL Immature s1
PVL Immature s2
CD4+ T-cells : CCR7
CD4+ T-cells : IL7R
CD4+ T-regs : FOXP3
CD4+ Tfh : CXCL13
CD8+ T-cells : ZFP36
T-cells : IFIT1
CD8+ T-cells : IFNG
CD8+ T-cells : LAG3
T-cells : KI67
NK cells : AREG
NKT cells : FCGR3A

subset_A2

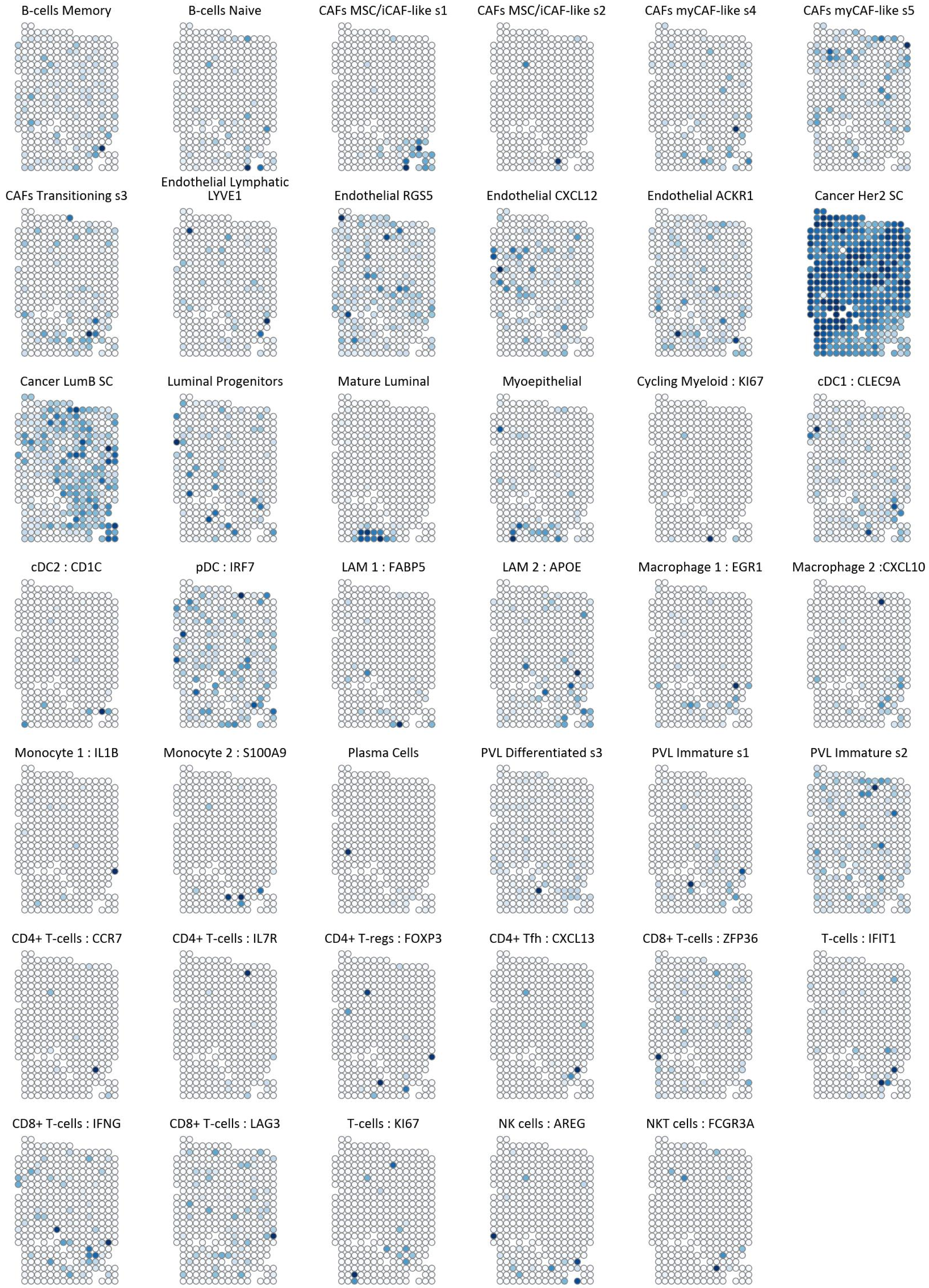
B-cells Memory
B-cells Naive
CAFs MSC/iCAF-like s1
CAFs MSC/iCAF-like s2
CAFs myCAF-like s4
CAFs myCAF-like s5
CAFs Transitioning s3
Endothelial Lymphatic LYVE1
Endothelial RGS5
Endothelial CXCL12
Endothelial ACKR1
Cancer Her2 SC
Cancer LumB SC
Luminal Progenitors
Mature Luminal
Myoepithelial
Cycling Myeloid : KI67
cDC1 : CLEC9A
cDC2 : CD1C
pDC : IRF7
LAM 1 : FABP5
LAM 2 : APOE
Macrophage 1 : EGR1
Macrophage 2 :CXCL10
Monocyte 1 : IL1B
Monocyte 2 : S100A9
Plasma Cells
PVL Differentiated s3
PVL Immature s1
PVL Immature s2
CD4+ T-cells : CCR7
CD4+ T-cells : IL7R
CD4+ T-regs : FOXP3
CD4+ Tfh : CXCL13
CD8+ T-cells : ZFP36
T-cells : IFIT1
CD8+ T-cells : IFNG
CD8+ T-cells : LAG3
T-cells : KI67
NK cells : AREG
NKT cells : FCGR3A

subset_A4

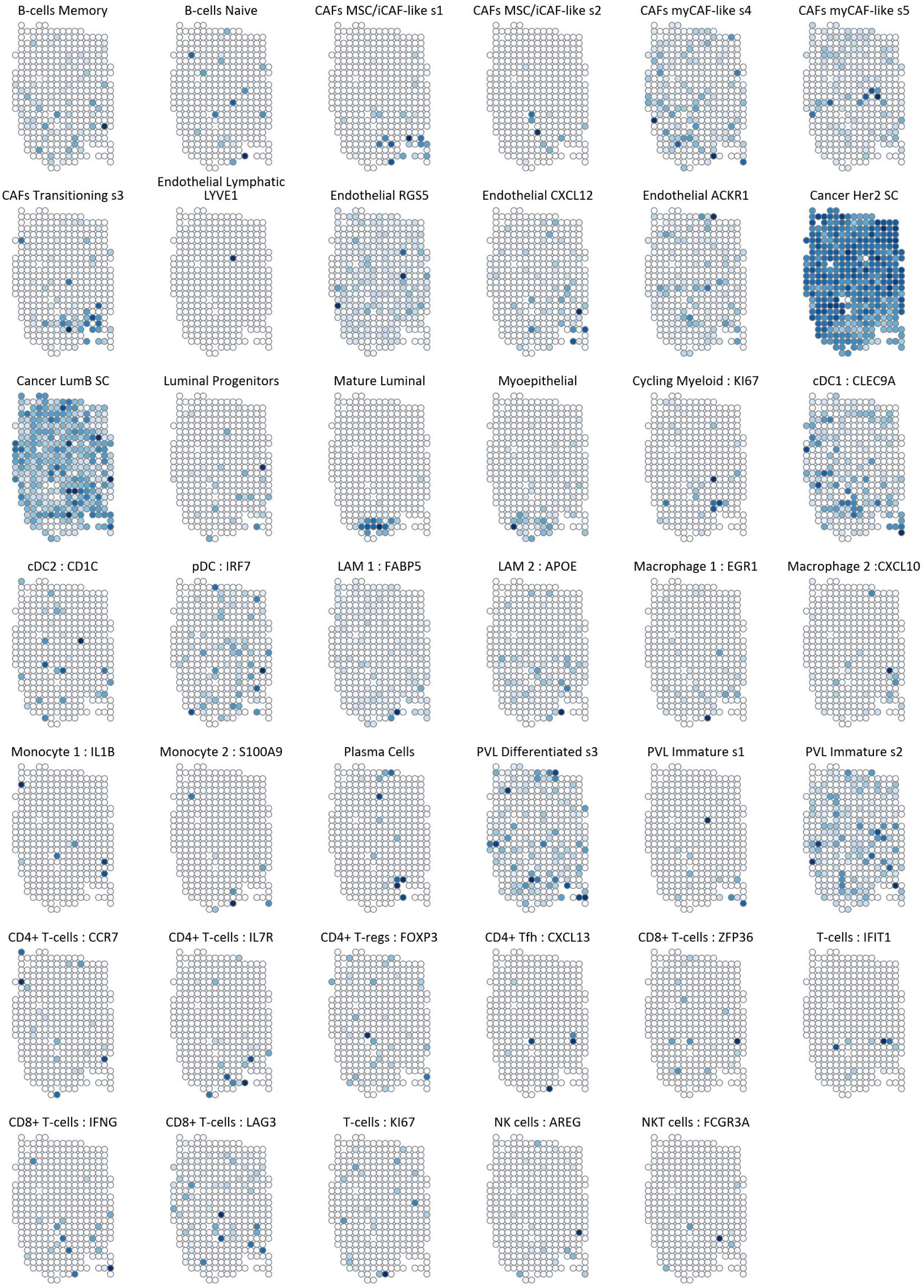
B-cells Memory
B-cells Naive
CAFs MSC/iCAF-like s1
CAFs MSC/iCAF-like s2
CAFs myCAF-like s4
CAFs myCAF-like s5
CAFs Transitioning s3
Endothelial Lymphatic LYVE1
Endothelial RGS5
Endothelial CXCL12
Endothelial ACKR1
Cancer Her2 SC
Cancer LumB SC
Luminal Progenitors
Mature Luminal
Myoepithelial
Cycling Myeloid : KI67
cDC1 : CLEC9A
cDC2 : CD1C
pDC : IRF7
LAM 1 : FABP5
LAM 2 : APOE
Macrophage 1 : EGR1
Macrophage 2 :CXCL10
Monocyte 1 : IL1B
Monocyte 2 : S100A9
Plasma Cells
PVL Differentiated s3
PVL Immature s1
PVL Immature s2
CD4+ T-cells : CCR7
CD4+ T-cells : IL7R
CD4+ T-regs : FOXP3
CD4+ Tfh : CXCL13
CD8+ T-cells : ZFP36
T-cells : IFIT1
CD8+ T-cells : IFNG
CD8+ T-cells : LAG3
T-cells : KI67
NK cells : AREG
NKT cells : FCGR3A

subset_A1

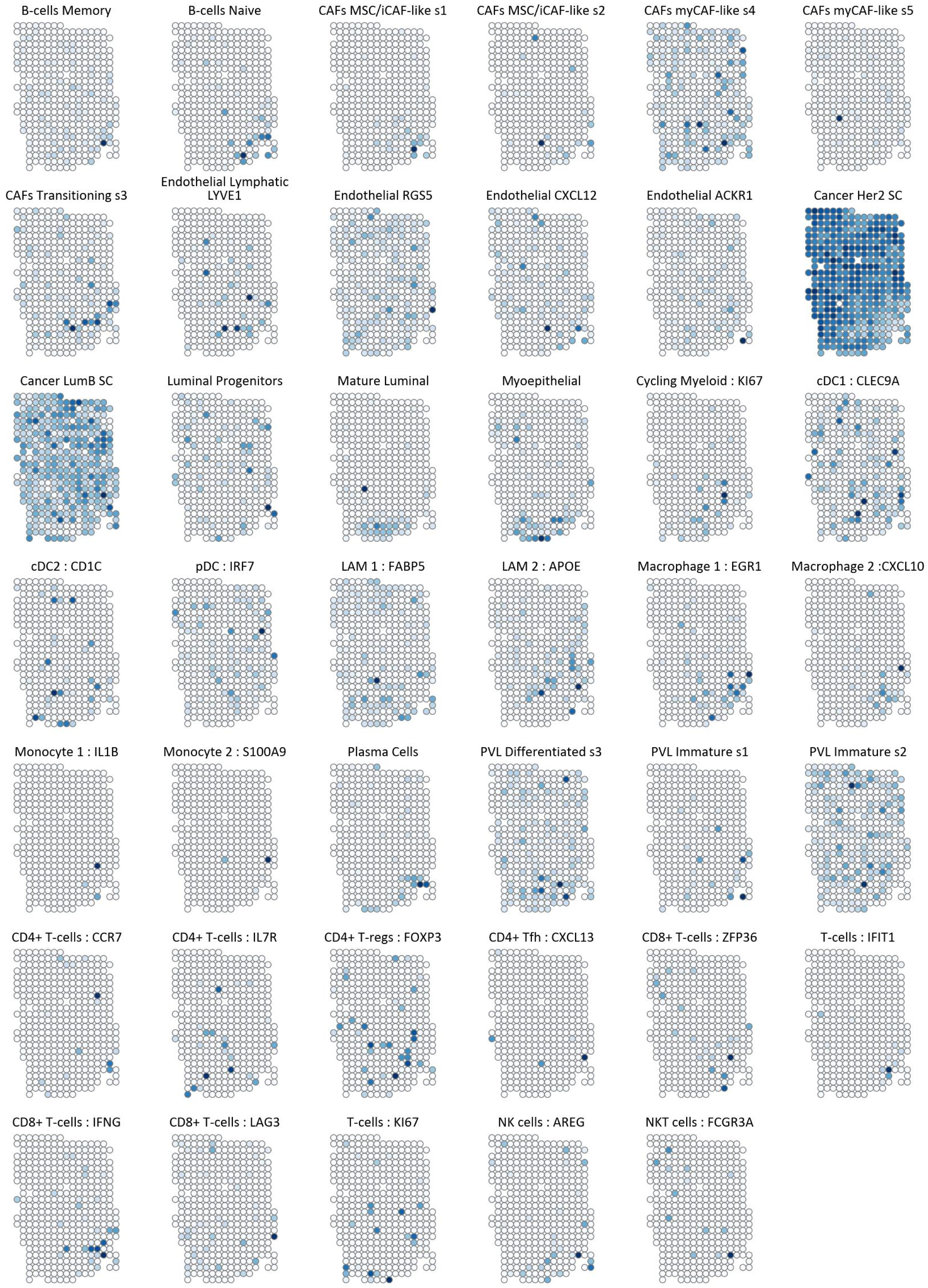
B-cells Memory
B-cells Naive
CAFs MSC/iCAF-like s1
CAFs MSC/iCAF-like s2
CAFs myCAF-like s4
CAFs myCAF-like s5
CAFs Transitioning s3
Endothelial Lymphatic LYVE1
Endothelial RGS5
Endothelial CXCL12
Endothelial ACKR1
Cancer Her2 SC
Cancer LumB SC
Luminal Progenitors
Mature Luminal
Myoepithelial
Cycling Myeloid : KI67
cDC1 : CLEC9A
cDC2 : CD1C
pDC : IRF7
LAM 1 : FABP5
LAM 2 : APOE
Macrophage 1 : EGR1
Macrophage 2 :CXCL10
Monocyte 1 : IL1B
Monocyte 2 : S100A9
Plasma Cells
PVL Differentiated s3
PVL Immature s1
PVL Immature s2
CD4+ T-cells : CCR7
CD4+ T-cells : IL7R
CD4+ T-regs : FOXP3
CD4+ Tfh : CXCL13
CD8+ T-cells : ZFP36
T-cells : IFIT1
CD8+ T-cells : IFNG
CD8+ T-cells : LAG3
T-cells : KI67
NK cells : AREG
NKT cells : FCGR3A

subset_D1

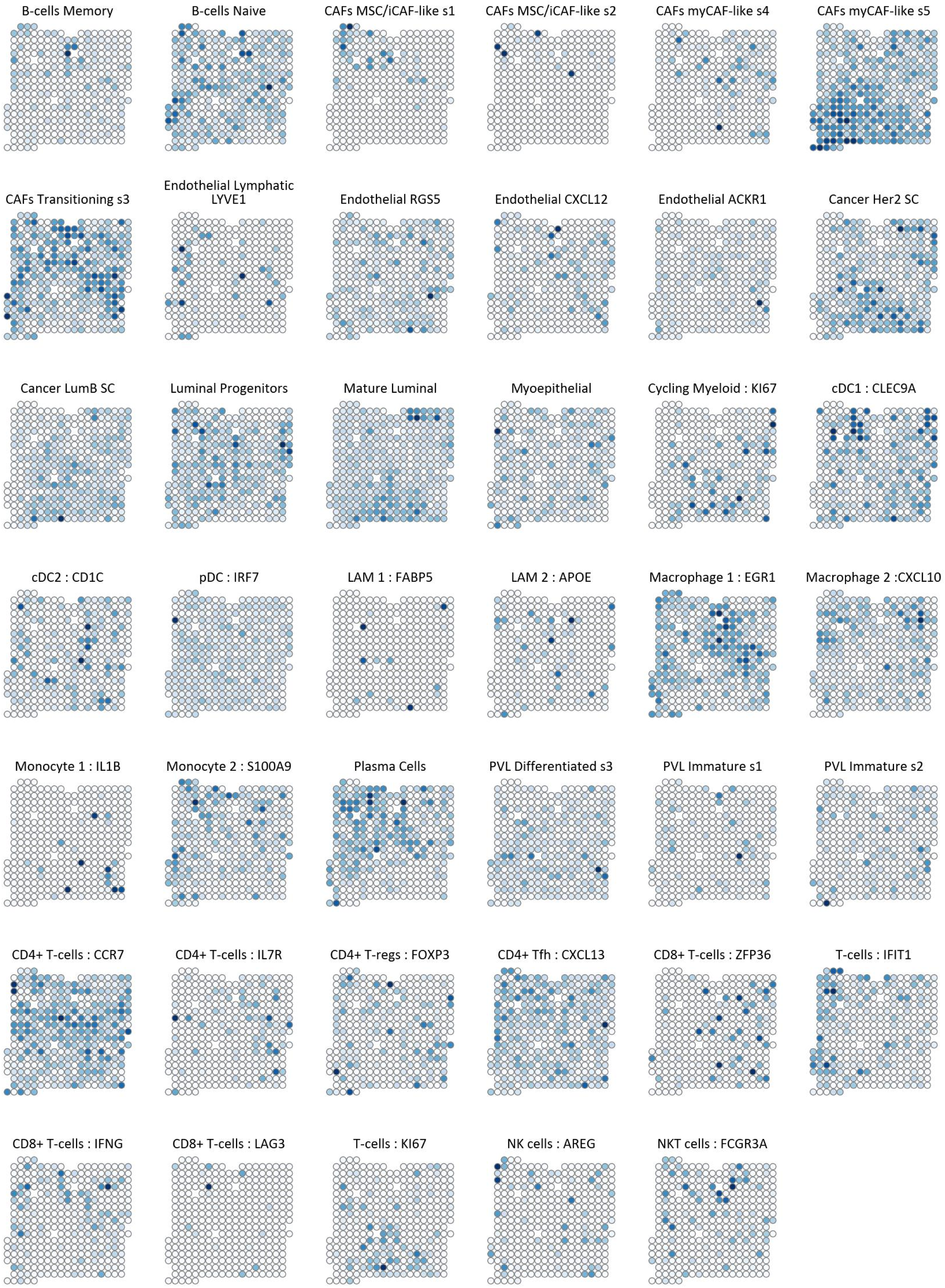
B-cells Memory
B-cells Naive
CAFs MSC/iCAF-like s1
CAFs MSC/iCAF-like s2
CAFs myCAF-like s4
CAFs myCAF-like s5
CAFs Transitioning s3
Endothelial Lymphatic LYVE1
Endothelial RGS5
Endothelial CXCL12
Endothelial ACKR1
Cancer Her2 SC
Cancer LumB SC
Luminal Progenitors
Mature Luminal
Myoepithelial
Cycling Myeloid : KI67
cDC1 : CLEC9A
cDC2 : CD1C
pDC : IRF7
LAM 1 : FABP5
LAM 2 : APOE
Macrophage 1 : EGR1
Macrophage 2 :CXCL10
Monocyte 1 : IL1B
Monocyte 2 : S100A9
Plasma Cells
PVL Differentiated s3
PVL Immature s1
PVL Immature s2
CD4+ T-cells : CCR7
CD4+ T-cells : IL7R
CD4+ T-regs : FOXP3
CD4+ Tfh : CXCL13
CD8+ T-cells : ZFP36
T-cells : IFIT1
CD8+ T-cells : IFNG
CD8+ T-cells : LAG3
T-cells : KI67
NK cells : AREG
NKT cells : FCGR3A

subset_C1

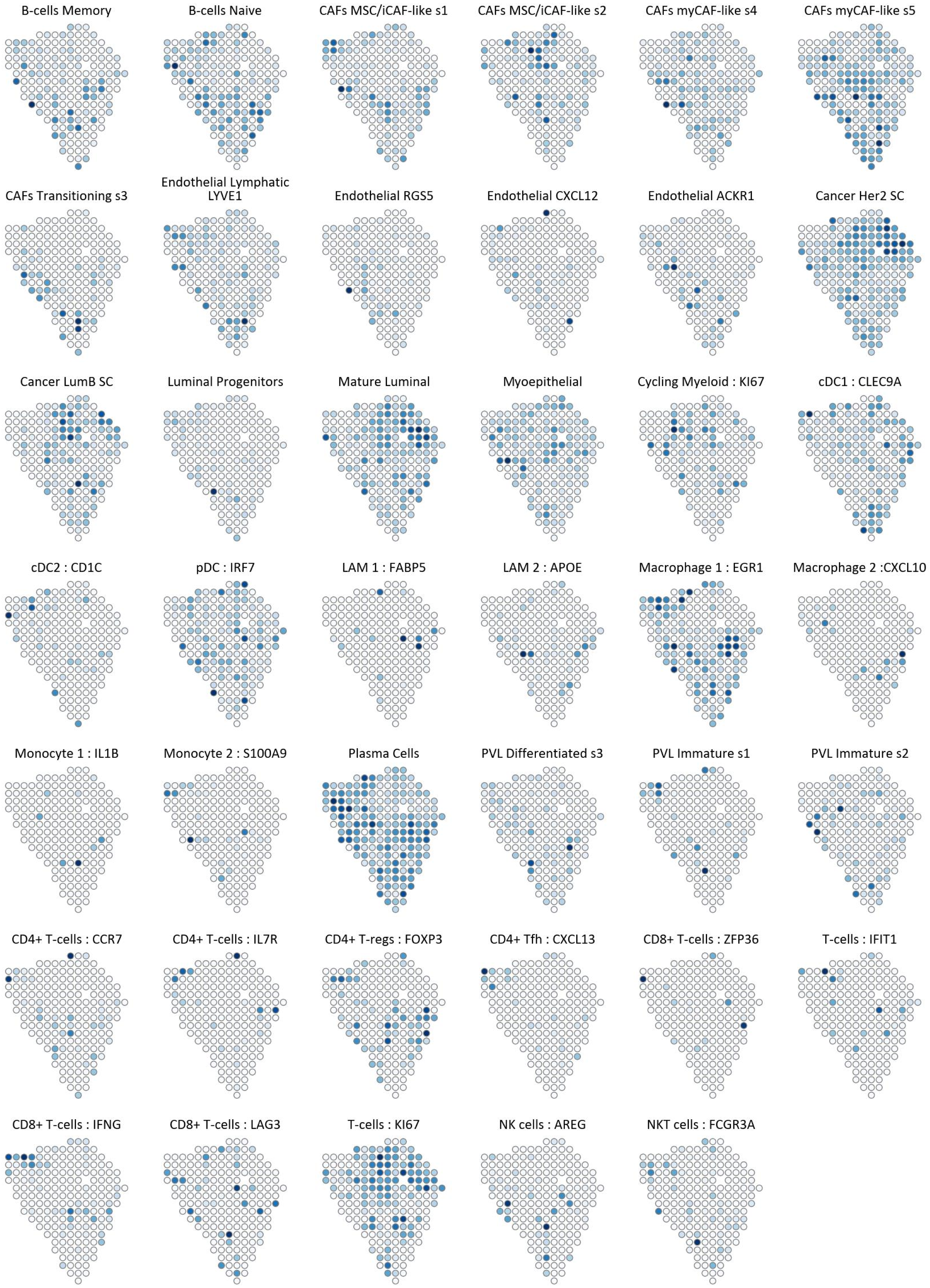
B-cells Memory
B-cells Naive
CAFs MSC/iCAF-like s1
CAFs MSC/iCAF-like s2
CAFs myCAF-like s4
CAFs myCAF-like s5
CAFs Transitioning s3
Endothelial Lymphatic LYVE1
Endothelial RGS5
Endothelial CXCL12
Endothelial ACKR1
Cancer Her2 SC
Cancer LumB SC
Luminal Progenitors
Mature Luminal
Myoepithelial
Cycling Myeloid : KI67
cDC1 : CLEC9A
cDC2 : CD1C
pDC : IRF7
LAM 1 : FABP5
LAM 2 : APOE
Macrophage 1 : EGR1
Macrophage 2 :CXCL10
Monocyte 1 : IL1B
Monocyte 2 : S100A9
Plasma Cells
PVL Differentiated s3
PVL Immature s1
PVL Immature s2
CD4+ T-cells : CCR7
CD4+ T-cells : IL7R
CD4+ T-regs : FOXP3
CD4+ Tfh : CXCL13
CD8+ T-cells : ZFP36
T-cells : IFIT1
CD8+ T-cells : IFNG
CD8+ T-cells : LAG3
T-cells : KI67
NK cells : AREG
NKT cells : FCGR3A

subset_D6

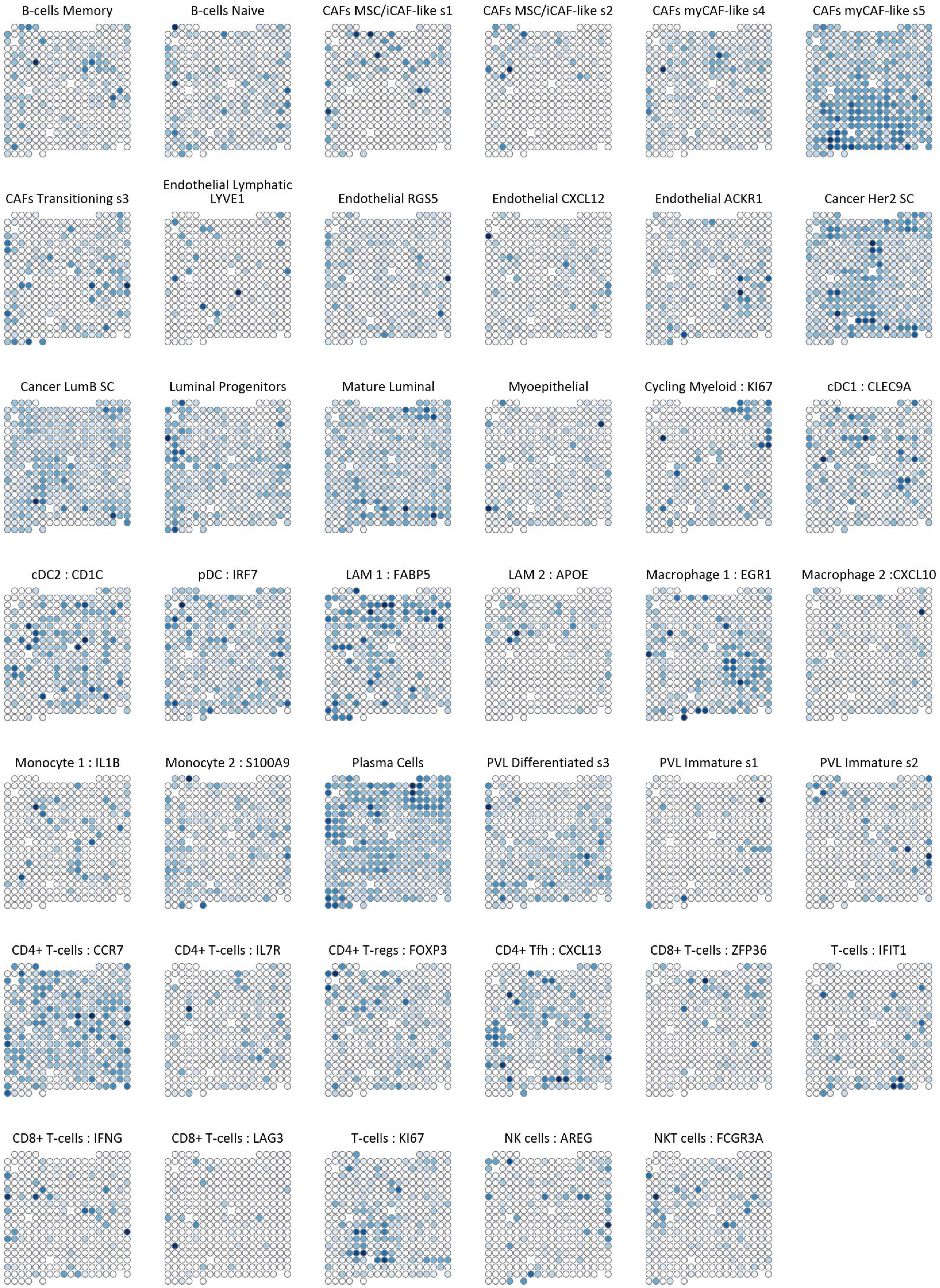
B-cells Memory
B-cells Naive
CAFs MSC/iCAF-like s1
CAFs MSC/iCAF-like s2
CAFs myCAF-like s4
CAFs myCAF-like s5
CAFs Transitioning s3
Endothelial Lymphatic LYVE1
Endothelial RGS5
Endothelial CXCL12
Endothelial ACKR1
Cancer Her2 SC
Cancer LumB SC
Luminal Progenitors
Mature Luminal
Myoepithelial
Cycling Myeloid : KI67
cDC1 : CLEC9A
cDC2 : CD1C
pDC : IRF7
LAM 1 : FABP5
LAM 2 : APOE
Macrophage 1 : EGR1
Macrophage 2 :CXCL10
Monocyte 1 : IL1B
Monocyte 2 : S100A9
Plasma Cells
PVL Differentiated s3
PVL Immature s1
PVL Immature s2
CD4+ T-cells : CCR7
CD4+ T-cells : IL7R
CD4+ T-regs : FOXP3
CD4+ Tfh : CXCL13
CD8+ T-cells : ZFP36
T-cells : IFIT1
CD8+ T-cells : IFNG
CD8+ T-cells : LAG3
T-cells : KI67
NK cells : AREG
NKT cells : FCGR3A

subset_A5

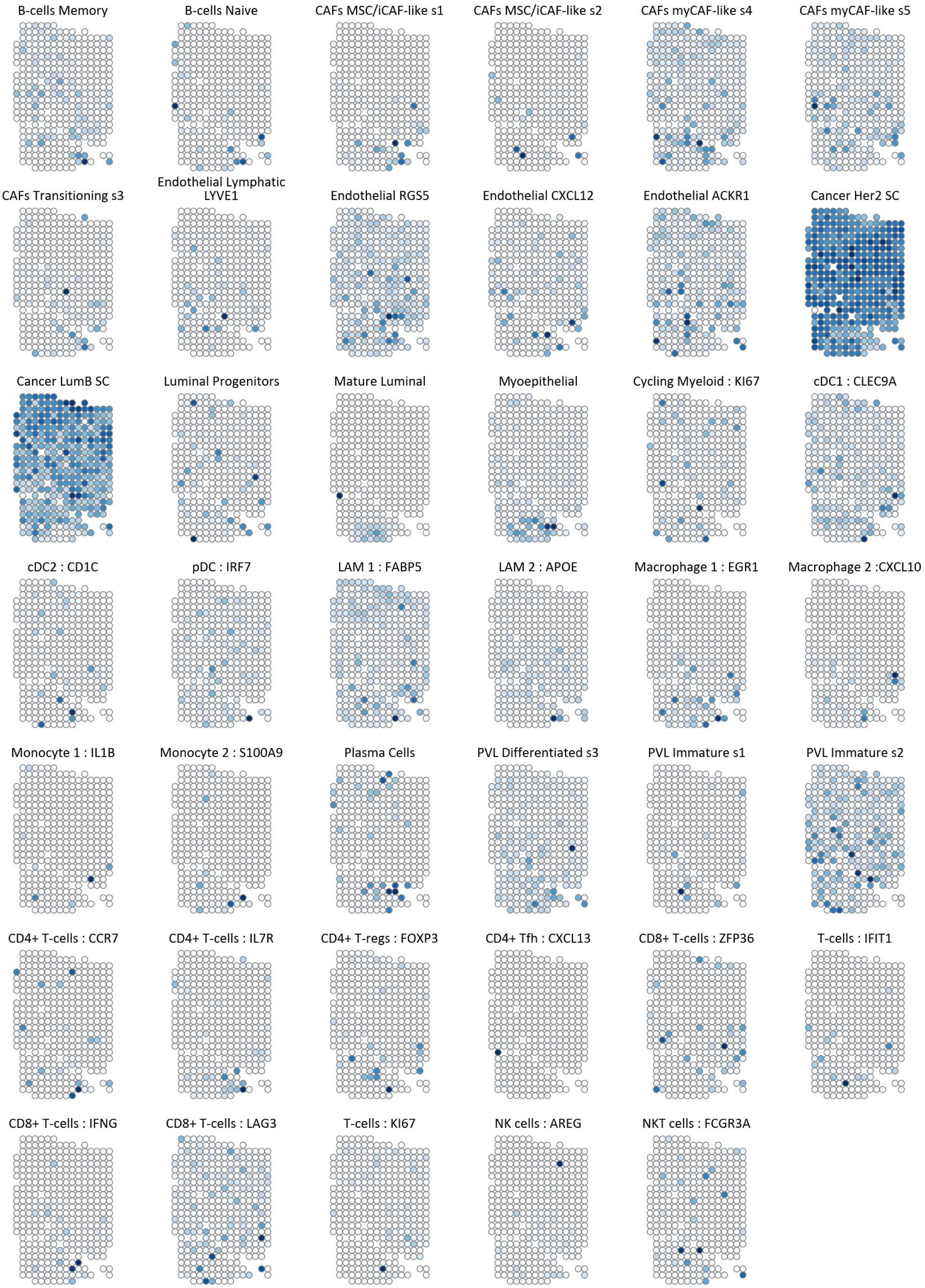
B-cells Memory
B-cells Naive
CAFs MSC/iCAF-like s1
CAFs MSC/iCAF-like s2
CAFs myCAF-like s4
CAFs myCAF-like s5
CAFs Transitioning s3
Endothelial Lymphatic LYVE1
Endothelial RGS5
Endothelial CXCL12
Endothelial ACKR1
Cancer Her2 SC
Cancer LumB SC
Luminal Progenitors
Mature Luminal
Myoepithelial
Cycling Myeloid : KI67
cDC1 : CLEC9A
cDC2 : CD1C
pDC : IRF7
LAM 1 : FABP5
LAM 2 : APOE
Macrophage 1 : EGR1
Macrophage 2 :CXCL10
Monocyte 1 : IL1B
Monocyte 2 : S100A9
Plasma Cells
PVL Differentiated s3
PVL Immature s1
PVL Immature s2
CD4+ T-cells : CCR7
CD4+ T-cells : IL7R
CD4+ T-regs : FOXP3
CD4+ Tfh : CXCL13
CD8+ T-cells : ZFP36
T-cells : IFIT1
CD8+ T-cells : IFNG
CD8+ T-cells : LAG3
T-cells : KI67
NK cells : AREG
NKT cells : FCGR3A

subset_H2

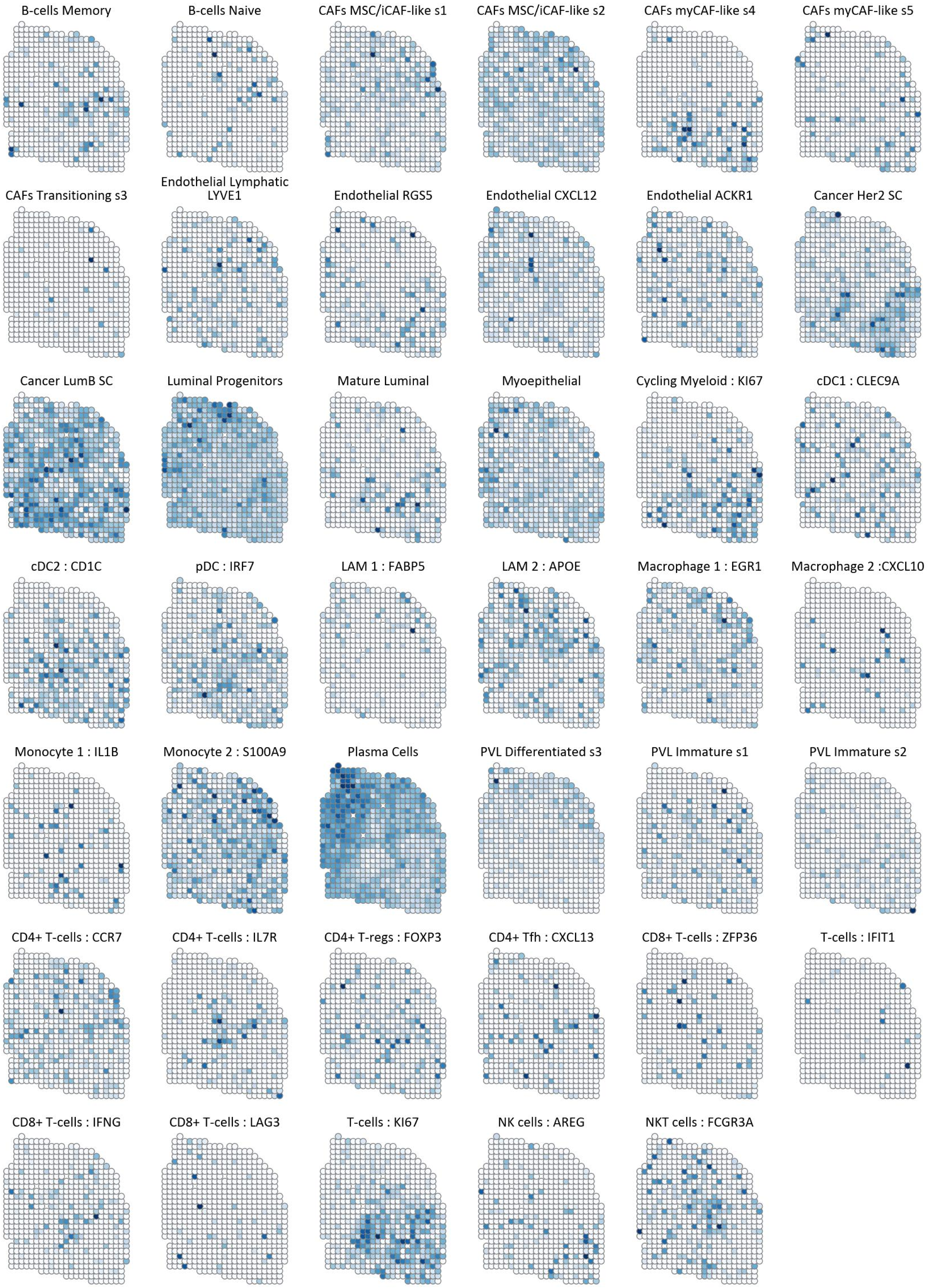

B-cells Memory
B-cells Naive
CAFs MSC/iCAF-like s1
CAFs MSC/iCAF-like s2
CAFs myCAF-like s4
CAFs myCAF-like s5
CAFs Transitioning s3
Endothelial Lymphatic LYVE1
Endothelial RGS5
Endothelial CXCL12
Endothelial ACKR1
Cancer Her2 SC
Cancer LumB SC
Luminal Progenitors
Mature Luminal
Myoepithelial
Cycling Myeloid : KI67
cDC1 : CLEC9A
cDC2 : CD1C
pDC : IRF7
LAM 1 : FABP5
LAM 2 : APOE
Macrophage 1 : EGR1
Macrophage 2 :CXCL10
Monocyte 1 : IL1B
Monocyte 2 : S100A9
Plasma Cells
PVL Differentiated s3
PVL Immature s1
PVL Immature s2
CD4+ T-cells : CCR7
CD4+ T-cells : IL7R
CD4+ T-regs : FOXP3
CD4+ Tfh : CXCL13
CD8+ T-cells : ZFP36
T-cells : IFIT1
CD8+ T-cells : IFNG
CD8+ T-cells : LAG3
T-cells : KI67
NK cells : AREG
NKT cells : FCGR3A

subset_D2

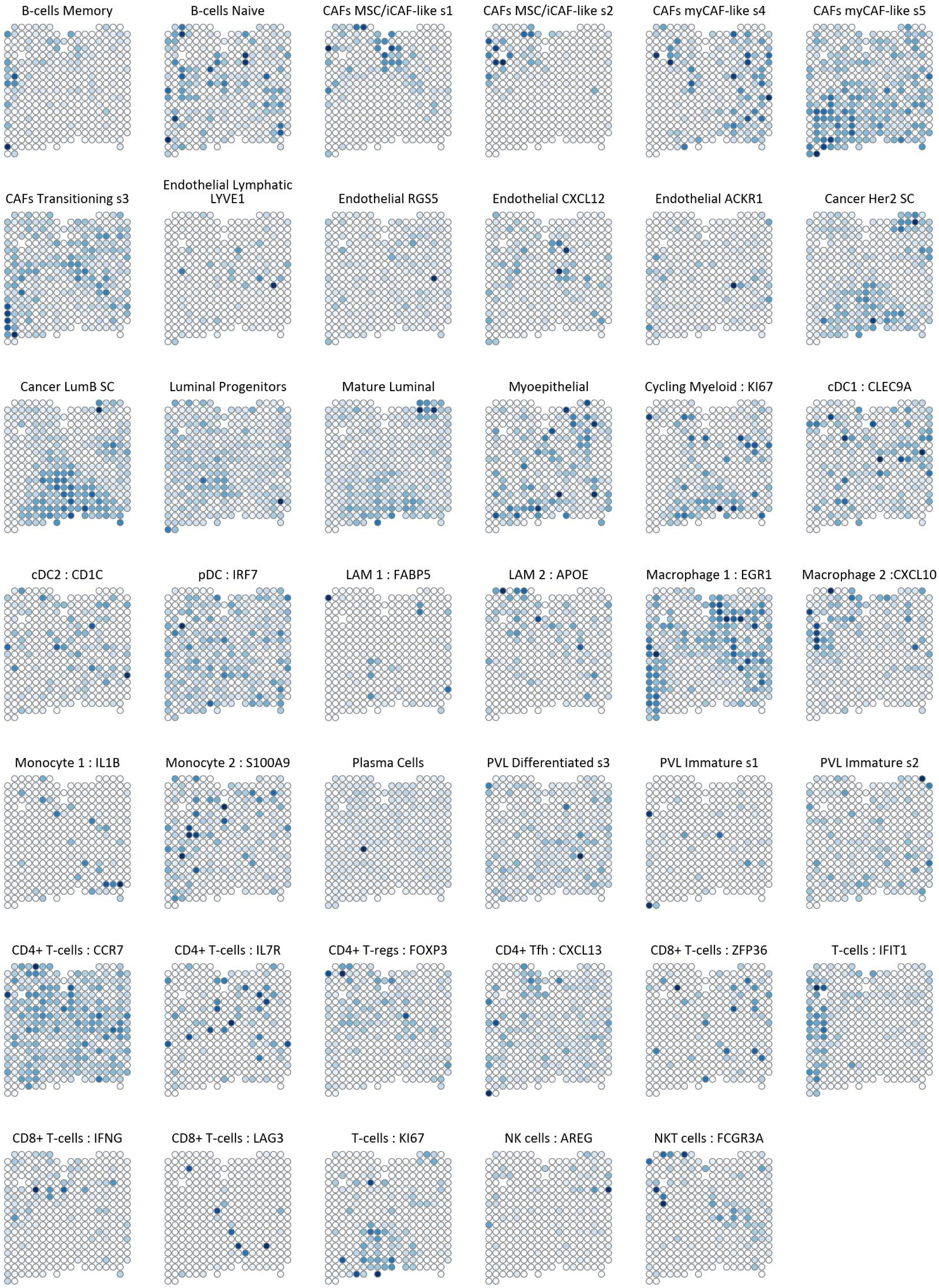

B-cells Memory
B-cells Naive
CAFs MSC/iCAF-like s1
CAFs MSC/iCAF-like s2
CAFs myCAF-like s4
CAFs myCAF-like s5
CAFs Transitioning s3
Endothelial Lymphatic LYVE1
Endothelial RGS5
Endothelial CXCL12
Endothelial ACKR1
Cancer Her2 SC
Cancer LumB SC
Luminal Progenitors
Mature Luminal
Myoepithelial
Cycling Myeloid : KI67
cDC1 : CLEC9A
cDC2 : CD1C
pDC : IRF7
LAM 1 : FABP5
LAM 2 : APOE
Macrophage 1 : EGR1
Macrophage 2 :CXCL10
Monocyte 1 : IL1B
Monocyte 2 : S100A9
Plasma Cells
PVL Differentiated s3
PVL Immature s1
PVL Immature s2
CD4+ T-cells : CCR7
CD4+ T-cells : IL7R
CD4+ T-regs : FOXP3
CD4+ Tfh : CXCL13
CD8+ T-cells : ZFP36
T-cells : IFIT1
CD8+ T-cells : IFNG
CD8+ T-cells : LAG3
T-cells : KI67
NK cells : AREG
NKT cells : FCGR3A

subset_H1

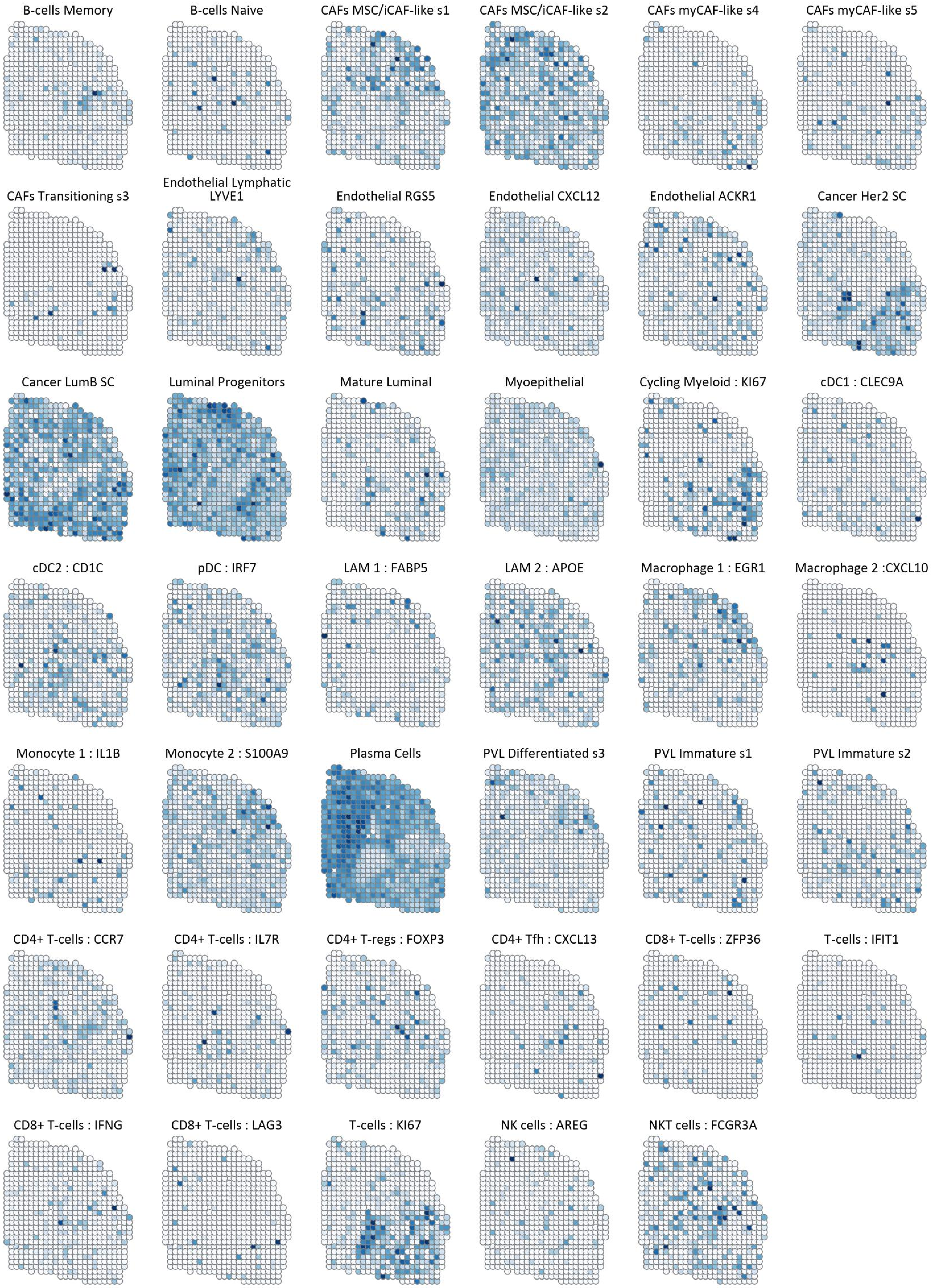
B-cells Memory
B-cells Naive
CAFs MSC/iCAF-like s1
CAFs MSC/iCAF-like s2
CAFs myCAF-like s4
CAFs myCAF-like s5
CAFs Transitioning s3
Endothelial Lymphatic LYVE1
Endothelial RGS5
Endothelial CXCL12
Endothelial ACKR1
Cancer Her2 SC
Cancer LumB SC
Luminal Progenitors
Mature Luminal
Myoepithelial
Cycling Myeloid : KI67
cDC1 : CLEC9A
cDC2 : CD1C
pDC : IRF7
LAM 1 : FABP5
LAM 2 : APOE
Macrophage 1 : EGR1
Macrophage 2 :CXCL10
Monocyte 1 : IL1B
Monocyte 2 : S100A9
Plasma Cells
PVL Differentiated s3
PVL Immature s1
PVL Immature s2
CD4+ T-cells : CCR7
CD4+ T-cells : IL7R
CD4+ T-regs : FOXP3
CD4+ Tfh : CXCL13
CD8+ T-cells : ZFP36
T-cells : IFIT1
CD8+ T-cells : IFNG
CD8+ T-cells : LAG3
T-cells : KI67
NK cells : AREG
NKT cells : FCGR3A

subset_F1

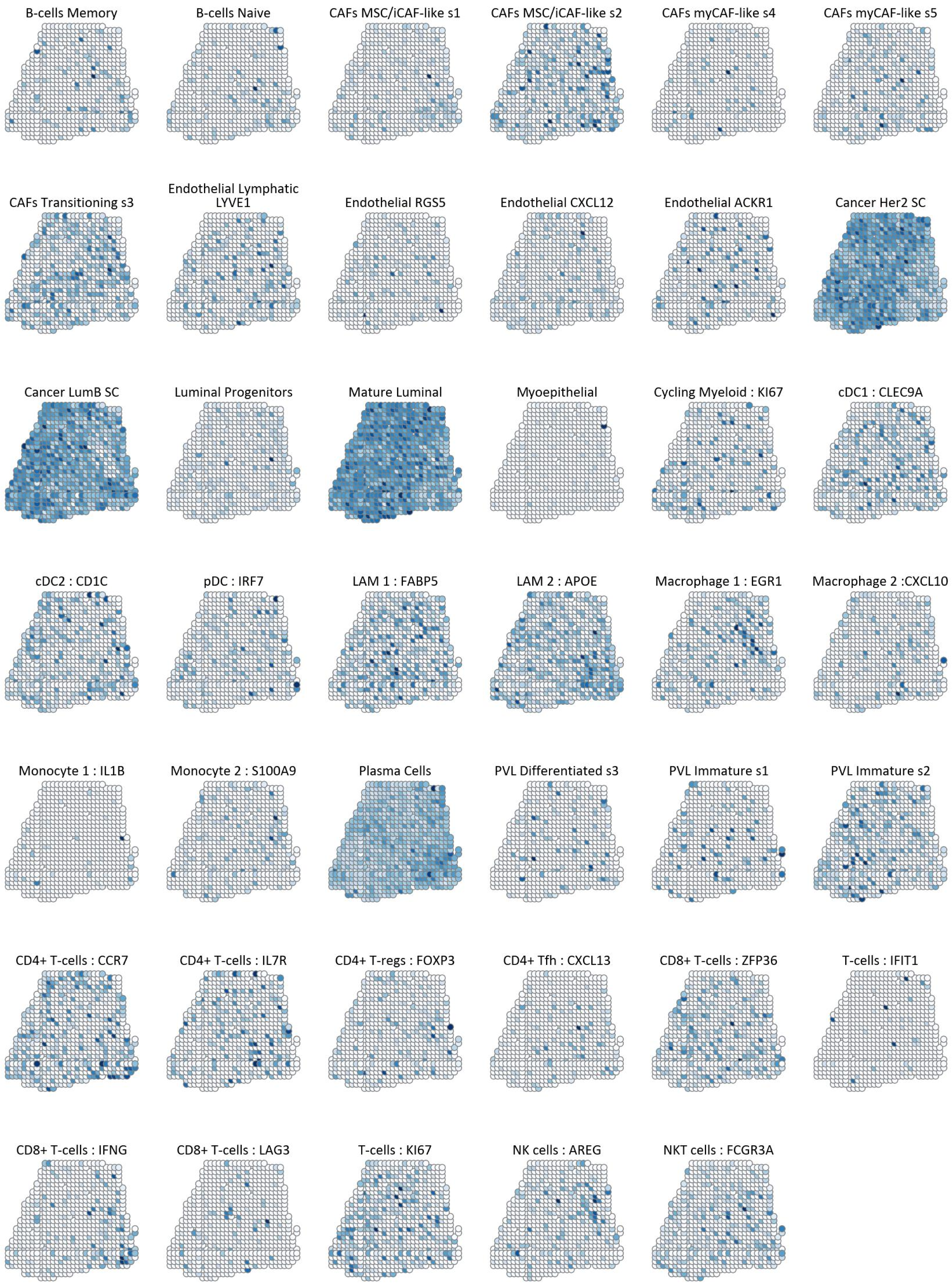
B-cells Memory
B-cells Naive
CAFs MSC/iCAF-like s1
CAFs MSC/iCAF-like s2
CAFs myCAF-like s4
CAFs myCAF-like s5
CAFs Transitioning s3
Endothelial Lymphatic LYVE1
Endothelial RGS5
Endothelial CXCL12
Endothelial ACKR1
Cancer Her2 SC
Cancer LumB SC
Luminal Progenitors
Mature Luminal
Myoepithelial
Cycling Myeloid : KI67
cDC1 : CLEC9A
cDC2 : CD1C
pDC : IRF7
LAM 1 : FABP5
LAM 2 : APOE
Macrophage 1 : EGR1
Macrophage 2 :CXCL10
Monocyte 1 : IL1B
Monocyte 2 : S100A9
Plasma Cells
PVL Differentiated s3
PVL Immature s1
PVL Immature s2
CD4+ T-cells : CCR7
CD4+ T-cells : IL7R
CD4+ T-regs : FOXP3
CD4+ Tfh : CXCL13
CD8+ T-cells : ZFP36
T-cells : IFIT1
CD8+ T-cells : IFNG
CD8+ T-cells : LAG3
T-cells : KI67
NK cells : AREG
NKT cells : FCGR3A

subset_B1

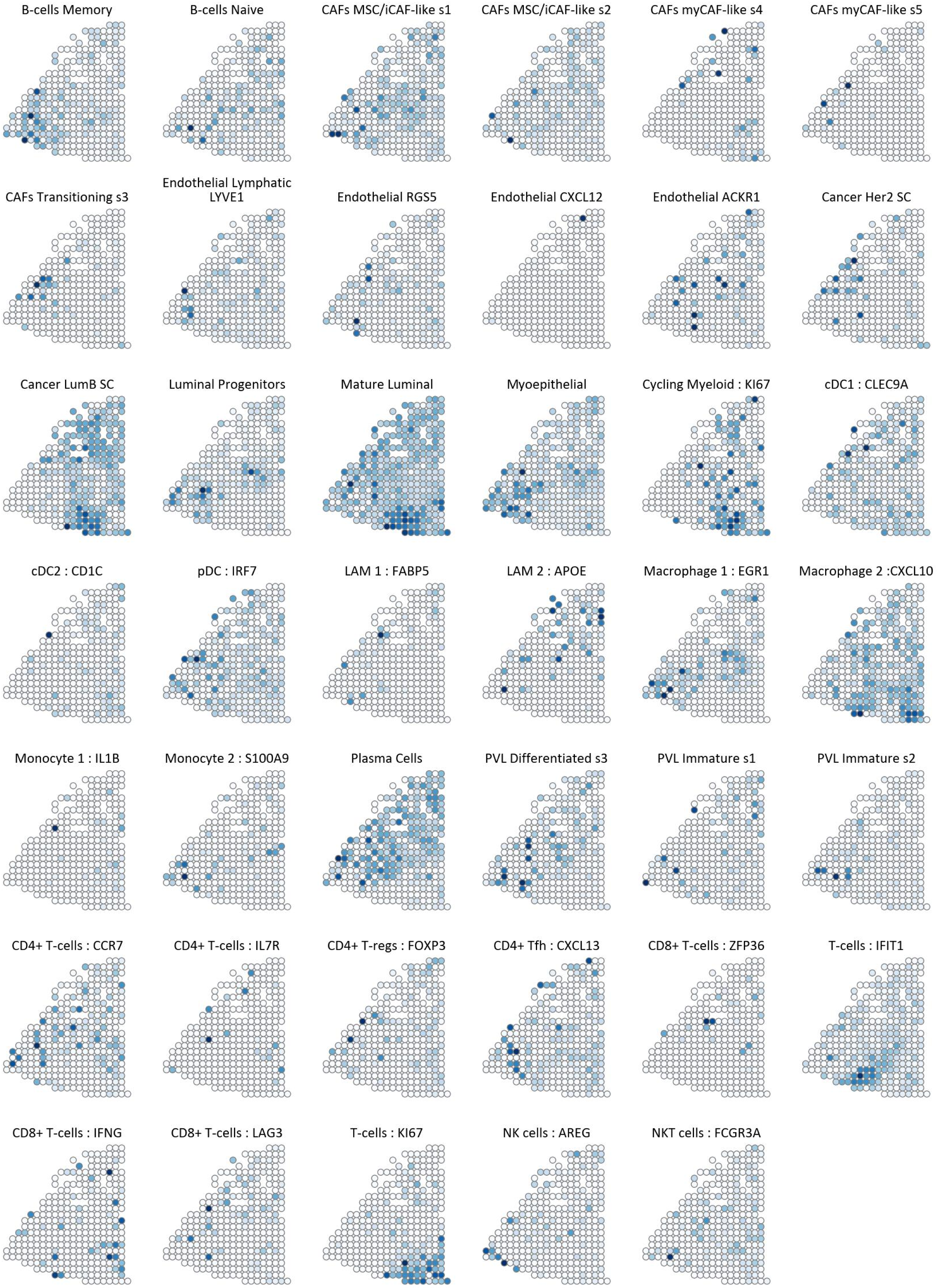

B-cells Memory
B-cells Naive
CAFs MSC/iCAF-like s1
CAFs MSC/iCAF-like s2
CAFs myCAF-like s4
CAFs myCAF-like s5
CAFs Transitioning s3
Endothelial Lymphatic LYVE1
Endothelial RGS5
Endothelial CXCL12
Endothelial ACKR1
Cancer Her2 SC
Cancer LumB SC
Luminal Progenitors
Mature Luminal
Myoepithelial
Cycling Myeloid : KI67
cDC1 : CLEC9A
cDC2 : CD1C
pDC : IRF7
LAM 1 : FABP5
LAM 2 : APOE
Macrophage 1 : EGR1
Macrophage 2 :CXCL10
Monocyte 1 : IL1B
Monocyte 2 : S100A9
Plasma Cells
PVL Differentiated s3
PVL Immature s1
PVL Immature s2
CD4+ T-cells : CCR7
CD4+ T-cells : IL7R
CD4+ T-regs : FOXP3
CD4+ Tfh : CXCL13
CD8+ T-cells : ZFP36
T-cells : IFIT1
CD8+ T-cells : IFNG
CD8+ T-cells : LAG3
T-cells : KI67
NK cells : AREG
NKT cells : FCGR3A

subset_G1

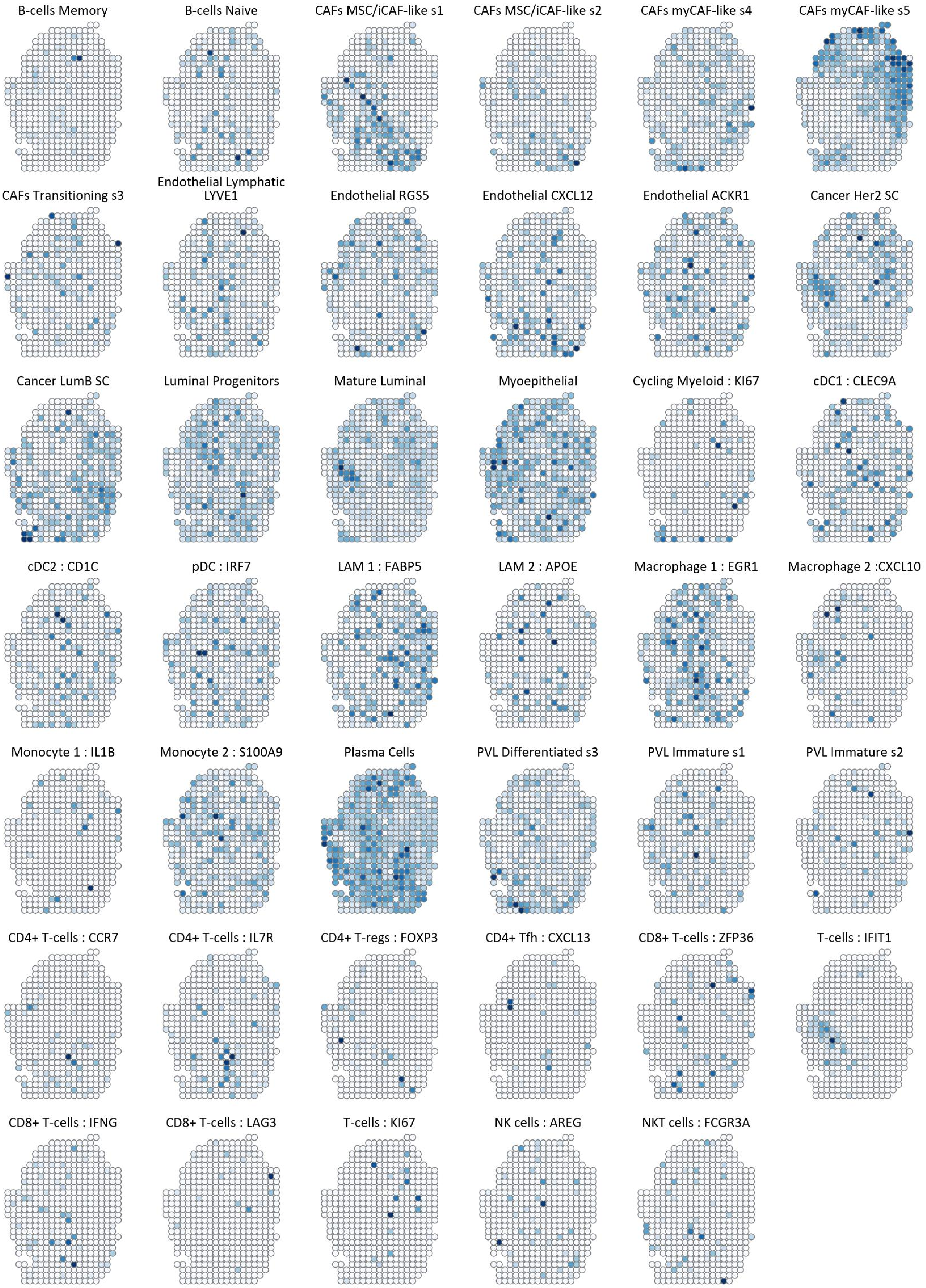
B-cells Memory
B-cells Naive
CAFs MSC/iCAF-like s1
CAFs MSC/iCAF-like s2
CAFs myCAF-like s4
CAFs myCAF-like s5
CAFs Transitioning s3
Endothelial Lymphatic LYVE1
Endothelial RGS5
Endothelial CXCL12
Endothelial ACKR1
Cancer Her2 SC
Cancer LumB SC
Luminal Progenitors
Mature Luminal
Myoepithelial
Cycling Myeloid : KI67
cDC1 : CLEC9A
cDC2 : CD1C
pDC : IRF7
LAM 1 : FABP5
LAM 2 : APOE
Macrophage 1 : EGR1
Macrophage 2 :CXCL10
Monocyte 1 : IL1B
Monocyte 2 : S100A9
Plasma Cells
PVL Differentiated s3
PVL Immature s1
PVL Immature s2
CD4+ T-cells : CCR7
CD4+ T-cells : IL7R
CD4+ T-regs : FOXP3
CD4+ Tfh : CXCL13
CD8+ T-cells : ZFP36
T-cells : IFIT1
CD8+ T-cells : IFNG
CD8+ T-cells : LAG3
T-cells : KI67
NK cells : AREG
NKT cells : FCGR3A

subset_D3

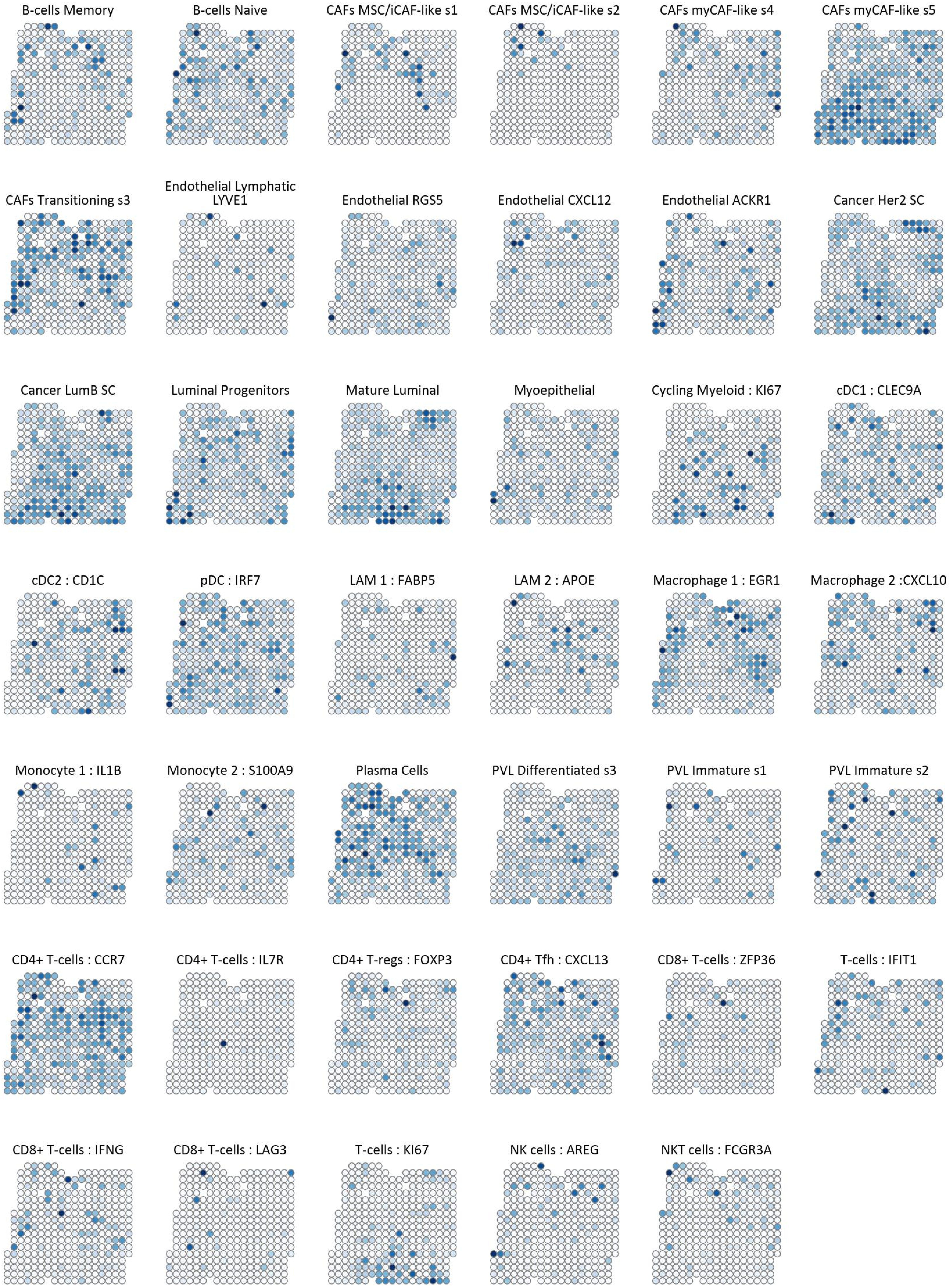
B-cells Memory
B-cells Naive
CAFs MSC/iCAF-like s1
CAFs MSC/iCAF-like s2
CAFs myCAF-like s4
CAFs myCAF-like s5
CAFs Transitioning s3
Endothelial Lymphatic LYVE1
Endothelial RGS5
Endothelial CXCL12
Endothelial ACKR1
Cancer Her2 SC
Cancer LumB SC
Luminal Progenitors
Mature Luminal
Myoepithelial
Cycling Myeloid : KI67
cDC1 : CLEC9A
cDC2 : CD1C
pDC : IRF7
LAM 1 : FABP5
LAM 2 : APOE
Macrophage 1 : EGR1
Macrophage 2 :CXCL10
Monocyte 1 : IL1B
Monocyte 2 : S100A9
Plasma Cells
PVL Differentiated s3
PVL Immature s1
PVL Immature s2
CD4+ T-cells : CCR7
CD4+ T-cells : IL7R
CD4+ T-regs : FOXP3
CD4+ Tfh : CXCL13
CD8+ T-cells : ZFP36
T-cells : IFIT1
CD8+ T-cells : IFNG
CD8+ T-cells : LAG3
T-cells : KI67
NK cells : AREG
NKT cells : FCGR3A

subset_C6

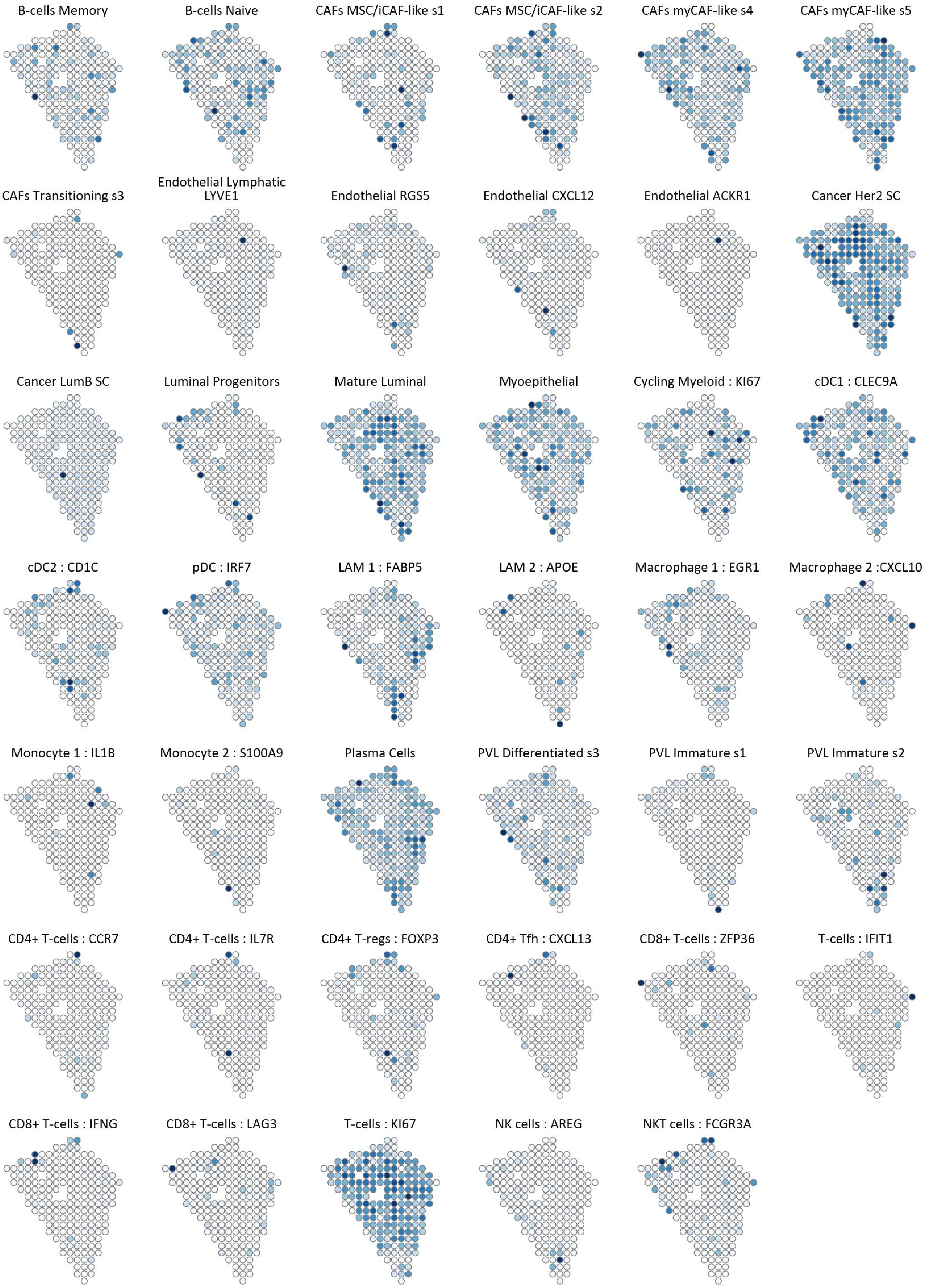
B-cells Memory
B-cells Naive
CAFs MSC/iCAF-like s1
CAFs MSC/iCAF-like s2
CAFs myCAF-like s4
CAFs myCAF-like s5
CAFs Transitioning s3
Endothelial Lymphatic LYVE1
Endothelial RGS5
Endothelial CXCL12
Endothelial ACKR1
Cancer Her2 SC
Cancer LumB SC
Luminal Progenitors
Mature Luminal
Myoepithelial
Cycling Myeloid : KI67
cDC1 : CLEC9A
cDC2 : CD1C
pDC : IRF7
LAM 1 : FABP5
LAM 2 : APOE
Macrophage 1 : EGR1
Macrophage 2 :CXCL10
Monocyte 1 : IL1B
Monocyte 2 : S100A9
Plasma Cells
PVL Differentiated s3
PVL Immature s1
PVL Immature s2
CD4+ T-cells : CCR7
CD4+ T-cells : IL7R
CD4+ T-regs : FOXP3
CD4+ Tfh : CXCL13
CD8+ T-cells : ZFP36
T-cells : IFIT1
CD8+ T-cells : IFNG
CD8+ T-cells : LAG3
T-cells : KI67
NK cells : AREG
NKT cells : FCGR3A

subset_B5

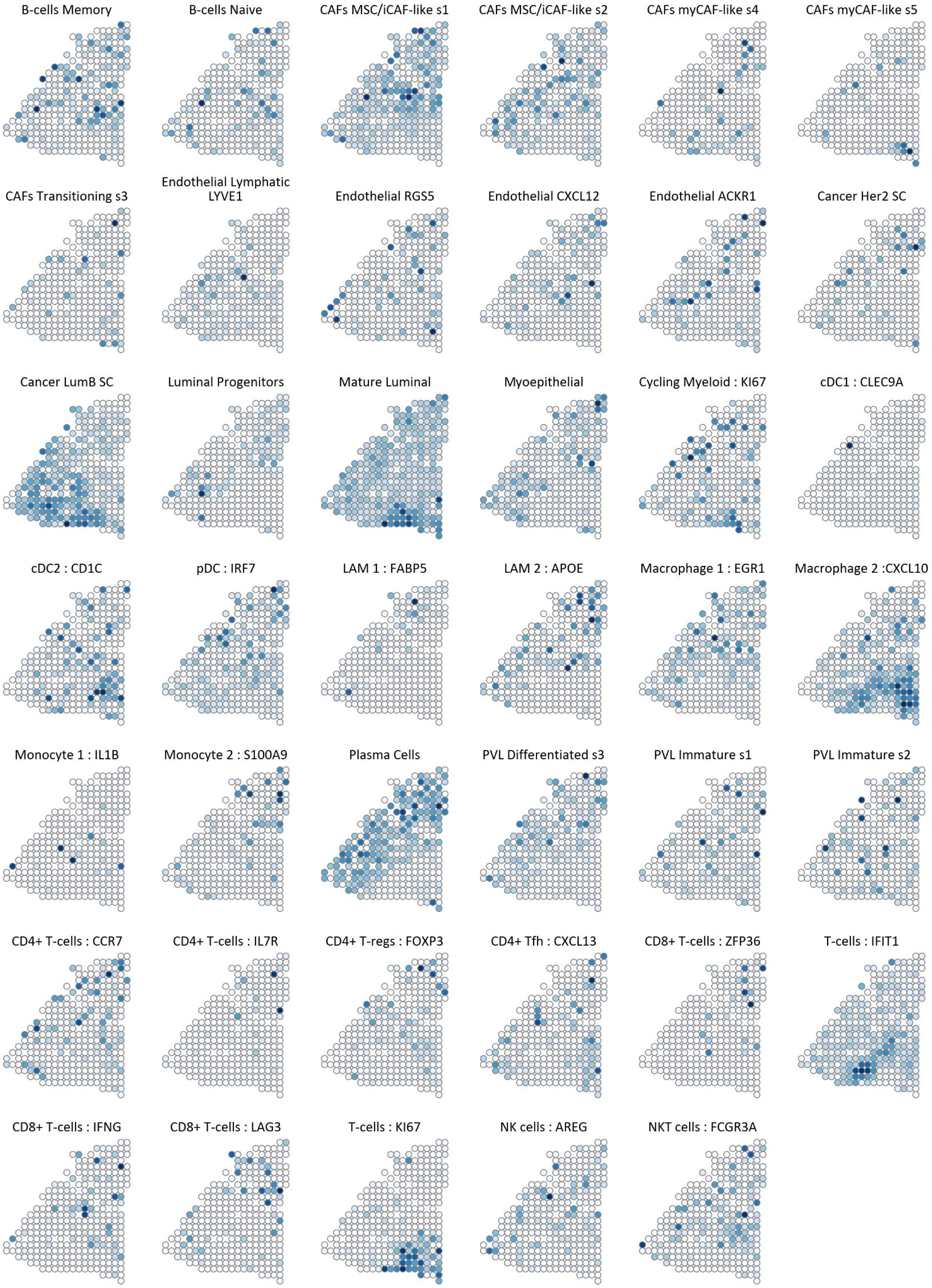

B-cells Memory
B-cells Naive
CAFs MSC/iCAF-like s1
CAFs MSC/iCAF-like s2
CAFs myCAF-like s4
CAFs myCAF-like s5
CAFs Transitioning s3
Endothelial Lymphatic LYVE1
Endothelial RGS5
Endothelial CXCL12
Endothelial ACKR1
Cancer Her2 SC
Cancer LumB SC
Luminal Progenitors
Mature Luminal
Myoepithelial
Cycling Myeloid : KI67
cDC1 : CLEC9A
cDC2 : CD1C
pDC : IRF7
LAM 1 : FABP5
LAM 2 : APOE
Macrophage 1 : EGR1
Macrophage 2 :CXCL10
Monocyte 1 : IL1B
Monocyte 2 : S100A9
Plasma Cells
PVL Differentiated s3
PVL Immature s1
PVL Immature s2
CD4+ T-cells : CCR7
CD4+ T-cells : IL7R
CD4+ T-regs : FOXP3
CD4+ Tfh : CXCL13
CD8+ T-cells : ZFP36
T-cells : IFIT1
CD8+ T-cells : IFNG
CD8+ T-cells : LAG3
T-cells : KI67
NK cells : AREG
NKT cells : FCGR3A

subset_E3

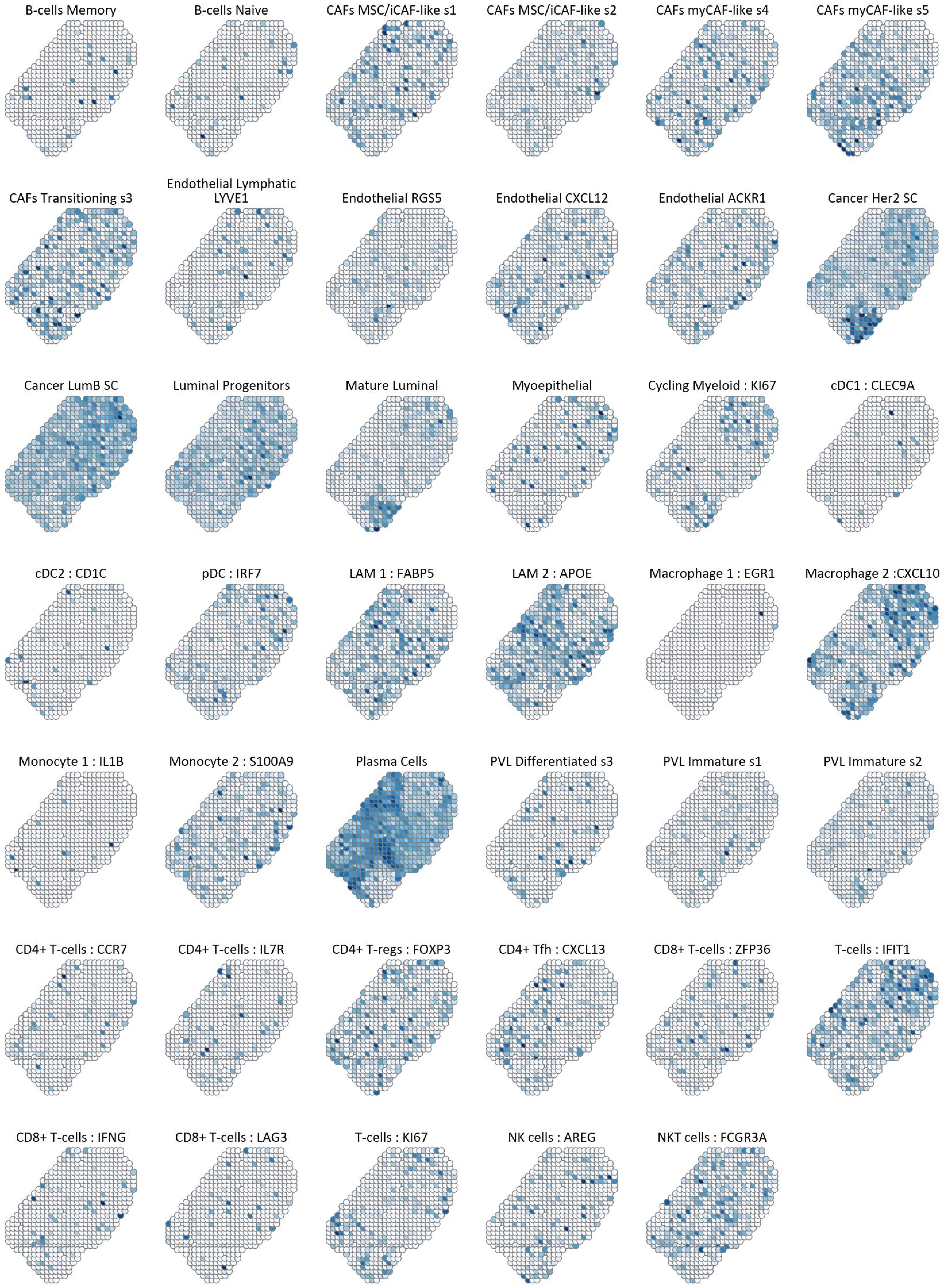

B-cells Memory
B-cells Naive
CAFs MSC/iCAF-like s1
CAFs MSC/iCAF-like s2
CAFs myCAF-like s4
CAFs myCAF-like s5
CAFs Transitioning s3
Endothelial Lymphatic LYVE1
Endothelial RGS5
Endothelial CXCL12
Endothelial ACKR1
Cancer Her2 SC
Cancer LumB SC
Luminal Progenitors
Mature Luminal
Myoepithelial
Cycling Myeloid : KI67
cDC1 : CLEC9A
cDC2 : CD1C
pDC : IRF7
LAM 1 : FABP5
LAM 2 : APOE
Macrophage 1 : EGR1
Macrophage 2 :CXCL10
Monocyte 1 : IL1B
Monocyte 2 : S100A9
Plasma Cells
PVL Differentiated s3
PVL Immature s1
PVL Immature s2
CD4+ T-cells : CCR7
CD4+ T-cells : IL7R
CD4+ T-regs : FOXP3
CD4+ Tfh : CXCL13
CD8+ T-cells : ZFP36
T-cells : IFIT1
CD8+ T-cells : IFNG
CD8+ T-cells : LAG3
T-cells : KI67
NK cells : AREG
NKT cells : FCGR3A

subset_H3

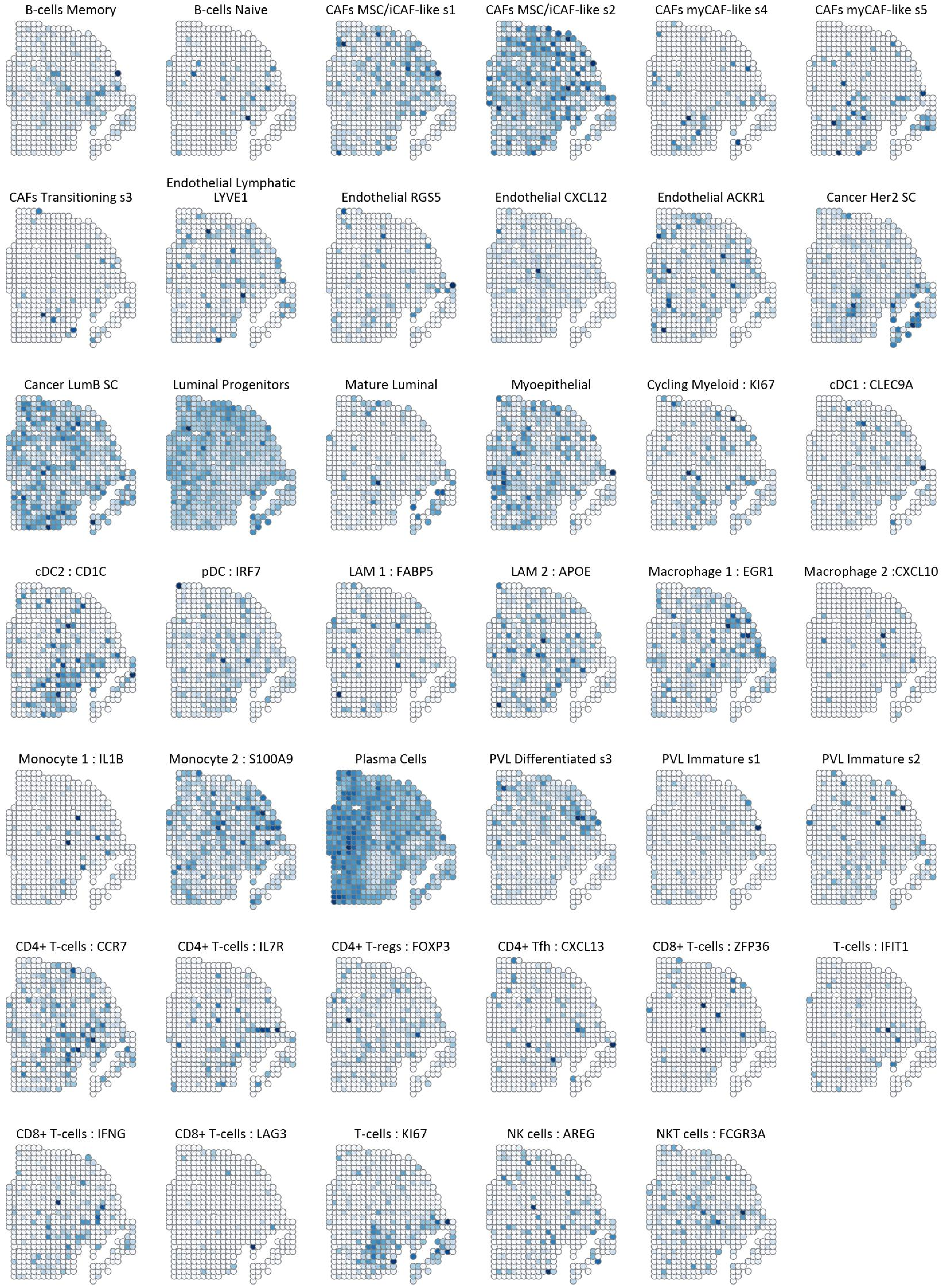
B-cells Memory
B-cells Naive
CAFs MSC/iCAF-like s1
CAFs MSC/iCAF-like s2
CAFs myCAF-like s4
CAFs myCAF-like s5
CAFs Transitioning s3
Endothelial Lymphatic LYVE1
Endothelial RGS5
Endothelial CXCL12
Endothelial ACKR1
Cancer Her2 SC
Cancer LumB SC
Luminal Progenitors
Mature Luminal
Myoepithelial
Cycling Myeloid : KI67
cDC1 : CLEC9A
cDC2 : CD1C
pDC : IRF7
LAM 1 : FABP5
LAM 2 : APOE
Macrophage 1 : EGR1
Macrophage 2 :CXCL10
Monocyte 1 : IL1B
Monocyte 2 : S100A9
Plasma Cells
PVL Differentiated s3
PVL Immature s1
PVL Immature s2
CD4+ T-cells : CCR7
CD4+ T-cells : IL7R
CD4+ T-regs : FOXP3
CD4+ Tfh : CXCL13
CD8+ T-cells : ZFP36
T-cells : IFIT1
CD8+ T-cells : IFNG
CD8+ T-cells : LAG3
T-cells : KI67
NK cells : AREG
NKT cells : FCGR3A

subset_D4

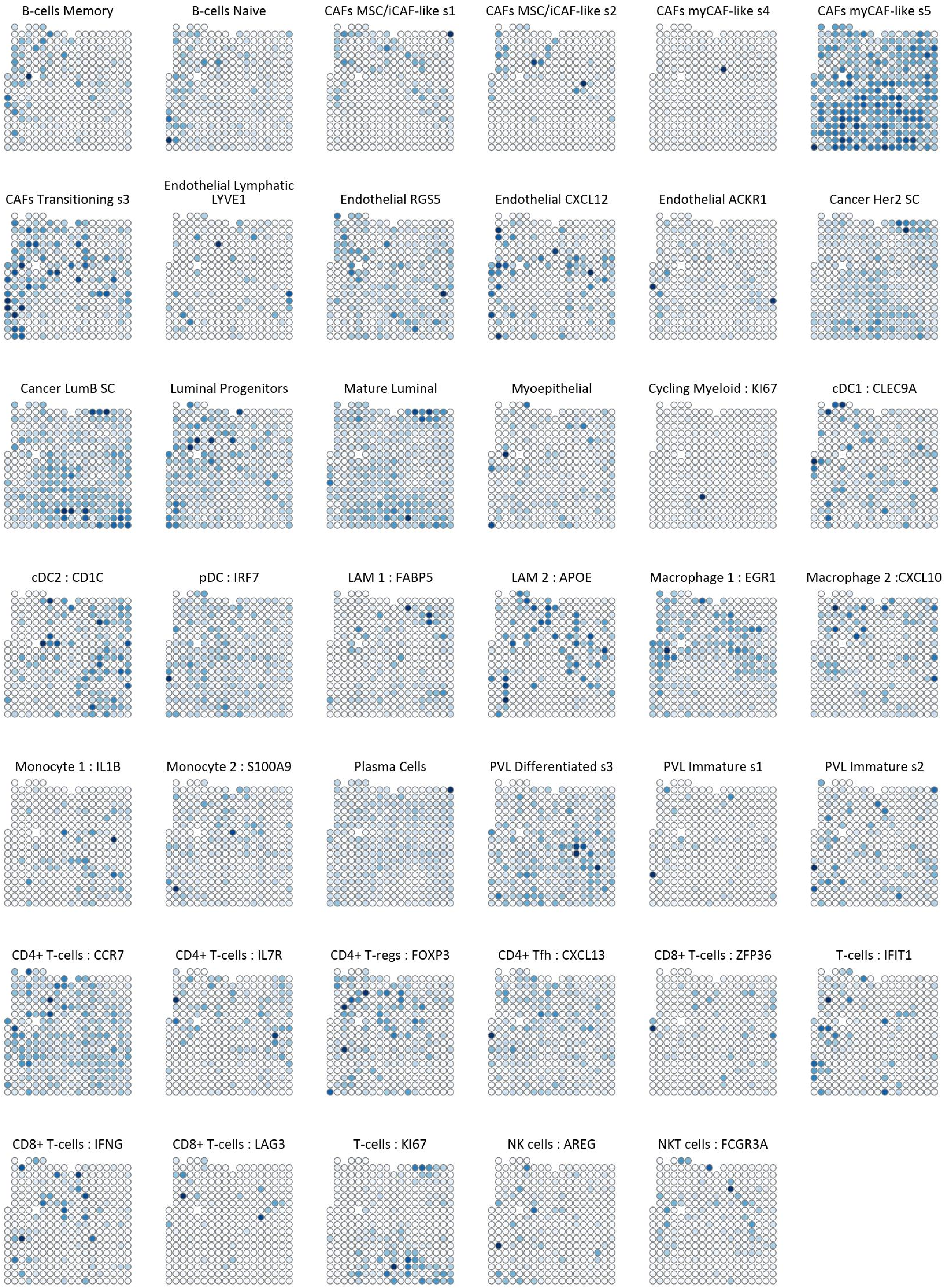
B-cells Memory
B-cells Naive
CAFs MSC/iCAF-like s1
CAFs MSC/iCAF-like s2
CAFs myCAF-like s4
CAFs myCAF-like s5
CAFs Transitioning s3
Endothelial Lymphatic LYVE1
Endothelial RGS5
Endothelial CXCL12
Endothelial ACKR1
Cancer Her2 SC
Cancer LumB SC
Luminal Progenitors
Mature Luminal
Myoepithelial
Cycling Myeloid : KI67
cDC1 : CLEC9A
cDC2 : CD1C
pDC : IRF7
LAM 1 : FABP5
LAM 2 : APOE
Macrophage 1 : EGR1
Macrophage 2 :CXCL10
Monocyte 1 : IL1B
Monocyte 2 : S100A9
Plasma Cells
PVL Differentiated s3
PVL Immature s1
PVL Immature s2
CD4+ T-cells : CCR7
CD4+ T-cells : IL7R
CD4+ T-regs : FOXP3
CD4+ Tfh : CXCL13
CD8+ T-cells : ZFP36
T-cells : IFIT1
CD8+ T-cells : IFNG
CD8+ T-cells : LAG3
T-cells : KI67
NK cells : AREG
NKT cells : FCGR3A

subset_B2

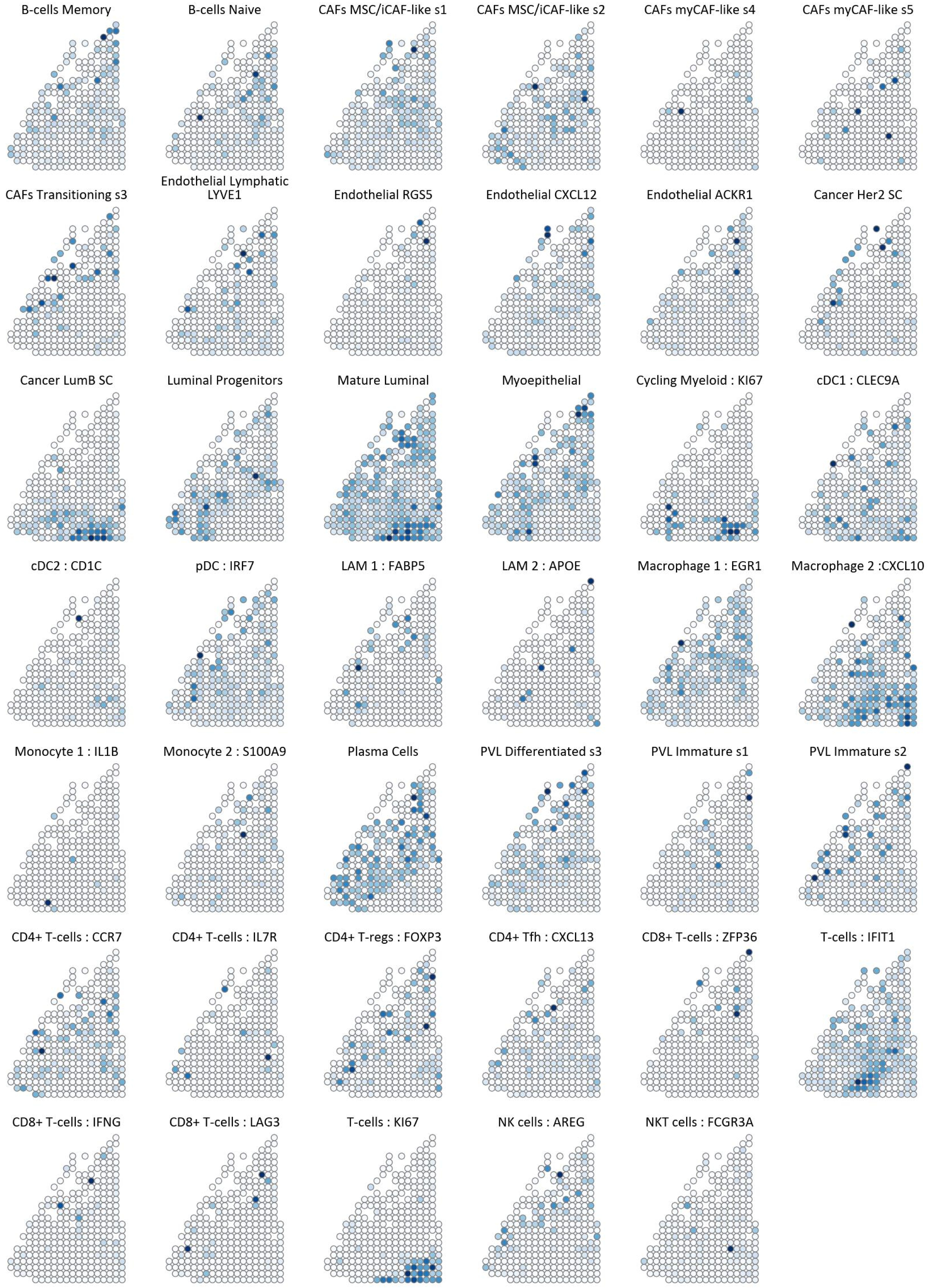

B-cells Memory
B-cells Naive
CAFs MSC/iCAF-like s1
CAFs MSC/iCAF-like s2
CAFs myCAF-like s4
CAFs myCAF-like s5
CAFs Transitioning s3
Endothelial Lymphatic LYVE1
Endothelial RGS5
Endothelial CXCL12
Endothelial ACKR1
Cancer Her2 SC
Cancer LumB SC
Luminal Progenitors
Mature Luminal
Myoepithelial
Cycling Myeloid : KI67
cDC1 : CLEC9A
cDC2 : CD1C
pDC : IRF7
LAM 1 : FABP5
LAM 2 : APOE
Macrophage 1 : EGR1
Macrophage 2 :CXCL10
Monocyte 1 : IL1B
Monocyte 2 : S100A9
Plasma Cells
PVL Differentiated s3
PVL Immature s1
PVL Immature s2
CD4+ T-cells : CCR7
CD4+ T-cells : IL7R
CD4+ T-regs : FOXP3
CD4+ Tfh : CXCL13
CD8+ T-cells : ZFP36
T-cells : IFIT1
CD8+ T-cells : IFNG
CD8+ T-cells : LAG3
T-cells : KI67
NK cells : AREG
NKT cells : FCGR3A

subset_A6

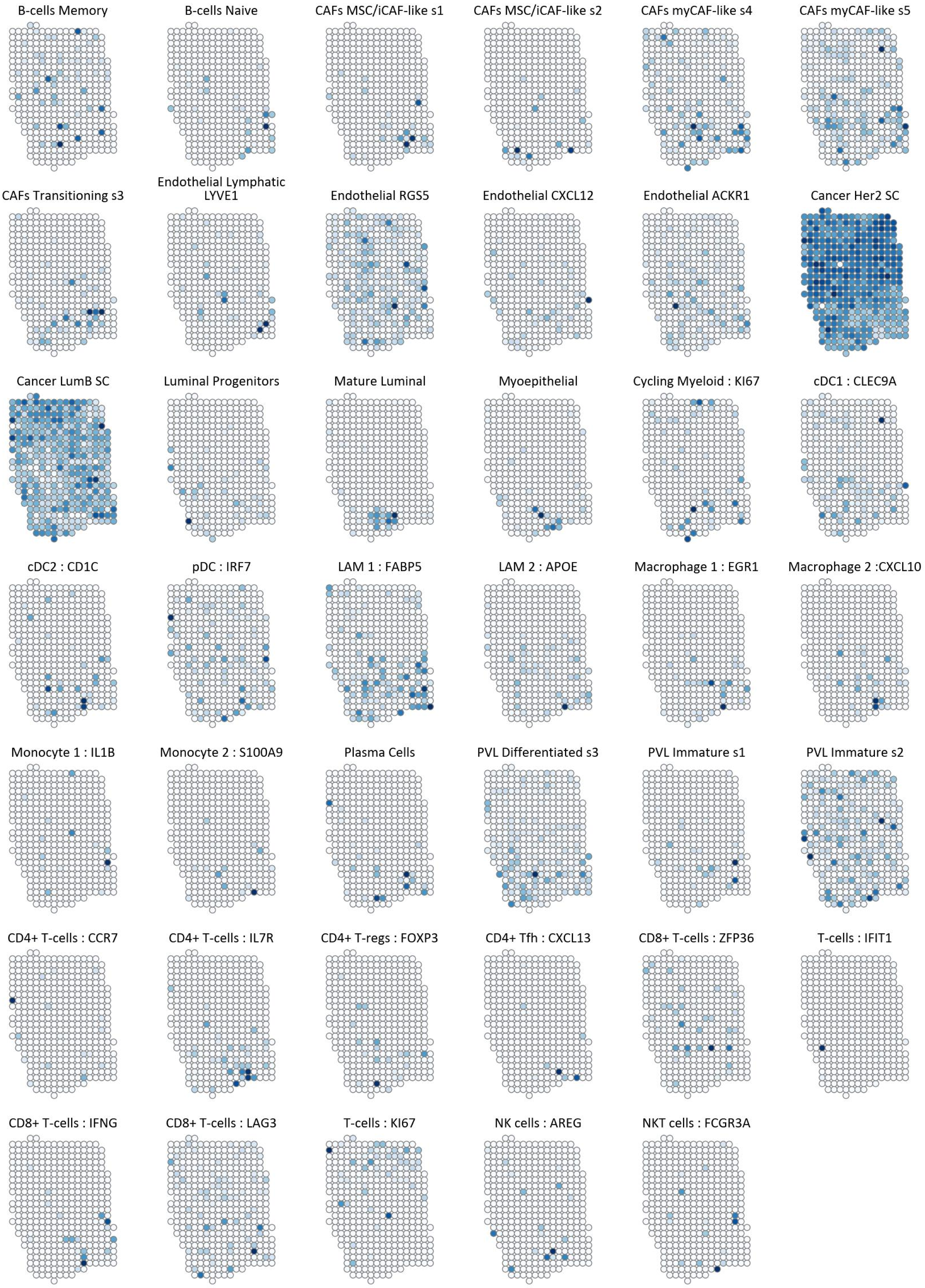

B-cells Memory
B-cells Naive
CAFs MSC/iCAF-like s1
CAFs MSC/iCAF-like s2
CAFs myCAF-like s4
CAFs myCAF-like s5
CAFs Transitioning s3
Endothelial Lymphatic LYVE1
Endothelial RGS5
Endothelial CXCL12
Endothelial ACKR1
Cancer Her2 SC
Cancer LumB SC
Luminal Progenitors
Mature Luminal
Myoepithelial
Cycling Myeloid : KI67
cDC1 : CLEC9A
cDC2 : CD1C
pDC : IRF7
LAM 1 : FABP5
LAM 2 : APOE
Macrophage 1 : EGR1
Macrophage 2 :CXCL10
Monocyte 1 : IL1B
Monocyte 2 : S100A9
Plasma Cells
PVL Differentiated s3
PVL Immature s1
PVL Immature s2
CD4+ T-cells : CCR7
CD4+ T-cells : IL7R
CD4+ T-regs : FOXP3
CD4+ Tfh : CXCL13
CD8+ T-cells : ZFP36
T-cells : IFIT1
CD8+ T-cells : IFNG
CD8+ T-cells : LAG3
T-cells : KI67
NK cells : AREG
NKT cells : FCGR3A

subset_F3

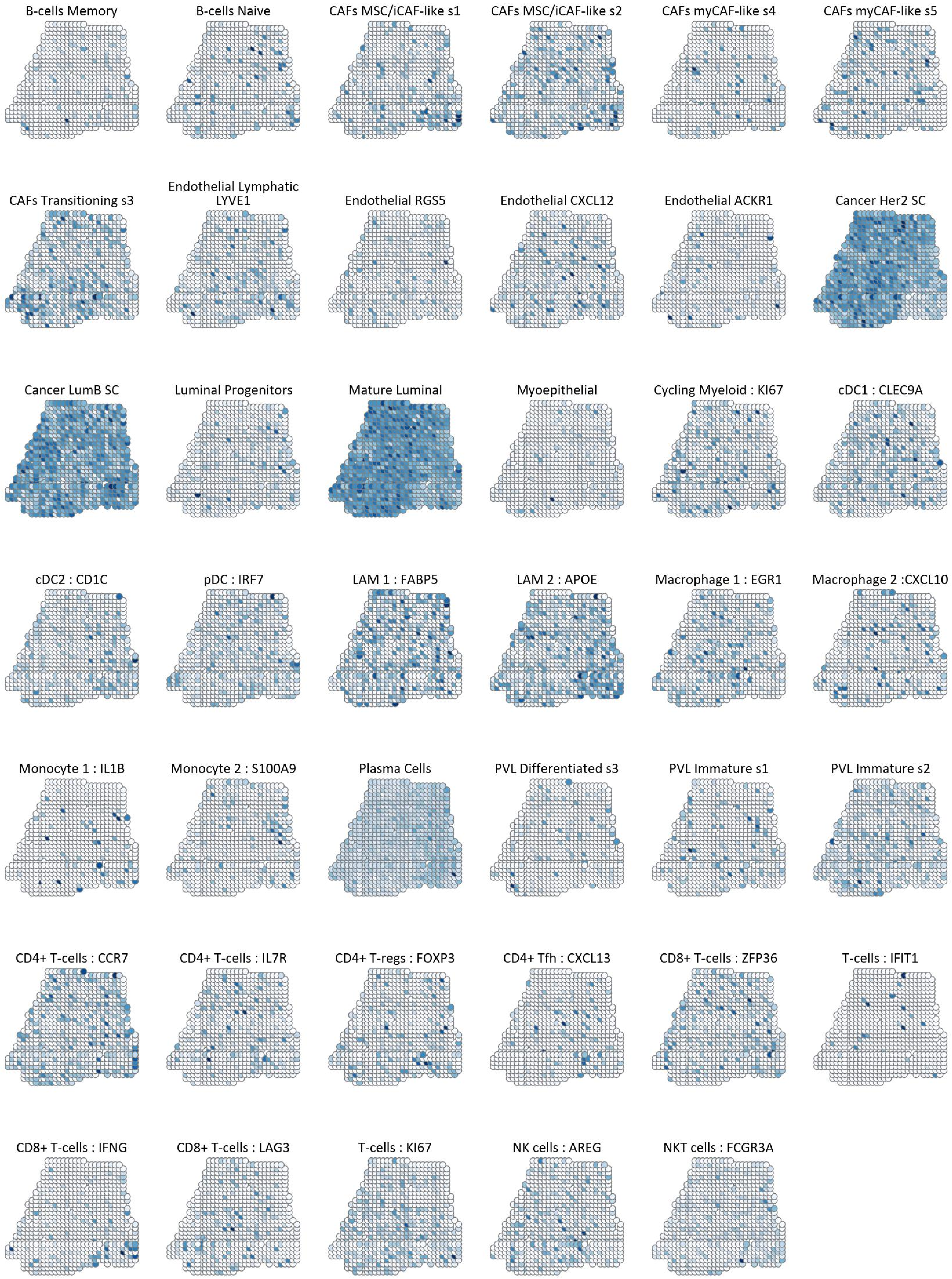
B-cells Memory
B-cells Naive
CAFs MSC/iCAF-like s1
CAFs MSC/iCAF-like s2
CAFs myCAF-like s4
CAFs myCAF-like s5
CAFs Transitioning s3
Endothelial Lymphatic LYVE1
Endothelial RGS5
Endothelial CXCL12
Endothelial ACKR1
Cancer Her2 SC
Cancer LumB SC
Luminal Progenitors
Mature Luminal
Myoepithelial
Cycling Myeloid : KI67
cDC1 : CLEC9A
cDC2 : CD1C
pDC : IRF7
LAM 1 : FABP5
LAM 2 : APOE
Macrophage 1 : EGR1
Macrophage 2 :CXCL10
Monocyte 1 : IL1B
Monocyte 2 : S100A9
Plasma Cells
PVL Differentiated s3
PVL Immature s1
PVL Immature s2
CD4+ T-cells : CCR7
CD4+ T-cells : IL7R
CD4+ T-regs : FOXP3
CD4+ Tfh : CXCL13
CD8+ T-cells : ZFP36
T-cells : IFIT1
CD8+ T-cells : IFNG
CD8+ T-cells : LAG3
T-cells : KI67
NK cells : AREG
NKT cells : FCGR3A

subset_A3

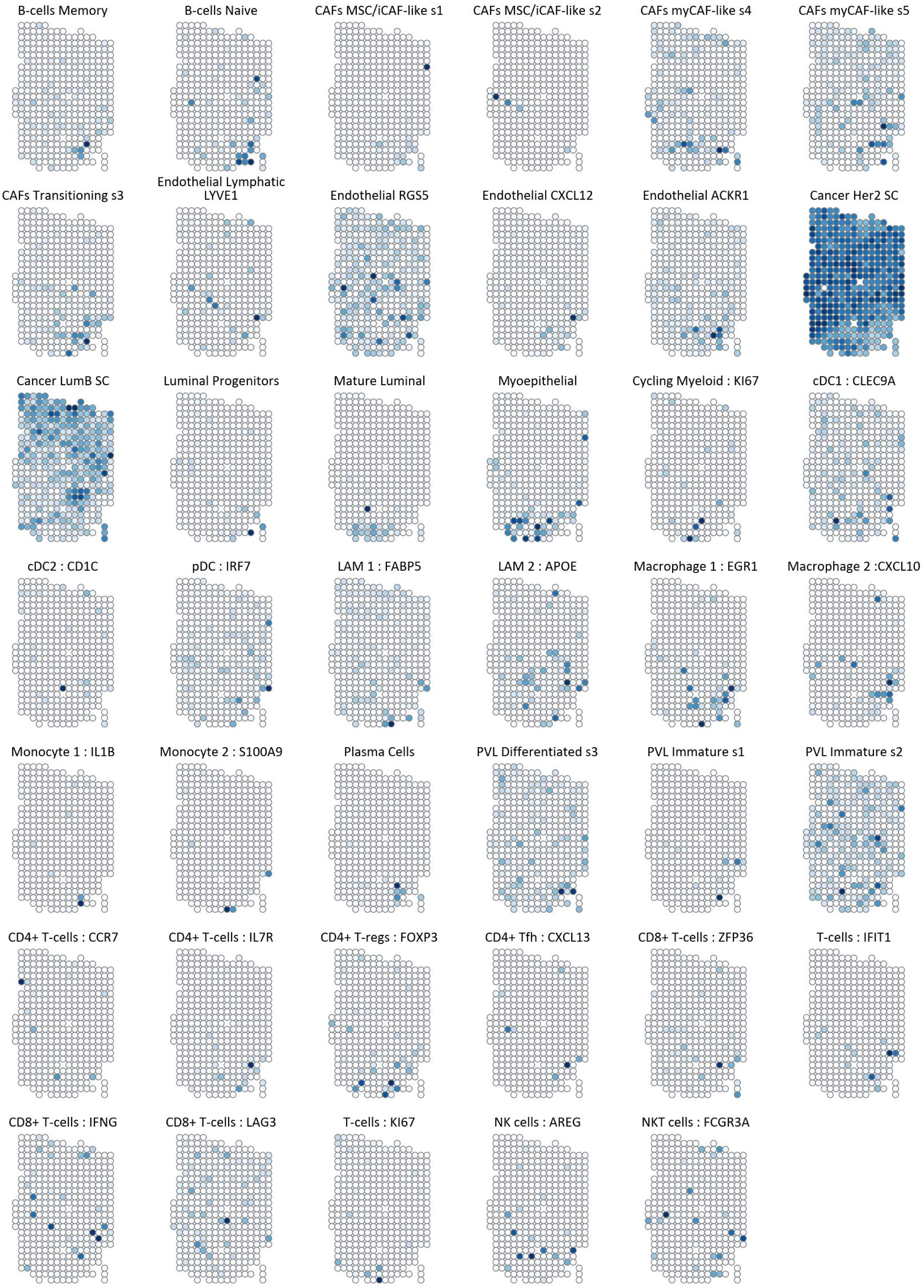
B-cells Memory
B-cells Naive
CAFs MSC/iCAF-like s1
CAFs MSC/iCAF-like s2
CAFs myCAF-like s4
CAFs myCAF-like s5
CAFs Transitioning s3
Endothelial Lymphatic LYVE1
Endothelial RGS5
Endothelial CXCL12
Endothelial ACKR1
Cancer Her2 SC
Cancer LumB SC
Luminal Progenitors
Mature Luminal
Myoepithelial
Cycling Myeloid : KI67
cDC1 : CLEC9A
cDC2 : CD1C
pDC : IRF7
LAM 1 : FABP5
LAM 2 : APOE
Macrophage 1 : EGR1
Macrophage 2 :CXCL10
Monocyte 1 : IL1B
Monocyte 2 : S100A9
Plasma Cells
PVL Differentiated s3
PVL Immature s1
PVL Immature s2
CD4+ T-cells : CCR7
CD4+ T-cells : IL7R
CD4+ T-regs : FOXP3
CD4+ Tfh : CXCL13
CD8+ T-cells : ZFP36
T-cells : IFIT1
CD8+ T-cells : IFNG
CD8+ T-cells : LAG3
T-cells : KI67
NK cells : AREG
NKT cells : FCGR3A

subset_D5

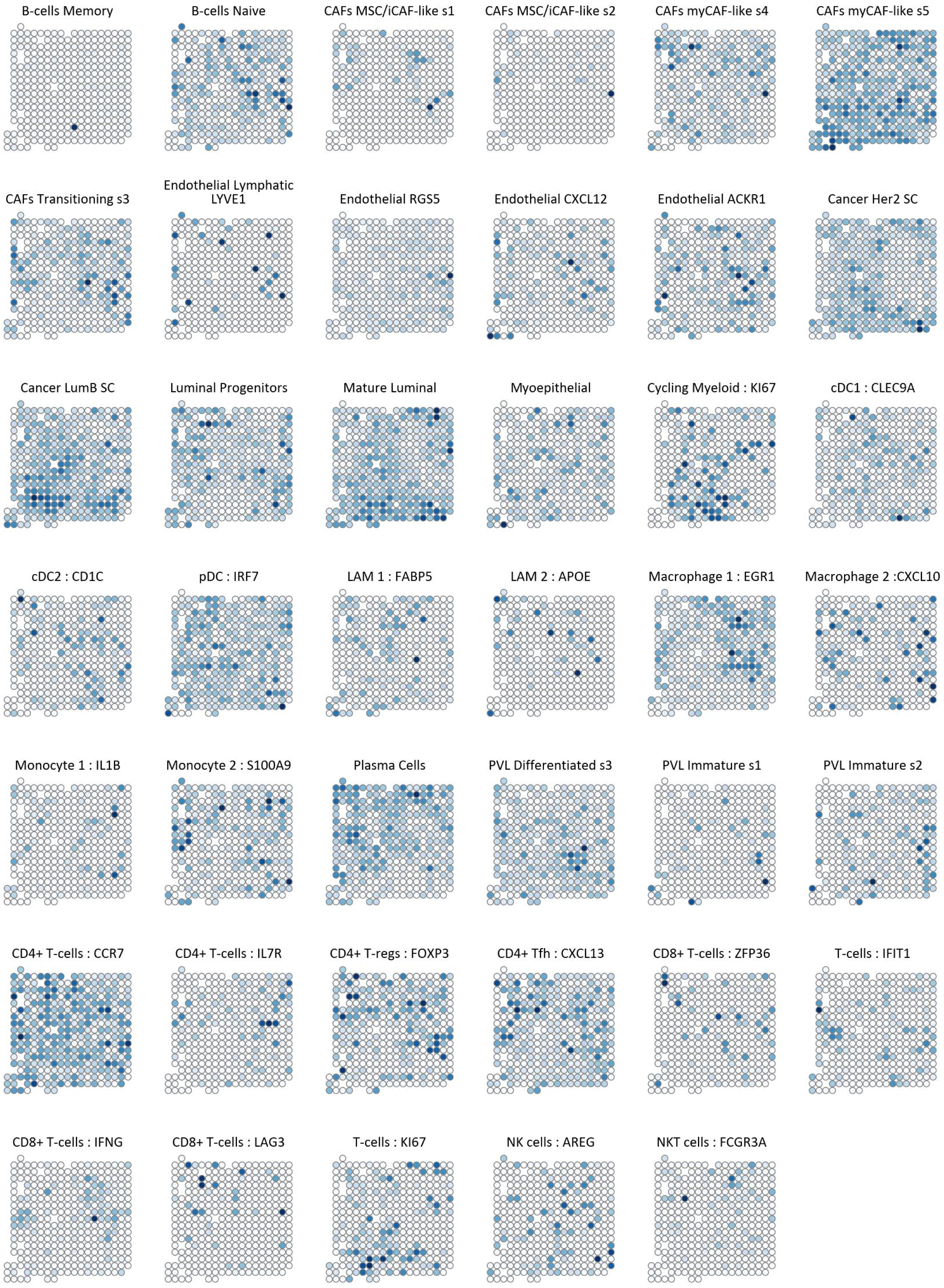

B-cells Memory
B-cells Naive
CAFs MSC/iCAF-like s1
CAFs MSC/iCAF-like s2
CAFs myCAF-like s4
CAFs myCAF-like s5
CAFs Transitioning s3
Endothelial Lymphatic LYVE1
Endothelial RGS5
Endothelial CXCL12
Endothelial ACKR1
Cancer Her2 SC
Cancer LumB SC
Luminal Progenitors
Mature Luminal
Myoepithelial
Cycling Myeloid : KI67
cDC1 : CLEC9A
cDC2 : CD1C
pDC : IRF7
LAM 1 : FABP5
LAM 2 : APOE
Macrophage 1 : EGR1
Macrophage 2 :CXCL10
Monocyte 1 : IL1B
Monocyte 2 : S100A9
Plasma Cells
PVL Differentiated s3
PVL Immature s1
PVL Immature s2
CD4+ T-cells : CCR7
CD4+ T-cells : IL7R
CD4+ T-regs : FOXP3
CD4+ Tfh : CXCL13
CD8+ T-cells : ZFP36
T-cells : IFIT1
CD8+ T-cells : IFNG
CD8+ T-cells : LAG3
T-cells : KI67
NK cells : AREG
NKT cells : FCGR3A

subset_F2

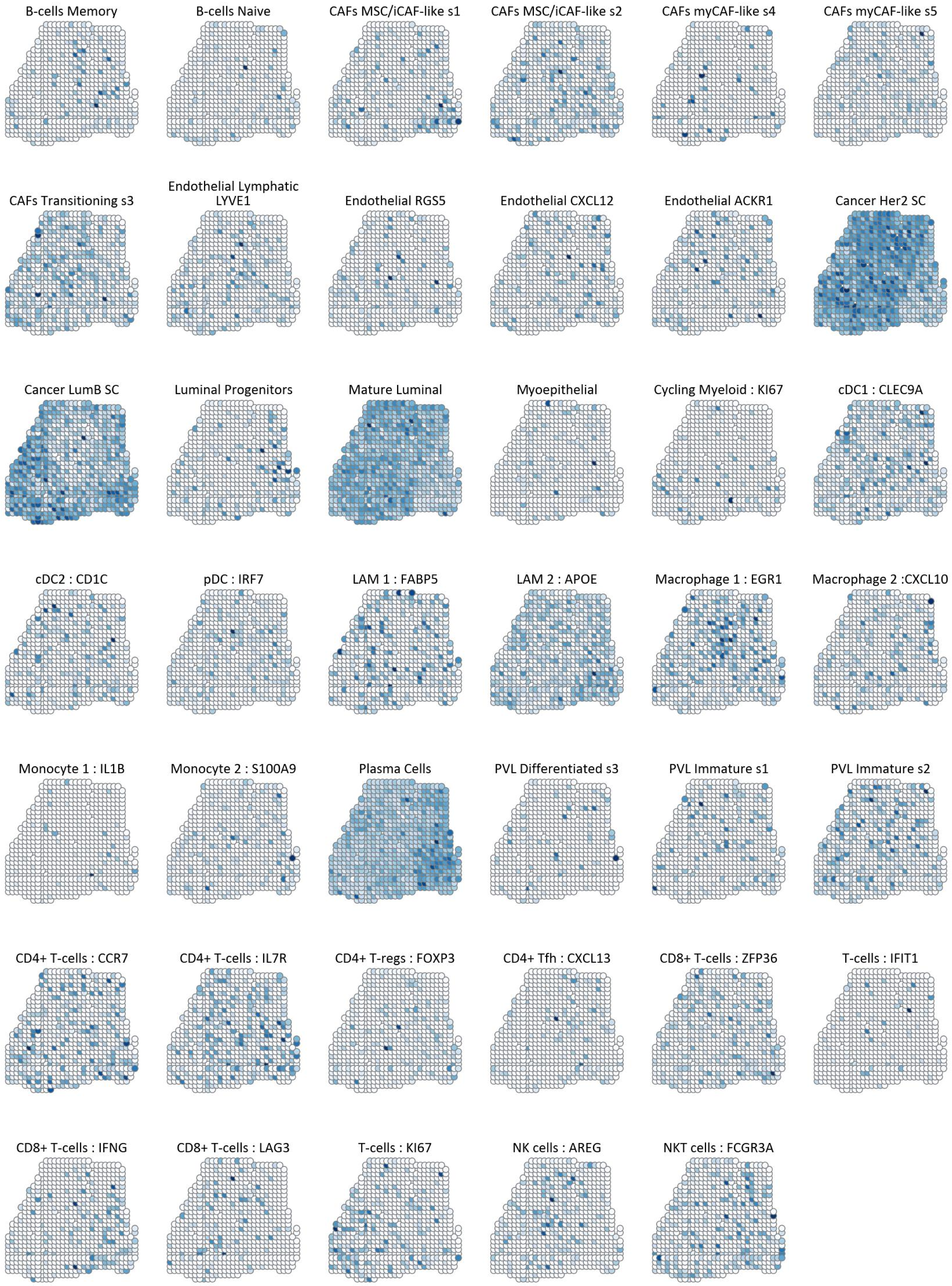
B-cells Memory
B-cells Naive
CAFs MSC/iCAF-like s1
CAFs MSC/iCAF-like s2
CAFs myCAF-like s4
CAFs myCAF-like s5
CAFs Transitioning s3
Endothelial Lymphatic LYVE1
Endothelial RGS5
Endothelial CXCL12
Endothelial ACKR1
Cancer Her2 SC
Cancer LumB SC
Luminal Progenitors
Mature Luminal
Myoepithelial
Cycling Myeloid : KI67
cDC1 : CLEC9A
cDC2 : CD1C
pDC : IRF7
LAM 1 : FABP5
LAM 2 : APOE
Macrophage 1 : EGR1
Macrophage 2 :CXCL10
Monocyte 1 : IL1B
Monocyte 2 : S100A9
Plasma Cells
PVL Differentiated s3
PVL Immature s1
PVL Immature s2
CD4+ T-cells : CCR7
CD4+ T-cells : IL7R
CD4+ T-regs : FOXP3
CD4+ Tfh : CXCL13
CD8+ T-cells : ZFP36
T-cells : IFIT1
CD8+ T-cells : IFNG
CD8+ T-cells : LAG3
T-cells : KI67
NK cells : AREG
NKT cells : FCGR3A

subset_G3

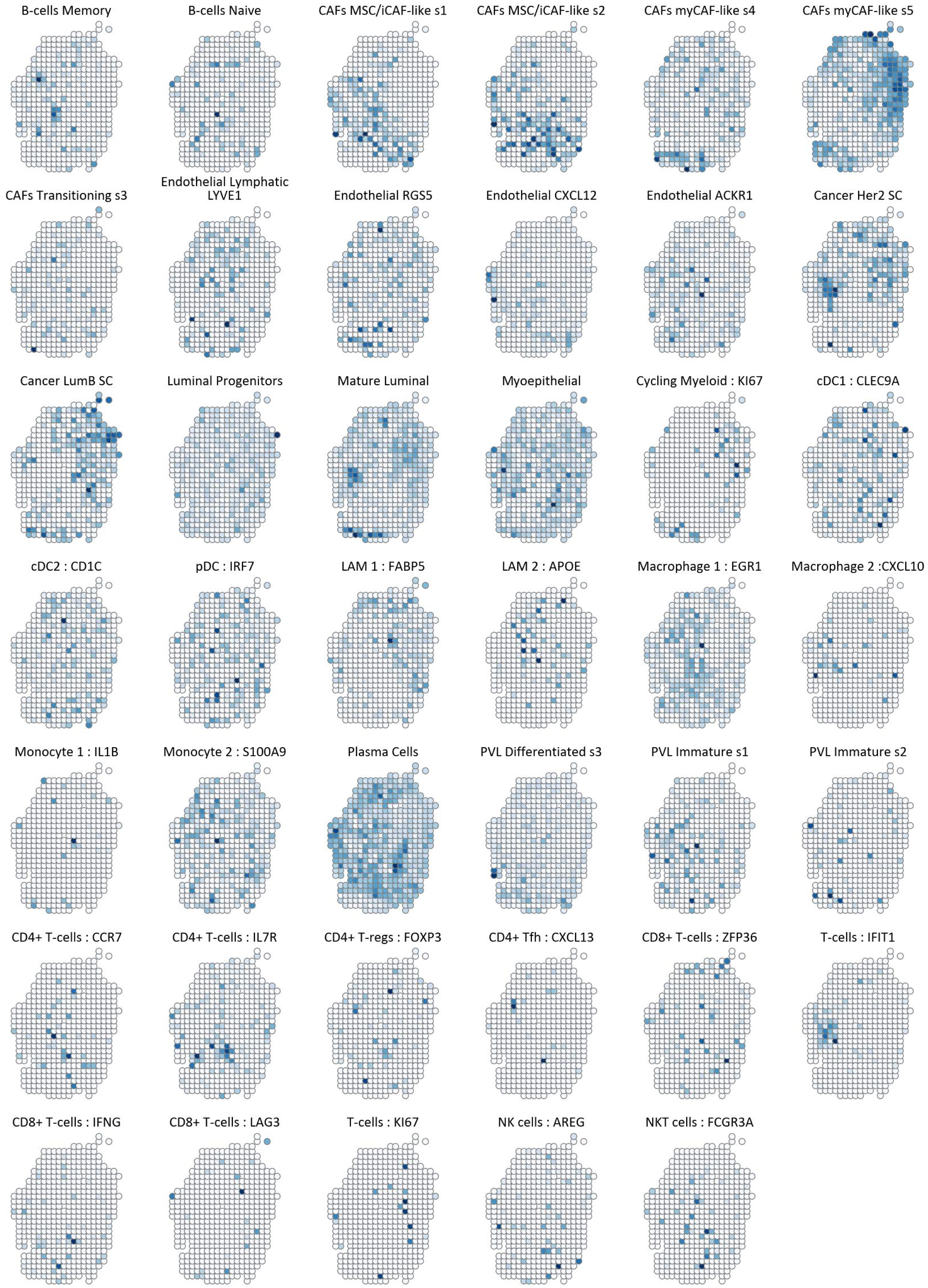
B-cells Memory
B-cells Naive
CAFs MSC/iCAF-like s1
CAFs MSC/iCAF-like s2
CAFs myCAF-like s4
CAFs myCAF-like s5
CAFs Transitioning s3
Endothelial Lymphatic LYVE1
Endothelial RGS5
Endothelial CXCL12
Endothelial ACKR1
Cancer Her2 SC
Cancer LumB SC
Luminal Progenitors
Mature Luminal
Myoepithelial
Cycling Myeloid : KI67
cDC1 : CLEC9A
cDC2 : CD1C
pDC : IRF7
LAM 1 : FABP5
LAM 2 : APOE
Macrophage 1 : EGR1
Macrophage 2 :CXCL10
Monocyte 1 : IL1B
Monocyte 2 : S100A9
Plasma Cells
PVL Differentiated s3
PVL Immature s1
PVL Immature s2
CD4+ T-cells : CCR7
CD4+ T-cells : IL7R
CD4+ T-regs : FOXP3
CD4+ Tfh : CXCL13
CD8+ T-cells : ZFP36
T-cells : IFIT1
CD8+ T-cells : IFNG
CD8+ T-cells : LAG3
T-cells : KI67
NK cells : AREG
NKT cells : FCGR3A

subset_B3

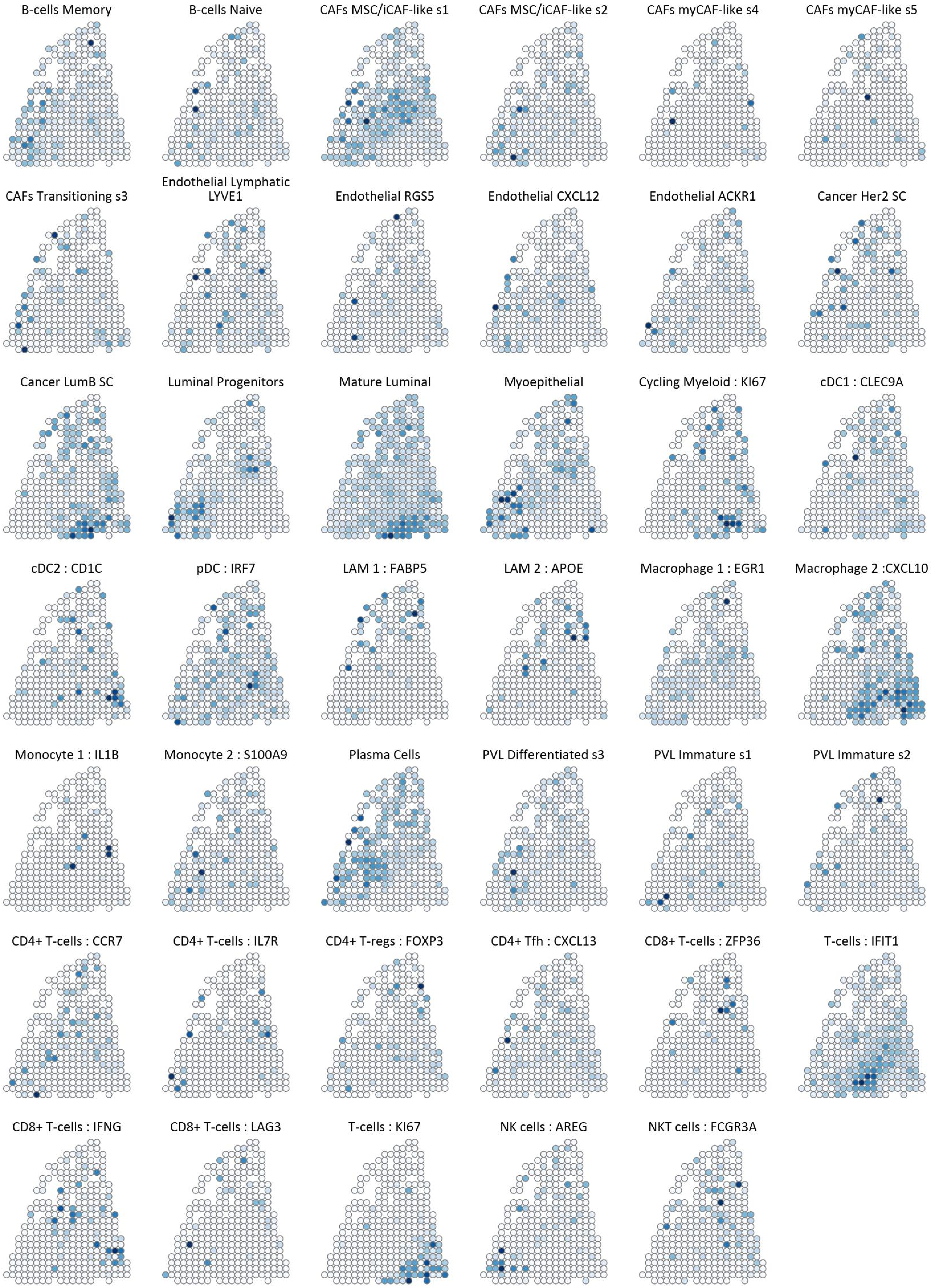
B-cells Memory
B-cells Naive
CAFs MSC/iCAF-like s1
CAFs MSC/iCAF-like s2
CAFs myCAF-like s4
CAFs myCAF-like s5
CAFs Transitioning s3
Endothelial Lymphatic LYVE1
Endothelial RGS5
Endothelial CXCL12
Endothelial ACKR1
Cancer Her2 SC
Cancer LumB SC
Luminal Progenitors
Mature Luminal
Myoepithelial
Cycling Myeloid : KI67
cDC1 : CLEC9A
cDC2 : CD1C
pDC : IRF7
LAM 1 : FABP5
LAM 2 : APOE
Macrophage 1 : EGR1
Macrophage 2 :CXCL10
Monocyte 1 : IL1B
Monocyte 2 : S100A9
Plasma Cells
PVL Differentiated s3
PVL Immature s1
PVL Immature s2
CD4+ T-cells : CCR7
CD4+ T-cells : IL7R
CD4+ T-regs : FOXP3
CD4+ Tfh : CXCL13
CD8+ T-cells : ZFP36
T-cells : IFIT1
CD8+ T-cells : IFNG
CD8+ T-cells : LAG3
T-cells : KI67
NK cells : AREG
NKT cells : FCGR3A

subset_B6

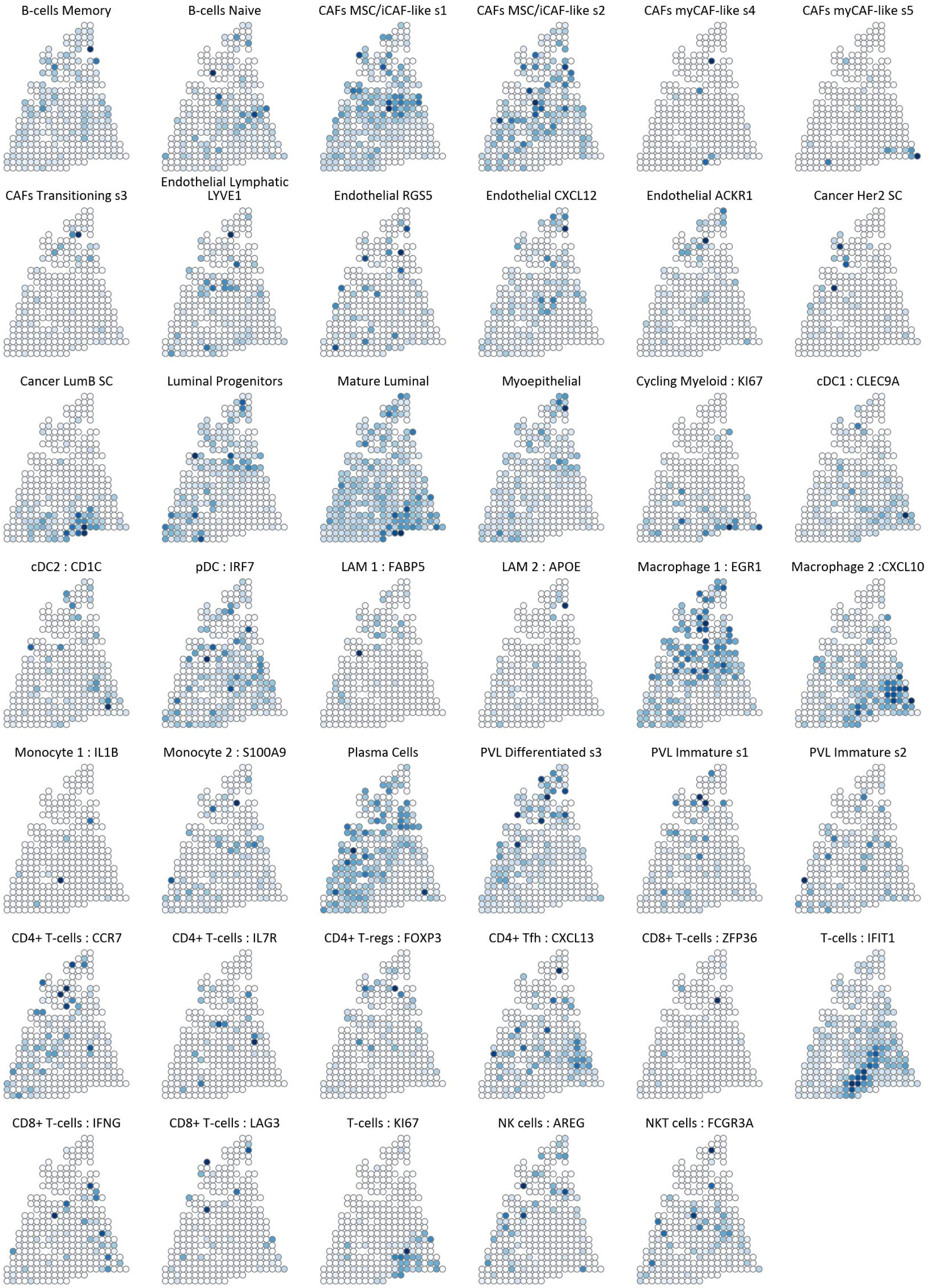

B-cells Memory
B-cells Naive
CAFs MSC/iCAF-like s1
CAFs MSC/iCAF-like s2
CAFs myCAF-like s4
CAFs myCAF-like s5
CAFs Transitioning s3
Endothelial Lymphatic LYVE1
Endothelial RGS5
Endothelial CXCL12
Endothelial ACKR1
Cancer Her2 SC
Cancer LumB SC
Luminal Progenitors
Mature Luminal
Myoepithelial
Cycling Myeloid : KI67
cDC1 : CLEC9A
cDC2 : CD1C
pDC : IRF7
LAM 1 : FABP5
LAM 2 : APOE
Macrophage 1 : EGR1
Macrophage 2 :CXCL10
Monocyte 1 : IL1B
Monocyte 2 : S100A9
Plasma Cells
PVL Differentiated s3
PVL Immature s1
PVL Immature s2
CD4+ T-cells : CCR7
CD4+ T-cells : IL7R
CD4+ T-regs : FOXP3
CD4+ Tfh : CXCL13
CD8+ T-cells : ZFP36
T-cells : IFIT1
CD8+ T-cells : IFNG
CD8+ T-cells : LAG3
T-cells : KI67
NK cells : AREG
NKT cells : FCGR3A

subset_C3

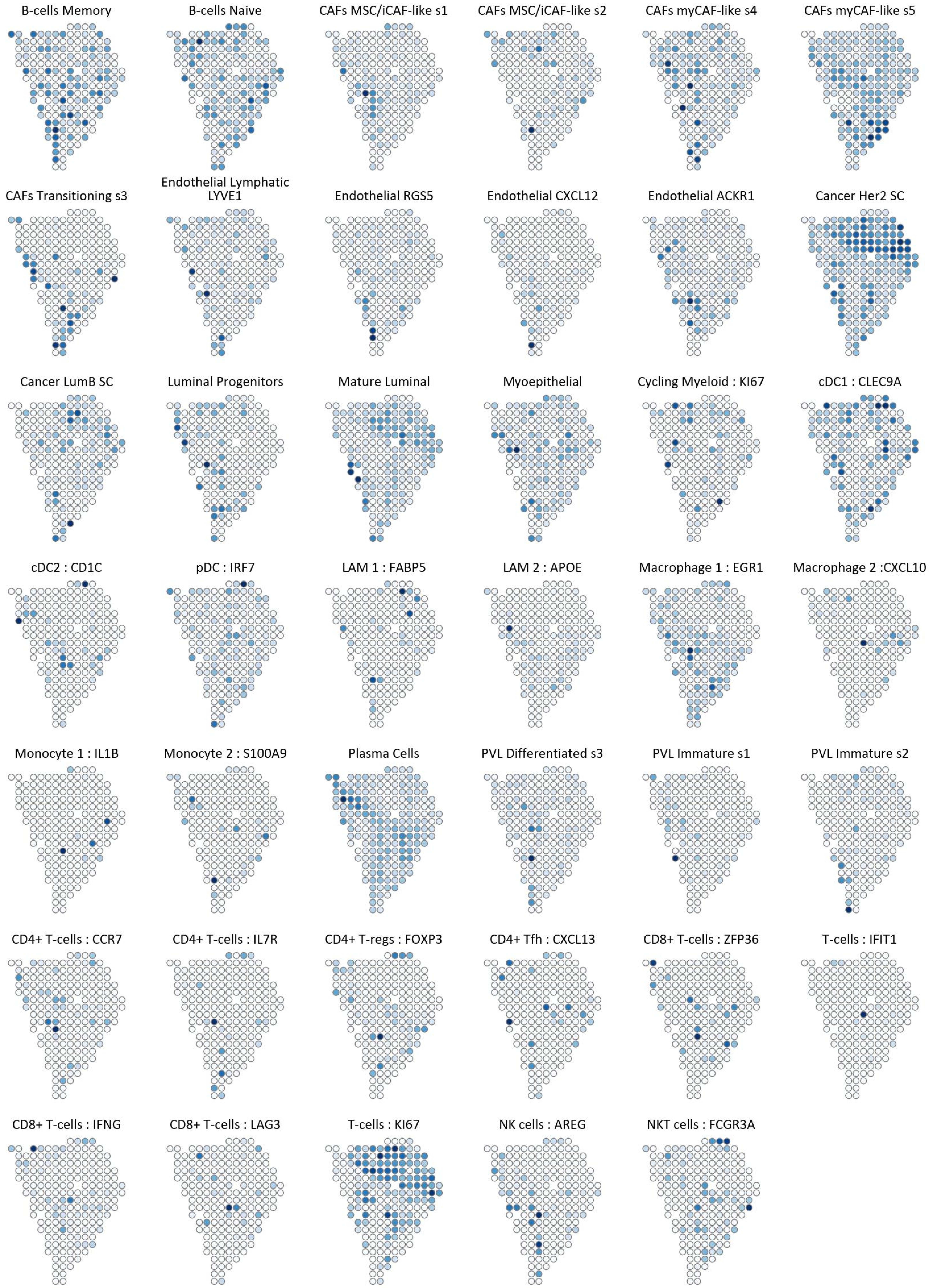
B-cells Memory
B-cells Naive
CAFs MSC/iCAF-like s1
CAFs MSC/iCAF-like s2
CAFs myCAF-like s4
CAFs myCAF-like s5
CAFs Transitioning s3
Endothelial Lymphatic LYVE1
Endothelial RGS5
Endothelial CXCL12
Endothelial ACKR1
Cancer Her2 SC
Cancer LumB SC
Luminal Progenitors
Mature Luminal
Myoepithelial
Cycling Myeloid : KI67
cDC1 : CLEC9A
cDC2 : CD1C
pDC : IRF7
LAM 1 : FABP5
LAM 2 : APOE
Macrophage 1 : EGR1
Macrophage 2 :CXCL10
Monocyte 1 : IL1B
Monocyte 2 : S100A9
Plasma Cells
PVL Differentiated s3
PVL Immature s1
PVL Immature s2
CD4+ T-cells : CCR7
CD4+ T-cells : IL7R
CD4+ T-regs : FOXP3
CD4+ Tfh : CXCL13
CD8+ T-cells : ZFP36
T-cells : IFIT1
CD8+ T-cells : IFNG
CD8+ T-cells : LAG3
T-cells : KI67
NK cells : AREG
NKT cells : FCGR3A
