# Supplementary material for: Spatial deconvolution of HER2-positive breast cancer delineates tumor-associated cell type interactions
Source: Nat Commun. 2021 Oct 14;12:6012. doi: 10.1038/s41467-021-26271-2 (PMC8516894; doi:10.1038/s41467-021-26271-2)

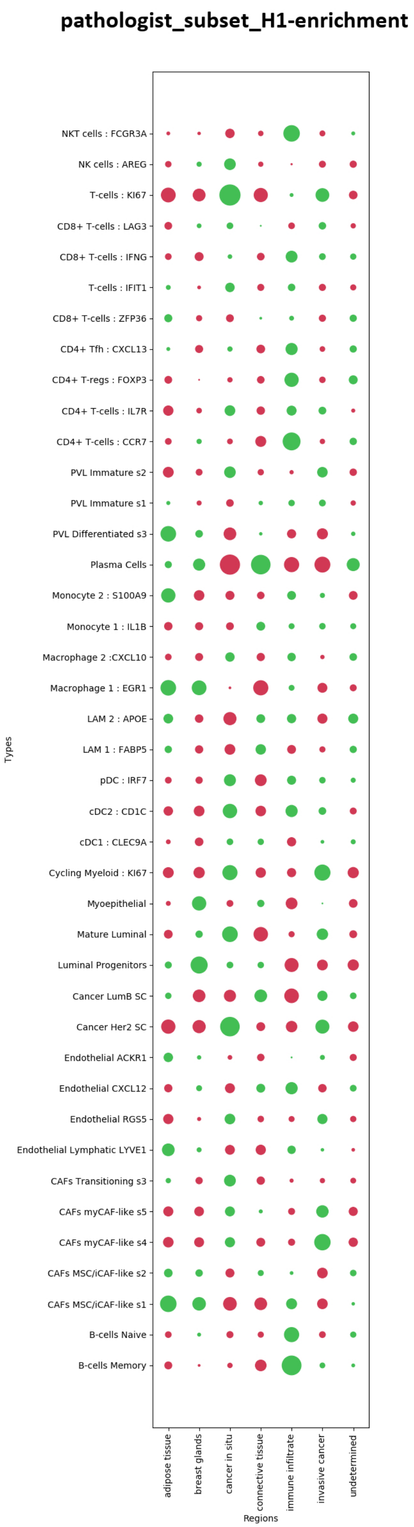
pathologist_subset_H1-enrichment
NKT cells : FCGR3A
NK cells : AREG
T-cells : KI67
CD8+ T-cells : LAG3
CD8+ T-cells : IFNG
T-cells : IFIT1
CD8+ T-cells : ZFP36
CD4+ Tfh : CXCL13
CD4+ T-regs : FOXP3
CD4+ T-cells : IL7R
CD4+ T-cells : CCR7
PVL Immature s2
PVL Immature s1
PVL Differentiated s3
Plasma Cells
Monocyte 2 : S100A9
Monocyte 1 : IL1B
Macrophage 2 :CXCL10
Macrophage 1 : EGR1
LAM 2 : APOE
LAM 1 : FABP5
pDC : IRF7
cDC2 : CD1C
cDC1 : CLEC9A
Cycling Myeloid : KI67
Myoepithelial
Mature Luminal
Luminal Progenitors
Cancer LumB SC
Cancer Her2 SC
Endothelial ACKR1
Endothelial CXCL12
Endothelial RGS5
Endothelial Lymphatic LYVE1
CAFs Transitioning s3
CAFs myCAF-like s5
CAFs myCAF-like s4
CAFs MSC/iCAF-like s2
CAFs MSC/iCAF-like s1
B-cells Naive
B-cells Memory
Types
adipose tissue
breast glands
cancer in situ
connective tissue
immune infiltrate
invasive cancer
undetermined
Regions

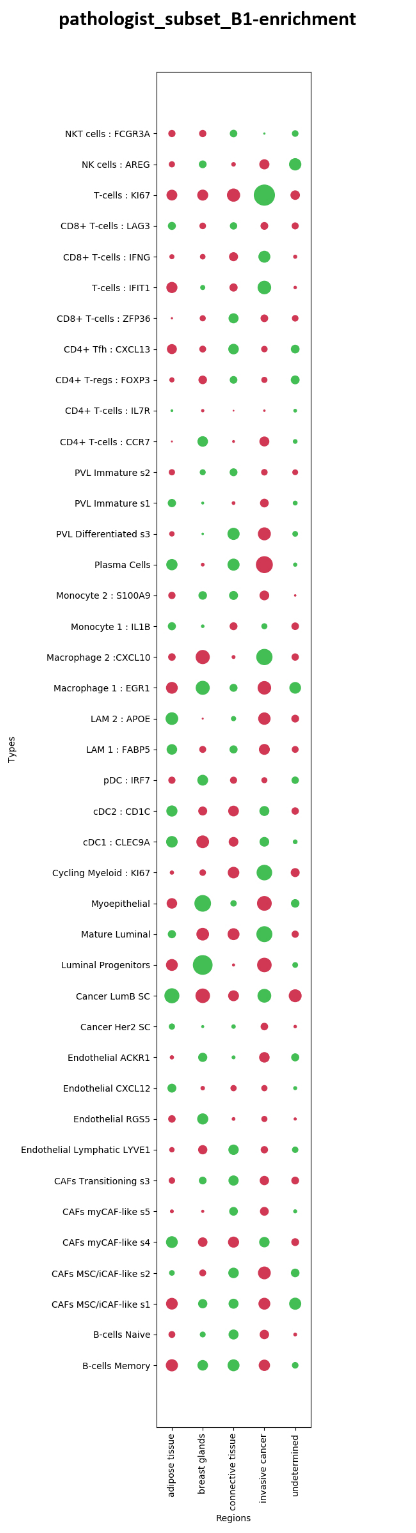
pathologist_subset_B1-enrichment
NKT cells : FCGR3A
NK cells : AREG
T-cells : KI67
CD8+ T-cells : LAG3
CD8+ T-cells : IFNG
T-cells : IFIT1
CD8+ T-cells : ZFP36
CD4+ Tfh : CXCL13
CD4+ T-regs : FOXP3
CD4+ T-cells : IL7R
CD4+ T-cells : CCR7
PVL Immature s2
PVL Immature s1
PVL Differentiated s3
Plasma Cells
Monocyte 2 : S100A9
Monocyte 1 : IL1B
Macrophage 2 :CXCL10
Macrophage 1 : EGR1
LAM 2 : APOE
LAM 1 : FABP5
pDC : IRF7
cDC2 : CD1C
cDC1 : CLEC9A
Cycling Myeloid : KI67
Myoepithelial
Mature Luminal
Luminal Progenitors
Cancer LumB SC
Cancer Her2 SC
Endothelial ACKR1
Endothelial CXCL12
Endothelial RGS5
Endothelial Lymphatic LYVE1
CAFs Transitioning s3
CAFs myCAF-like s5
CAFs myCAF-like s4
CAFs MSC/iCAF-like s2
CAFs MSC/iCAF-like s1
B-cells Naive
B-cells Memory
Types
adipose tissue
breast glands
connective tissue
invasive cancer
undetermined
Regions

pathologist_subset_F1-enrichment

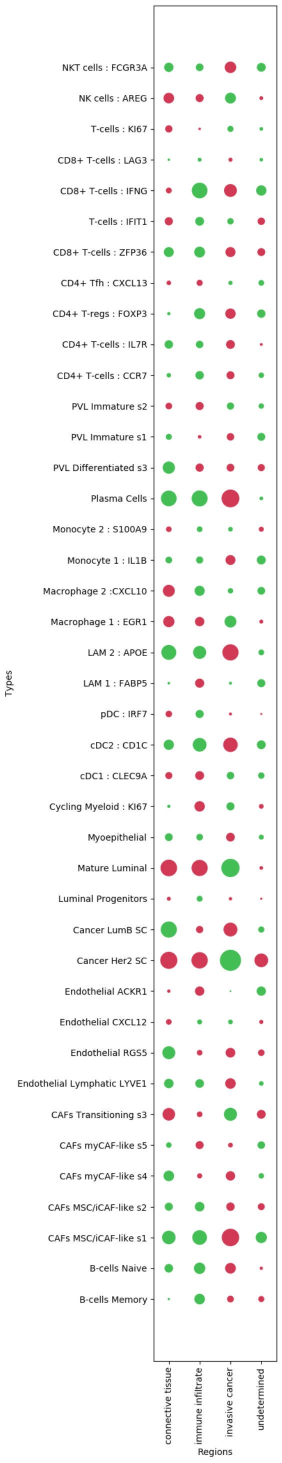

pathologist_subset_C1-enrichment

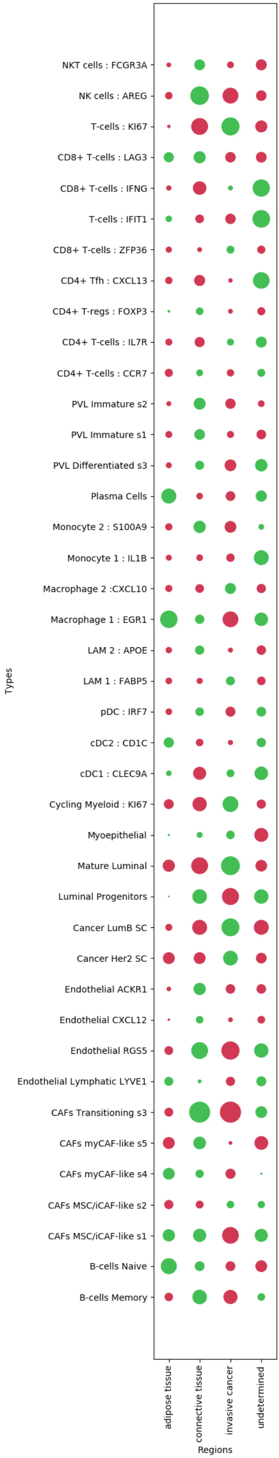

pathologist_subset_G2-enrichment

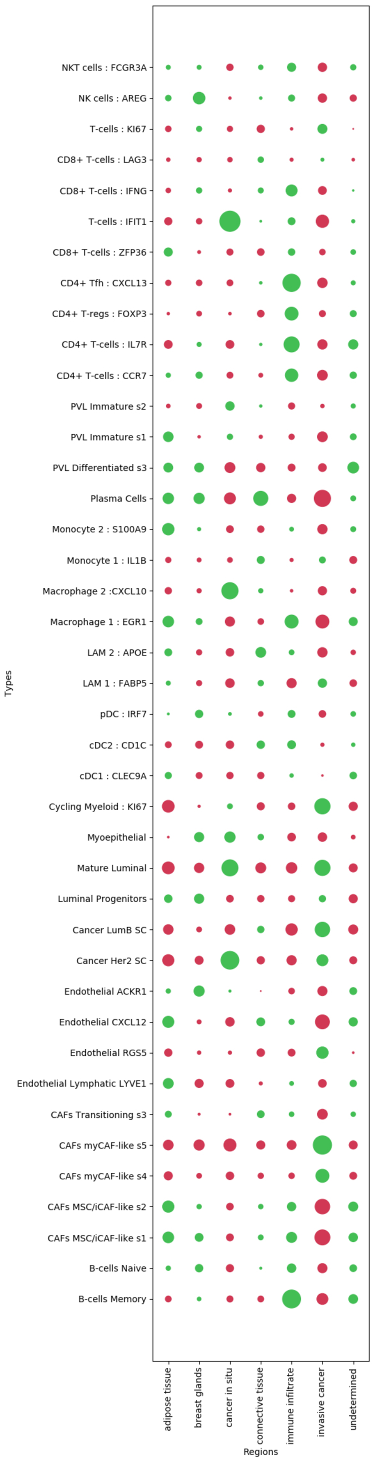

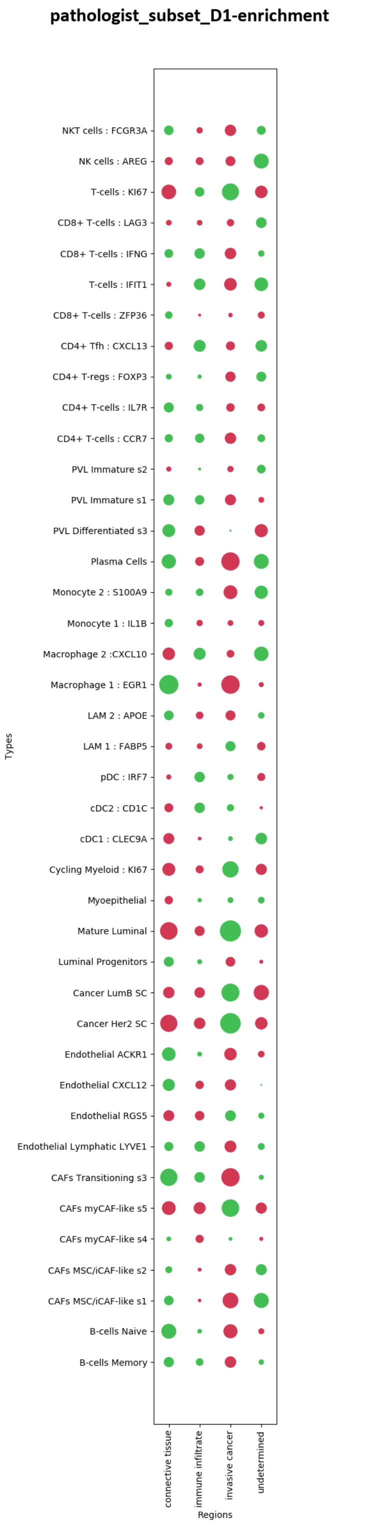
pathologist_subset_D1-enrichment
NKT cells : FCGR3A
NK cells : AREG
T-cells : KI67
CD8+ T-cells : LAG3
CD8+ T-cells : IFNG
T-cells : IFIT1
CD8+ T-cells : ZFP36
CD4+ Tfh : CXCL13
CD4+ T-regs : FOXP3
CD4+ T-cells : IL7R
CD4+ T-cells : CCR7
PVL Immature s2
PVL Immature s1
PVL Differentiated s3
Plasma Cells
Monocyte 2 : S100A9
Monocyte 1 : IL1B
Macrophage 2 :CXCL10
Macrophage 1 : EGR1
LAM 2 : APOE
LAM 1 : FABP5
pDC : IRF7
cDC2 : CD1C
cDC1 : CLEC9A
Cycling Myeloid : KI67
Myoepithelial
Mature Luminal
Luminal Progenitors
Cancer LumB SC
Cancer Her2 SC
Endothelial ACKR1
Endothelial CXCL12
Endothelial RGS5
Endothelial Lymphatic LYVE1
CAFs Transitioning s3
CAFs myCAF-like s5
CAFs myCAF-like s4
CAFs MSC/iCAF-like s2
CAFs MSC/iCAF-like s1
B-cells Naive
B-cells Memory
Types
connective tissue
immune infiltrate
invasive cancer
undetermined
Regions

pathologist_subset_A1-enrichment

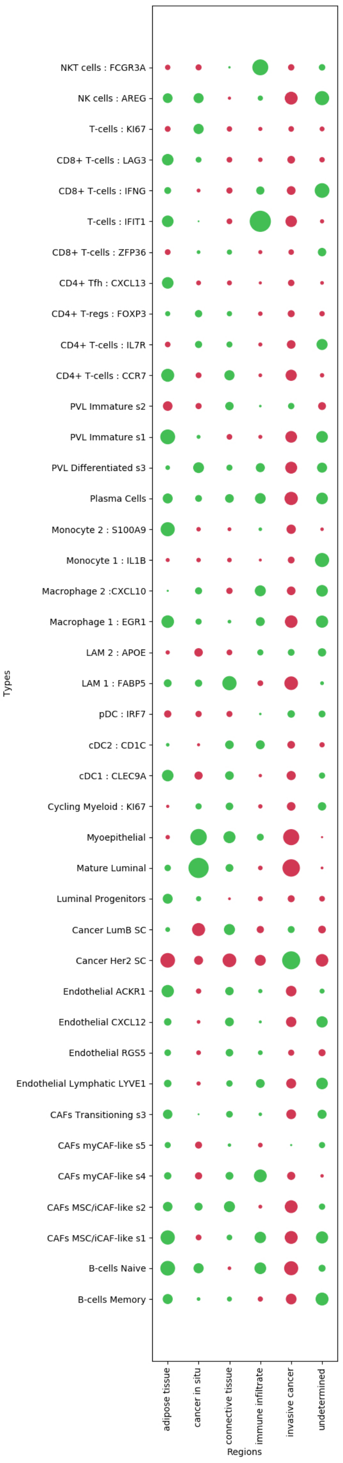

pathologist_subset_E1-enrichment

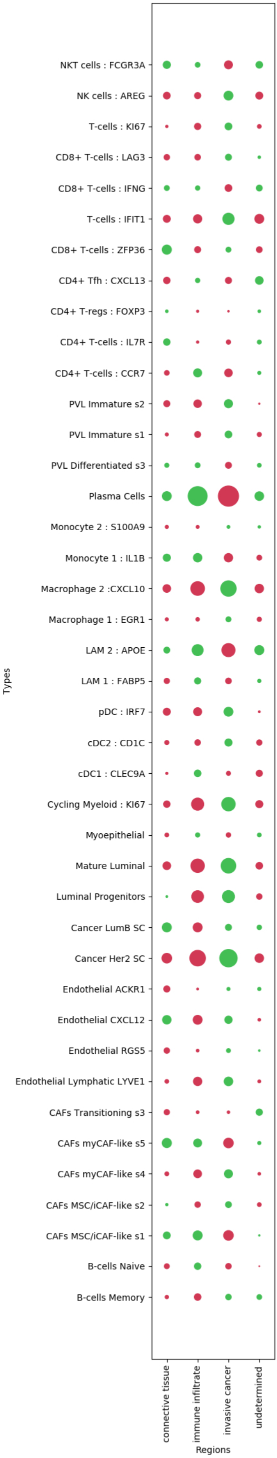

pathologist_major_H1-enrichment

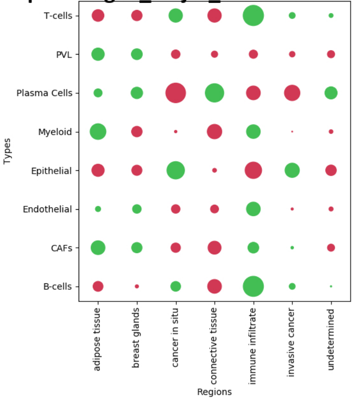

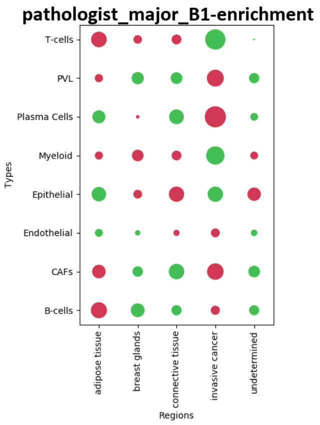
pathologist_major_B1-enrichment
T-cells
PVL
Plasma Cells
Myeloid
Epithelial
Endothelial
CAFs
B-cells
Types
adipose tissue
breast glands
connective tissue
invasive cancer
undetermined
Regions

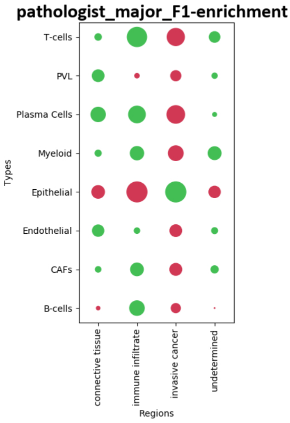
pathologist_major_F1-enrichment
T-cells
PVL
Plasma Cells
Myeloid
Epithelial
Endothelial
CAFs
B-cells
Types
connective tissue
immune infiltrate
invasive cancer
undetermined
Regions

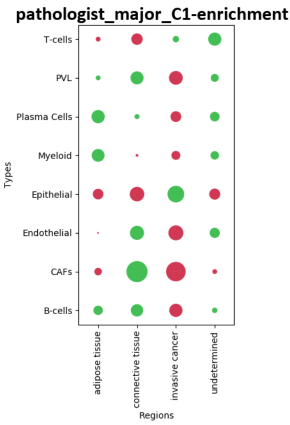
pathologist_major_C1-enrichment
T-cells
PVL
Plasma Cells
Myeloid
Epithelial
Endothelial
CAFs
B-cells
Types
adipose tissue
connective tissue
invasive cancer
undetermined
Regions

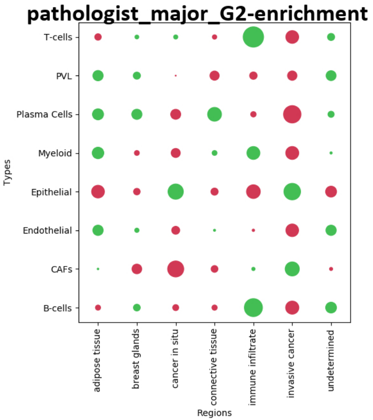

pathologist_major_G2-enrichment
T-cells
PVL
Plasma Cells
Myeloid
Epithelial
Endothelial
CAFs
B-cells
Types
adipose tissue
breast glands
cancer in situ
connective tissue
immune infiltrate
invasive cancer
undetermined
Regions

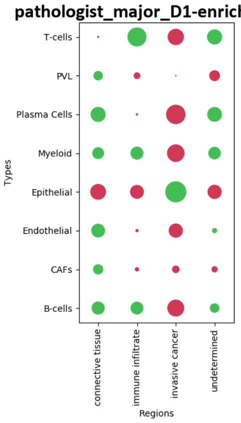
pathologist_major_D1-enrichment
T-cells
PVL
Plasma Cells
Myeloid
Epithelial
Endothelial
CAFs
B-cells
Types
connective tissue
immune infiltrate
invasive cancer
undetermined
Regions

pathologist_major_A1-enrichment

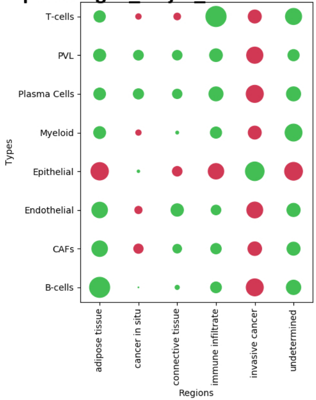

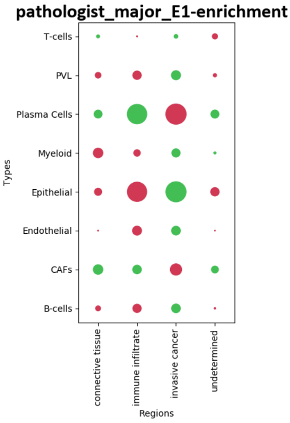

pathologist_major_E1-enrichment
T-cells
PVL
Plasma Cells
Myeloid
Epithelial
Endothelial
CAFs
B-cells
Types
connective tissue
immune infiltrate
invasive cancer
undetermined
Regions

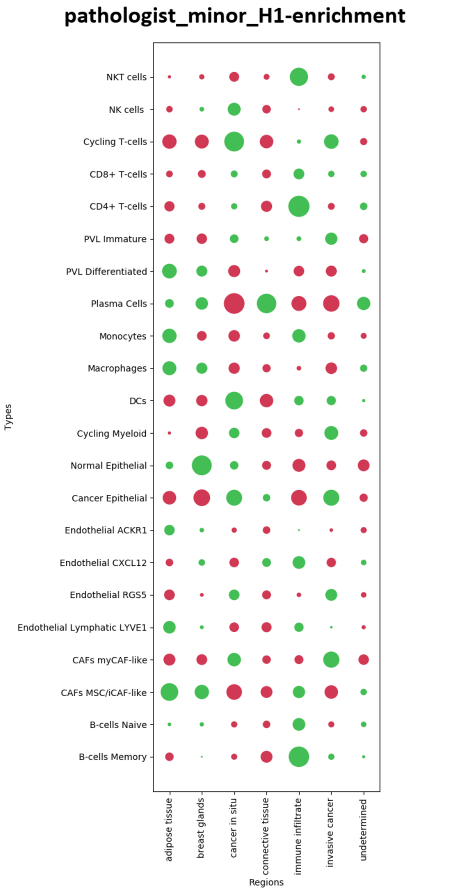
pathologist_minor_H1-enrichment
NKT cells
NK cells
Cycling T-cells
CD8+ T-cells
CD4+ T-cells
PVL Immature
PVL Differentiated
Plasma Cells
Monocytes
Macrophages
DCs
Cycling Myeloid
Normal Epithelial
Cancer Epithelial
Endothelial ACKR1
Endothelial CXCL12
Endothelial RGS5
Endothelial Lymphatic LYVE1
CAFs myCAF-like
CAFs MSC/iCAF-like
B-cells Naive
B-cells Memory
Types
adipose tissue
breast glands
cancer in situ
connective tissue
immune infiltrate
invasive cancer
undetermined
Regions

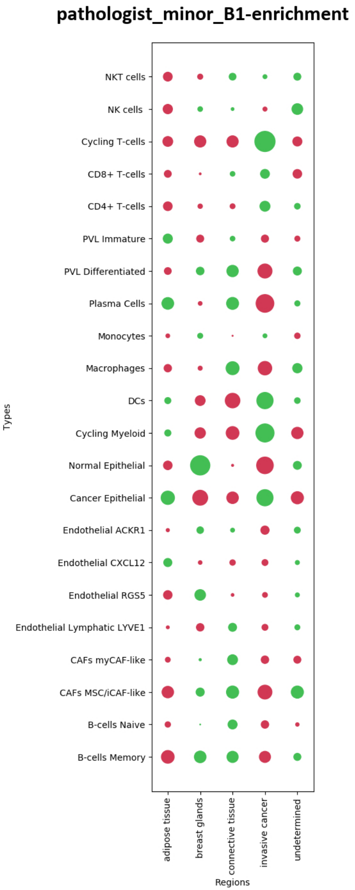

pathologist_minor_B1-enrichment
NKT cells
NK cells
Cycling T-cells
CD8+ T-cells
CD4+ T-cells
PVL Immature
PVL Differentiated
Plasma Cells
Monocytes
Macrophages
DCs
Cycling Myeloid
Normal Epithelial
Cancer Epithelial
Endothelial ACKR1
Endothelial CXCL12
Endothelial RGS5
Endothelial Lymphatic LYVE1
CAFs myCAF-like
CAFs MSC/iCAF-like
B-cells Naive
B-cells Memory
Types
adipose tissue
breast glands
connective tissue
invasive cancer
undetermined
Regions

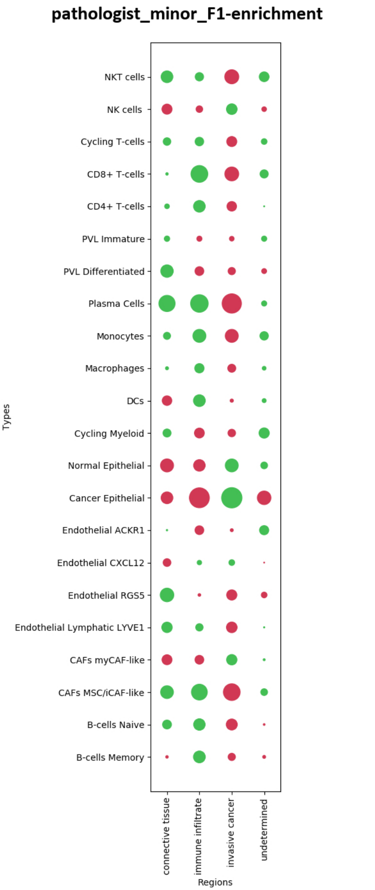
pathologist_minor_F1-enrichment
NKT cells
NK cells
Cycling T-cells
CD8+ T-cells
CD4+ T-cells
PVL Immature
PVL Differentiated
Plasma Cells
Monocytes
Macrophages
DCs
Cycling Myeloid
Normal Epithelial
Cancer Epithelial
Endothelial ACKR1
Endothelial CXCL12
Endothelial RGS5
Endothelial Lymphatic LYVE1
CAFs myCAF-like
CAFs MSC/iCAF-like
B-cells Naive
B-cells Memory
Types
connective tissue
immune infiltrate
invasive cancer
undetermined
Regions

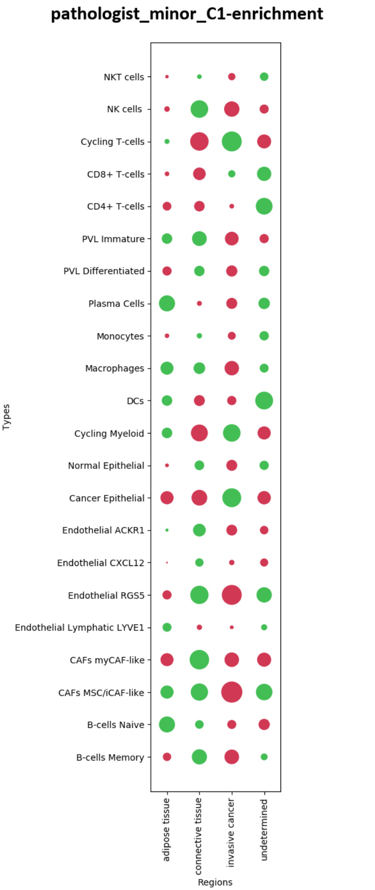
pathologist_minor_C1-enrichment
NKT cells
NK cells
Cycling T-cells
CD8+ T-cells
CD4+ T-cells
PVL Immature
PVL Differentiated
Plasma Cells
Monocytes
Macrophages
DCs
Cycling Myeloid
Normal Epithelial
Cancer Epithelial
Endothelial ACKR1
Endothelial CXCL12
Endothelial RGS5
Endothelial Lymphatic LYVE1
CAFs myCAF-like
CAFs MSC/iCAF-like
B-cells Naive
B-cells Memory
Types
adipose tissue
connective tissue
invasive cancer
undetermined
Regions

pathologist_minor_G2-enrichment

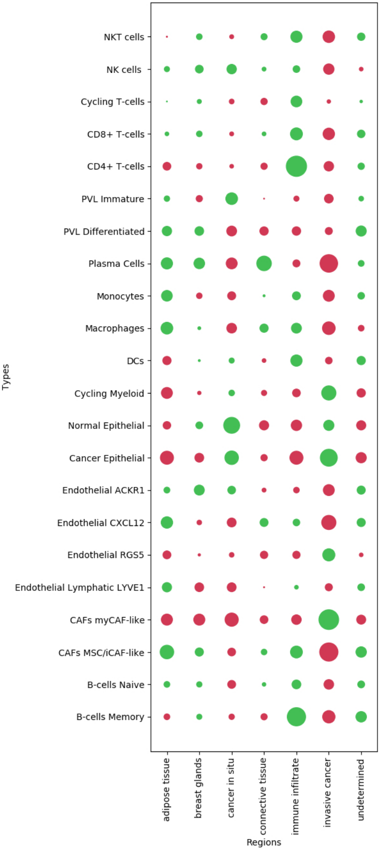

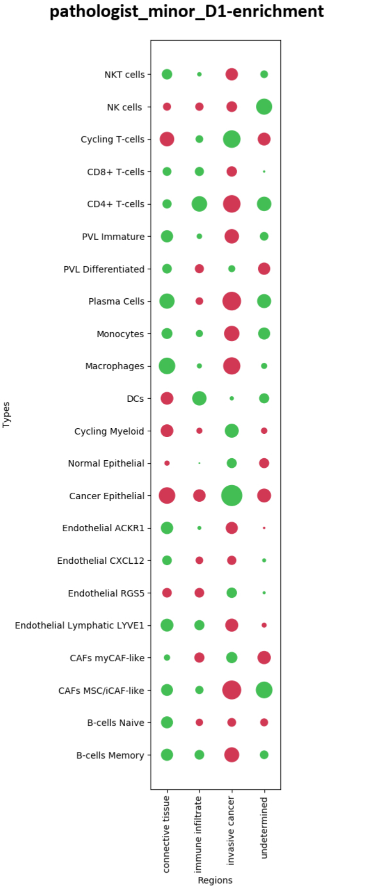
pathologist_minor_D1-enrichment
NKT cells
NK cells
Cycling T-cells
CD8+ T-cells
CD4+ T-cells
PVL Immature
PVL Differentiated
Plasma Cells
Monocytes
Macrophages
DCs
Cycling Myeloid
Normal Epithelial
Cancer Epithelial
Endothelial ACKR1
Endothelial CXCL12
Endothelial RGS5
Endothelial Lymphatic LYVE1
CAFs myCAF-like
CAFs MSC/iCAF-like
B-cells Naive
B-cells Memory
Types
connective tissue
immune infiltrate
invasive cancer
undetermined
Regions

pathologist_minor_A1-enrichment

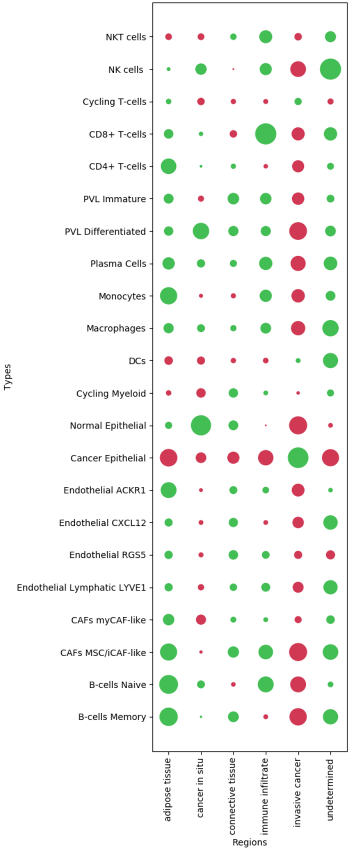

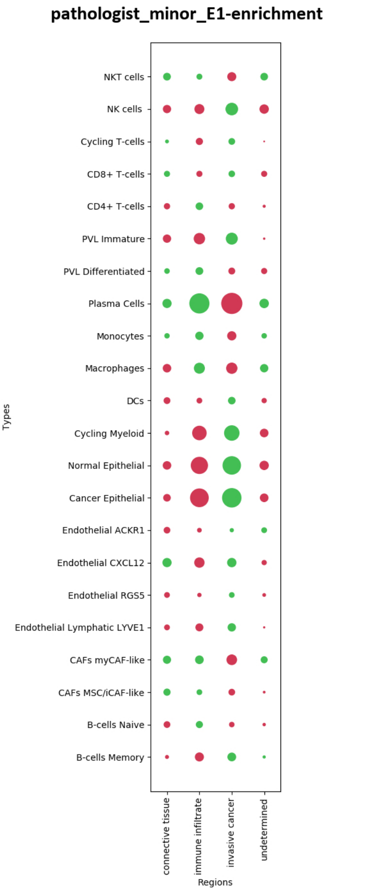

pathologist_minor_E1-enrichment
NKT cells
NK cells
Cycling T-cells
CD8+ T-cells
CD4+ T-cells
PVL Immature
PVL Differentiated
Plasma Cells
Monocytes
Macrophages
DCs
Cycling Myeloid
Normal Epithelial
Cancer Epithelial
Endothelial ACKR1
Endothelial CXCL12
Endothelial RGS5
Endothelial Lymphatic LYVE1
CAFs myCAF-like
CAFs MSC/iCAF-like
B-cells Naive
B-cells Memory
Types
connective tissue
immune infiltrate
invasive cancer
undetermined
Regions

cluster_subset_C6-enrichment

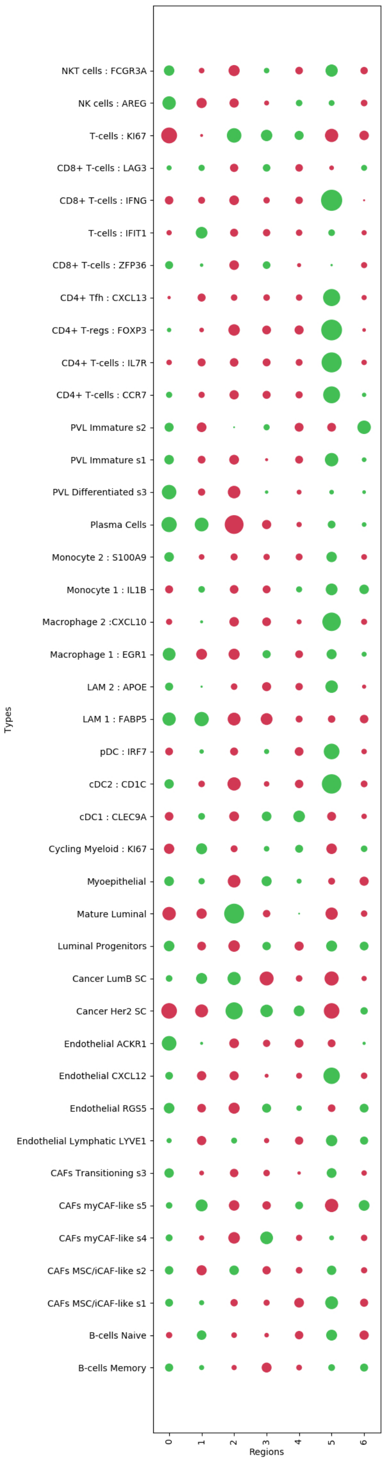

cluster_subset_C4-enrichment

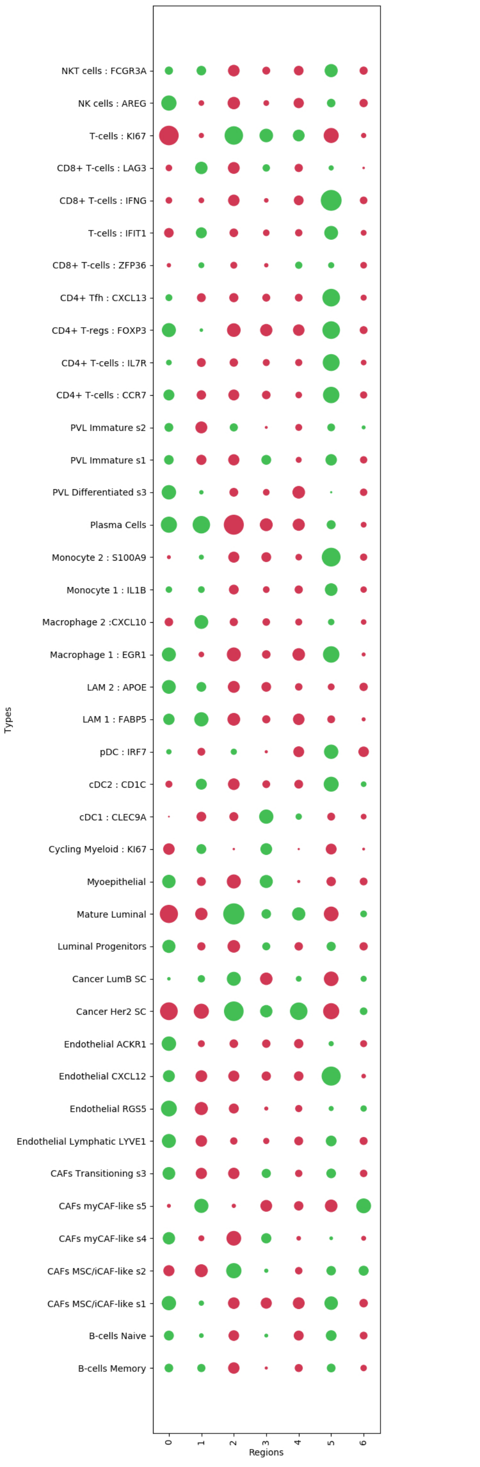

cluster_subset_C3-enrichment

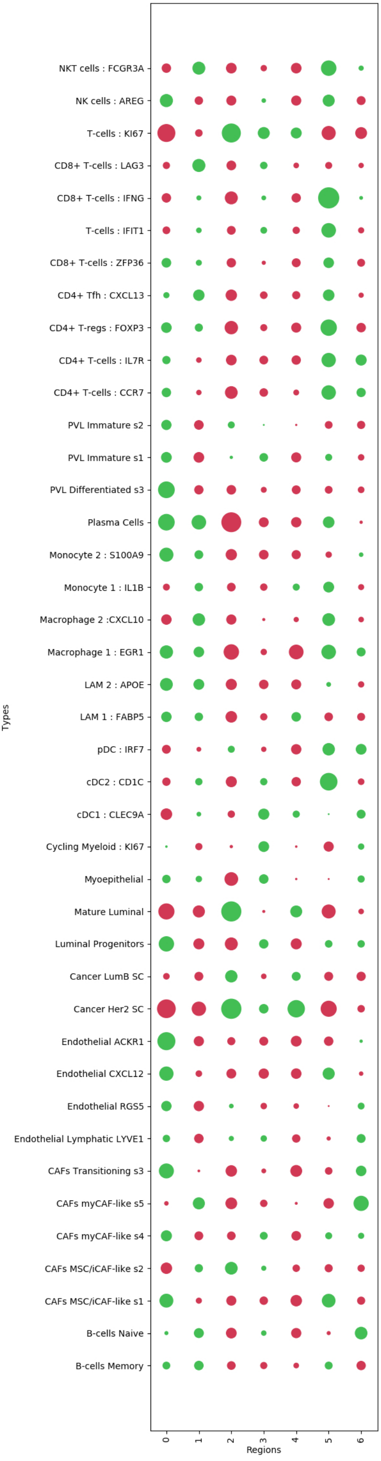

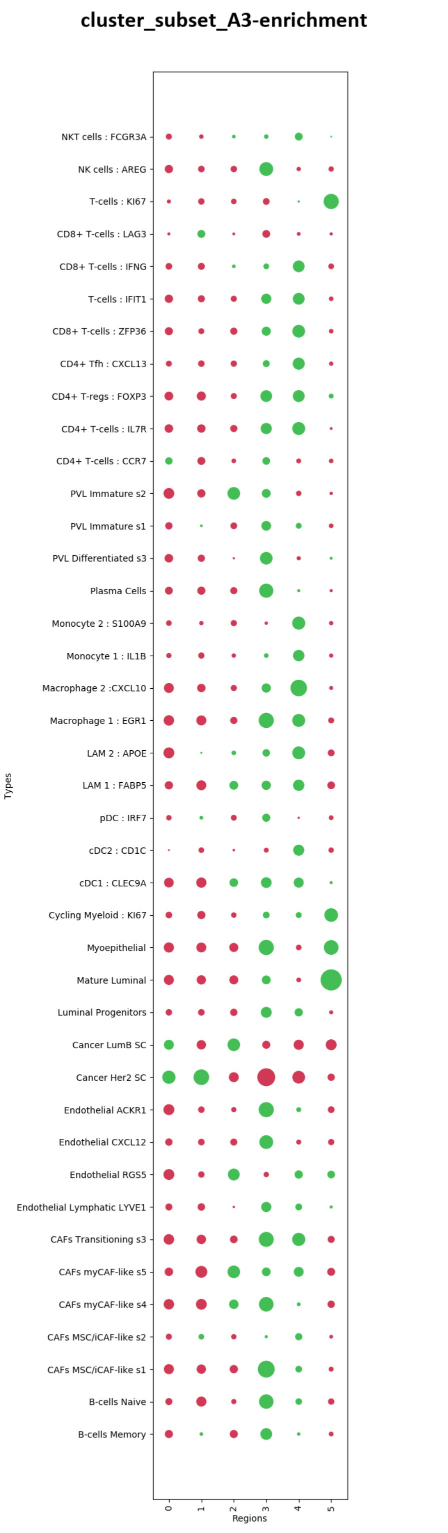
cluster_subset_A3-enrichment
NKT cells : FCGR3A
NK cells : AREG
T-cells : KI67
CD8+ T-cells : LAG3
CD8+ T-cells : IFNG
T-cells : IFIT1
CD8+ T-cells : ZFP36
CD4+ Tfh : CXCL13
CD4+ T-regs : FOXP3
CD4+ T-cells : IL7R
CD4+ T-cells : CCR7
PVL Immature s2
PVL Immature s1
PVL Differentiated s3
Plasma Cells
Monocyte 2 : S100A9
Monocyte 1 : IL1B
Macrophage 2 :CXCL10
Macrophage 1 : EGR1
LAM 2 : APOE
LAM 1 : FABP5
pDC : IRF7
cDC2 : CD1C
cDC1 : CLEC9A
Cycling Myeloid : KI67
Myoepithelial
Mature Luminal
Luminal Progenitors
Cancer LumB SC
Cancer Her2 SC
Endothelial ACKR1
Endothelial CXCL12
Endothelial RGS5
Endothelial Lymphatic LYVE1
CAFs Transitioning s3
CAFs myCAF-like s5
CAFs myCAF-like s4
CAFs MSC/iCAF-like s2
CAFs MSC/iCAF-like s1
B-cells Naive
B-cells Memory
Types
0
1
2
3
4
5
Regions

cluster_subset_H1-enrichment

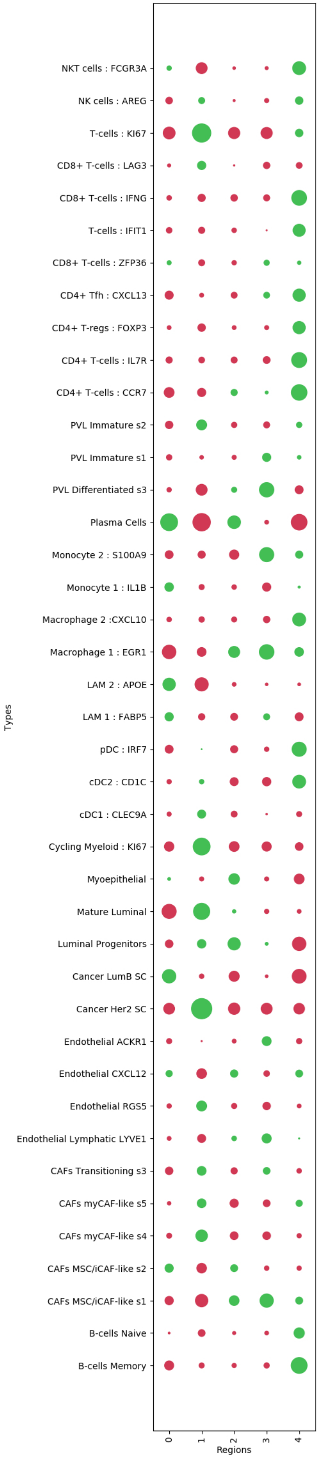

cluster_subset_B4-enrichment

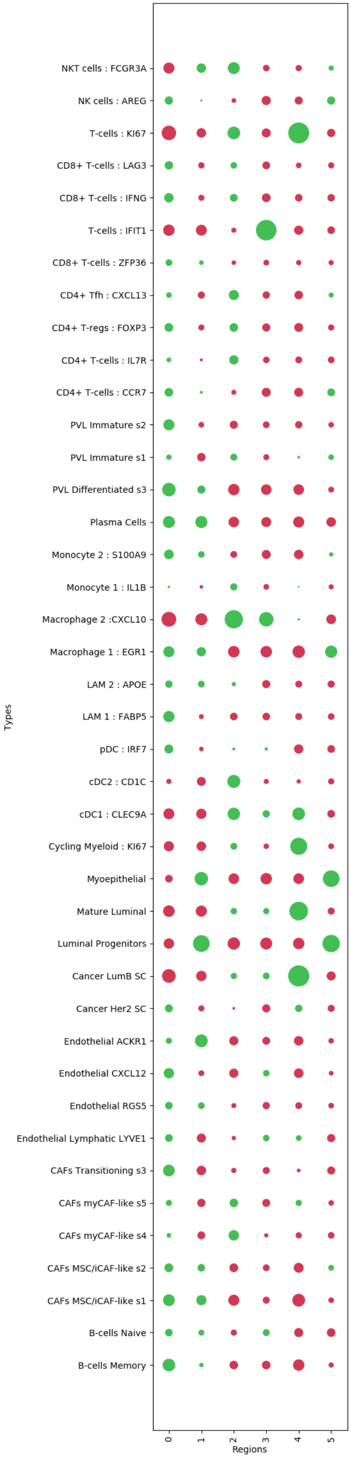

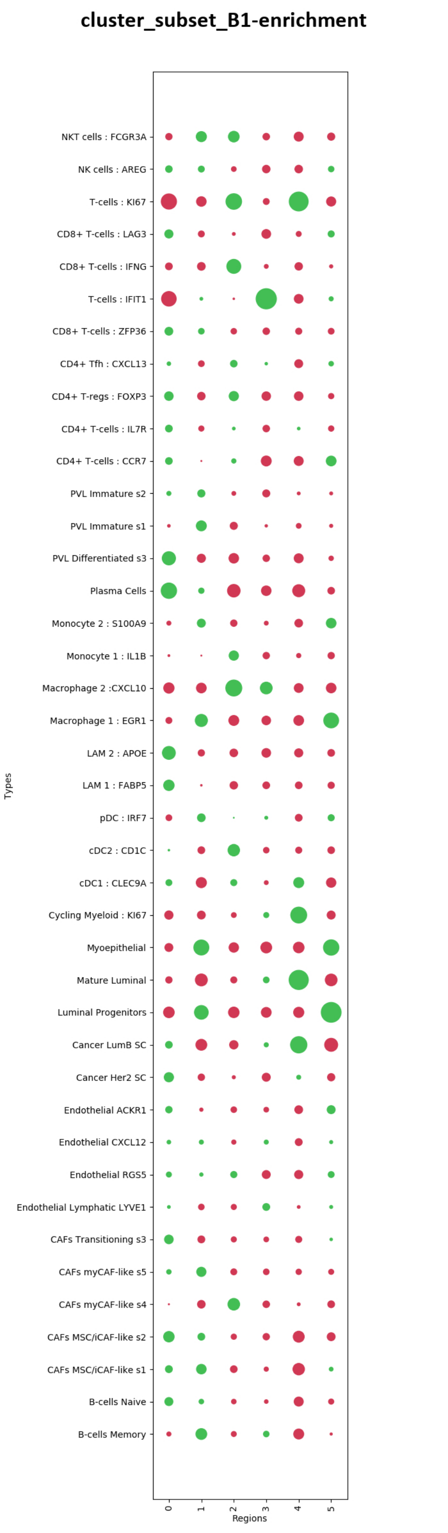

cluster_subset_B1-enrichment
Types
Regions
NKT cells : FCGR3A
NK cells : AREG
T-cells : KI67
CD8+ T-cells : LAG3
CD8+ T-cells : IFNG
T-cells : IFIT1
CD8+ T-cells : ZFP36
CD4+ Tfh : CXCL13
CD4+ T-regs : FOXP3
CD4+ T-cells : IL7R
CD4+ T-cells : CCR7
PVL Immature s2
PVL Immature s1
PVL Differentiated s3
Plasma Cells
Monocyte 2 : S100A9
Monocyte 1 : IL1B
Macrophage 2 :CXCL10
Macrophage 1 : EGR1
LAM 2 : APOE
LAM 1 : FABP5
pDC : IRF7
cDC2 : CD1C
cDC1 : CLEC9A
Cycling Myeloid : KI67
Myoepithelial
Mature Luminal
Luminal Progenitors
Cancer LumB SC
Cancer Her2 SC
Endothelial ACKR1
Endothelial CXCL12
Endothelial RGS5
Endothelial Lymphatic LYVE1
CAFs Transitioning s3
CAFs myCAF-like s5
CAFs myCAF-like s4
CAFs MSC/iCAF-like s2
CAFs MSC/iCAF-like s1
B-cells Naive
B-cells Memory
0
1
2
3
4
5

cluster_subset_C2-enrichment

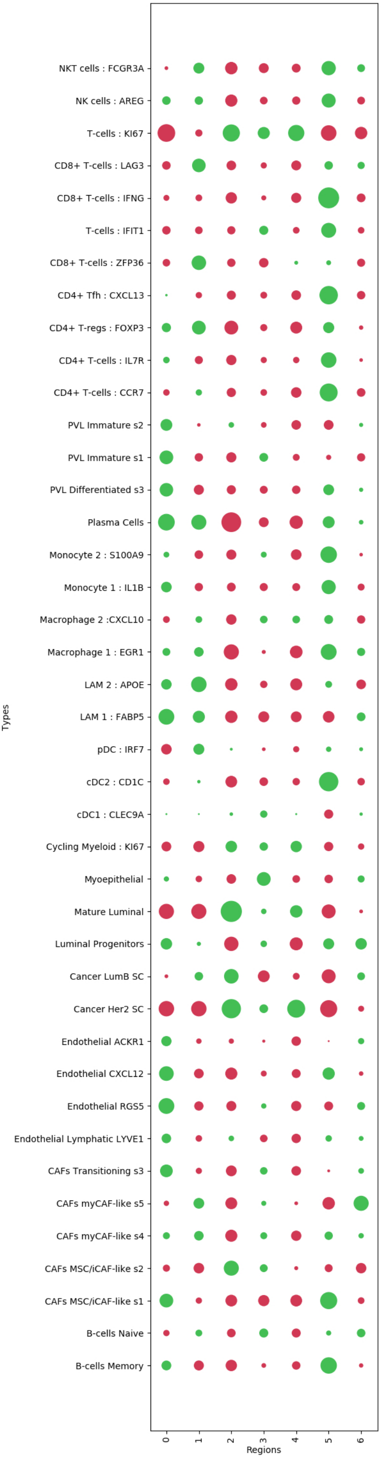

cluster_subset_A4-enrichment

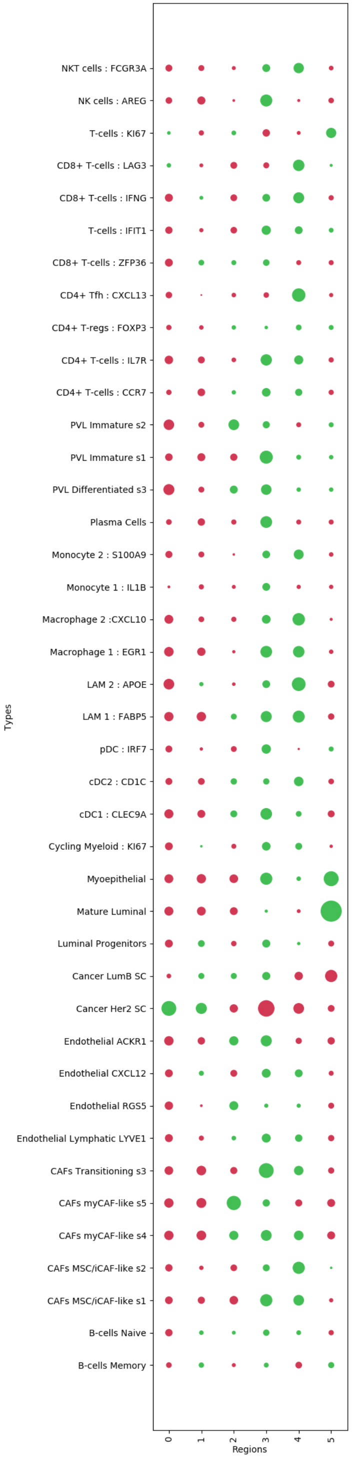

cluster_subset_F1-enrichment

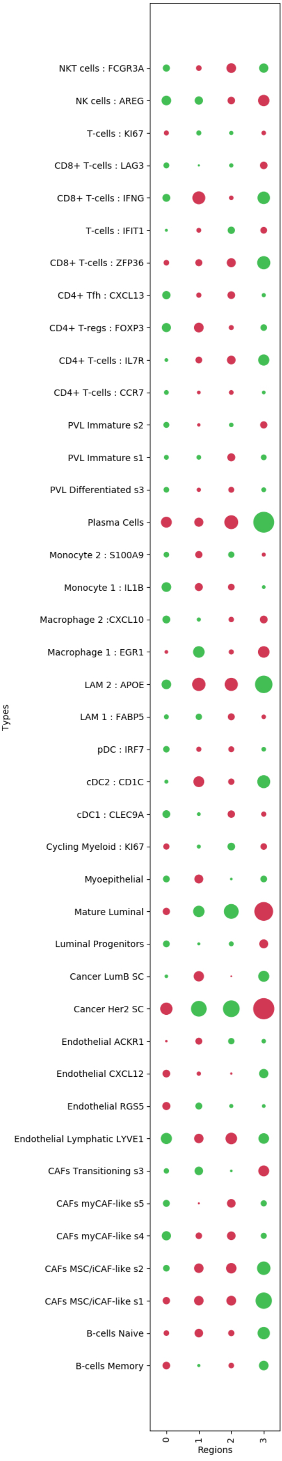

cluster_subset_D6-enrichment

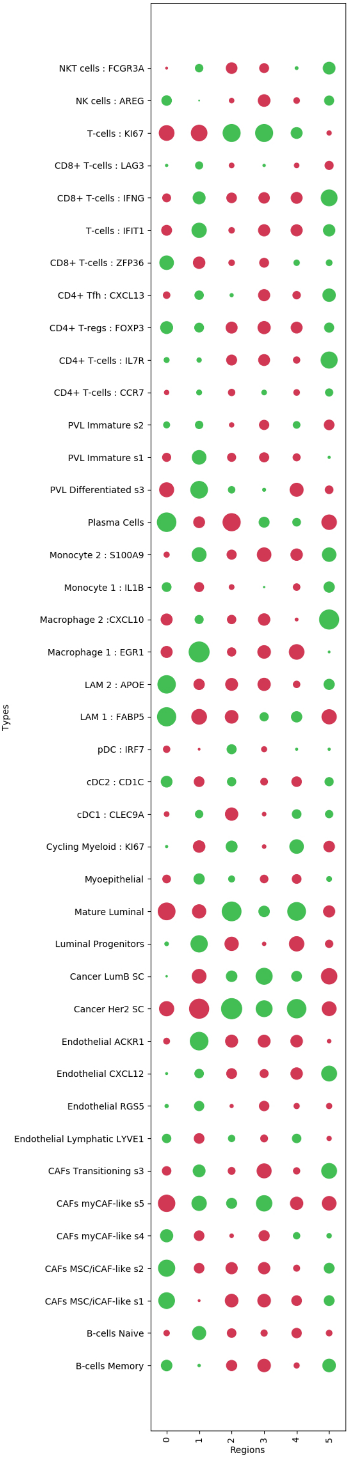

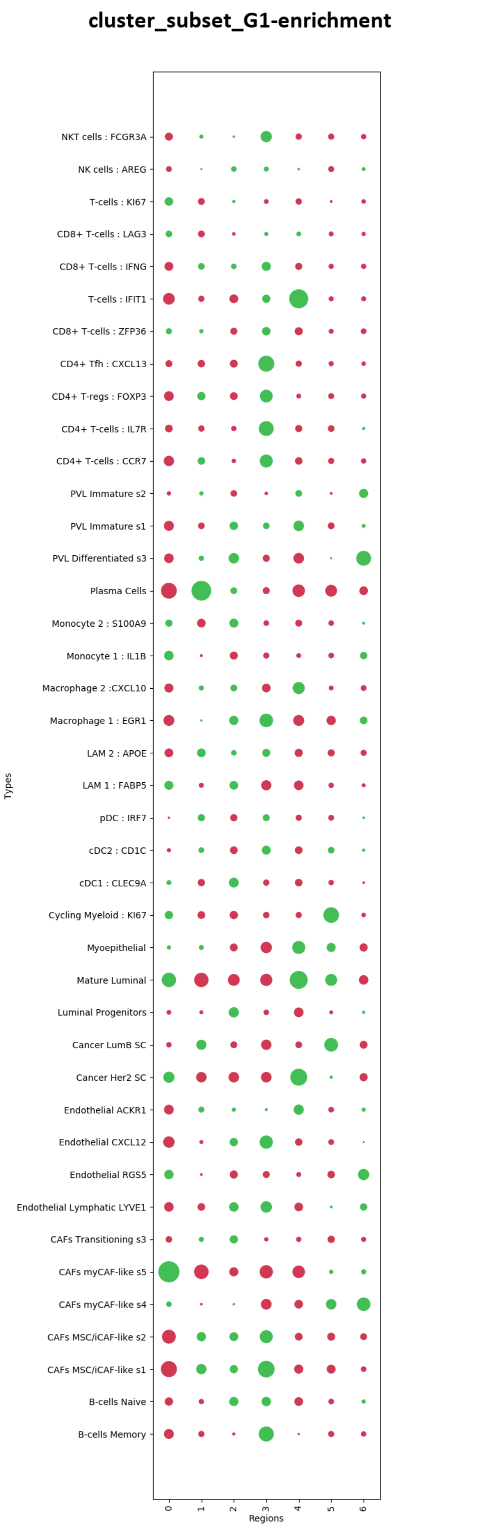
cluster_subset_G1-enrichment
NKT cells : FCGR3A
NK cells : AREG
T-cells : KI67
CD8+ T-cells : LAG3
CD8+ T-cells : IFNG
T-cells : IFIT1
CD8+ T-cells : ZFP36
CD4+ Tfh : CXCL13
CD4+ T-regs : FOXP3
CD4+ T-cells : IL7R
CD4+ T-cells : CCR7
PVL Immature s2
PVL Immature s1
PVL Differentiated s3
Plasma Cells
Monocyte 2 : S100A9
Monocyte 1 : IL1B
Macrophage 2 :CXCL10
Macrophage 1 : EGR1
LAM 2 : APOE
LAM 1 : FABP5
pDC : IRF7
cDC2 : CD1C
cDC1 : CLEC9A
Cycling Myeloid : KI67
Myoepithelial
Mature Luminal
Luminal Progenitors
Cancer LumB SC
Cancer Her2 SC
Endothelial ACKR1
Endothelial CXCL12
Endothelial RGS5
Endothelial Lymphatic LYVE1
CAFs Transitioning s3
CAFs myCAF-like s5
CAFs myCAF-like s4
CAFs MSC/iCAF-like s2
CAFs MSC/iCAF-like s1
B-cells Naive
B-cells Memory
Types
0
1
2
3
4
5
6
Regions

cluster_subset_C5-enrichment

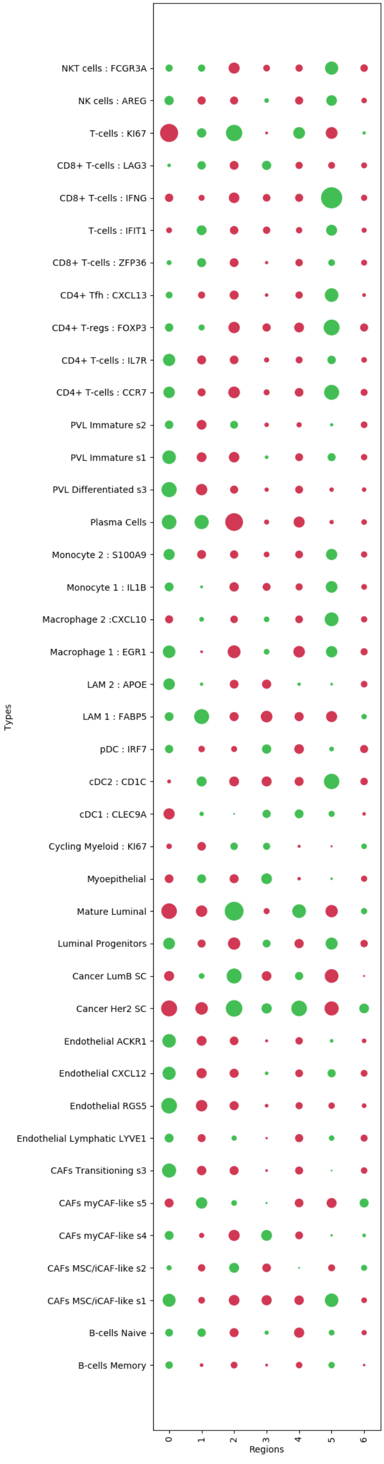

cluster_subset_C1-enrichment

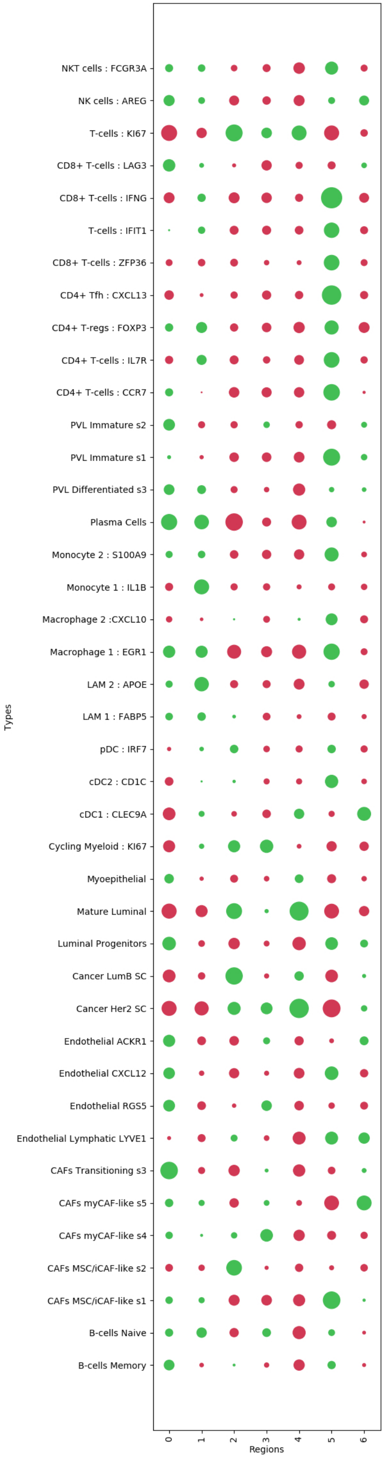

cluster_subset_B6-enrichment

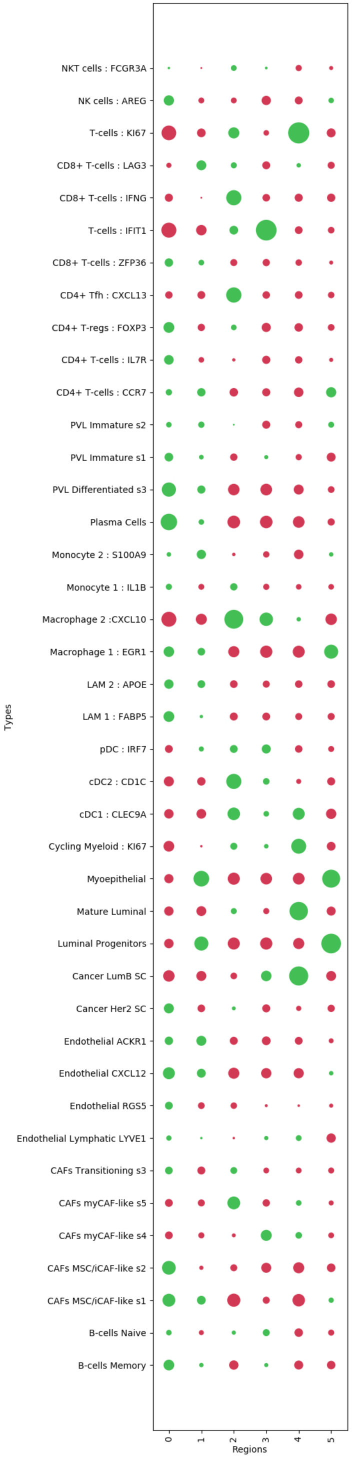

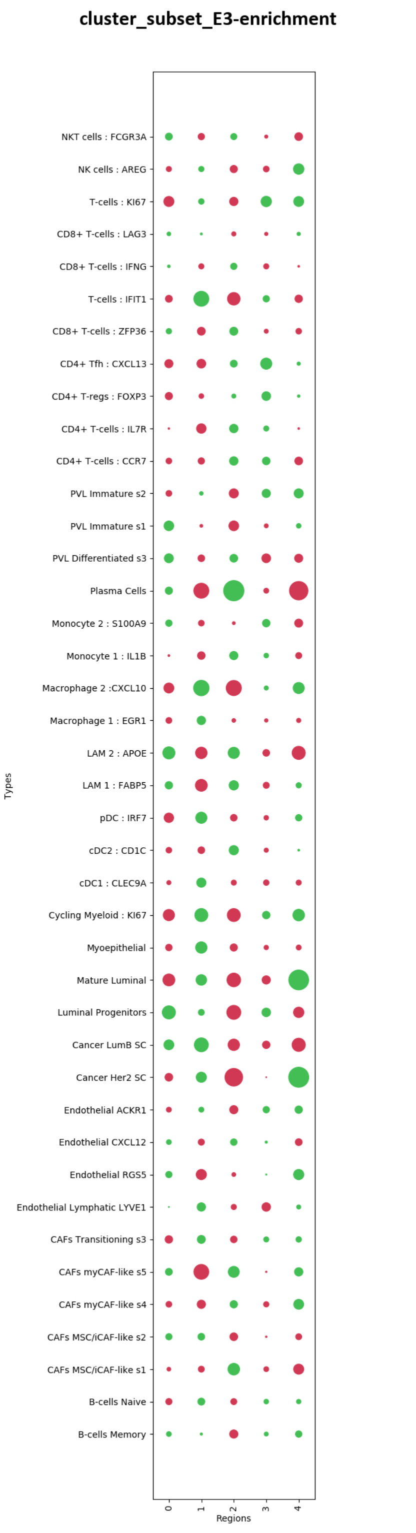
cluster_subset_E3-enrichment
NKT cells : FCGR3A
NK cells : AREG
T-cells : KI67
CD8+ T-cells : LAG3
CD8+ T-cells : IFNG
T-cells : IFIT1
CD8+ T-cells : ZFP36
CD4+ Tfh : CXCL13
CD4+ T-regs : FOXP3
CD4+ T-cells : IL7R
CD4+ T-cells : CCR7
PVL Immature s2
PVL Immature s1
PVL Differentiated s3
Plasma Cells
Monocyte 2 : S100A9
Monocyte 1 : IL1B
Macrophage 2 :CXCL10
Macrophage 1 : EGR1
LAM 2 : APOE
LAM 1 : FABP5
pDC : IRF7
cDC2 : CD1C
cDC1 : CLEC9A
Cycling Myeloid : KI67
Myoepithelial
Mature Luminal
Luminal Progenitors
Cancer LumB SC
Cancer Her2 SC
Endothelial ACKR1
Endothelial CXCL12
Endothelial RGS5
Endothelial Lymphatic LYVE1
CAFs Transitioning s3
CAFs myCAF-like s5
CAFs myCAF-like s4
CAFs MSC/iCAF-like s2
CAFs MSC/iCAF-like s1
B-cells Naive
B-cells Memory
Types
0
1
2
3
4
Regions

cluster_subset_D2-enrichment

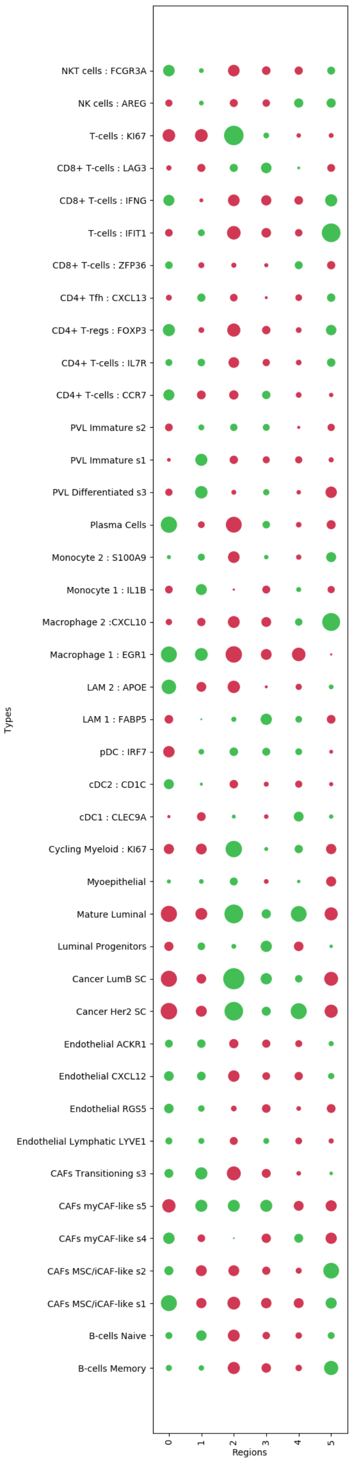

cluster_subset_D3-enrichment

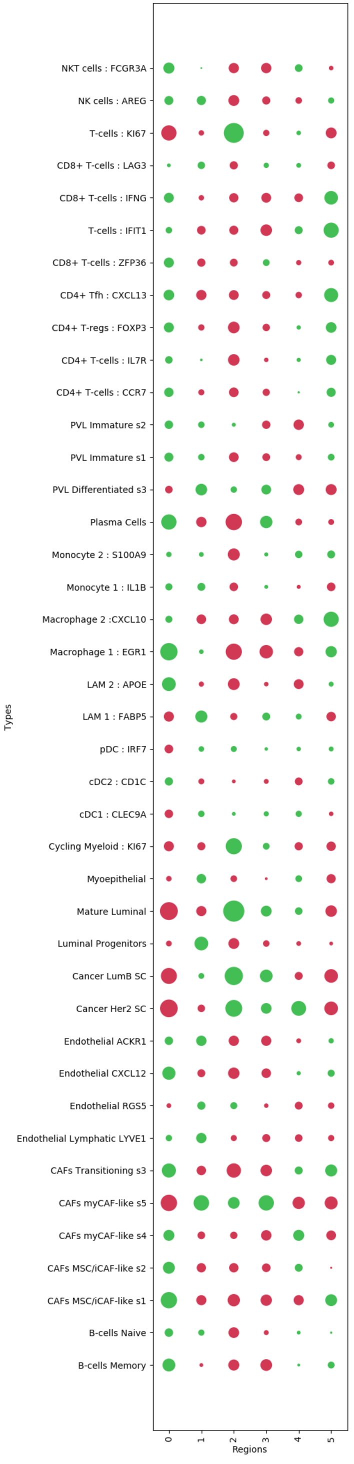

cluster_subset_G2-enrichment

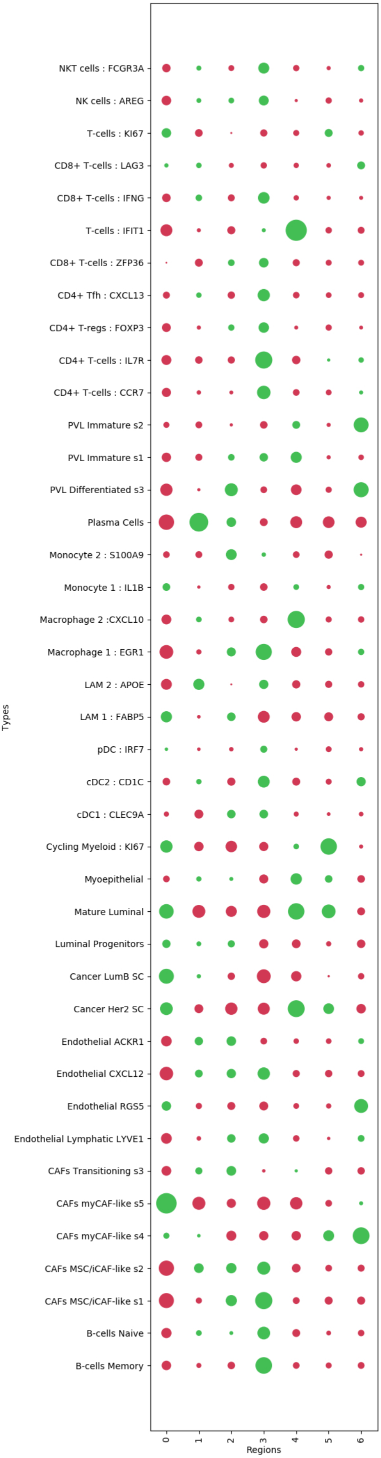

cluster_subset_B3-enrichment

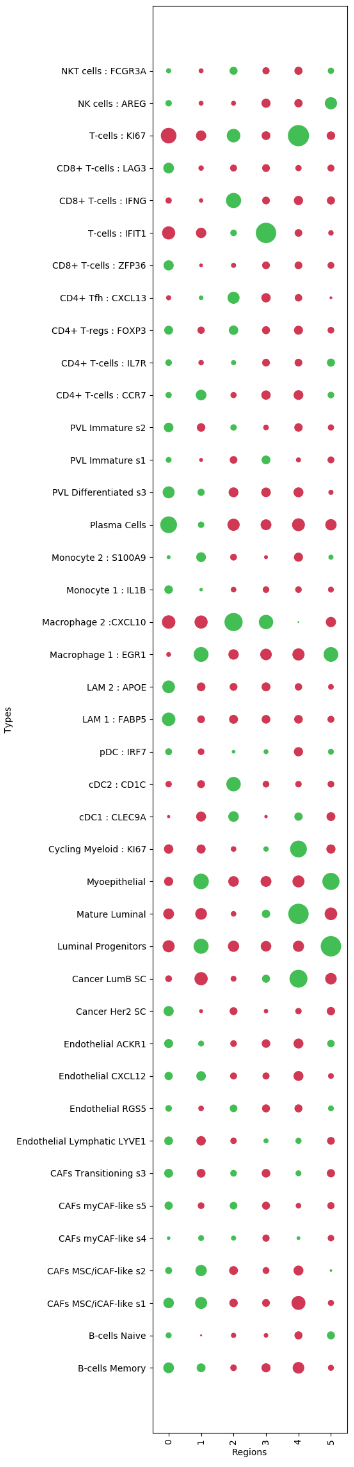

cluster_subset_F2-enrichment

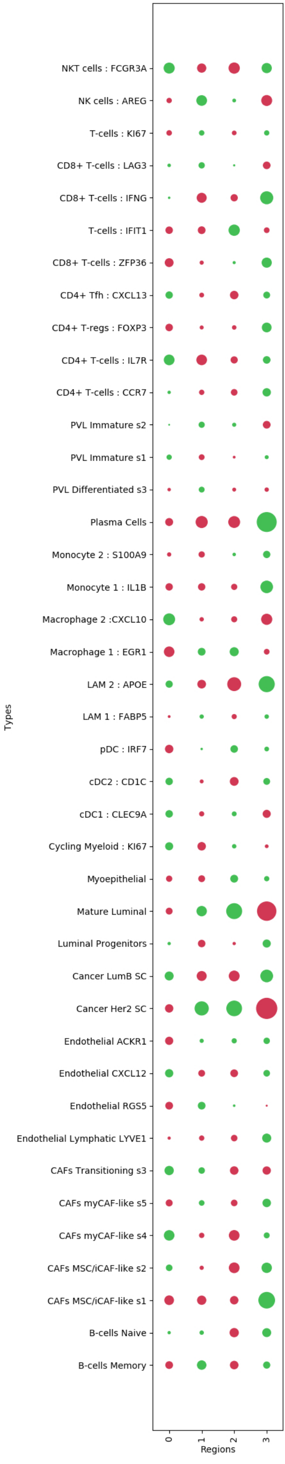

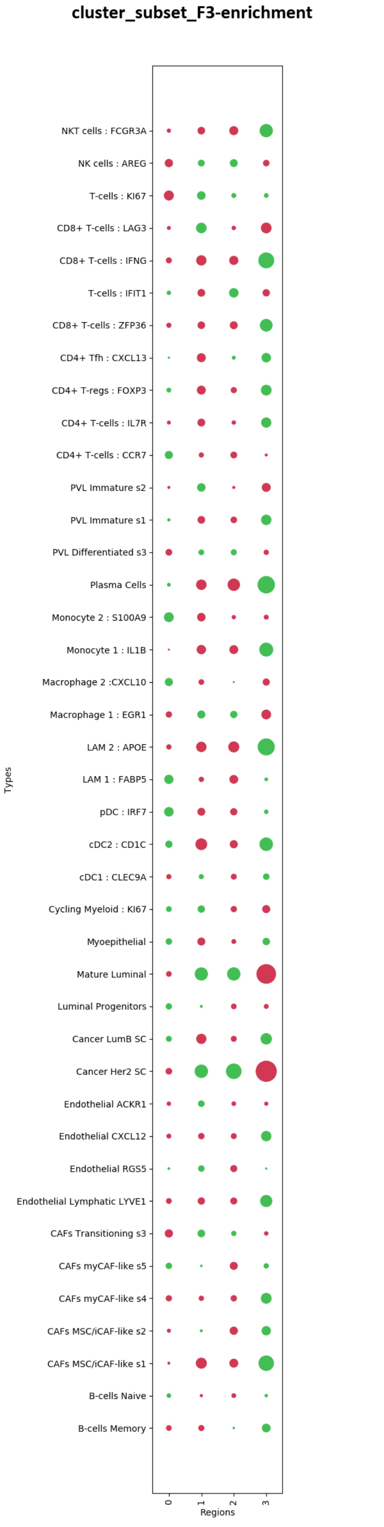
cluster_subset_F3-enrichment
NKT cells : FCGR3A
NK cells : AREG
T-cells : KI67
CD8+ T-cells : LAG3
CD8+ T-cells : IFNG
T-cells : IFIT1
CD8+ T-cells : ZFP36
CD4+ Tfh : CXCL13
CD4+ T-regs : FOXP3
CD4+ T-cells : IL7R
CD4+ T-cells : CCR7
PVL Immature s2
PVL Immature s1
PVL Differentiated s3
Plasma Cells
Monocyte 2 : S100A9
Monocyte 1 : IL1B
Macrophage 2 :CXCL10
Macrophage 1 : EGR1
LAM 2 : APOE
LAM 1 : FABP5
pDC : IRF7
cDC2 : CD1C
cDC1 : CLEC9A
Cycling Myeloid : KI67
Myoepithelial
Mature Luminal
Luminal Progenitors
Cancer LumB SC
Cancer Her2 SC
Endothelial ACKR1
Endothelial CXCL12
Endothelial RGS5
Endothelial Lymphatic LYVE1
CAFs Transitioning s3
CAFs myCAF-like s5
CAFs myCAF-like s4
CAFs MSC/iCAF-like s2
CAFs MSC/iCAF-like s1
B-cells Naive
B-cells Memory
Types
0
1
2
3
Regions

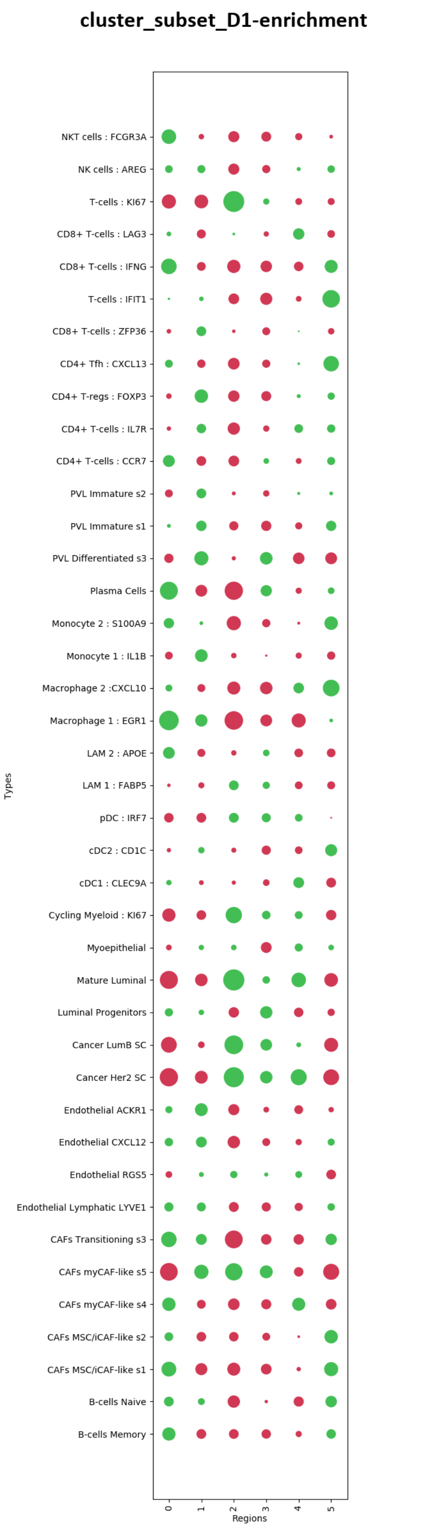
cluster_subset_D1-enrichment
NKT cells : FCGR3A
NK cells : AREG
T-cells : KI67
CD8+ T-cells : LAG3
CD8+ T-cells : IFNG
T-cells : IFIT1
CD8+ T-cells : ZFP36
CD4+ Tfh : CXCL13
CD4+ T-regs : FOXP3
CD4+ T-cells : IL7R
CD4+ T-cells : CCR7
PVL Immature s2
PVL Immature s1
PVL Differentiated s3
Plasma Cells
Monocyte 2 : S100A9
Monocyte 1 : IL1B
Macrophage 2 :CXCL10
Macrophage 1 : EGR1
LAM 2 : APOE
LAM 1 : FABP5
pDC : IRF7
cDC2 : CD1C
cDC1 : CLEC9A
Cycling Myeloid : KI67
Myoepithelial
Mature Luminal
Luminal Progenitors
Cancer LumB SC
Cancer Her2 SC
Endothelial ACKR1
Endothelial CXCL12
Endothelial RGS5
Endothelial Lymphatic LYVE1
CAFs Transitioning s3
CAFs myCAF-like s5
CAFs myCAF-like s4
CAFs MSC/iCAF-like s2
CAFs MSC/iCAF-like s1
B-cells Naive
B-cells Memory
Types
0
1
2
3
4
5
Regions

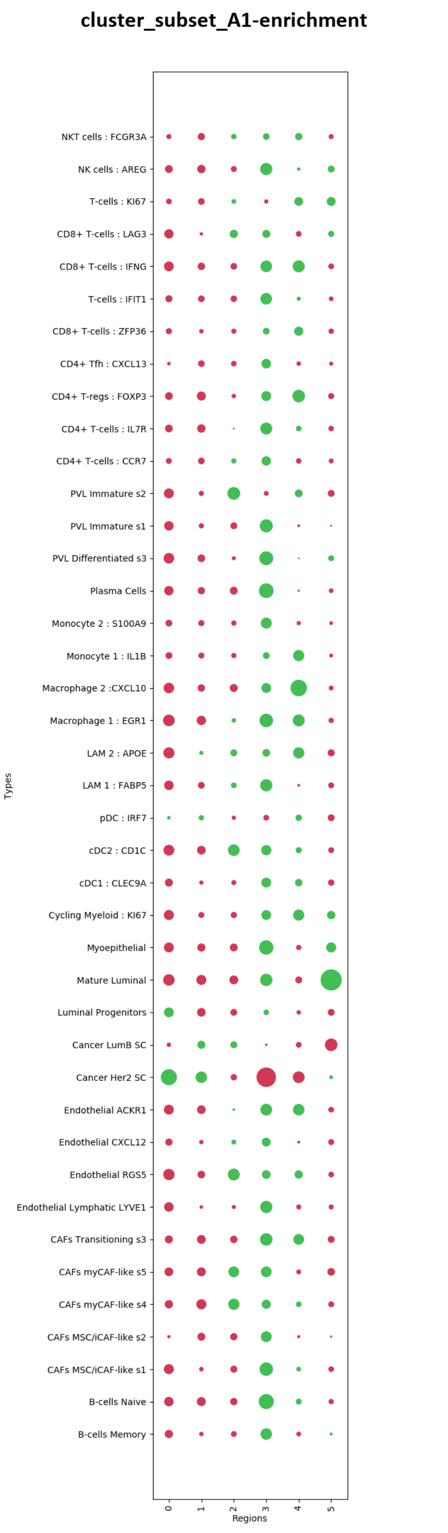
cluster_subset_A1-enrichment
NKT cells : FCGR3A
NK cells : AREG
T-cells : KI67
CD8+ T-cells : LAG3
CD8+ T-cells : IFNG
T-cells : IFIT1
CD8+ T-cells : ZFP36
CD4+ Tfh : CXCL13
CD4+ T-regs : FOXP3
CD4+ T-cells : IL7R
CD4+ T-cells : CCR7
PVL Immature s2
PVL Immature s1
PVL Differentiated s3
Plasma Cells
Monocyte 2 : S100A9
Monocyte 1 : IL1B
Macrophage 2 :CXCL10
Macrophage 1 : EGR1
LAM 2 : APOE
LAM 1 : FABP5
pDC : IRF7
cDC2 : CD1C
cDC1 : CLEC9A
Cycling Myeloid : KI67
Myoepithelial
Mature Luminal
Luminal Progenitors
Cancer LumB SC
Cancer Her2 SC
Endothelial ACKR1
Endothelial CXCL12
Endothelial RGS5
Endothelial Lymphatic LYVE1
CAFs Transitioning s3
CAFs myCAF-like s5
CAFs myCAF-like s4
CAFs MSC/iCAF-like s2
CAFs MSC/iCAF-like s1
B-cells Naive
B-cells Memory
Types
0
1
2
3
4
5
Regions

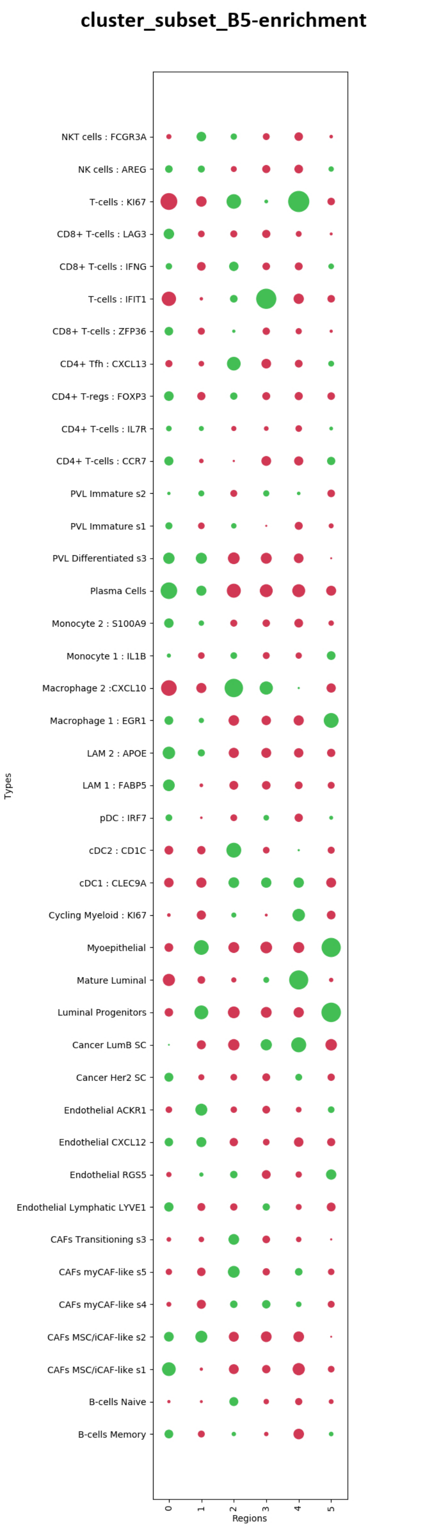

cluster_subset_B5-enrichment
NKT cells : FCGR3A
NK cells : AREG
T-cells : KI67
CD8+ T-cells : LAG3
CD8+ T-cells : IFNG
T-cells : IFIT1
CD8+ T-cells : ZFP36
CD4+ Tfh : CXCL13
CD4+ T-regs : FOXP3
CD4+ T-cells : IL7R
CD4+ T-cells : CCR7
PVL Immature s2
PVL Immature s1
PVL Differentiated s3
Plasma Cells
Monocyte 2 : S100A9
Monocyte 1 : IL1B
Macrophage 2 :CXCL10
Macrophage 1 : EGR1
LAM 2 : APOE
LAM 1 : FABP5
pDC : IRF7
cDC2 : CD1C
cDC1 : CLEC9A
Cycling Myeloid : KI67
Myoepithelial
Mature Luminal
Luminal Progenitors
Cancer LumB SC
Cancer Her2 SC
Endothelial ACKR1
Endothelial CXCL12
Endothelial RGS5
Endothelial Lymphatic LYVE1
CAFs Transitioning s3
CAFs myCAF-like s5
CAFs myCAF-like s4
CAFs MSC/iCAF-like s2
CAFs MSC/iCAF-like s1
B-cells Naive
B-cells Memory
Types
0
1
2
3
4
5
Regions

cluster_subset_E2-enrichment

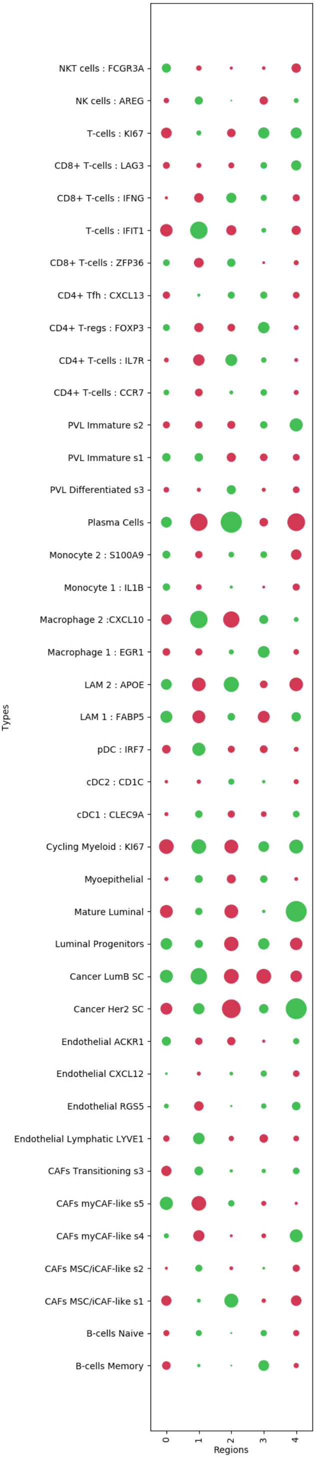

cluster_subset_A5-enrichment

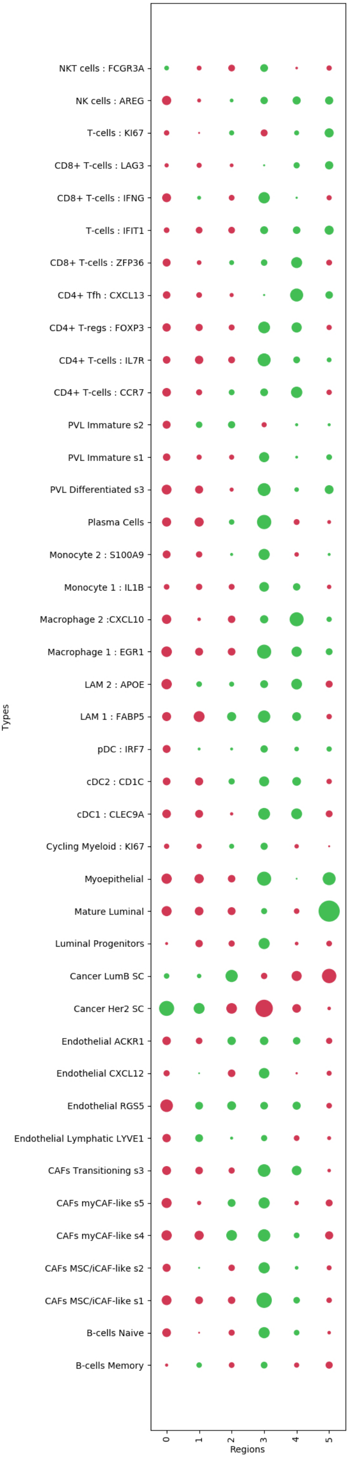

cluster_subset_G3-enrichment

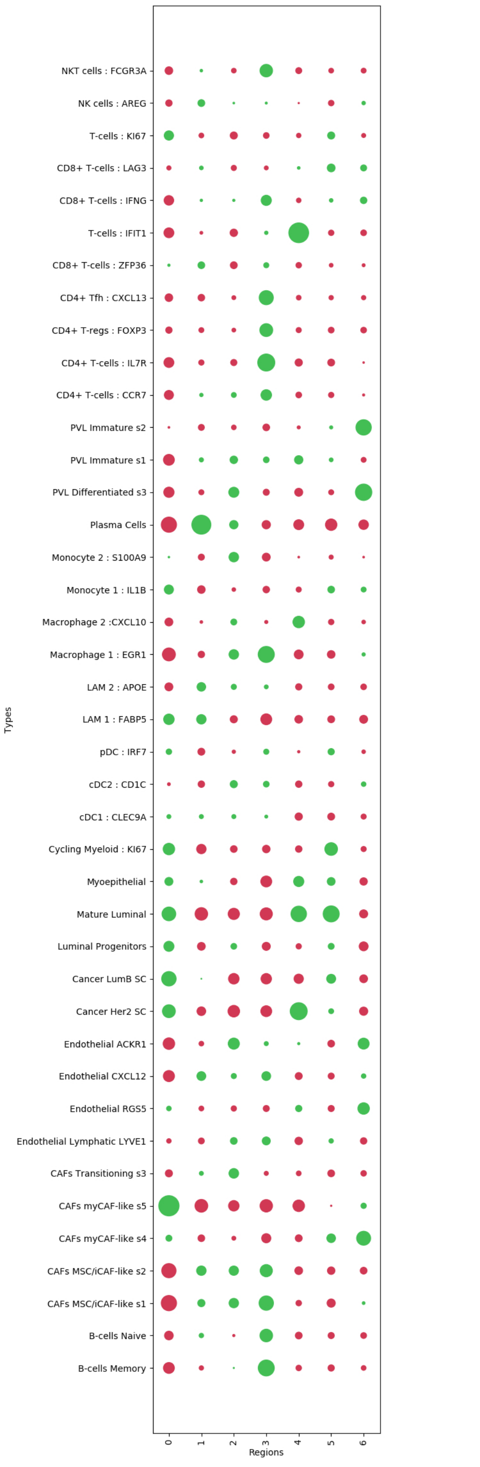

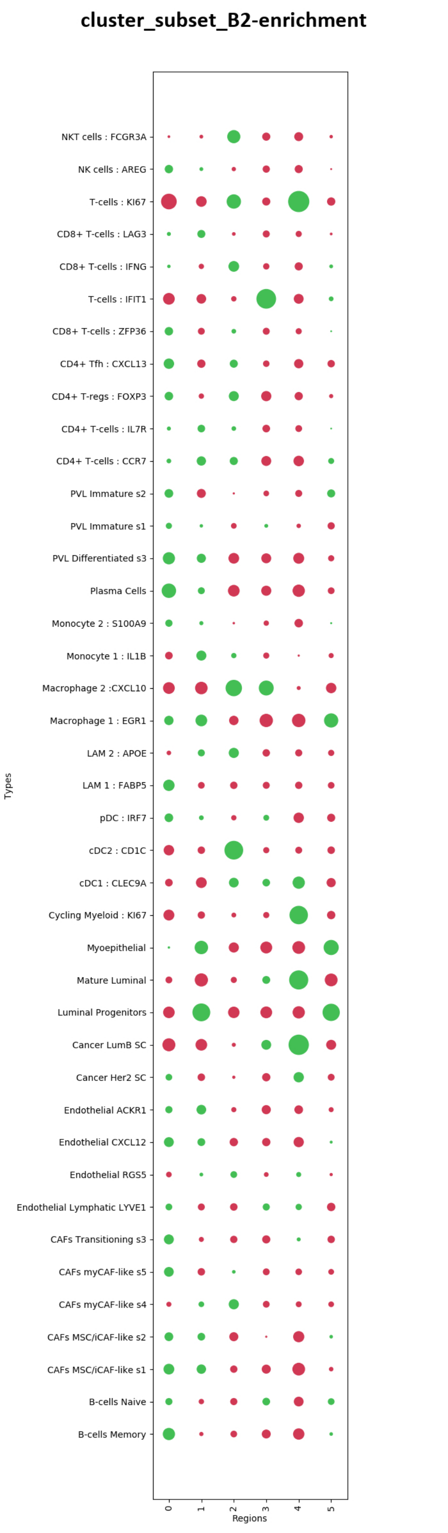

cluster_subset_B2-enrichment
NKT cells : FCGR3A
NK cells : AREG
T-cells : KI67
CD8+ T-cells : LAG3
CD8+ T-cells : IFNG
T-cells : IFIT1
CD8+ T-cells : ZFP36
CD4+ Tfh : CXCL13
CD4+ T-regs : FOXP3
CD4+ T-cells : IL7R
CD4+ T-cells : CCR7
PVL Immature s2
PVL Immature s1
PVL Differentiated s3
Plasma Cells
Monocyte 2 : S100A9
Monocyte 1 : IL1B
Macrophage 2 :CXCL10
Macrophage 1 : EGR1
LAM 2 : APOE
LAM 1 : FABP5
pDC : IRF7
cDC2 : CD1C
cDC1 : CLEC9A
Cycling Myeloid : KI67
Myoepithelial
Mature Luminal
Luminal Progenitors
Cancer LumB SC
Cancer Her2 SC
Endothelial ACKR1
Endothelial CXCL12
Endothelial RGS5
Endothelial Lymphatic LYVE1
CAFs Transitioning s3
CAFs myCAF-like s5
CAFs myCAF-like s4
CAFs MSC/iCAF-like s2
CAFs MSC/iCAF-like s1
B-cells Naive
B-cells Memory
Types
0
1
2
3
4
5
Regions

cluster_subset_H2-enrichment

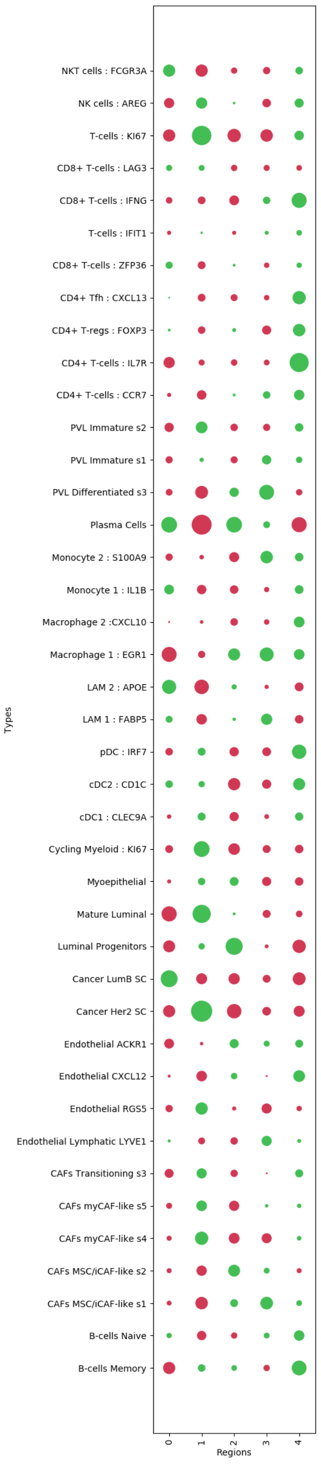

cluster_subset_E1-enrichment

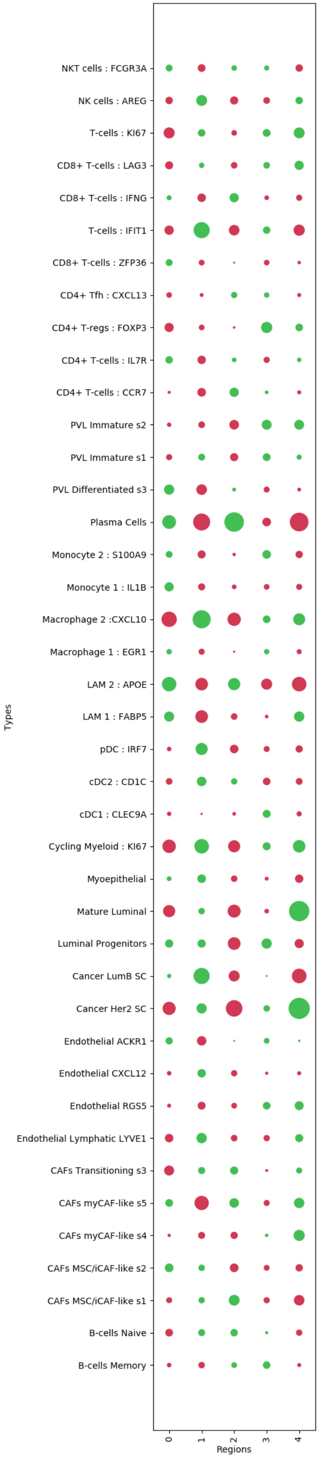

cluster_subset_A2-enrichment

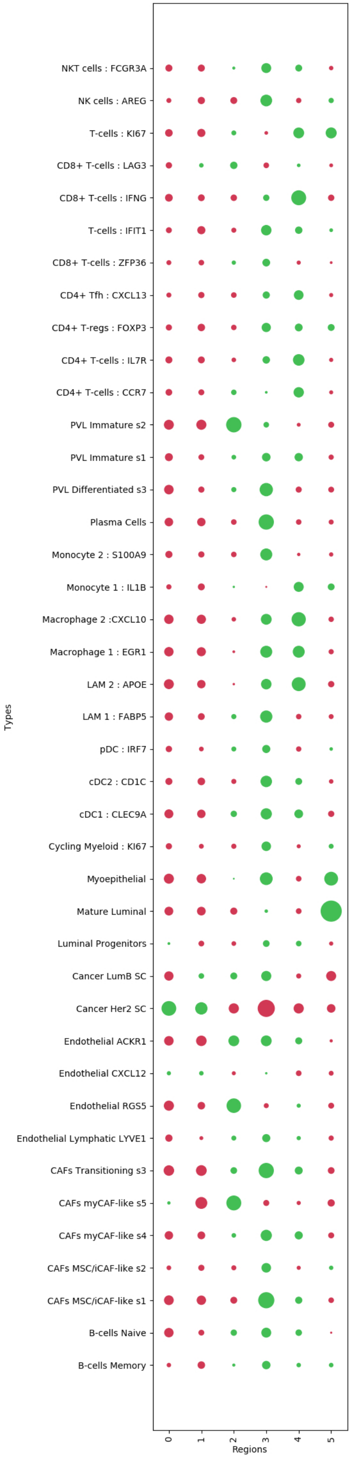

cluster_subset_A6-enrichment

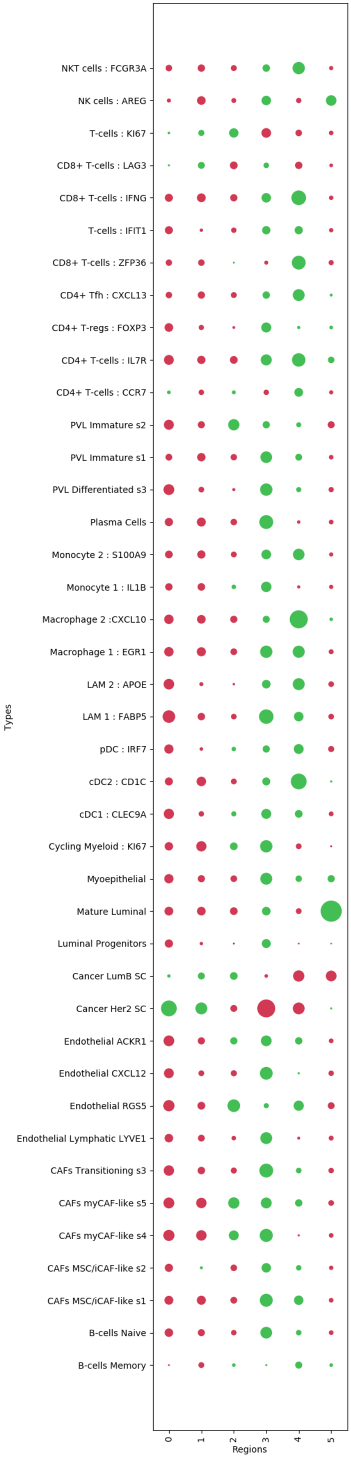

cluster_subset_D4-enrichment

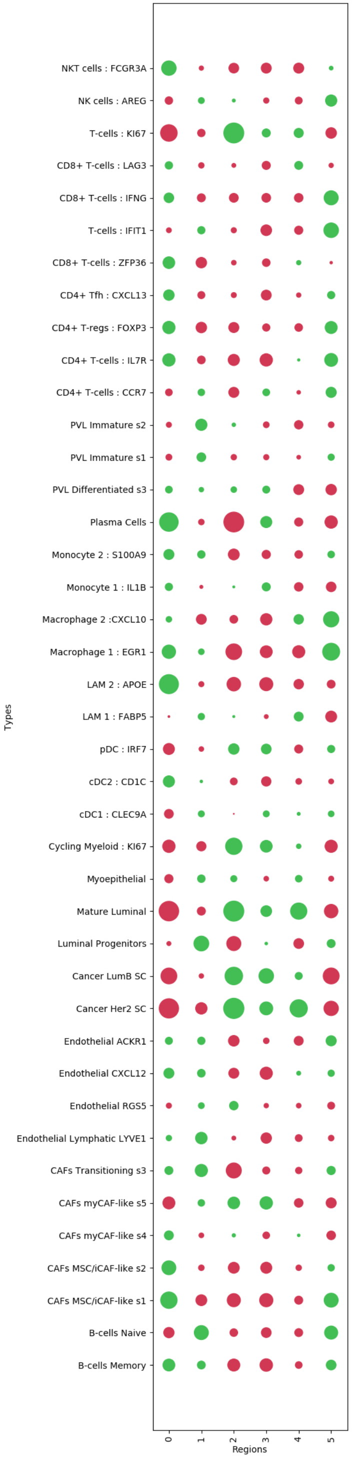

cluster_subset_D5-enrichment

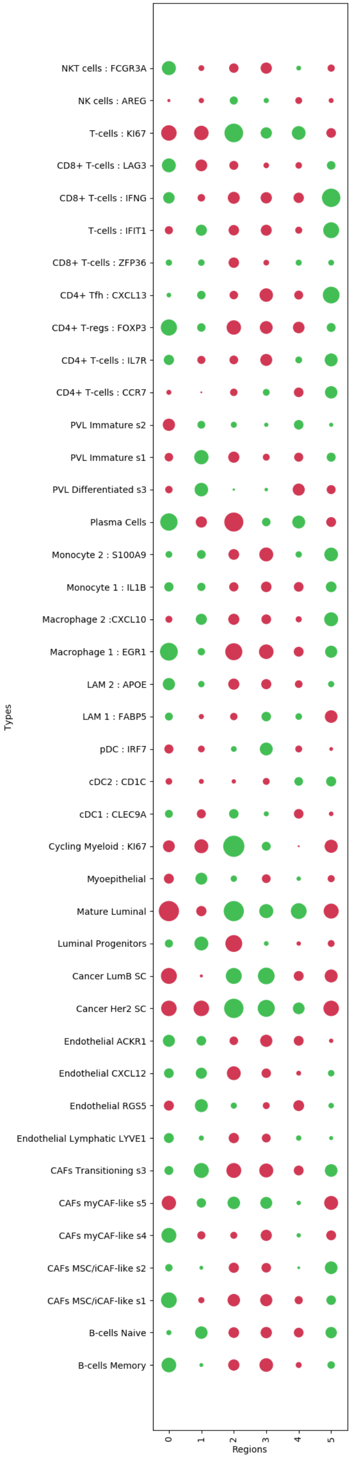

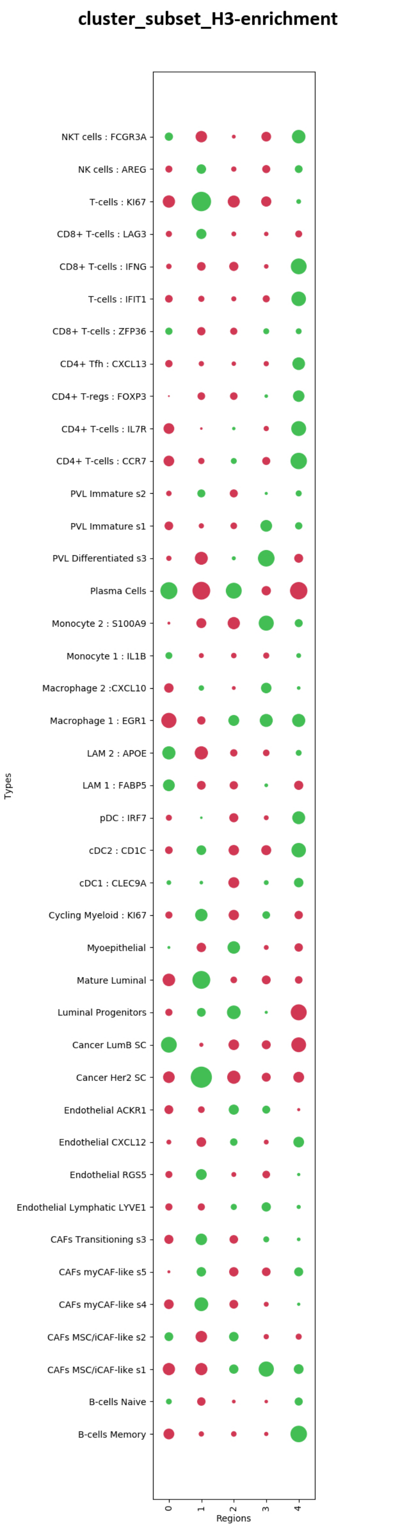
cluster_subset_H3-enrichment
Types
NKT cells : FCGR3A
NK cells : AREG
T-cells : KI67
CD8+ T-cells : LAG3
CD8+ T-cells : IFNG
T-cells : IFIT1
CD8+ T-cells : ZFP36
CD4+ Tfh : CXCL13
CD4+ T-regs : FOXP3
CD4+ T-cells : IL7R
CD4+ T-cells : CCR7
PVL Immature s2
PVL Immature s1
PVL Differentiated s3
Plasma Cells
Monocyte 2 : S100A9
Monocyte 1 : IL1B
Macrophage 2 :CXCL10
Macrophage 1 : EGR1
LAM 2 : APOE
LAM 1 : FABP5
pDC : IRF7
cDC2 : CD1C
cDC1 : CLEC9A
Cycling Myeloid : KI67
Myoepithelial
Mature Luminal
Luminal Progenitors
Cancer LumB SC
Cancer Her2 SC
Endothelial ACKR1
Endothelial CXCL12
Endothelial RGS5
Endothelial Lymphatic LYVE1
CAFs Transitioning s3
CAFs myCAF-like s5
CAFs myCAF-like s4
CAFs MSC/iCAF-like s2
CAFs MSC/iCAF-like s1
B-cells Naive
B-cells Memory
0
1
2
3
4
Regions

cluster_major_C6-enrichment

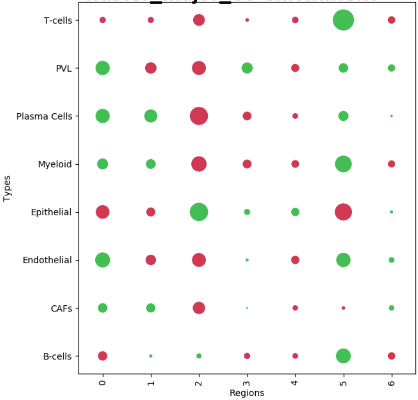

cluster_major_C4-enrichment

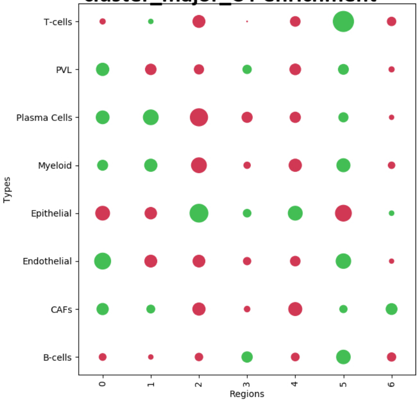

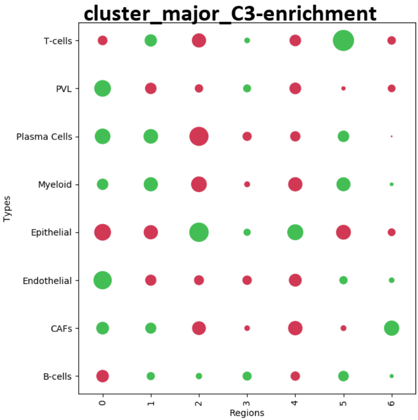
cluster_major_C3-enrichment
T-cells
PVL
Plasma Cells
Myeloid
Epithelial
Endothelial
CAFs
B-cells
Types
0
1
2
3
4
5
6
Regions

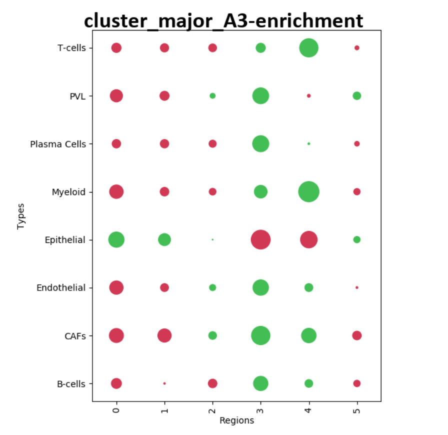

cluster_major_A3-enrichment
T-cells
PVL
Plasma Cells
Myeloid
Epithelial
Endothelial
CAFs
B-cells
Types
0
1
2
3
4
5
Regions

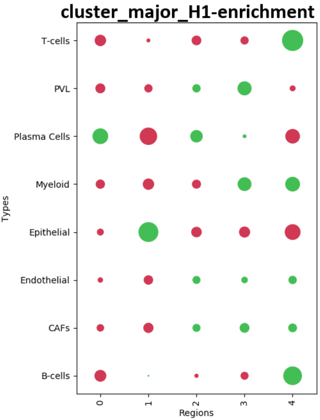
cluster_major_H1-enrichment
T-cells
PVL
Plasma Cells
Myeloid
Epithelial
Endothelial
CAFs
B-cells
Types
0
1
2
3
4
Regions

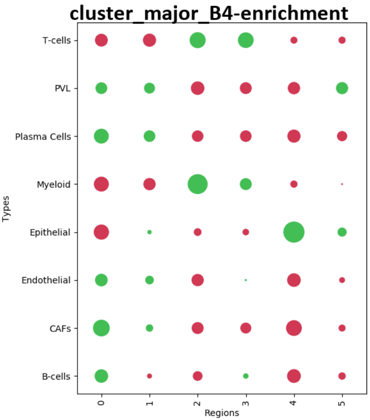

cluster_major_B4-enrichment
T-cells
PVL
Plasma Cells
Myeloid
Epithelial
Endothelial
CAFs
B-cells
Types
0
1
2
3
4
5
Regions

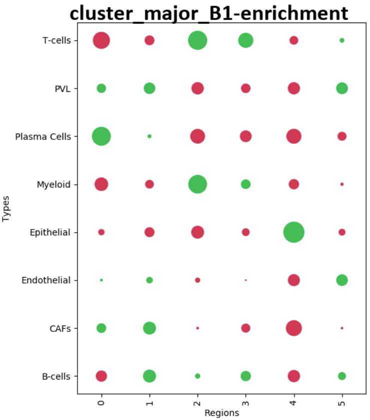

cluster_major_B1-enrichment
T-cells
PVL
Plasma Cells
Myeloid
Epithelial
Endothelial
CAFs
B-cells
Types
0
1
2
3
4
5
Regions

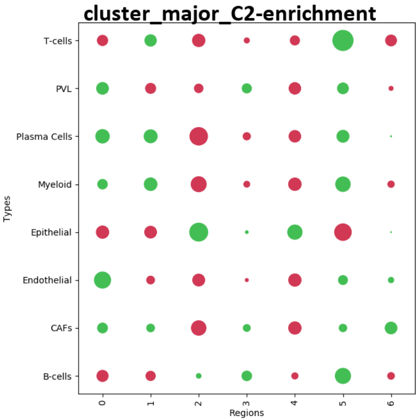
cluster_major_C2-enrichment
T-cells
PVL
Plasma Cells
Myeloid
Epithelial
Endothelial
CAFs
B-cells
Types
0
1
2
3
4
5
6
Regions

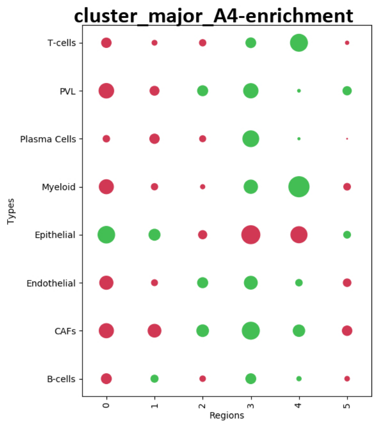
cluster_major_A4-enrichment
T-cells
PVL
Plasma Cells
Myeloid
Epithelial
Endothelial
CAFs
B-cells
Types
0
1
2
3
4
5
Regions

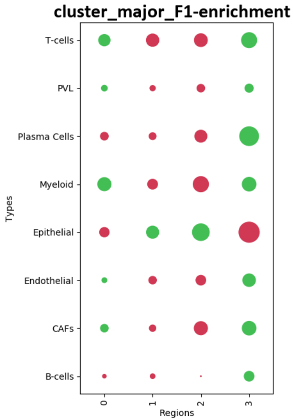

cluster_major_F1-enrichment
T-cells
PVL
Plasma Cells
Myeloid
Epithelial
Endothelial
CAFs
B-cells
Types
0
1
2
3
Regions

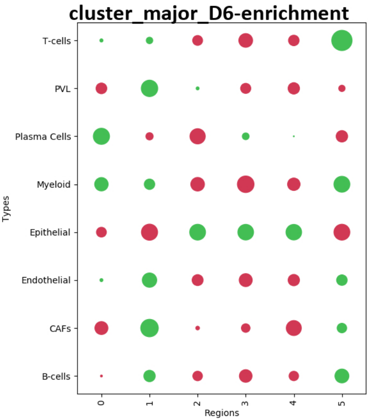
cluster_major_D6-enrichment
T-cells
PVL
Plasma Cells
Myeloid
Epithelial
Endothelial
CAFs
B-cells
Types
0
1
2
3
4
5
Regions

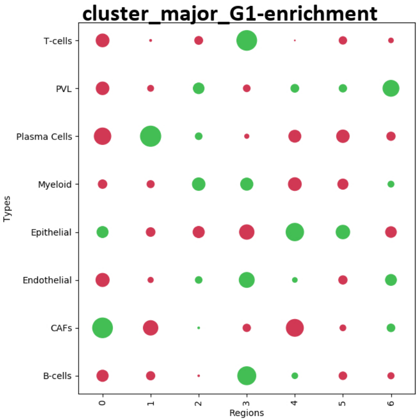
cluster_major_G1-enrichment
T-cells
PVL
Plasma Cells
Myeloid
Epithelial
Endothelial
CAFs
B-cells
Types
0
1
2
3
4
5
6
Regions

cluster_major_C5-enrichment

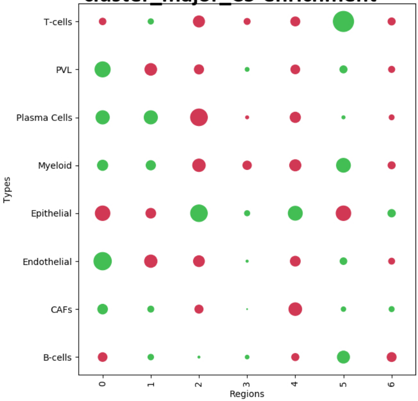

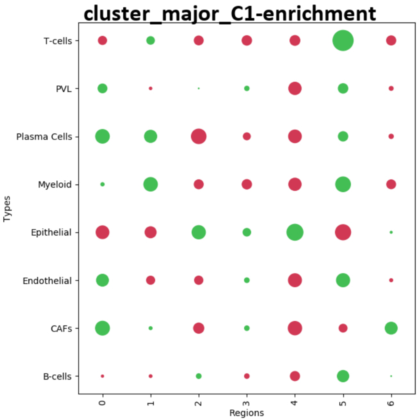

cluster_major_C1-enrichment
T-cells
PVL
Plasma Cells
Myeloid
Epithelial
Endothelial
CAFs
B-cells
Types
0
1
2
3
4
5
6
Regions

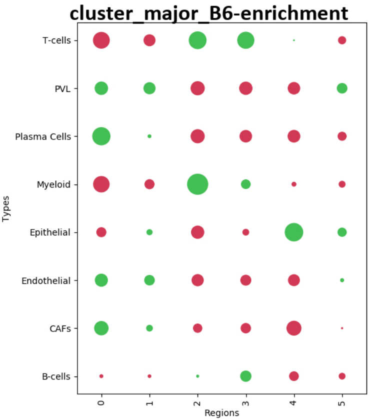
cluster_major_B6-enrichment
T-cells
PVL
Plasma Cells
Myeloid
Epithelial
Endothelial
CAFs
B-cells
Types
0
1
2
3
4
5
Regions

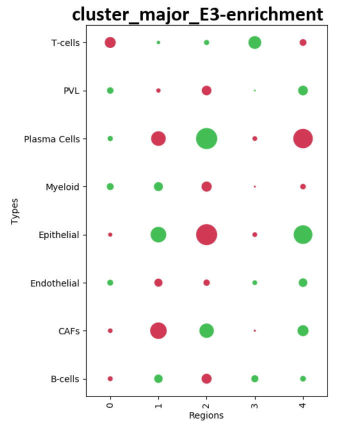
cluster_major_E3-enrichment
T-cells
PVL
Plasma Cells
Myeloid
Epithelial
Endothelial
CAFs
B-cells
Types
0
1
2
3
4
Regions

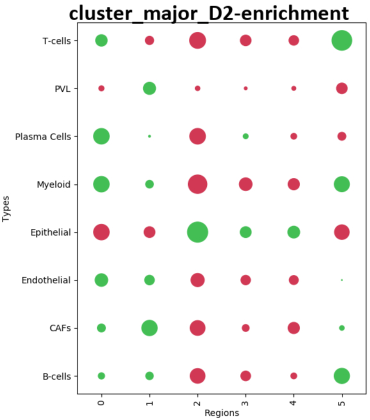
cluster_major_D2-enrichment
T-cells
PVL
Plasma Cells
Myeloid
Epithelial
Endothelial
CAFs
B-cells
Types
0
1
2
3
4
5
Regions

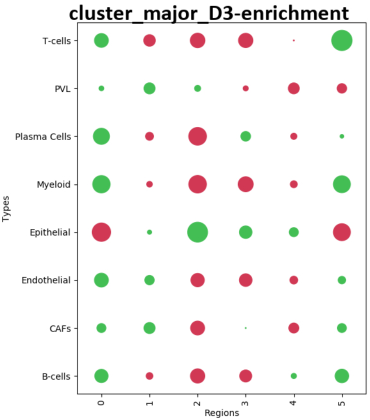

cluster_major_D3-enrichment
T-cells
PVL
Plasma Cells
Myeloid
Epithelial
Endothelial
CAFs
B-cells
Types
0
1
2
3
4
5
Regions

cluster_major_G2-enrichment

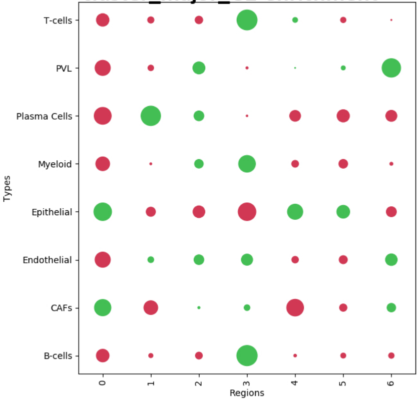

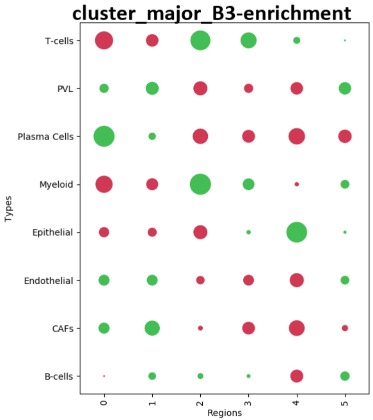

cluster_major_B3-enrichment
T-cells
PVL
Plasma Cells
Myeloid
Epithelial
Endothelial
CAFs
B-cells
Types
0
1
2
3
4
5
Regions

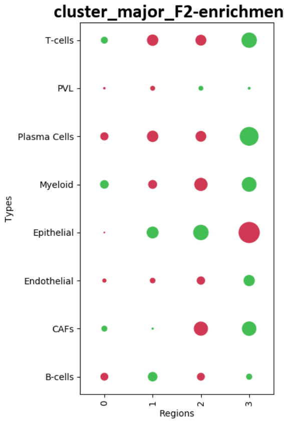
cluster_major_F2-enrichment
T-cells
PVL
Plasma Cells
Myeloid
Epithelial
Endothelial
CAFs
B-cells
Types
0
1
2
3
Regions

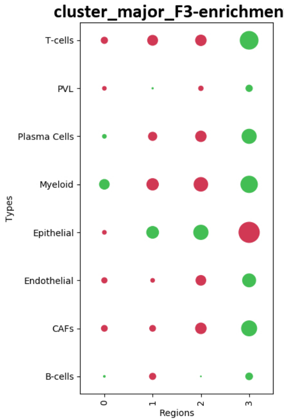

cluster_major_F3-enrichment
T-cells
PVL
Plasma Cells
Myeloid
Epithelial
Endothelial
CAFs
B-cells
Types
0
1
2
3
Regions

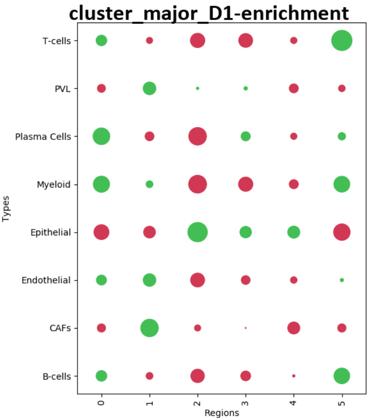

cluster_major_D1-enrichment
T-cells
PVL
Plasma Cells
Myeloid
Epithelial
Endothelial
CAFs
B-cells
Types
0
1
2
3
4
5
Regions

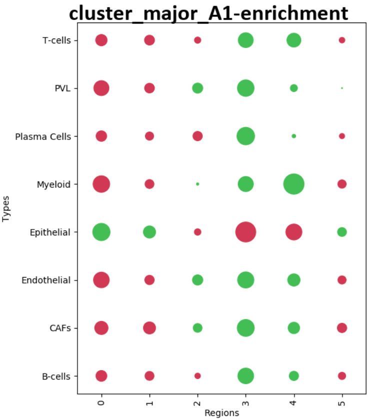
cluster_major_A1-enrichment
T-cells
PVL
Plasma Cells
Myeloid
Epithelial
Endothelial
CAFs
B-cells
Types
0
1
2
3
4
5
Regions

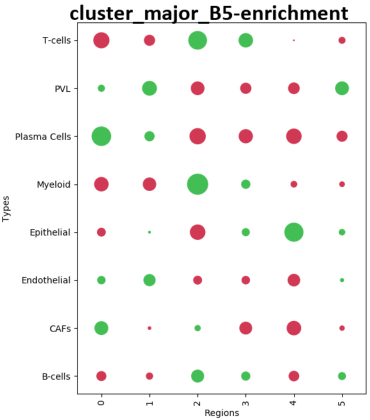

cluster_major_B5-enrichment
T-cells
PVL
Plasma Cells
Myeloid
Epithelial
Endothelial
CAFs
B-cells
Types
0
1
2
3
4
5
Regions

cluster_major_E2-enrichment

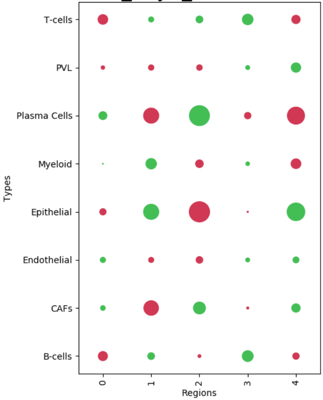

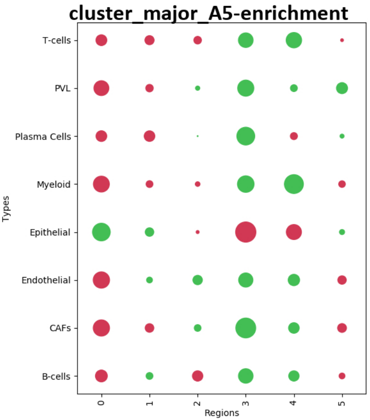

cluster_major_A5-enrichment
T-cells
PVL
Plasma Cells
Myeloid
Epithelial
Endothelial
CAFs
B-cells
Types
0
1
2
3
4
5
Regions

cluster_major_G3-enrichment

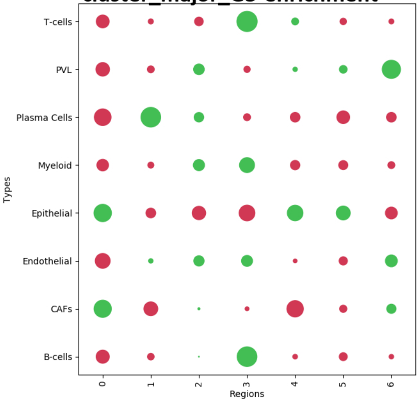

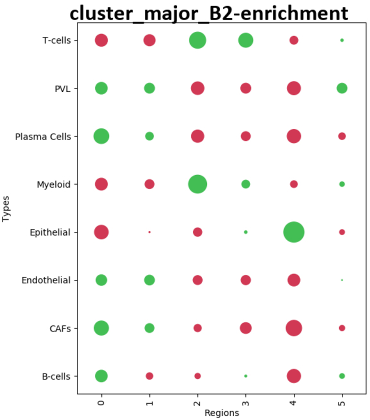
cluster_major_B2-enrichment
T-cells
PVL
Plasma Cells
Myeloid
Epithelial
Endothelial
CAFs
B-cells
Types
0
1
2
3
4
5
Regions

cluster_major_H2-enrichment

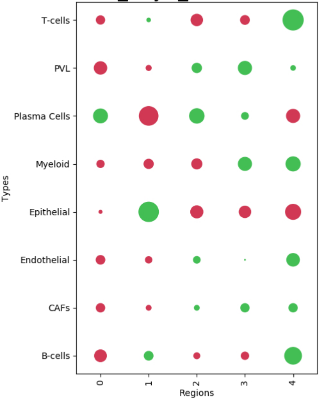

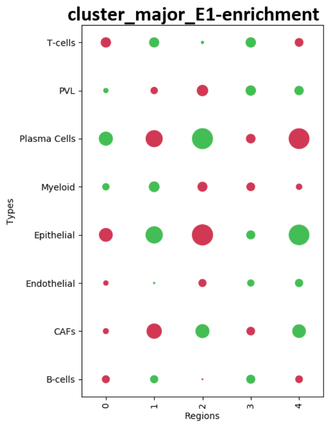
cluster_major_E1-enrichment
T-cells
PVL
Plasma Cells
Myeloid
Epithelial
Endothelial
CAFs
B-cells
Types
0
1
2
3
4
Regions

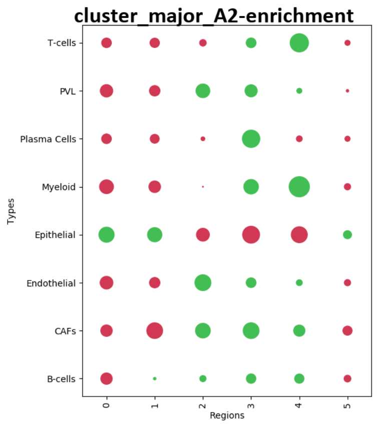

cluster_major_A2-enrichment
T-cells
PVL
Plasma Cells
Myeloid
Epithelial
Endothelial
CAFs
B-cells
Types
0
1
2
3
4
5
Regions

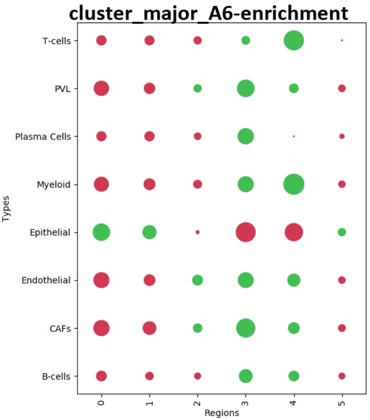
cluster_major_A6-enrichment
T-cells
PVL
Plasma Cells
Myeloid
Epithelial
Endothelial
CAFs
B-cells
Types
0
1
2
3
4
5
Regions

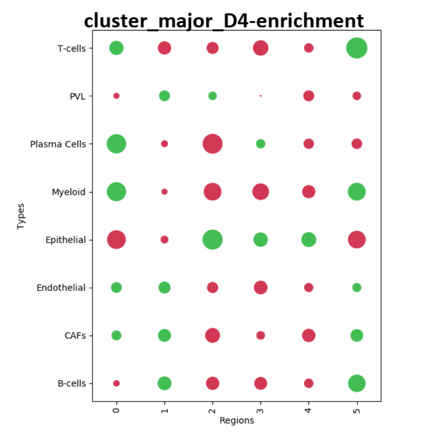
cluster_major_D4-enrichment
T-cells
PVL
Plasma Cells
Myeloid
Epithelial
Endothelial
CAFs
B-cells
Types
0
1
2
3
4
5
Regions

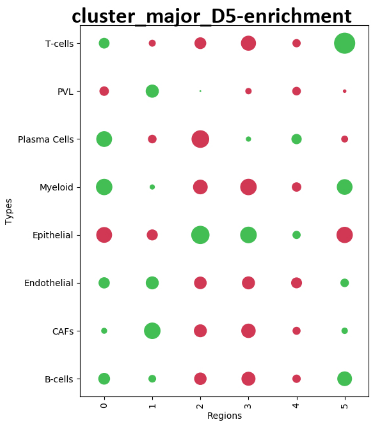

cluster_major_D5-enrichment
T-cells
PVL
Plasma Cells
Myeloid
Epithelial
Endothelial
CAFs
B-cells
Types
0
1
2
3
4
5
Regions

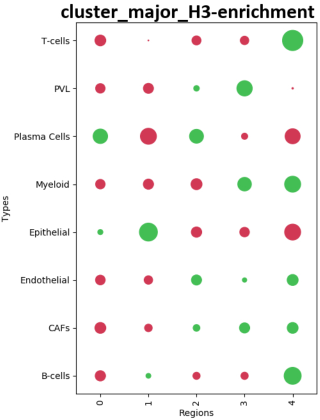

cluster_major_H3-enrichment
T-cells
PVL
Plasma Cells
Myeloid
Epithelial
Endothelial
CAFs
B-cells
Types
0
1
2
3
4
Regions

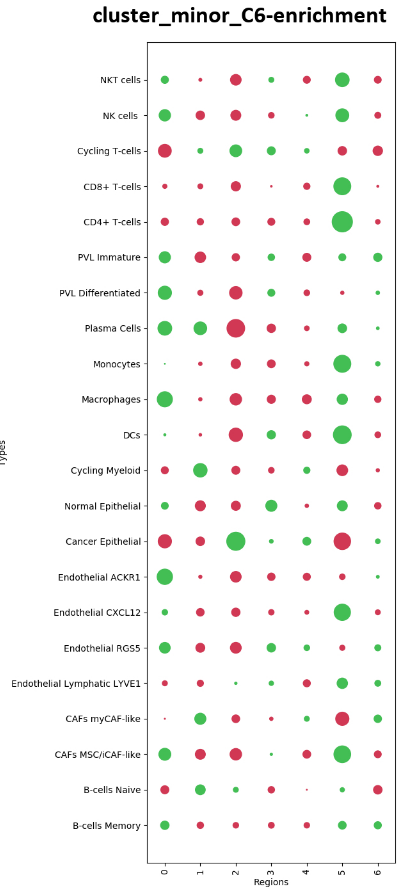
cluster_minor_C6-enrichment
NKT cells
NK cells
Cycling T-cells
CD8+ T-cells
CD4+ T-cells
PVL Immature
PVL Differentiated
Plasma Cells
Monocytes
Macrophages
DCs
Cycling Myeloid
Normal Epithelial
Cancer Epithelial
Endothelial ACKR1
Endothelial CXCL12
Endothelial RGS5
Endothelial Lymphatic LYVE1
CAFs myCAF-like
CAFs MSC/iCAF-like
B-cells Naive
B-cells Memory
Types
0
1
2
3
4
5
6
Regions

cluster_minor_C4-enrichment

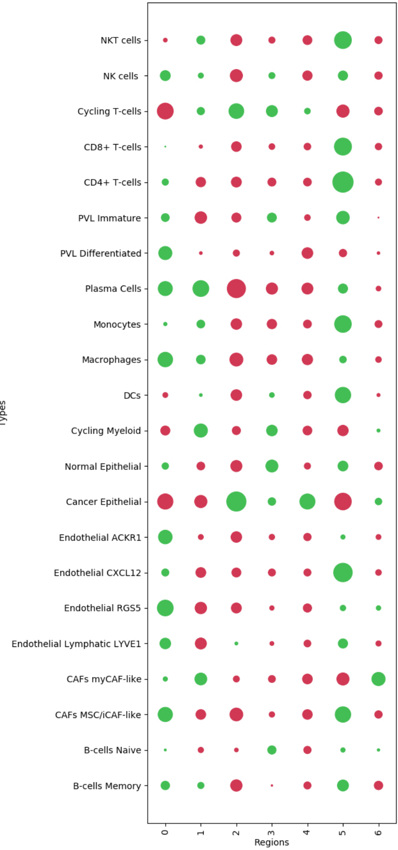

cluster_minor_C3-enrichment

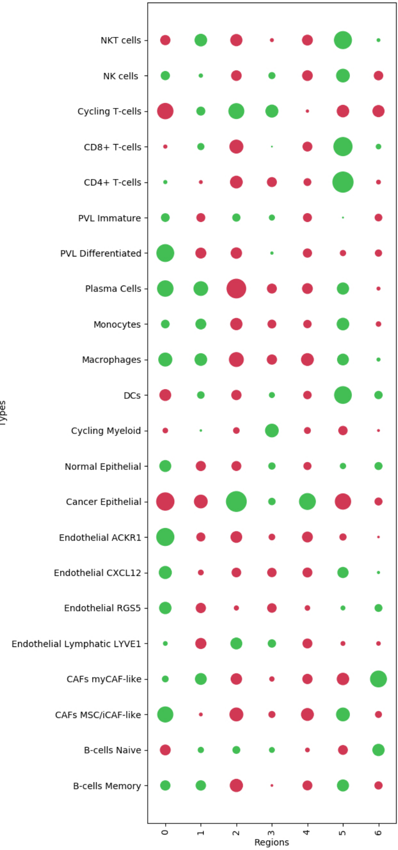

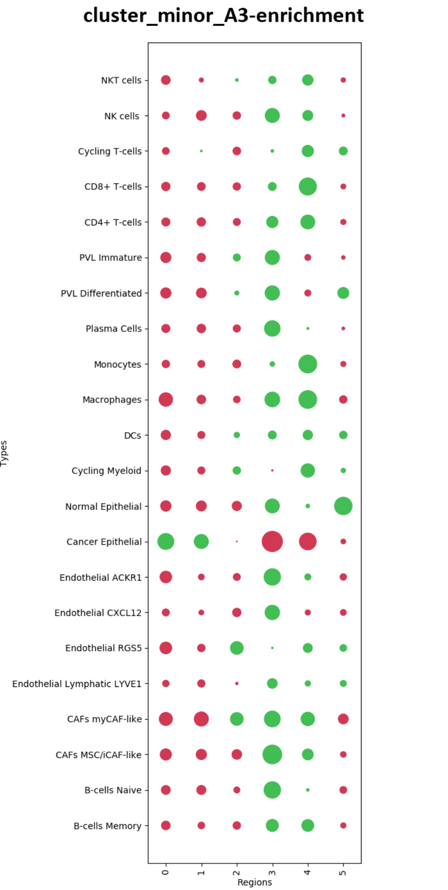

cluster_minor_A3-enrichment
NKT cells
NK cells
Cycling T-cells
CD8+ T-cells
CD4+ T-cells
PVL Immature
PVL Differentiated
Plasma Cells
Monocytes
Macrophages
DCs
Cycling Myeloid
Normal Epithelial
Cancer Epithelial
Endothelial ACKR1
Endothelial CXCL12
Endothelial RGS5
Endothelial Lymphatic LYVE1
CAFs myCAF-like
CAFs MSC/iCAF-like
B-cells Naive
B-cells Memory
Types
0
1
2
3
4
5
Regions

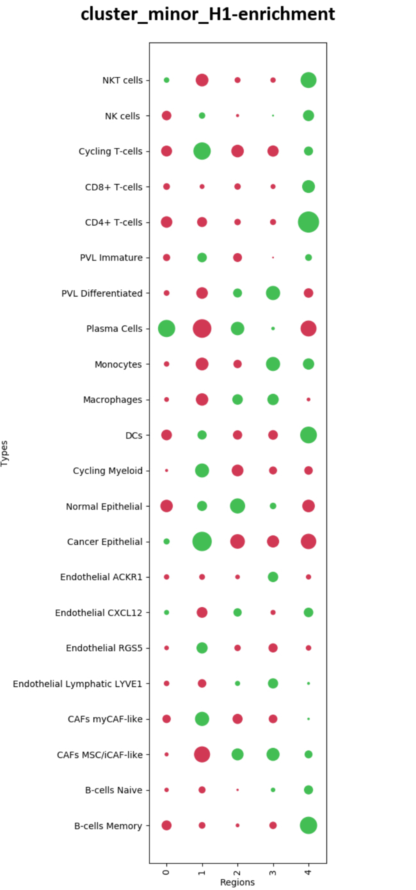
cluster_minor_H1-enrichment
NKT cells
NK cells
Cycling T-cells
CD8+ T-cells
CD4+ T-cells
PVL Immature
PVL Differentiated
Plasma Cells
Monocytes
Macrophages
DCs
Cycling Myeloid
Normal Epithelial
Cancer Epithelial
Endothelial ACKR1
Endothelial CXCL12
Endothelial RGS5
Endothelial Lymphatic LYVE1
CAFs myCAF-like
CAFs MSC/iCAF-like
B-cells Naive
B-cells Memory
Types
0
1
2
3
4
Regions

cluster_minor_B4-enrichment

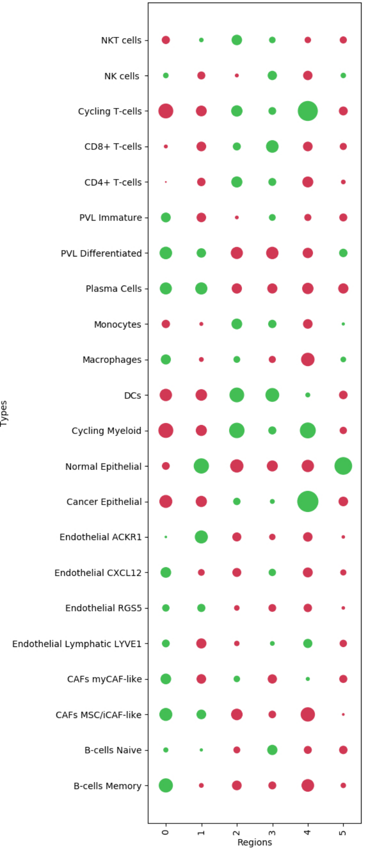

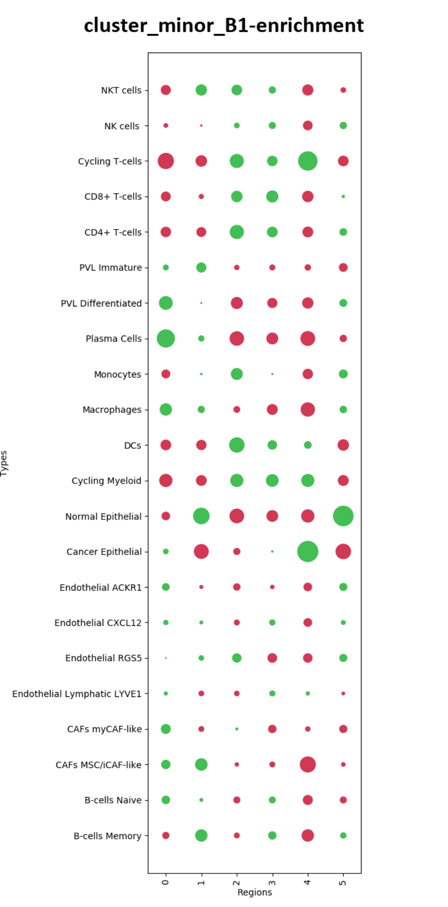
cluster_minor_B1-enrichment
NKT cells
NK cells
Cycling T-cells
CD8+ T-cells
CD4+ T-cells
PVL Immature
PVL Differentiated
Plasma Cells
Monocytes
Macrophages
DCs
Cycling Myeloid
Normal Epithelial
Cancer Epithelial
Endothelial ACKR1
Endothelial CXCL12
Endothelial RGS5
Endothelial Lymphatic LYVE1
CAFs myCAF-like
CAFs MSC/iCAF-like
B-cells Naive
B-cells Memory
Types
0
1
2
3
4
5
Regions

cluster_minor_C2-enrichment

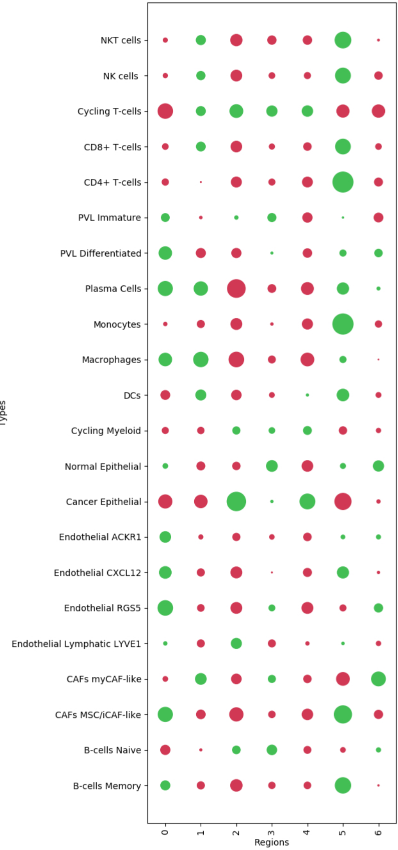

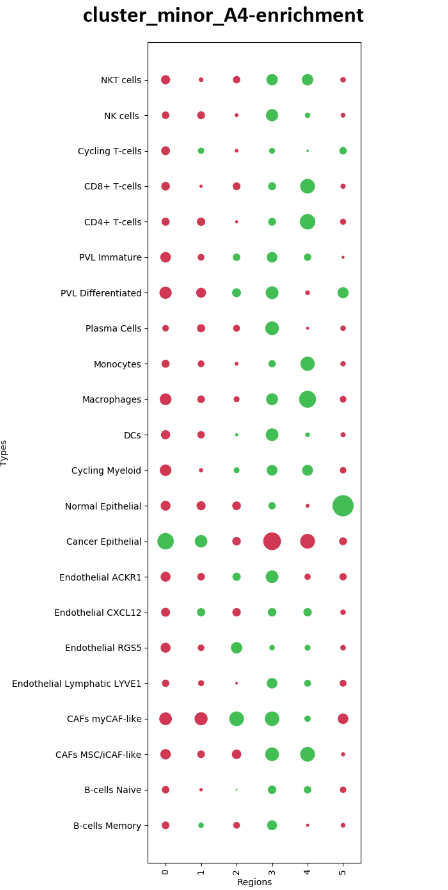
cluster_minor_A4-enrichment
NKT cells
NK cells
Cycling T-cells
CD8+ T-cells
CD4+ T-cells
PVL Immature
PVL Differentiated
Plasma Cells
Monocytes
Macrophages
DCs
Cycling Myeloid
Normal Epithelial
Cancer Epithelial
Endothelial ACKR1
Endothelial CXCL12
Endothelial RGS5
Endothelial Lymphatic LYVE1
CAFs myCAF-like
CAFs MSC/iCAF-like
B-cells Naive
B-cells Memory
Types
0
1
2
3
4
5
Regions

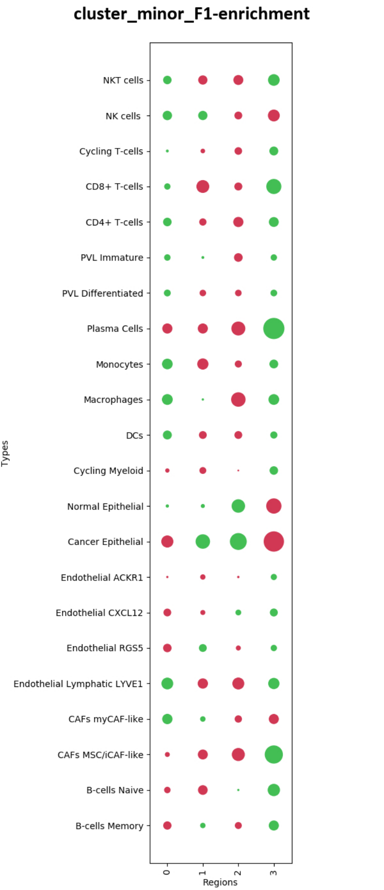
cluster_minor_F1-enrichment
NKT cells
NK cells
Cycling T-cells
CD8+ T-cells
CD4+ T-cells
PVL Immature
PVL Differentiated
Plasma Cells
Monocytes
Macrophages
DCs
Cycling Myeloid
Normal Epithelial
Cancer Epithelial
Endothelial ACKR1
Endothelial CXCL12
Endothelial RGS5
Endothelial Lymphatic LYVE1
CAFs myCAF-like
CAFs MSC/iCAF-like
B-cells Naive
B-cells Memory
Types
0
1
2
3
Regions

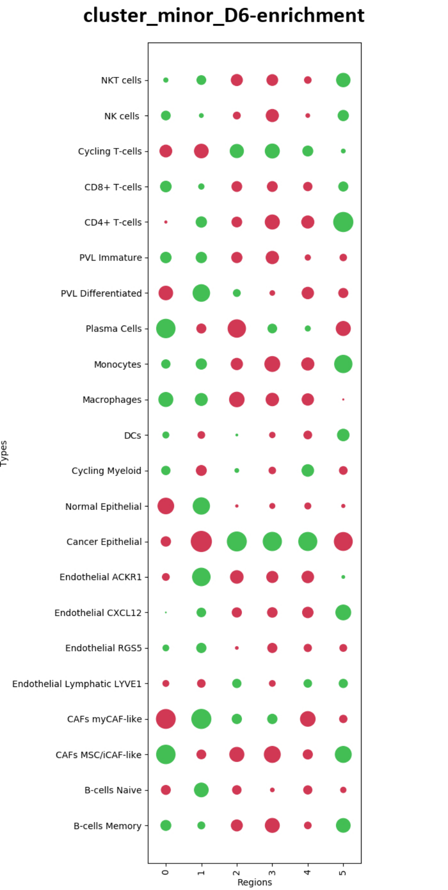
cluster_minor_D6-enrichment
NKT cells
NK cells
Cycling T-cells
CD8+ T-cells
CD4+ T-cells
PVL Immature
PVL Differentiated
Plasma Cells
Monocytes
Macrophages
DCs
Cycling Myeloid
Normal Epithelial
Cancer Epithelial
Endothelial ACKR1
Endothelial CXCL12
Endothelial RGS5
Endothelial Lymphatic LYVE1
CAFs myCAF-like
CAFs MSC/iCAF-like
B-cells Naive
B-cells Memory
Types
0
1
2
3
4
5
Regions

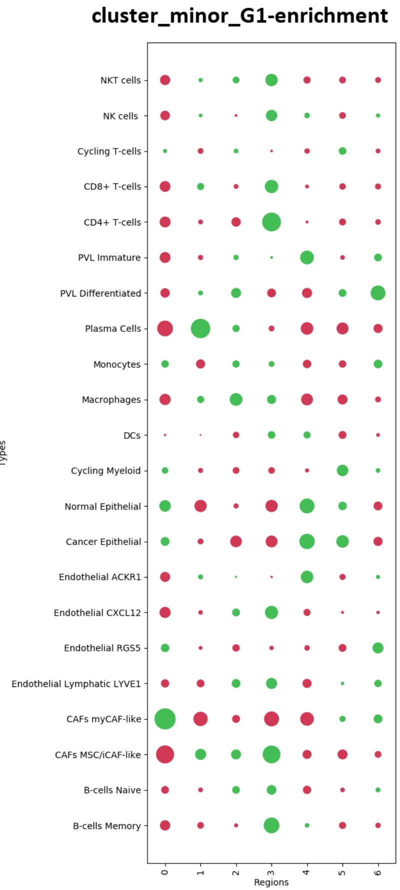
cluster_minor_G1-enrichment
NKT cells
NK cells
Cycling T-cells
CD8+ T-cells
CD4+ T-cells
PVL Immature
PVL Differentiated
Plasma Cells
Monocytes
Macrophages
DCs
Cycling Myeloid
Normal Epithelial
Cancer Epithelial
Endothelial ACKR1
Endothelial CXCL12
Endothelial RGS5
Endothelial Lymphatic LYVE1
CAFs myCAF-like
CAFs MSC/iCAF-like
B-cells Naive
B-cells Memory
Types
0
1
2
3
4
5
6
Regions

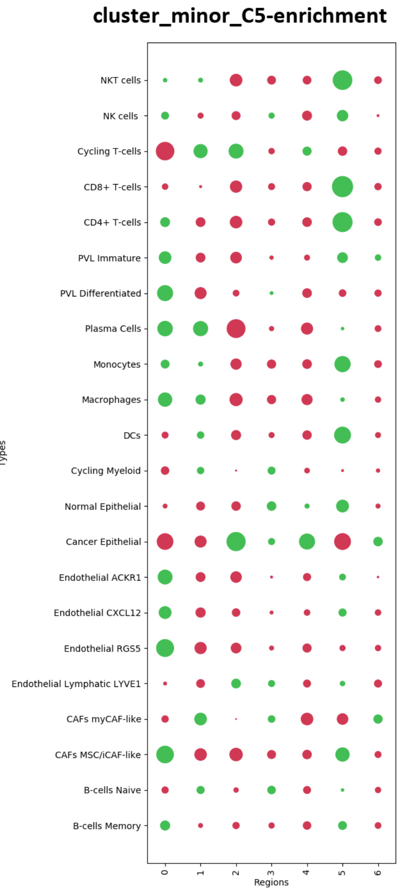
cluster_minor_C5-enrichment
NKT cells
NK cells
Cycling T-cells
CD8+ T-cells
CD4+ T-cells
PVL Immature
PVL Differentiated
Plasma Cells
Monocytes
Macrophages
DCs
Cycling Myeloid
Normal Epithelial
Cancer Epithelial
Endothelial ACKR1
Endothelial CXCL12
Endothelial RGS5
Endothelial Lymphatic LYVE1
CAFs myCAF-like
CAFs MSC/iCAF-like
B-cells Naive
B-cells Memory
Types
0
1
2
3
4
5
6
Regions

cluster_minor_C1-enrichment

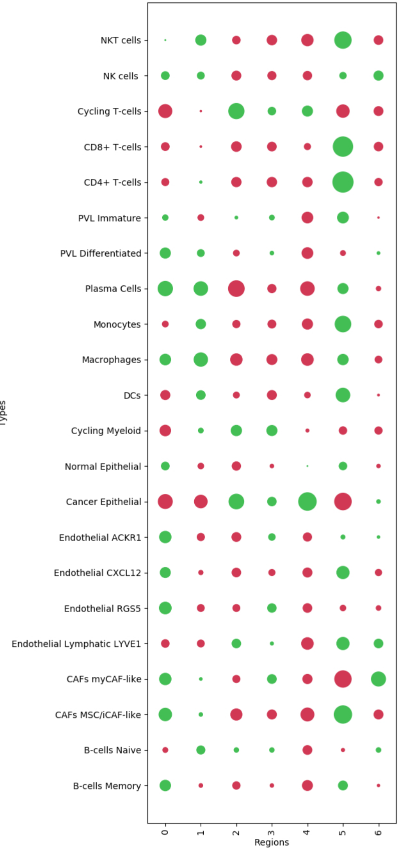

cluster_minor_B6-enrichment

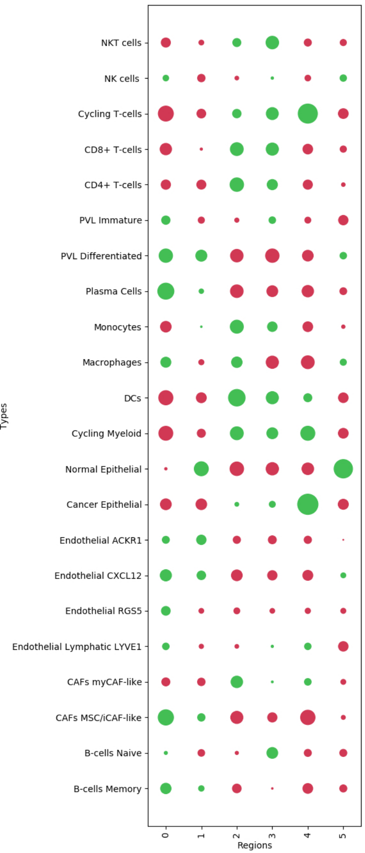

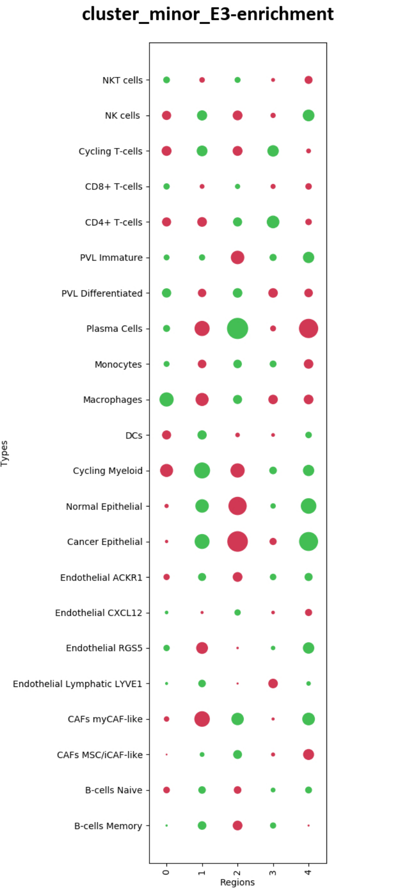
cluster_minor_E3-enrichment
NKT cells
NK cells
Cycling T-cells
CD8+ T-cells
CD4+ T-cells
PVL Immature
PVL Differentiated
Plasma Cells
Monocytes
Macrophages
DCs
Cycling Myeloid
Normal Epithelial
Cancer Epithelial
Endothelial ACKR1
Endothelial CXCL12
Endothelial RGS5
Endothelial Lymphatic LYVE1
CAFs myCAF-like
CAFs MSC/iCAF-like
B-cells Naive
B-cells Memory
Types
0
1
2
3
4
Regions

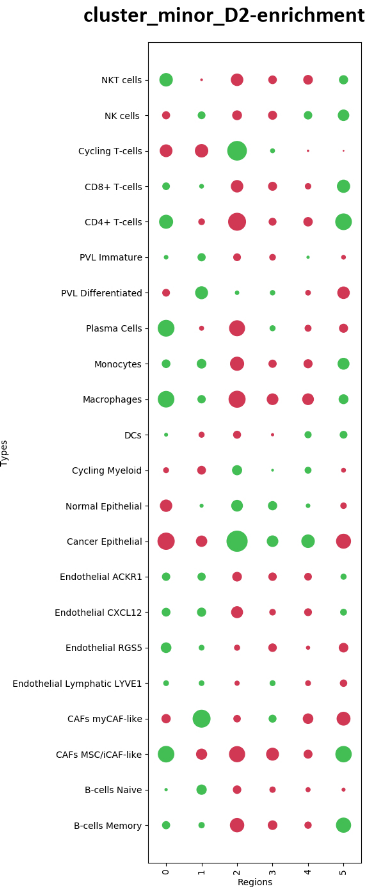
cluster_minor_D2-enrichment
NKT cells
NK cells
Cycling T-cells
CD8+ T-cells
CD4+ T-cells
PVL Immature
PVL Differentiated
Plasma Cells
Monocytes
Macrophages
DCs
Cycling Myeloid
Normal Epithelial
Cancer Epithelial
Endothelial ACKR1
Endothelial CXCL12
Endothelial RGS5
Endothelial Lymphatic LYVE1
CAFs myCAF-like
CAFs MSC/iCAF-like
B-cells Naive
B-cells Memory
Types
0
1
2
3
4
5
Regions

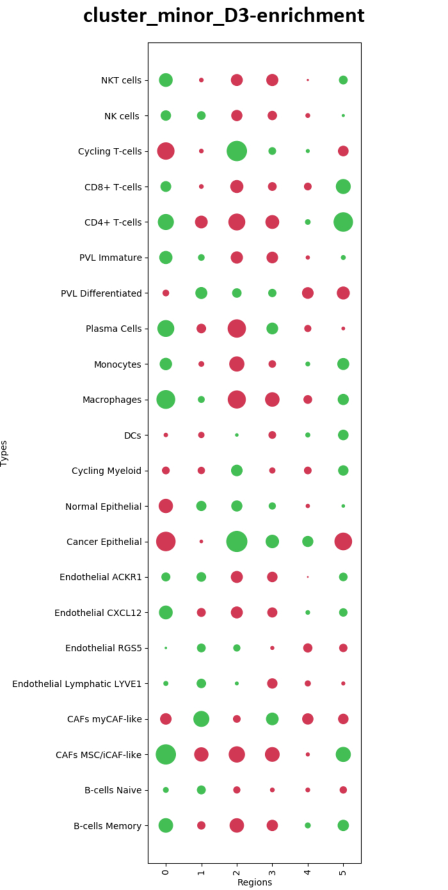

cluster_minor_D3-enrichment
NKT cells
NK cells
Cycling T-cells
CD8+ T-cells
CD4+ T-cells
PVL Immature
PVL Differentiated
Plasma Cells
Monocytes
Macrophages
DCs
Cycling Myeloid
Normal Epithelial
Cancer Epithelial
Endothelial ACKR1
Endothelial CXCL12
Endothelial RGS5
Endothelial Lymphatic LYVE1
CAFs myCAF-like
CAFs MSC/iCAF-like
B-cells Naive
B-cells Memory
Types
0
1
2
3
4
5
Regions

cluster_minor_G2-enrichment

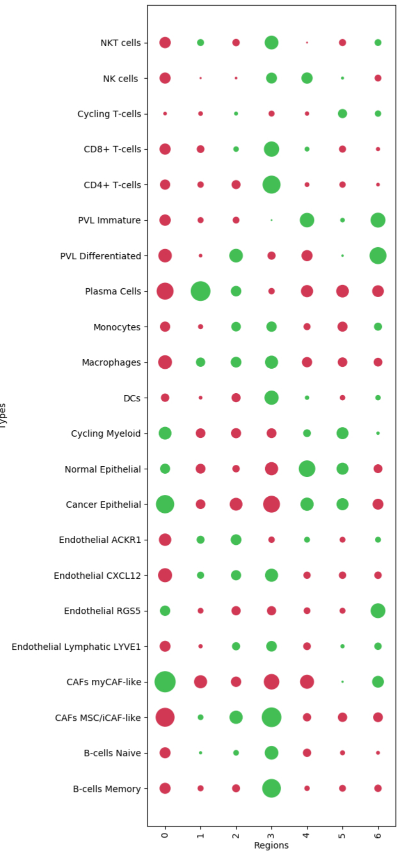

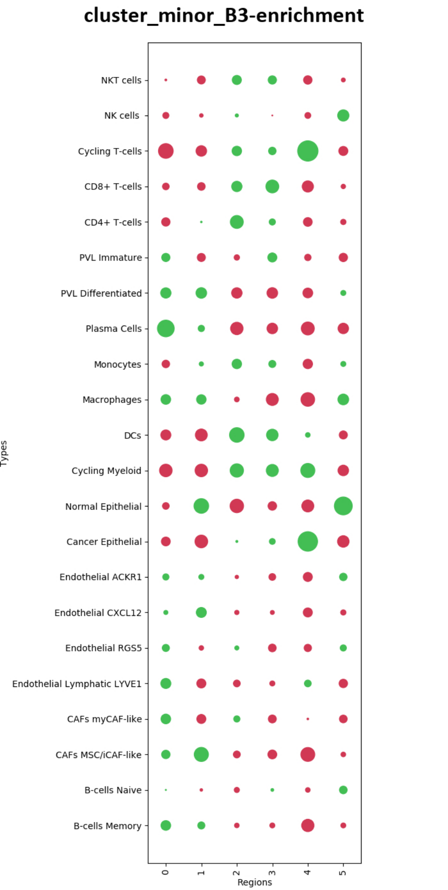
cluster_minor_B3-enrichment
Types
NKT cells
NK cells
Cycling T-cells
CD8+ T-cells
CD4+ T-cells
PVL Immature
PVL Differentiated
Plasma Cells
Monocytes
Macrophages
DCs
Cycling Myeloid
Normal Epithelial
Cancer Epithelial
Endothelial ACKR1
Endothelial CXCL12
Endothelial RGS5
Endothelial Lymphatic LYVE1
CAFs myCAF-like
CAFs MSC/iCAF-like
B-cells Naive
B-cells Memory
0
1
2
3
4
5
Regions

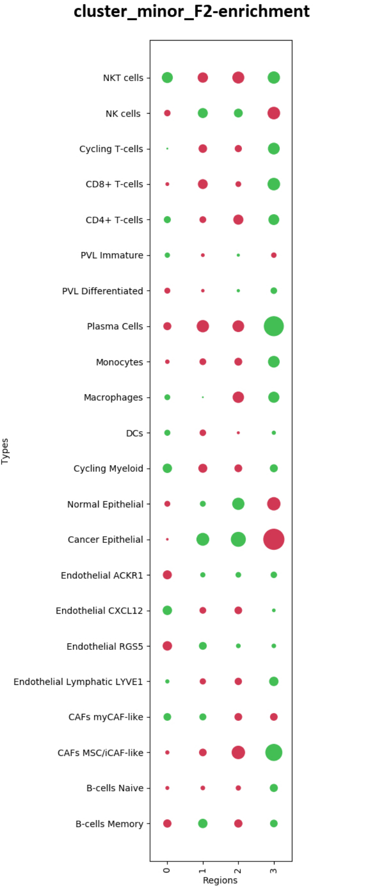

cluster_minor_F2-enrichment
NKT cells
NK cells
Cycling T-cells
CD8+ T-cells
CD4+ T-cells
PVL Immature
PVL Differentiated
Plasma Cells
Monocytes
Macrophages
DCs
Cycling Myeloid
Normal Epithelial
Cancer Epithelial
Endothelial ACKR1
Endothelial CXCL12
Endothelial RGS5
Endothelial Lymphatic LYVE1
CAFs myCAF-like
CAFs MSC/iCAF-like
B-cells Naive
B-cells Memory
Types
0
1
2
3
Regions

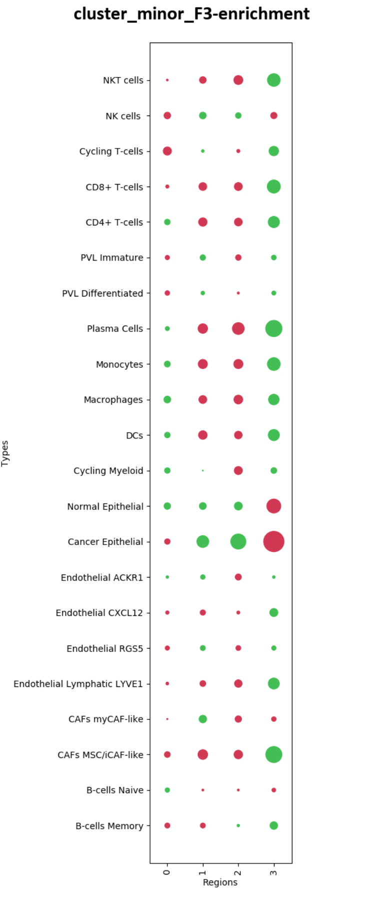
cluster_minor_F3-enrichment
NKT cells
NK cells
Cycling T-cells
CD8+ T-cells
CD4+ T-cells
PVL Immature
PVL Differentiated
Plasma Cells
Monocytes
Macrophages
DCs
Cycling Myeloid
Normal Epithelial
Cancer Epithelial
Endothelial ACKR1
Endothelial CXCL12
Endothelial RGS5
Endothelial Lymphatic LYVE1
CAFs myCAF-like
CAFs MSC/iCAF-like
B-cells Naive
B-cells Memory
Types
0
1
2
3
Regions

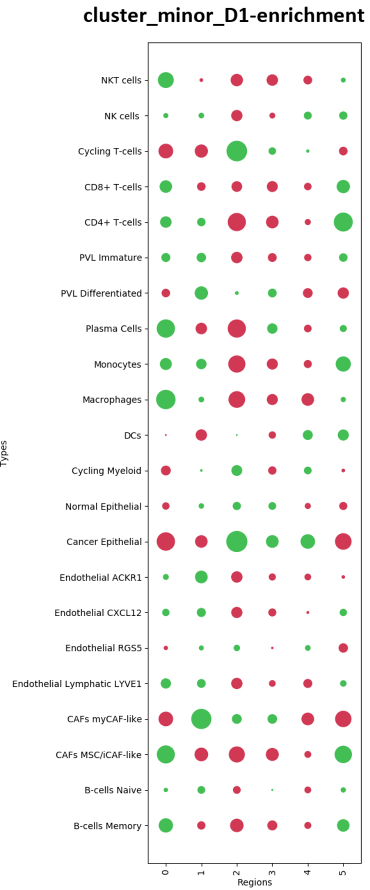
cluster_minor_D1-enrichment
NKT cells
NK cells
Cycling T-cells
CD8+ T-cells
CD4+ T-cells
PVL Immature
PVL Differentiated
Plasma Cells
Monocytes
Macrophages
DCs
Cycling Myeloid
Normal Epithelial
Cancer Epithelial
Endothelial ACKR1
Endothelial CXCL12
Endothelial RGS5
Endothelial Lymphatic LYVE1
CAFs myCAF-like
CAFs MSC/iCAF-like
B-cells Naive
B-cells Memory
Types
0
1
2
3
4
5
Regions

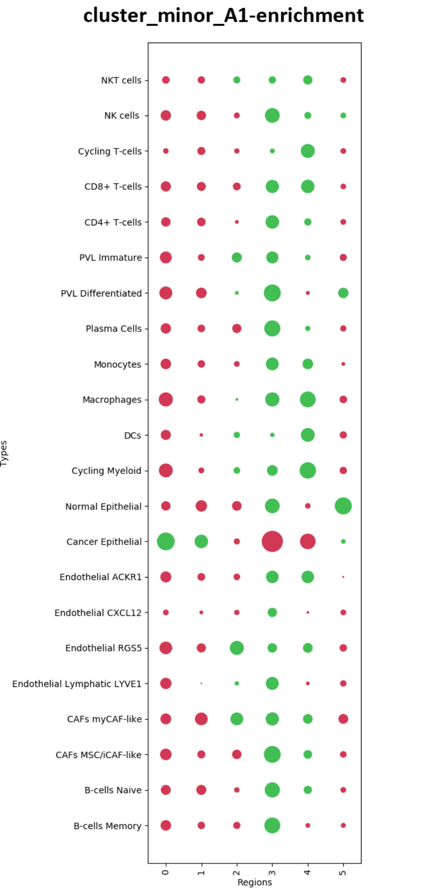
cluster_minor_A1-enrichment
NKT cells
NK cells
Cycling T-cells
CD8+ T-cells
CD4+ T-cells
PVL Immature
PVL Differentiated
Plasma Cells
Monocytes
Macrophages
DCs
Cycling Myeloid
Normal Epithelial
Cancer Epithelial
Endothelial ACKR1
Endothelial CXCL12
Endothelial RGS5
Endothelial Lymphatic LYVE1
CAFs myCAF-like
CAFs MSC/iCAF-like
B-cells Naive
B-cells Memory
Types
0
1
2
3
4
5
Regions

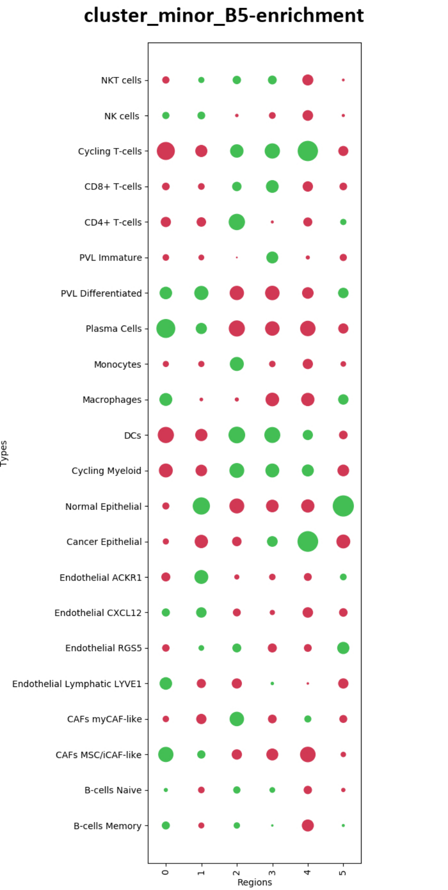
cluster_minor_B5-enrichment
NKT cells
NK cells
Cycling T-cells
CD8+ T-cells
CD4+ T-cells
PVL Immature
PVL Differentiated
Plasma Cells
Monocytes
Macrophages
DCs
Cycling Myeloid
Normal Epithelial
Cancer Epithelial
Endothelial ACKR1
Endothelial CXCL12
Endothelial RGS5
Endothelial Lymphatic LYVE1
CAFs myCAF-like
CAFs MSC/iCAF-like
B-cells Naive
B-cells Memory
Types
0
1
2
3
4
5
Regions

cluster_minor_E2-enrichment

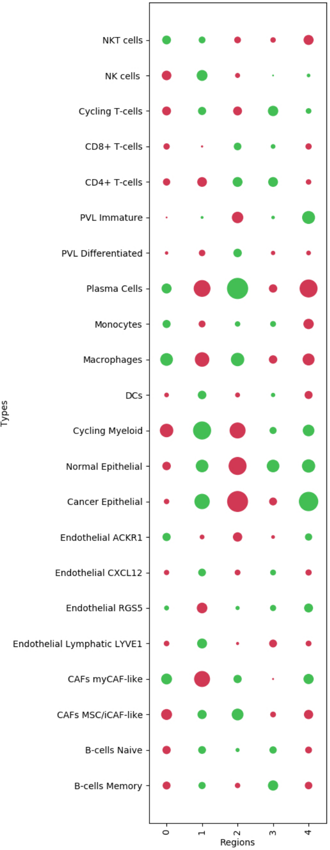

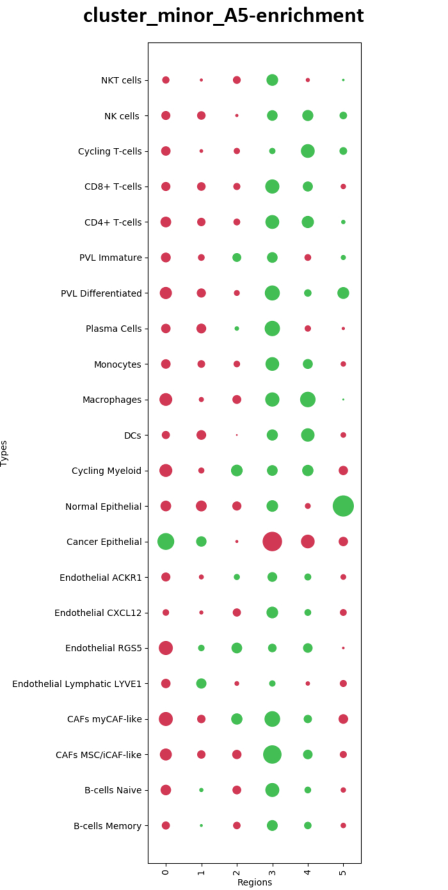
cluster_minor_A5-enrichment
NKT cells
NK cells
Cycling T-cells
CD8+ T-cells
CD4+ T-cells
PVL Immature
PVL Differentiated
Plasma Cells
Monocytes
Macrophages
DCs
Cycling Myeloid
Normal Epithelial
Cancer Epithelial
Endothelial ACKR1
Endothelial CXCL12
Endothelial RGS5
Endothelial Lymphatic LYVE1
CAFs myCAF-like
CAFs MSC/iCAF-like
B-cells Naive
B-cells Memory
Types
0
1
2
3
4
5
Regions

cluster_minor_G3-enrichment

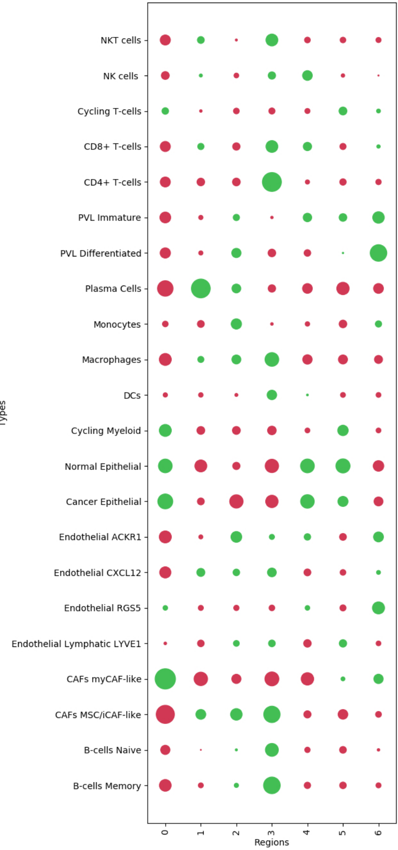

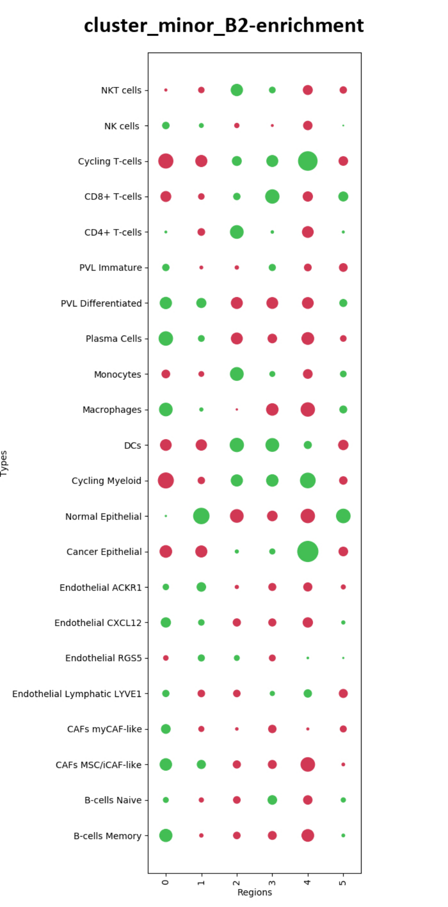
cluster_minor_B2-enrichment
NKT cells
NK cells
Cycling T-cells
CD8+ T-cells
CD4+ T-cells
PVL Immature
PVL Differentiated
Plasma Cells
Monocytes
Macrophages
DCs
Cycling Myeloid
Normal Epithelial
Cancer Epithelial
Endothelial ACKR1
Endothelial CXCL12
Endothelial RGS5
Endothelial Lymphatic LYVE1
CAFs myCAF-like
CAFs MSC/iCAF-like
B-cells Naive
B-cells Memory
Types
0
1
2
3
4
5
Regions

cluster_minor_H2-enrichment

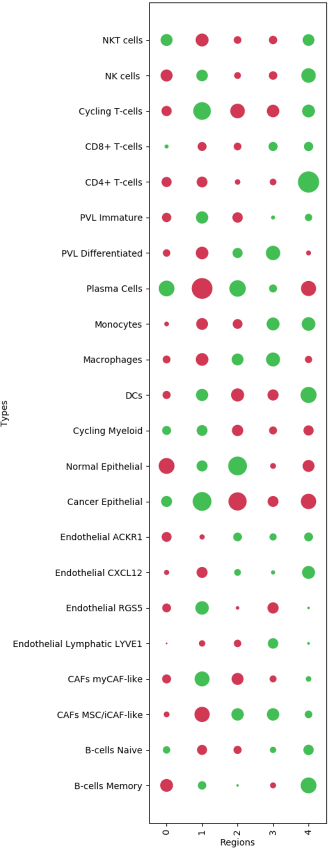

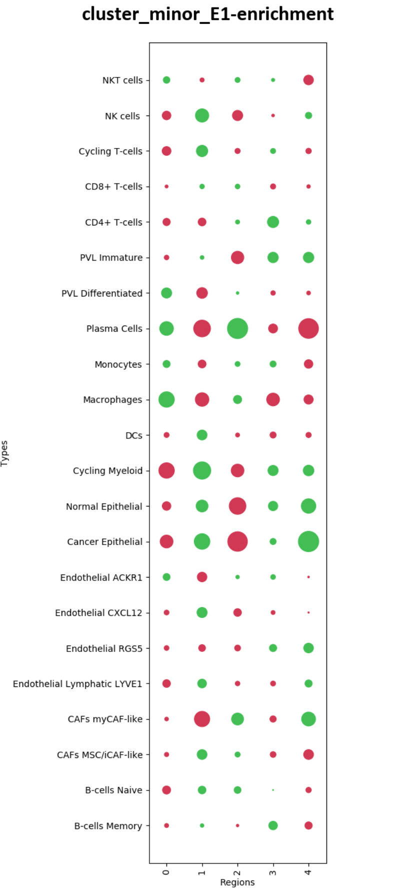
cluster_minor_E1-enrichment
NKT cells
NK cells
Cycling T-cells
CD8+ T-cells
CD4+ T-cells
PVL Immature
PVL Differentiated
Plasma Cells
Monocytes
Macrophages
DCs
Cycling Myeloid
Normal Epithelial
Cancer Epithelial
Endothelial ACKR1
Endothelial CXCL12
Endothelial RGS5
Endothelial Lymphatic LYVE1
CAFs myCAF-like
CAFs MSC/iCAF-like
B-cells Naive
B-cells Memory
Types
0
1
2
3
4
Regions

cluster_minor_A2-enrichment

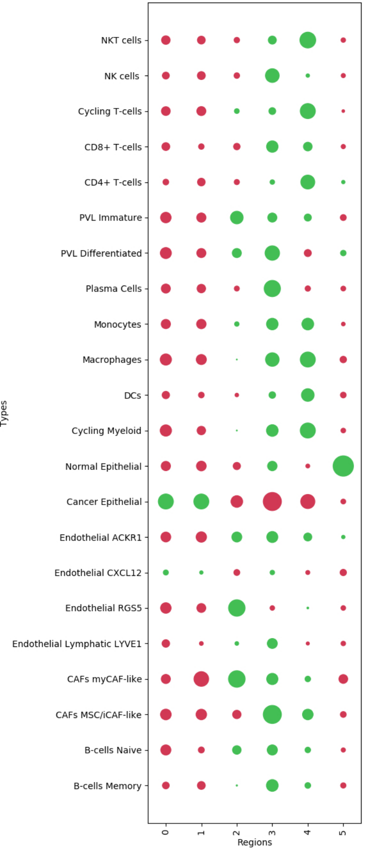

cluster_minor_A6-enrichment

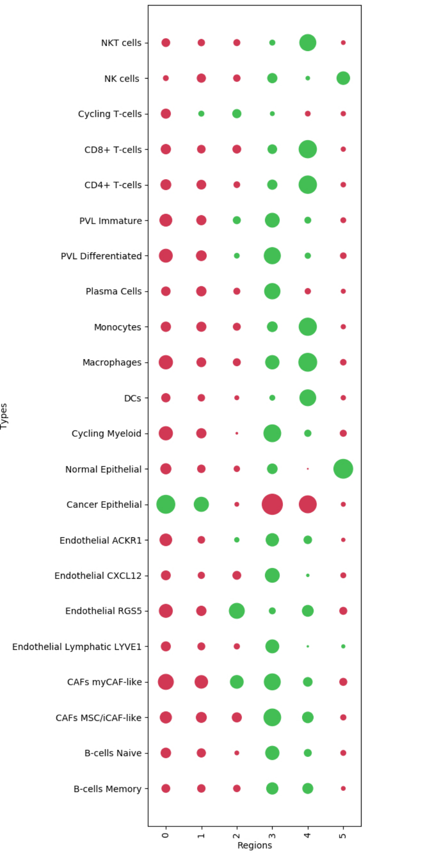

cluster_minor_D4-enrichment

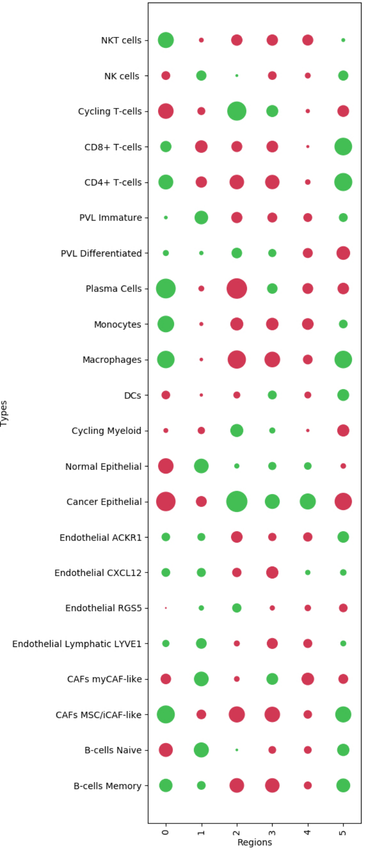

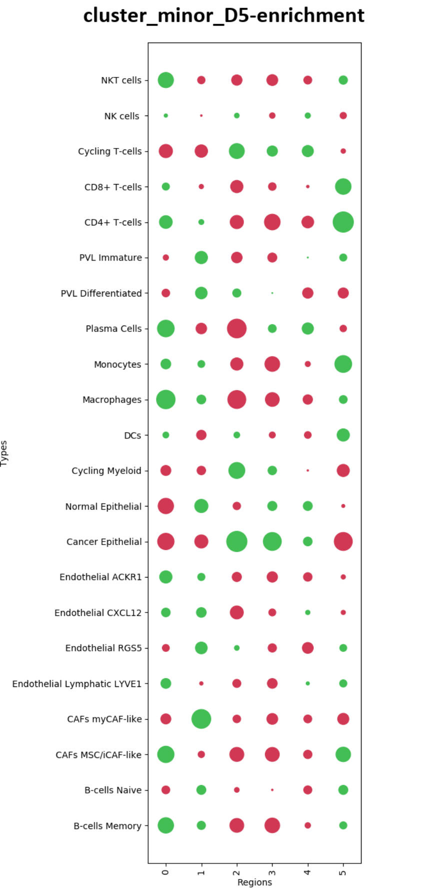
cluster_minor_D5-enrichment
NKT cells
NK cells
Cycling T-cells
CD8+ T-cells
CD4+ T-cells
PVL Immature
PVL Differentiated
Plasma Cells
Monocytes
Macrophages
DCs
Cycling Myeloid
Normal Epithelial
Cancer Epithelial
Endothelial ACKR1
Endothelial CXCL12
Endothelial RGS5
Endothelial Lymphatic LYVE1
CAFs myCAF-like
CAFs MSC/iCAF-like
B-cells Naive
B-cells Memory
Types
0
1
2
3
4
5
Regions

cluster_minor_H3-enrichment

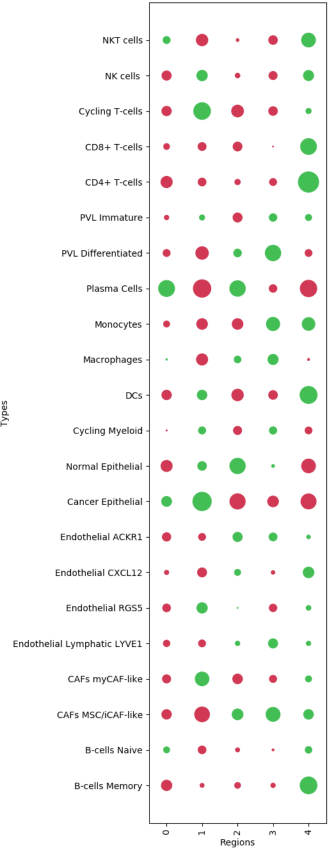

Supplement: Supplementary file 12 — Supplementary Data 9 [file 41467_2021_26271_MOESM12_ESM.pdf]
