# Supplementary material for: Spatial deconvolution of HER2-positive breast cancer delineates tumor-associated cell type interactions
Source: Nat Commun. 2021 Oct 14;12:6012. doi: 10.1038/s41467-021-26271-2 (PMC8516894; doi:10.1038/s41467-021-26271-2)
Supplement: Supplementary file 13 — Supplementary Data 10 [file 41467_2021_26271_MOESM13_ESM.pdf]

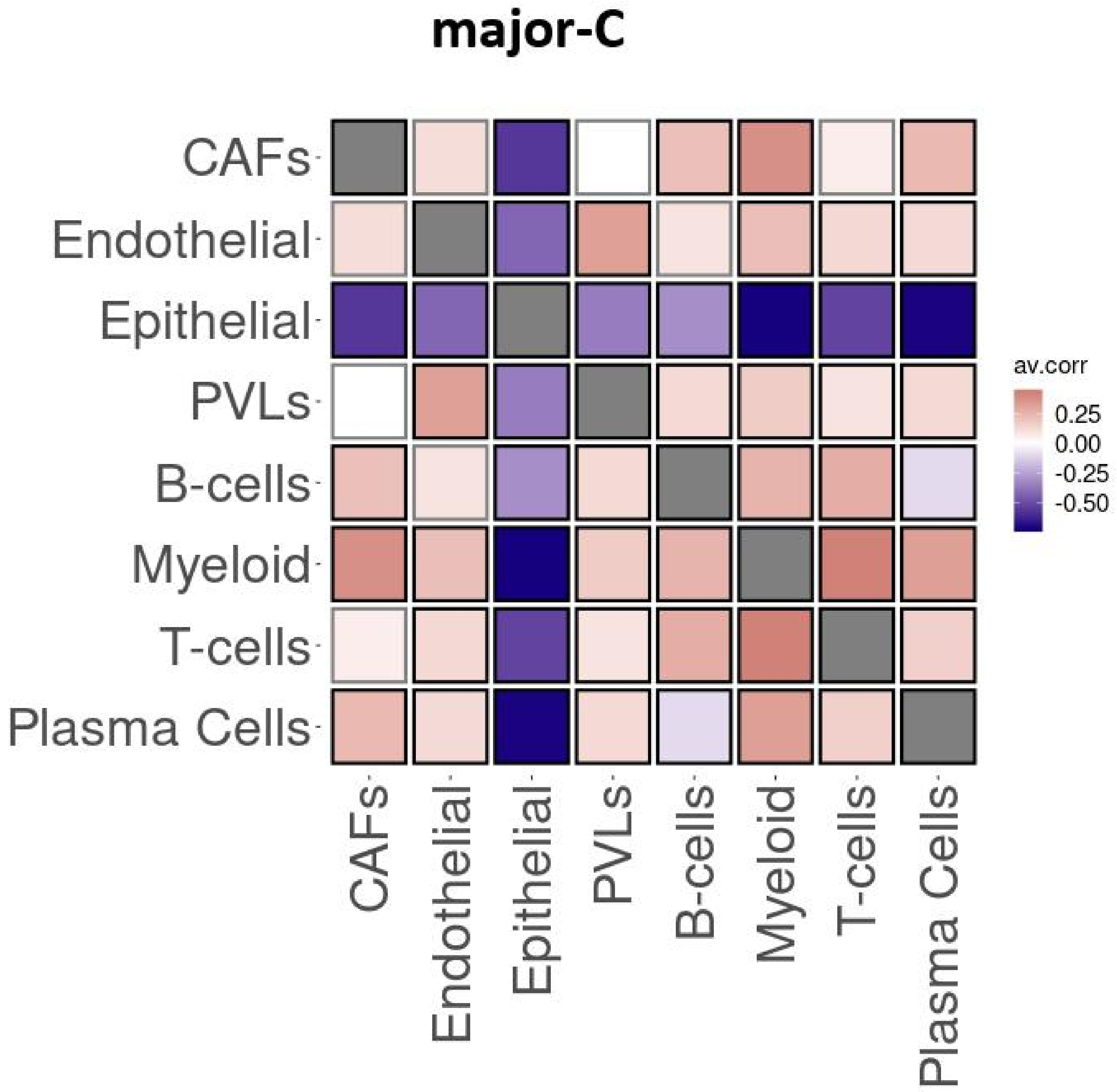

major-C
CAFs
Endothelial
Epithelial
PVLs
B-cells
Myeloid
T-cells
Plasma Cells
av.corr
0.25
0.00
-0.25
-0.50

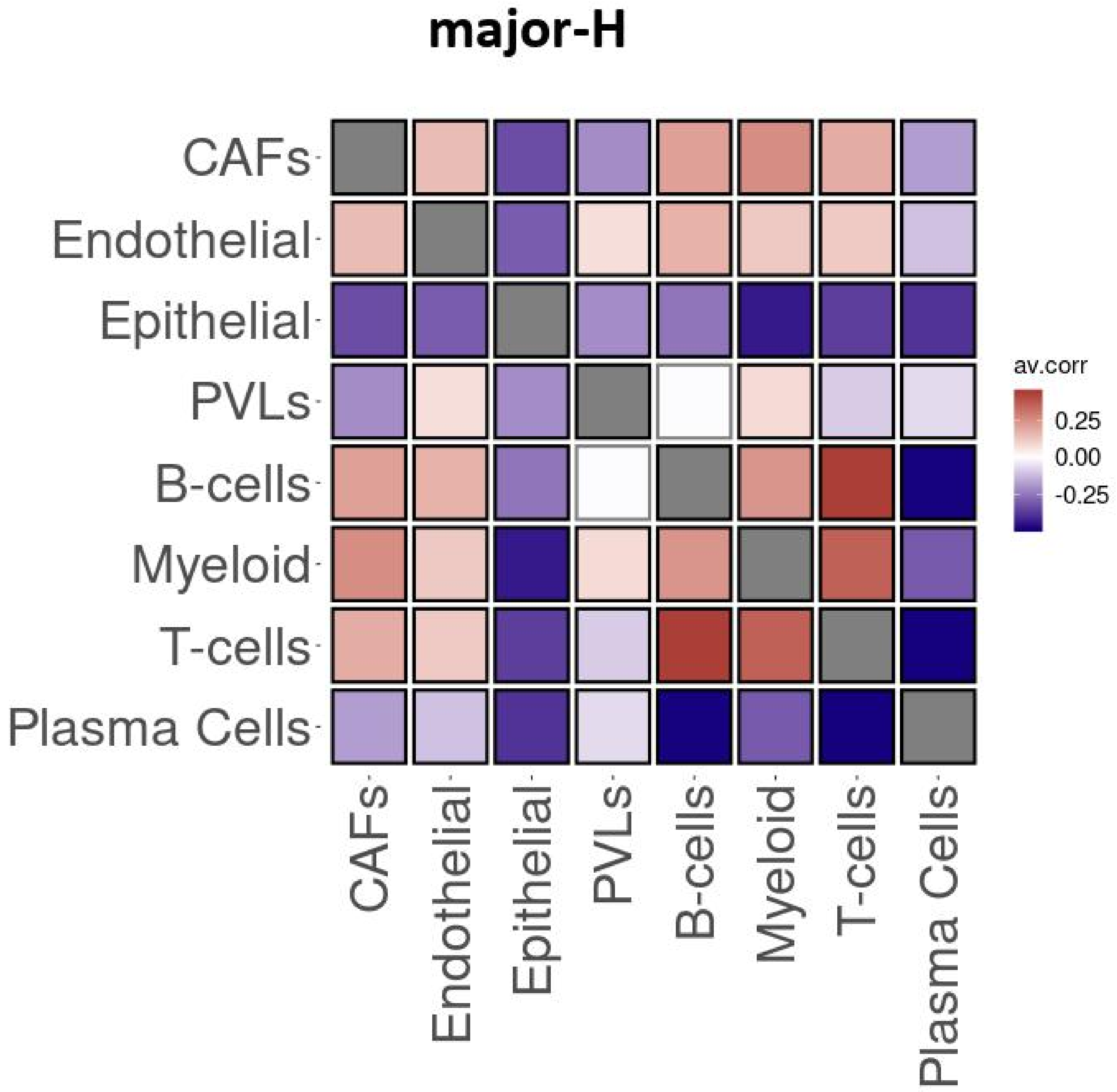
major-H
CAFs
Endothelial
Epithelial
PVLs
B-cells
Myeloid
T-cells
Plasma Cells
av.corr
0.25
0.00
-0.25

major-E

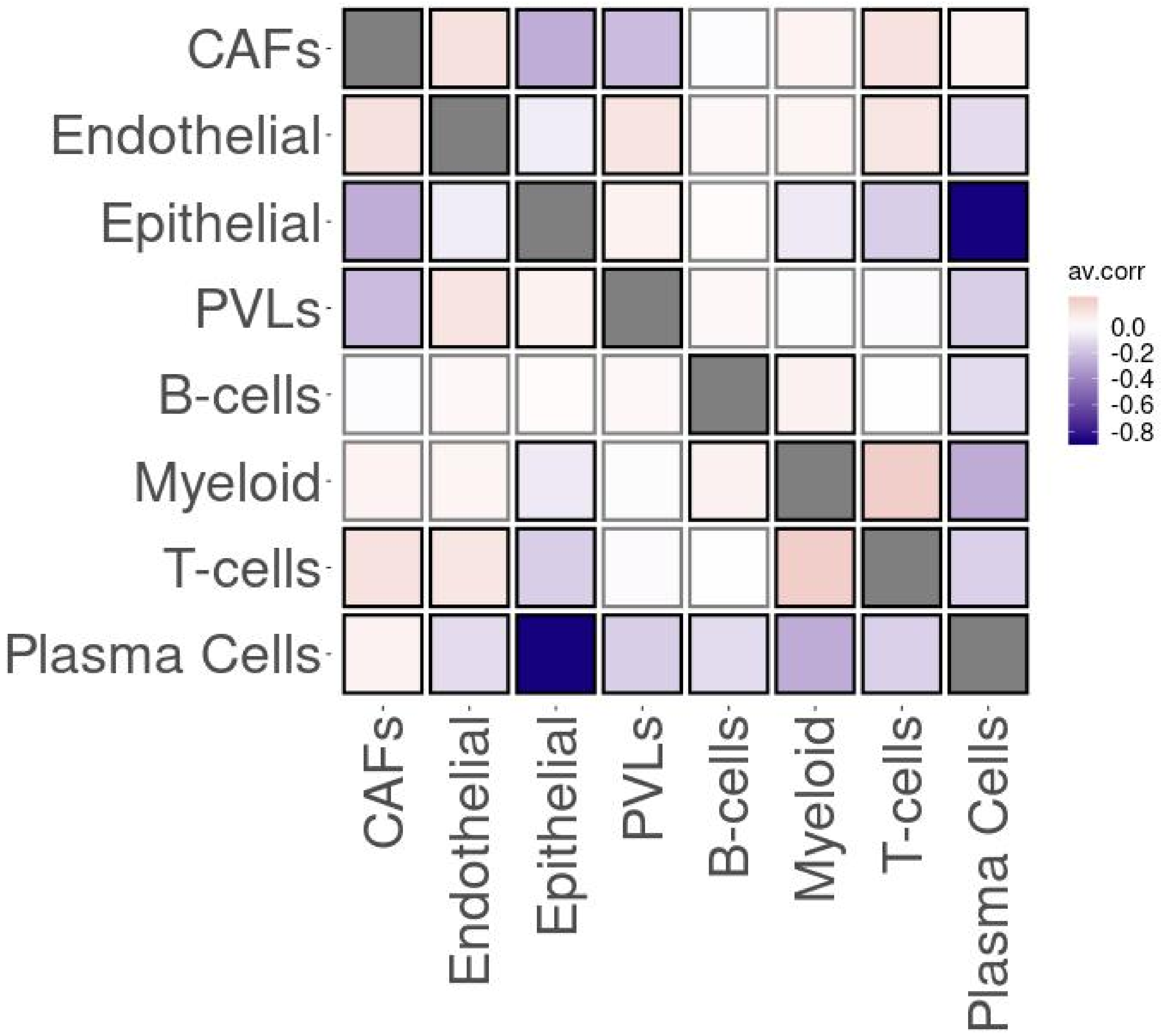
CAFs
Endothelial
Epithelial
PVLs
B-cells
Myeloid
T-cells
Plasma Cells
av.corr
0.0
-0.2
-0.4
-0.6
-0.8

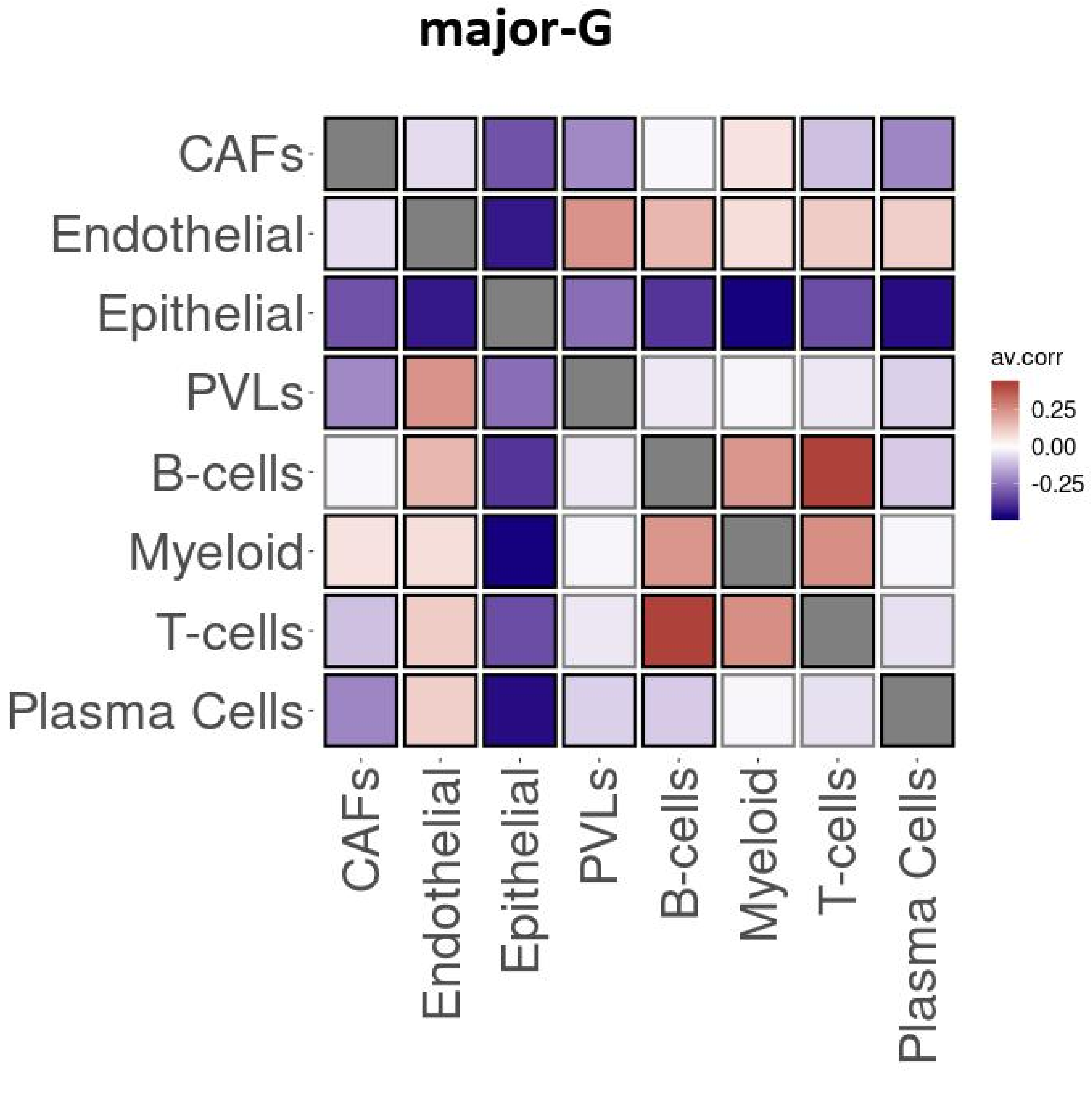

major-G
CAFs
Endothelial
Epithelial
PVLs
B-cells
Myeloid
T-cells
Plasma Cells
av.corr
0.25
0.00
-0.25

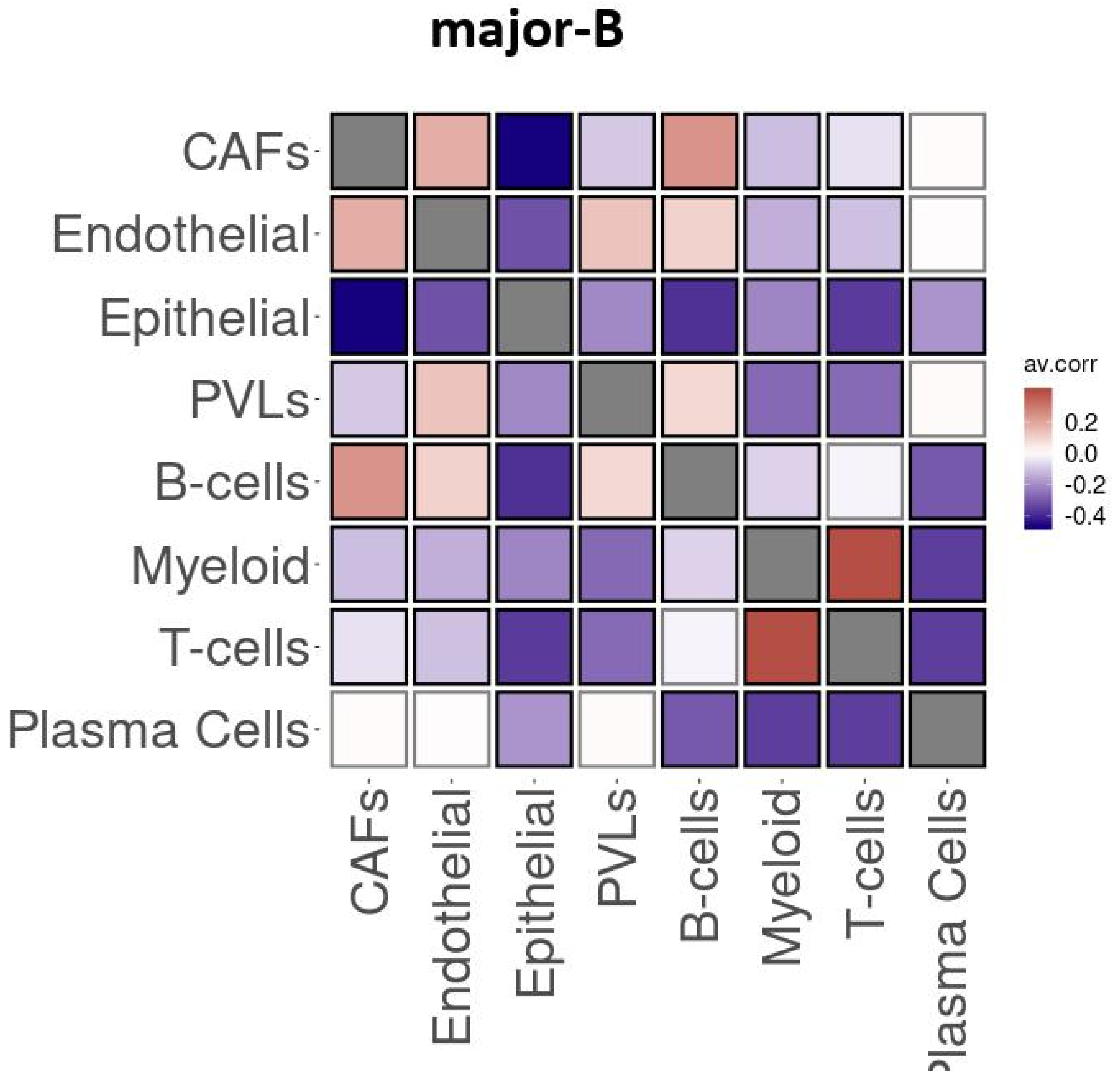
major-B
CAFs
Endothelial
Epithelial
PVLs
B-cells
Myeloid
T-cells
Plasma Cells
av.corr
0.2
0.0
-0.2
-0.4

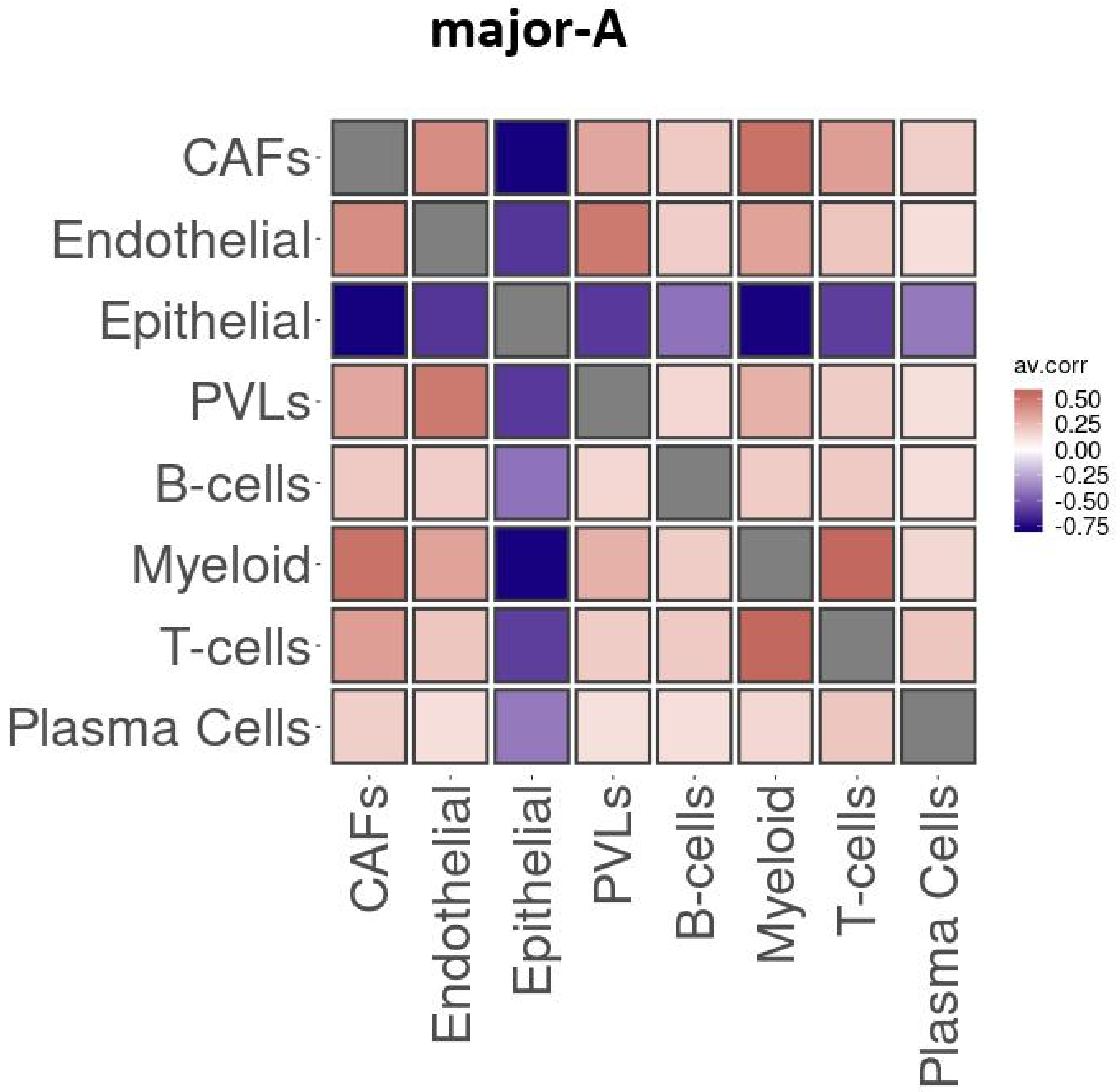
major-A
CAFs
Endothelial
Epithelial
PVLs
B-cells
Myeloid
T-cells
Plasma Cells
av.corr
0.50
0.25
0.00
-0.25
-0.50
-0.75

major-D

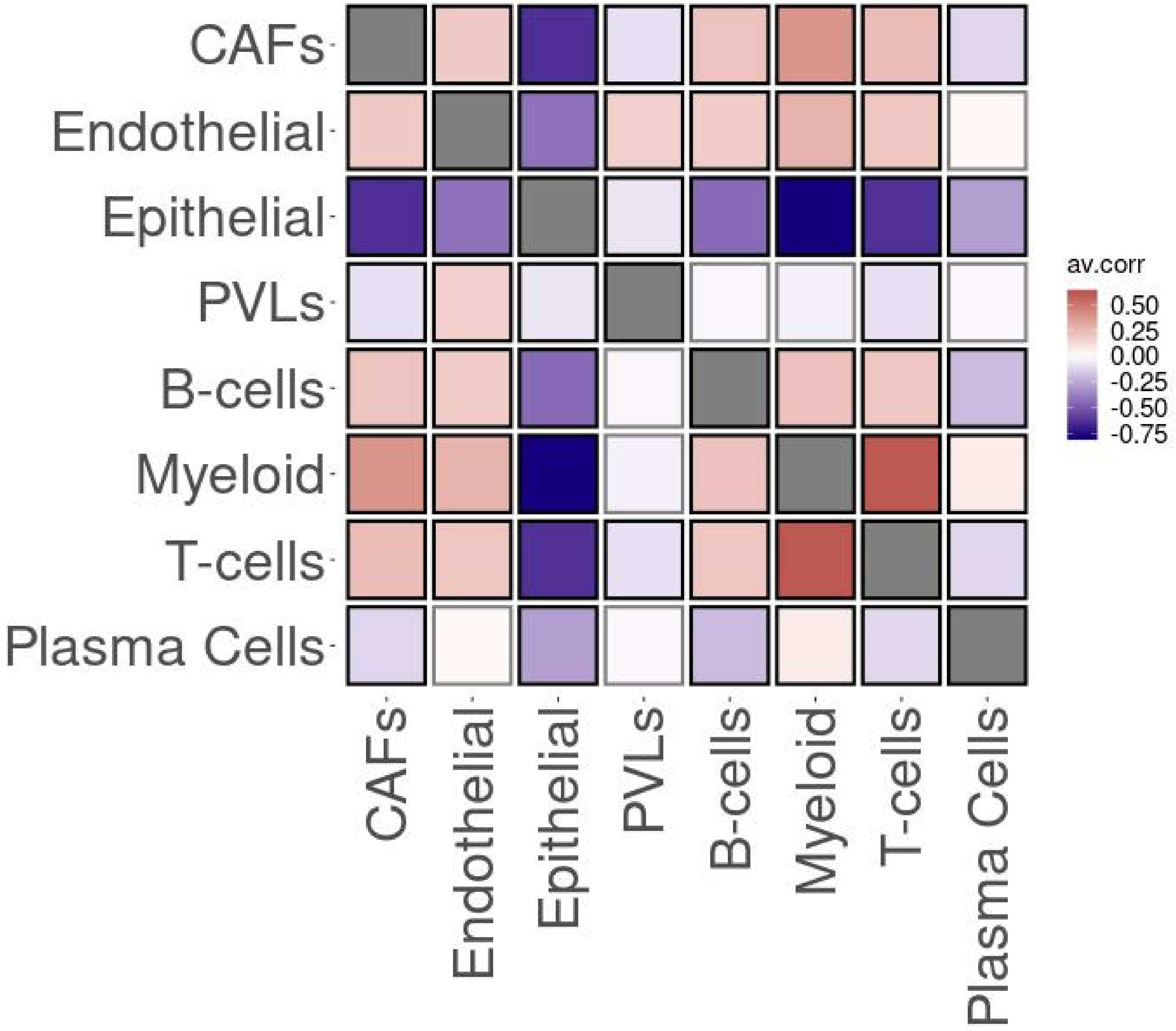
CAFs
Endothelial
Epithelial
PVLs
B-cells
Myeloid
T-cells
Plasma Cells
av.corr
0.50
0.25
0.00
-0.25
-0.50
-0.75

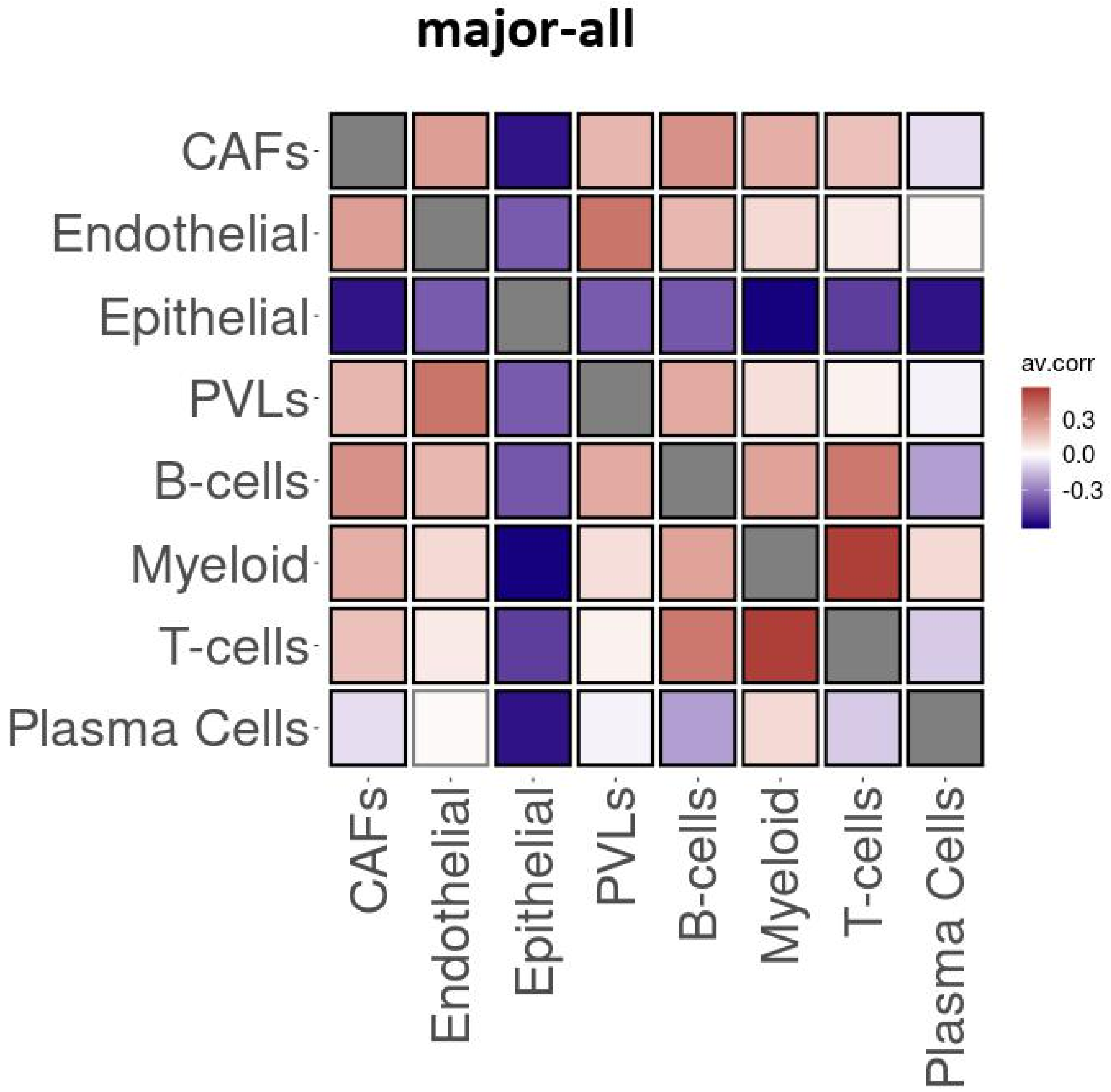
major-all
CAFs
Endothelial
Epithelial
PVLs
B-cells
Myeloid
T-cells
Plasma Cells
av.corr
0.3
0.0
-0.3

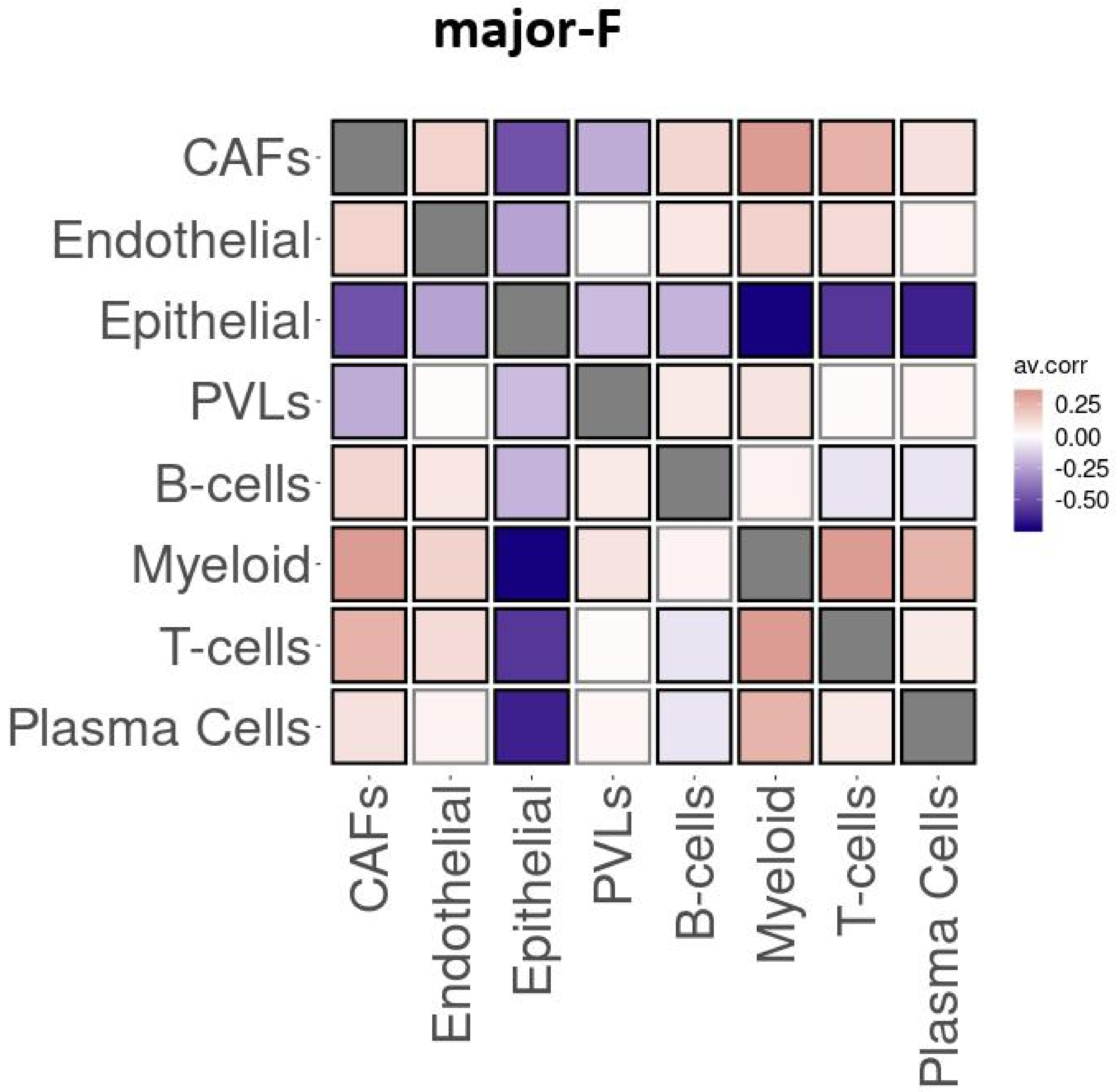

major-F
CAFs
Endothelial
Epithelial
PVLs
B-cells
Myeloid
T-cells
Plasma Cells
av.corr
0.25
0.00
-0.25
-0.50

minor-G

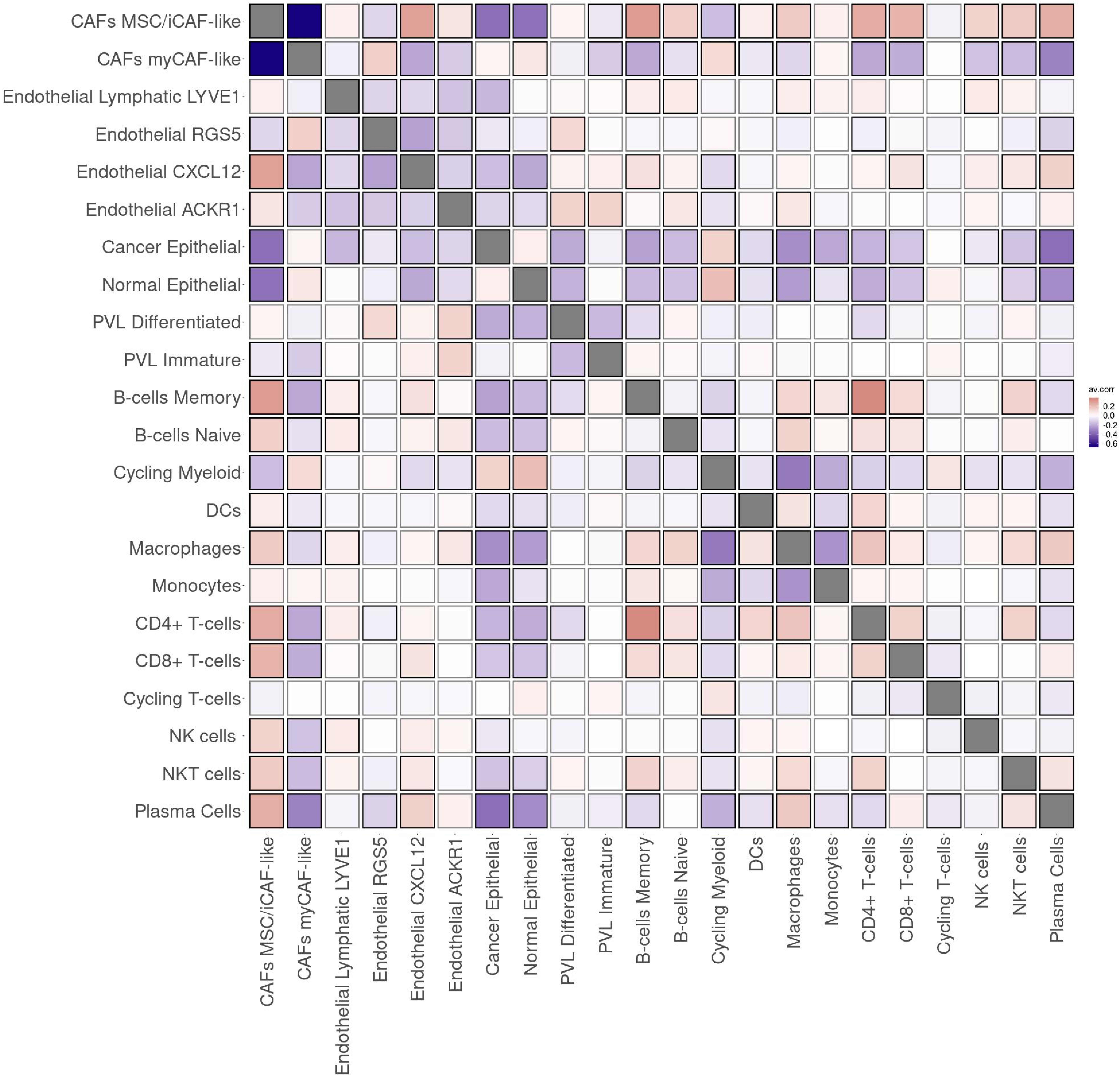

CAFs MSC/iCAF-like
CAFs myCAF-like
Endothelial Lymphatic LYVE1
Endothelial RGS5
Endothelial CXCL12
Endothelial ACKR1
Cancer Epithelial
Normal Epithelial
PVL Differentiated
PVL Immature
B-cells Memory
B-cells Naive
Cycling Myeloid
DCs
Macrophages
Monocytes
CD4+ T-cells
CD8+ T-cells
Cycling T-cells
NK cells
NKT cells
Plasma Cells
av.corr
0.2
0.0
-0.2
-0.4
-0.6

minor-B

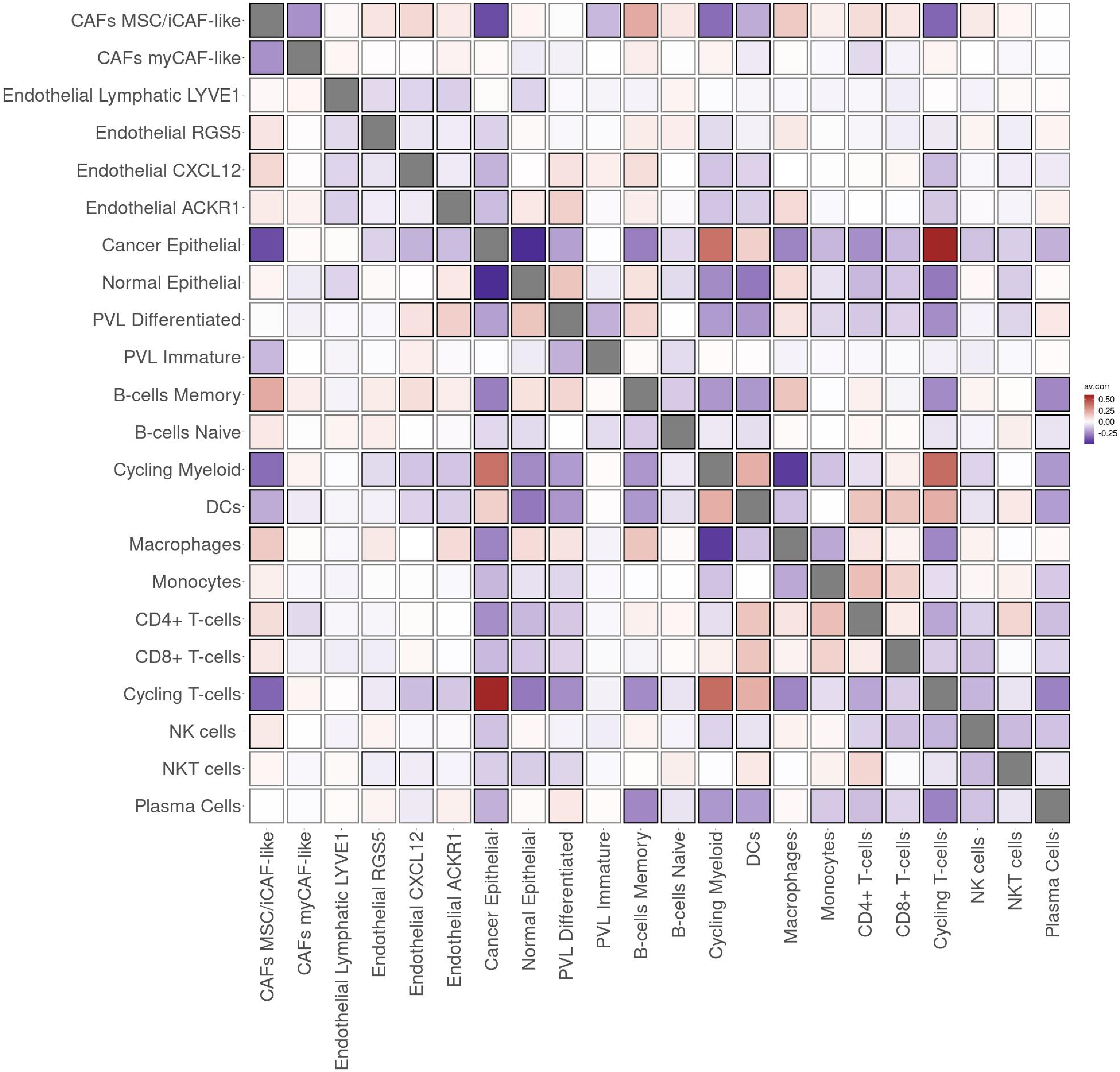
CAFs MSC/iCAF-like
CAFs myCAF-like
Endothelial Lymphatic LYVE1
Endothelial RGS5
Endothelial CXCL12
Endothelial ACKR1
Cancer Epithelial
Normal Epithelial
PVL Differentiated
PVL Immature
B-cells Memory
B-cells Naive
Cycling Myeloid
DCs
Macrophages
Monocytes
CD4+ T-cells
CD8+ T-cells
Cycling T-cells
NK cells
NKT cells
Plasma Cells
av.corr
0.50
0.25
0.00
-0.25

minor-t_cell-and-macro

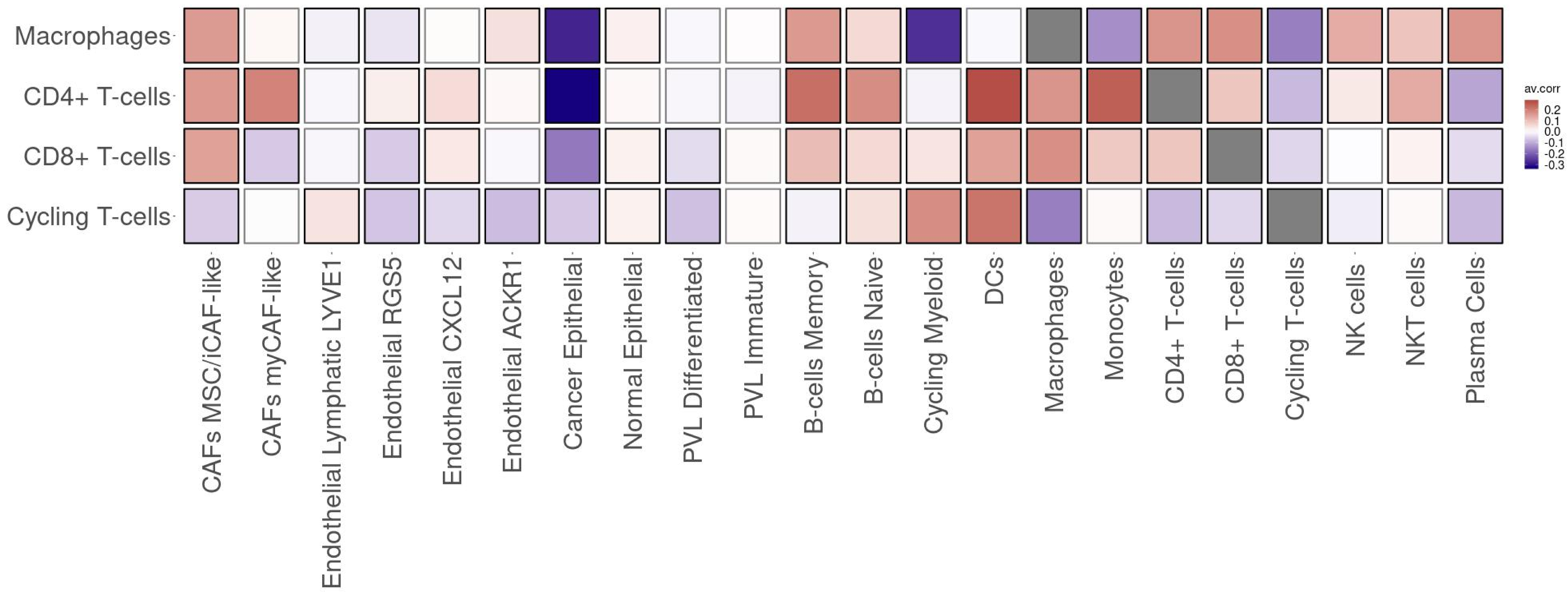

minor-all

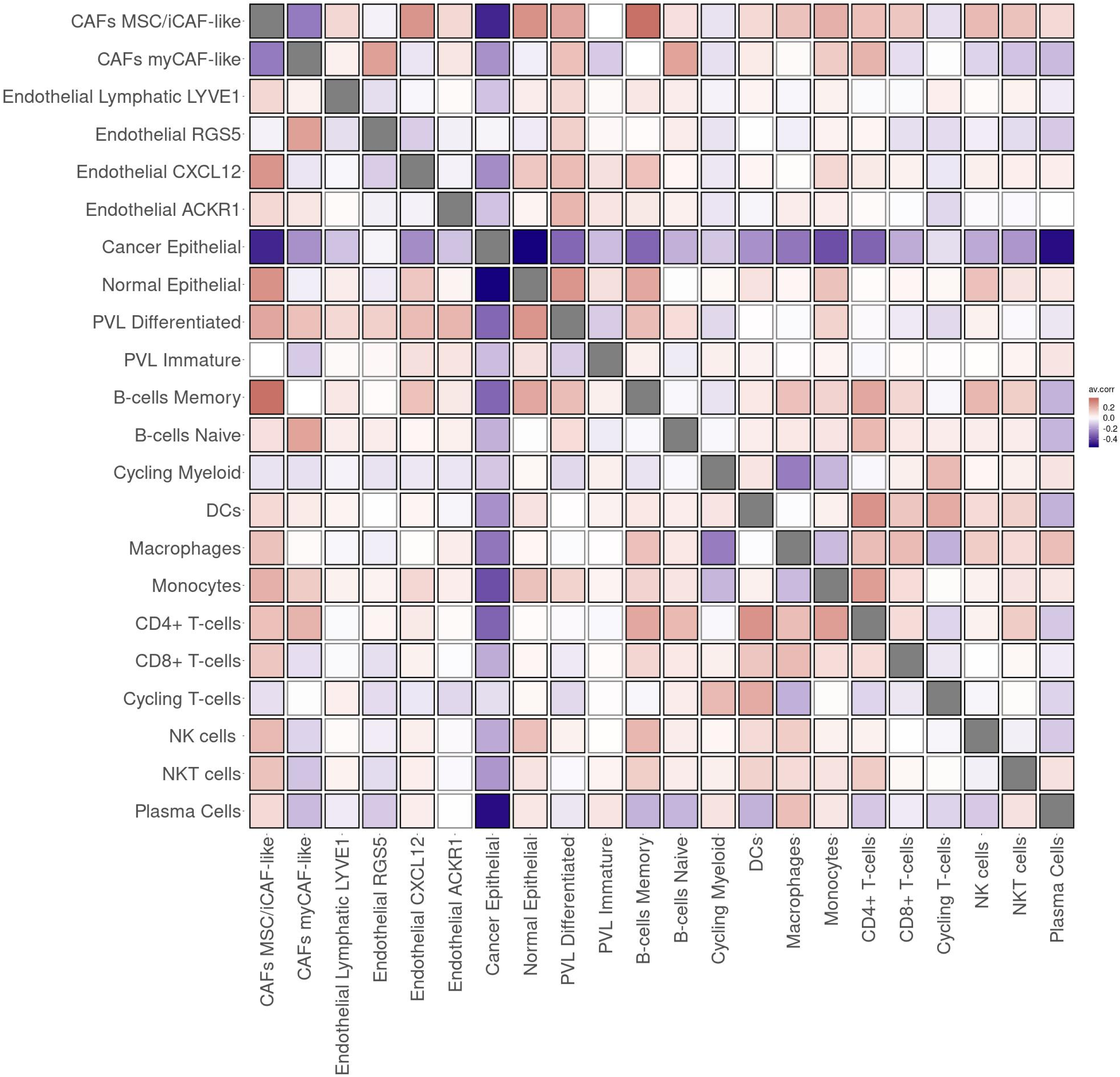
CAFs MSC/iCAF-like
CAFs myCAF-like
Endothelial Lymphatic LYVE1
Endothelial RGS5
Endothelial CXCL12
Endothelial ACKR1
Cancer Epithelial
Normal Epithelial
PVL Differentiated
PVL Immature
B-cells Memory
B-cells Naive
Cycling Myeloid
DCs
Macrophages
Monocytes
CD4+ T-cells
CD8+ T-cells
Cycling T-cells
NK cells
NKT cells
Plasma Cells
av.corr
0.2
0.0
-0.2
-0.4

minor-D

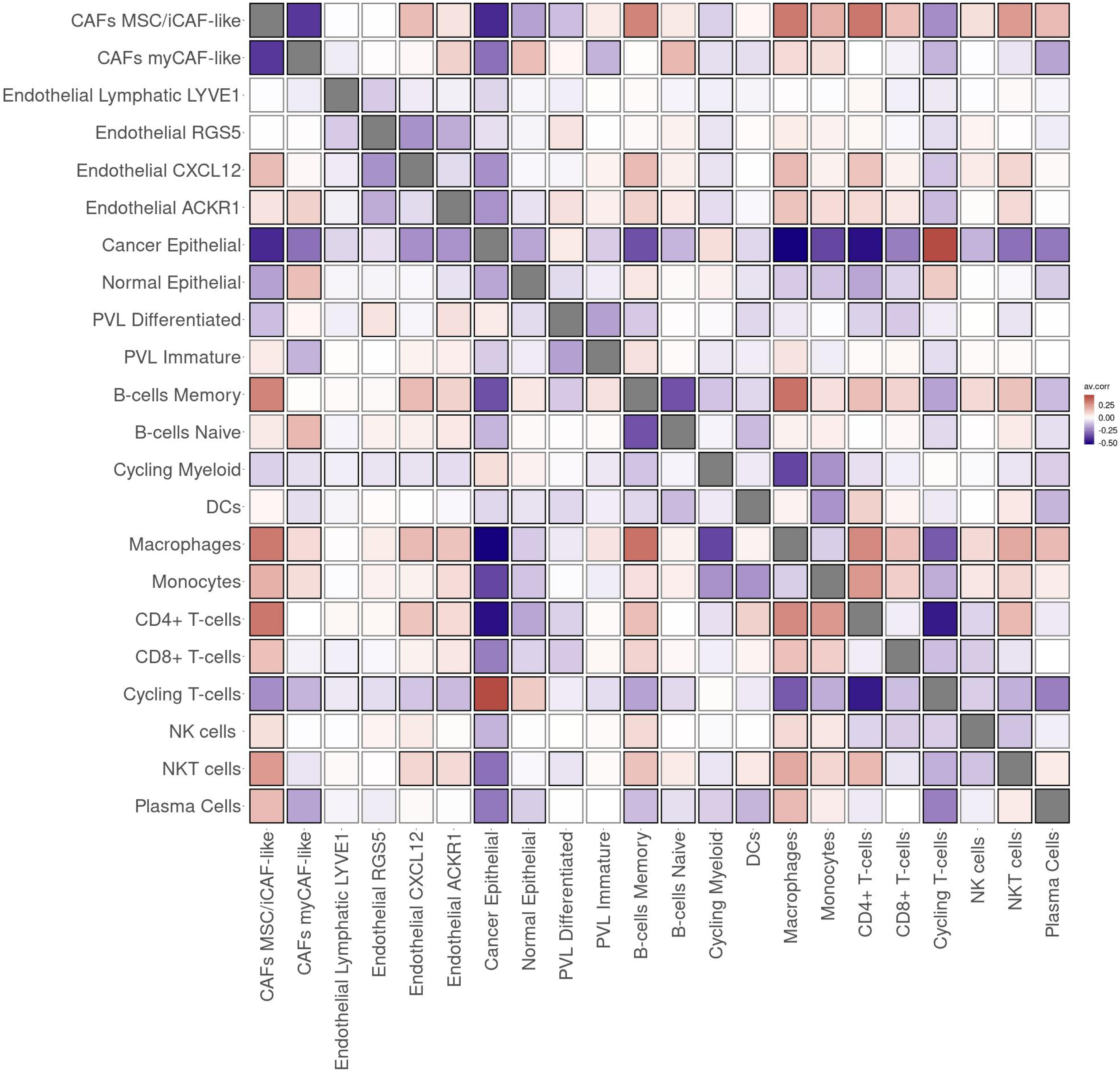

CAFs MSC/iCAF-like
CAFs myCAF-like
Endothelial Lymphatic LYVE1
Endothelial RGS5
Endothelial CXCL12
Endothelial ACKR1
Cancer Epithelial
Normal Epithelial
PVL Differentiated
PVL Immature
B-cells Memory
B-cells Naive
Cycling Myeloid
DCs
Macrophages
Monocytes
CD4+ T-cells
CD8+ T-cells
Cycling T-cells
NK cells
NKT cells
Plasma Cells
av.corr
0.25
0.00
-0.25
-0.50

minor-E

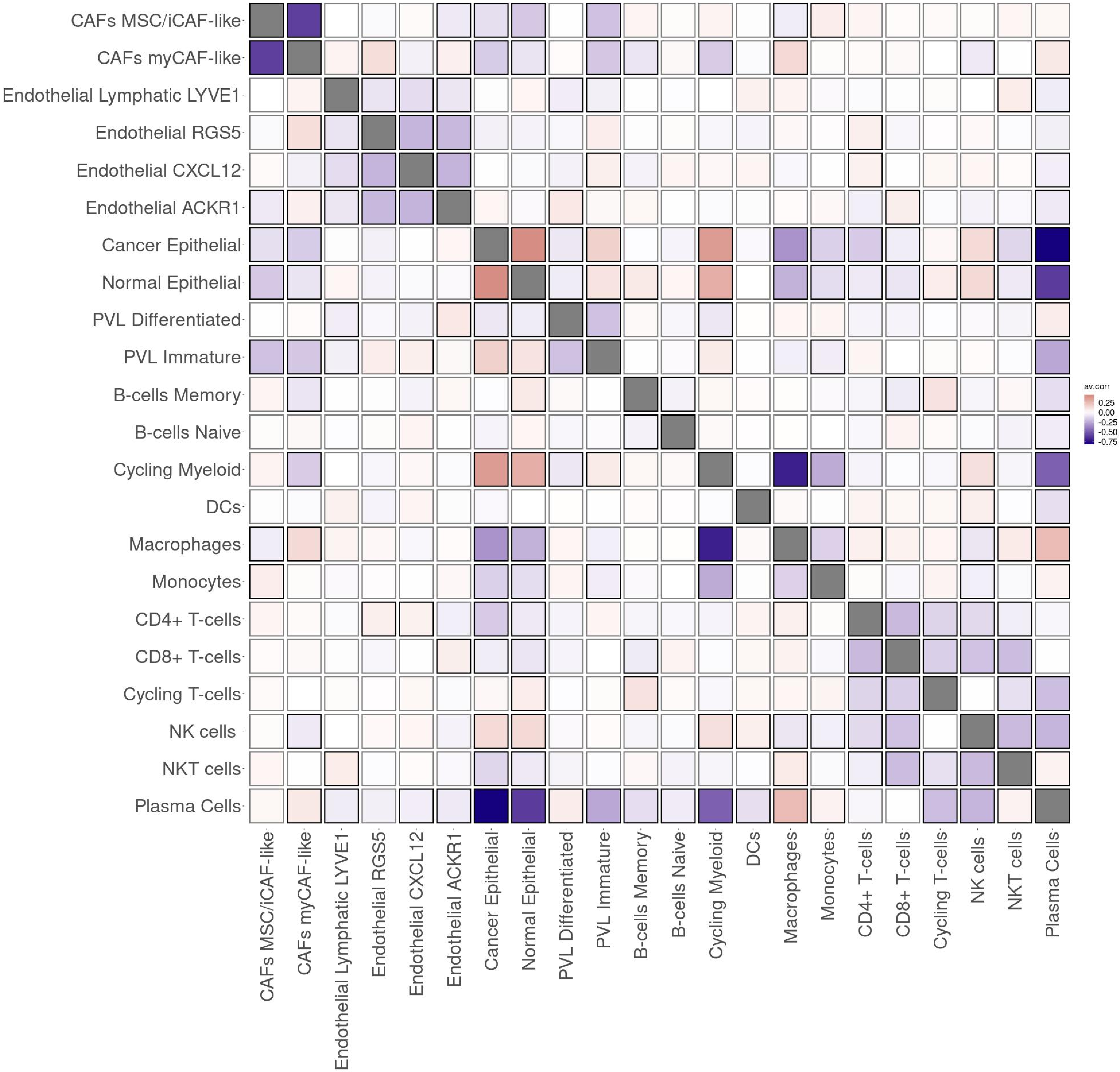

CAFs MSC/iCAF-like
CAFs myCAF-like
Endothelial Lymphatic LYVE1
Endothelial RGS5
Endothelial CXCL12
Endothelial ACKR1
Cancer Epithelial
Normal Epithelial
PVL Differentiated
PVL Immature
B-cells Memory
B-cells Naive
Cycling Myeloid
DCs
Macrophages
Monocytes
CD4+ T-cells
CD8+ T-cells
Cycling T-cells
NK cells
NKT cells
Plasma Cells
av.corr
0.25
0.00
-0.25
-0.50
-0.75

minor-F

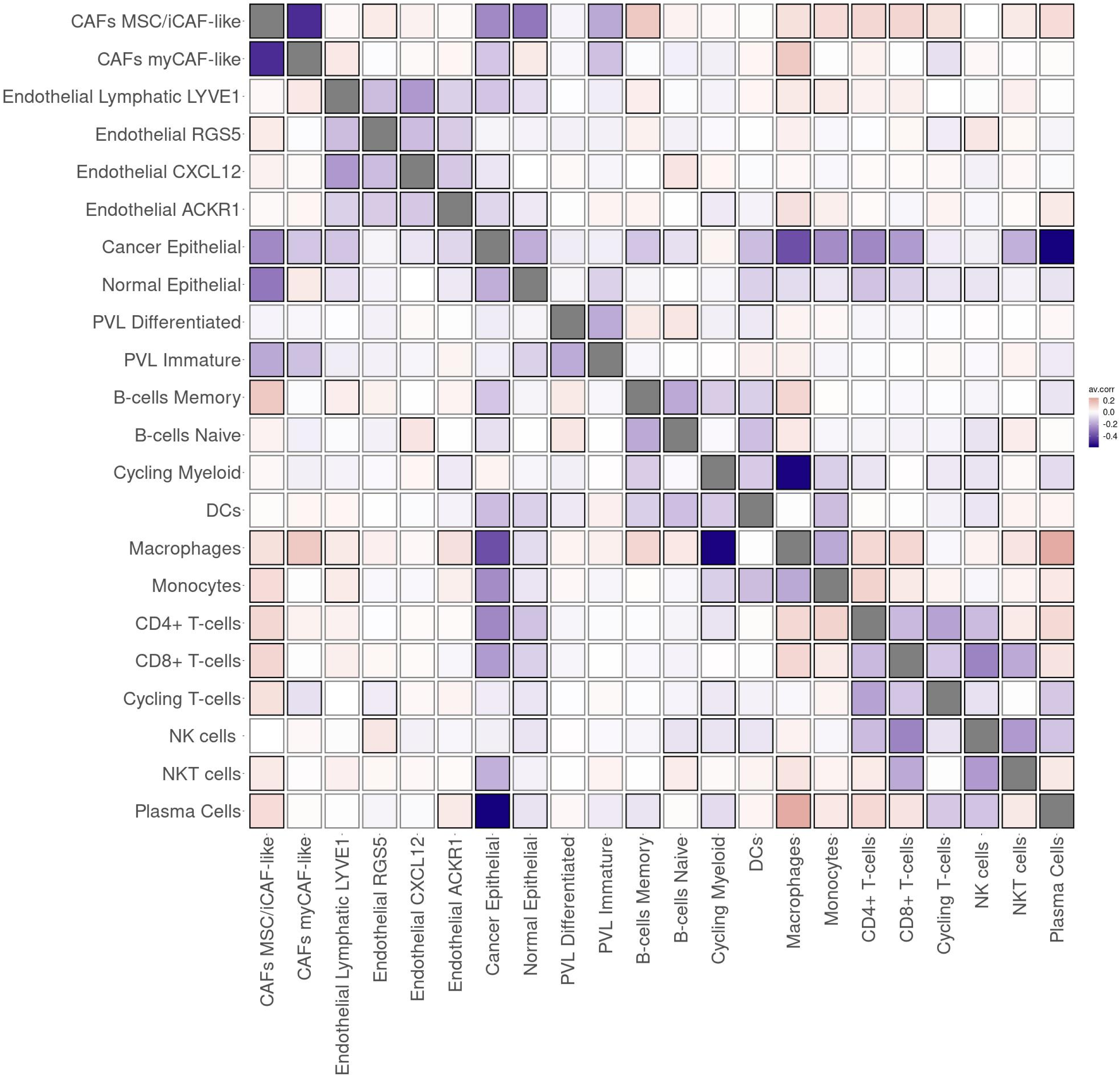
CAFs MSC/iCAF-like
CAFs myCAF-like
Endothelial Lymphatic LYVE1
Endothelial RGS5
Endothelial CXCL12
Endothelial ACKR1
Cancer Epithelial
Normal Epithelial
PVL Differentiated
PVL Immature
B-cells Memory
B-cells Naive
Cycling Myeloid
DCs
Macrophages
Monocytes
CD4+ T-cells
CD8+ T-cells
Cycling T-cells
NK cells
NKT cells
Plasma Cells
av.corr
0.2
0.0
-0.2
-0.4

minor-A

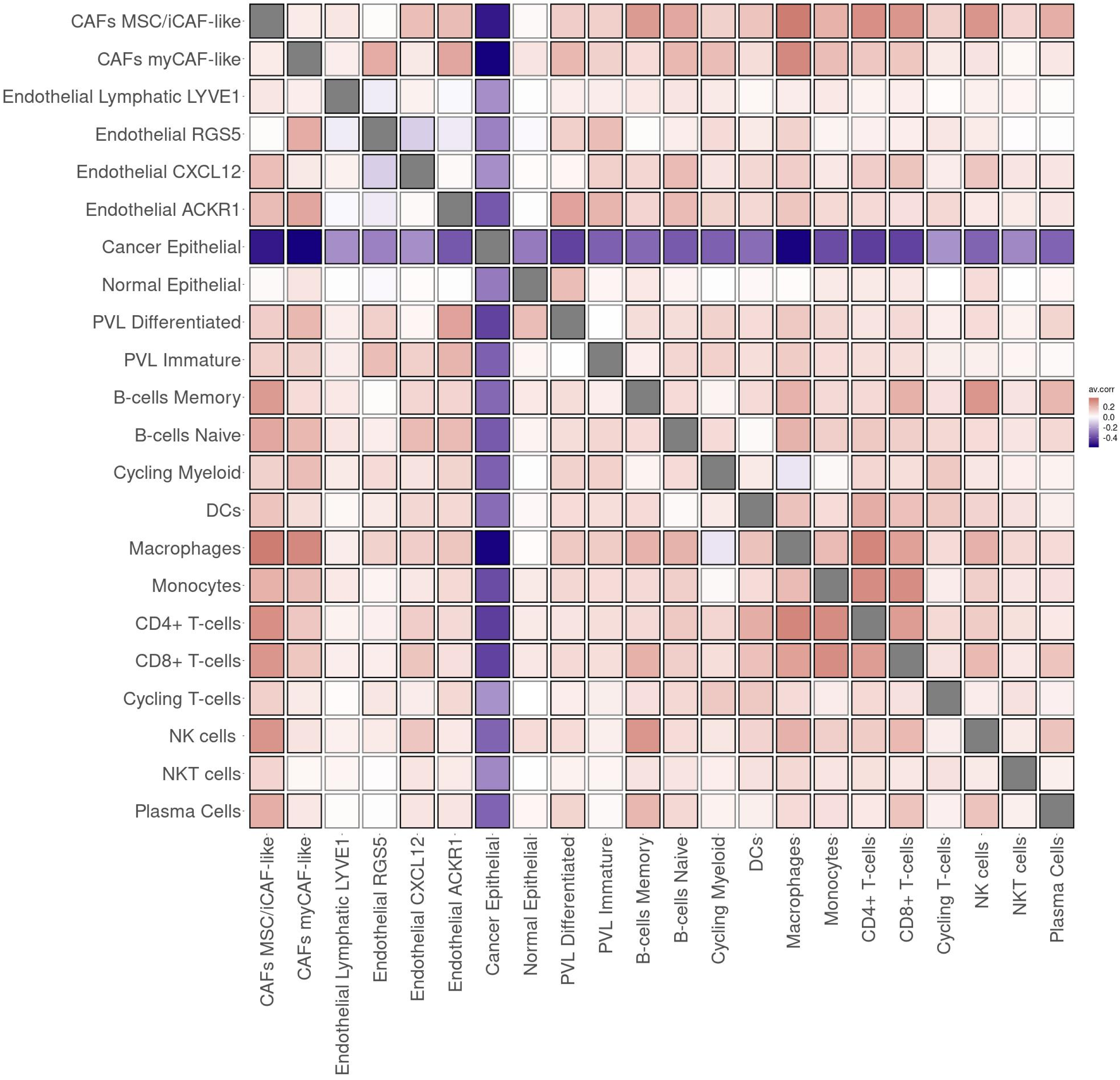
CAFs MSC/iCAF-like
CAFs myCAF-like
Endothelial Lymphatic LYVE1
Endothelial RGS5
Endothelial CXCL12
Endothelial ACKR1
Cancer Epithelial
Normal Epithelial
PVL Differentiated
PVL Immature
B-cells Memory
B-cells Naive
Cycling Myeloid
DCs
Macrophages
Monocytes
CD4+ T-cells
CD8+ T-cells
Cycling T-cells
NK cells
NKT cells
Plasma Cells
av.corr
0.2
0.0
-0.2
-0.4

minor-H

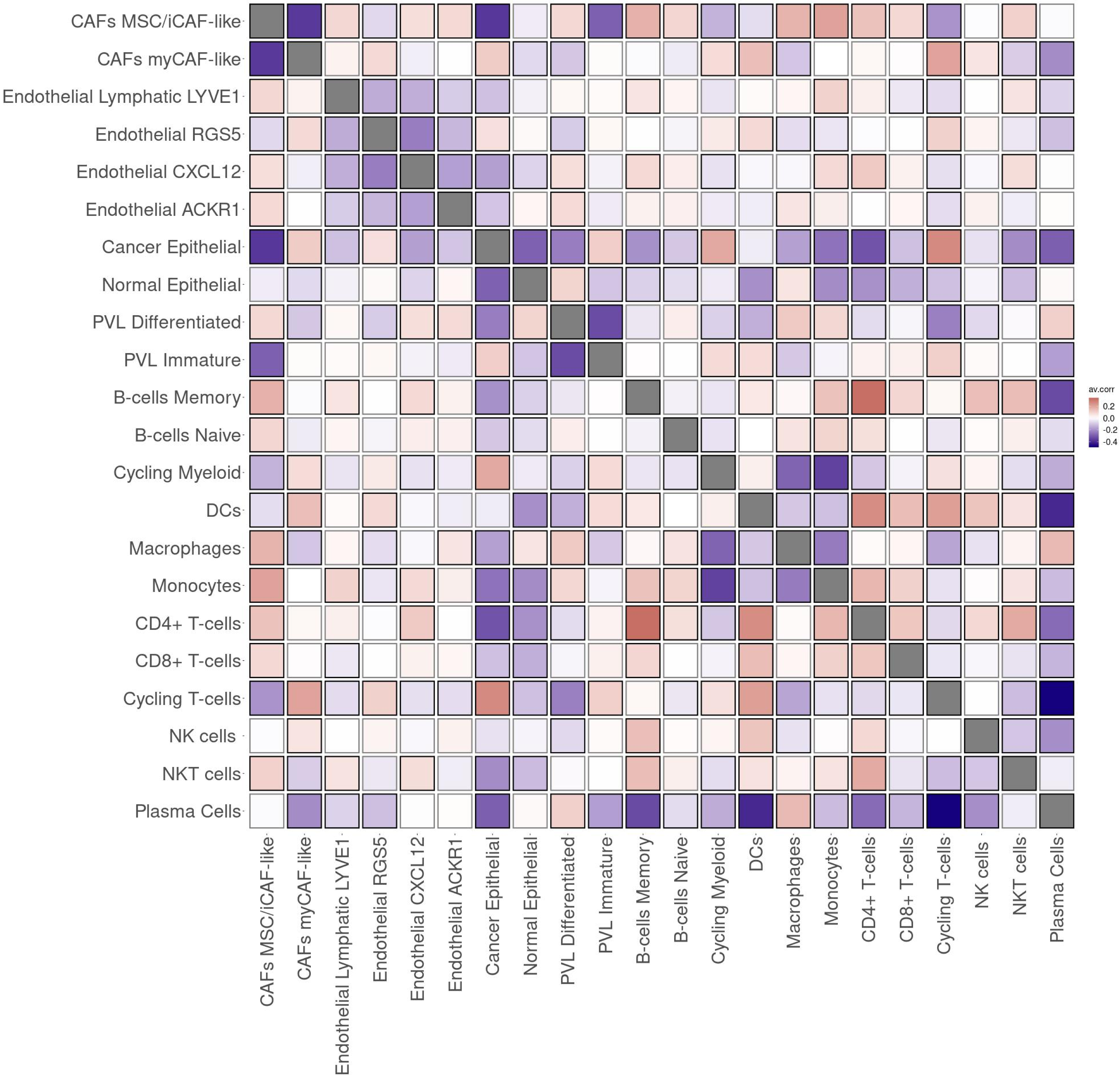
CAFs MSC/iCAF-like
CAFs myCAF-like
Endothelial Lymphatic LYVE1
Endothelial RGS5
Endothelial CXCL12
Endothelial ACKR1
Cancer Epithelial
Normal Epithelial
PVL Differentiated
PVL Immature
B-cells Memory
B-cells Naive
Cycling Myeloid
DCs
Macrophages
Monocytes
CD4+ T-cells
CD8+ T-cells
Cycling T-cells
NK cells
NKT cells
Plasma Cells
av.corr
0.2
0.0
-0.2
-0.4

minor-C

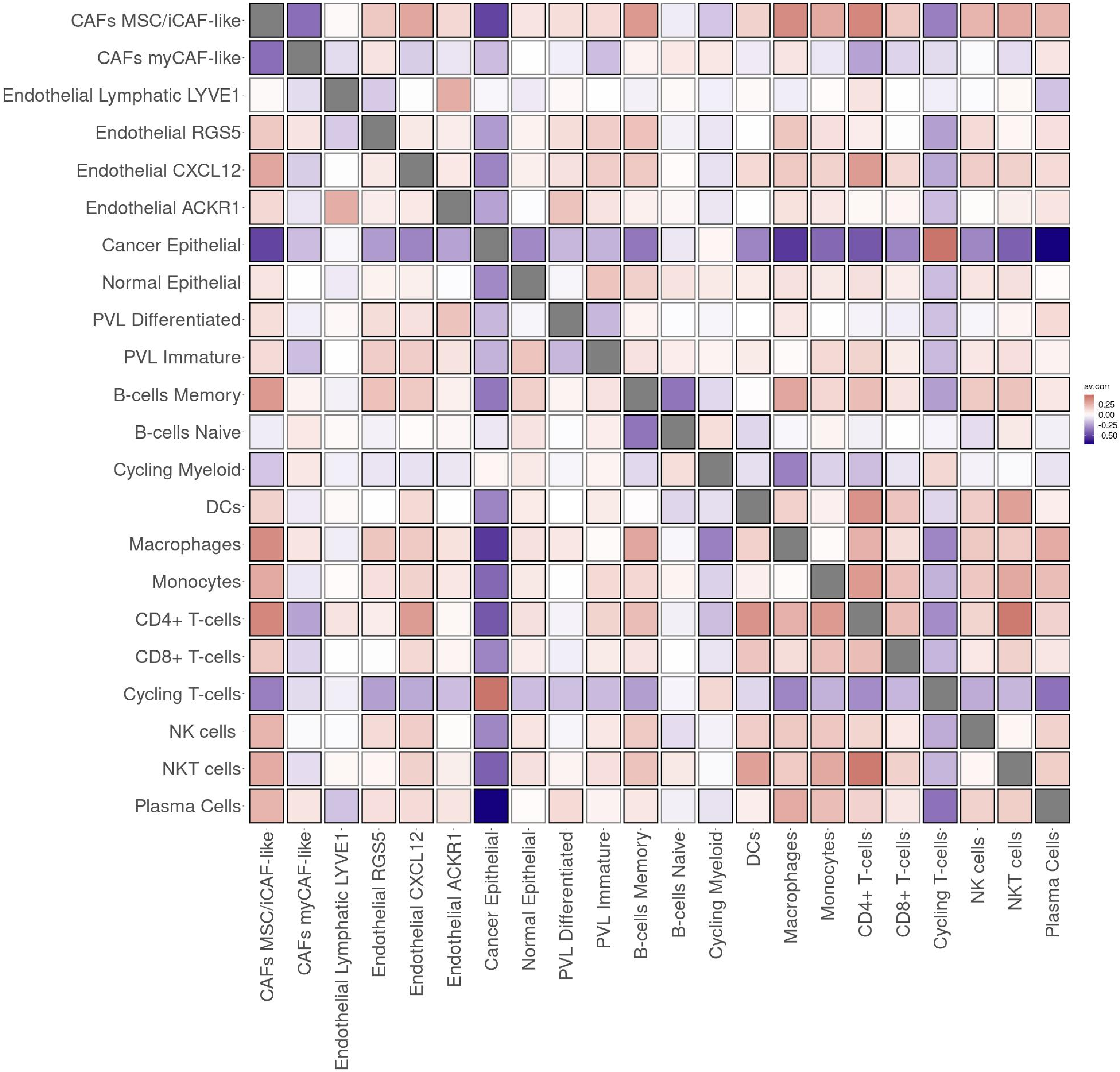
CAFs MSC/iCAF-like
CAFs myCAF-like
Endothelial Lymphatic LYVE1
Endothelial RGS5
Endothelial CXCL12
Endothelial ACKR1
Cancer Epithelial
Normal Epithelial
PVL Differentiated
PVL Immature
B-cells Memory
B-cells Naive
Cycling Myeloid
DCs
Macrophages
Monocytes
CD4+ T-cells
CD8+ T-cells
Cycling T-cells
NK cells
NKT cells
Plasma Cells
av.corr
0.25
0.00
-0.25
-0.50

subset-A

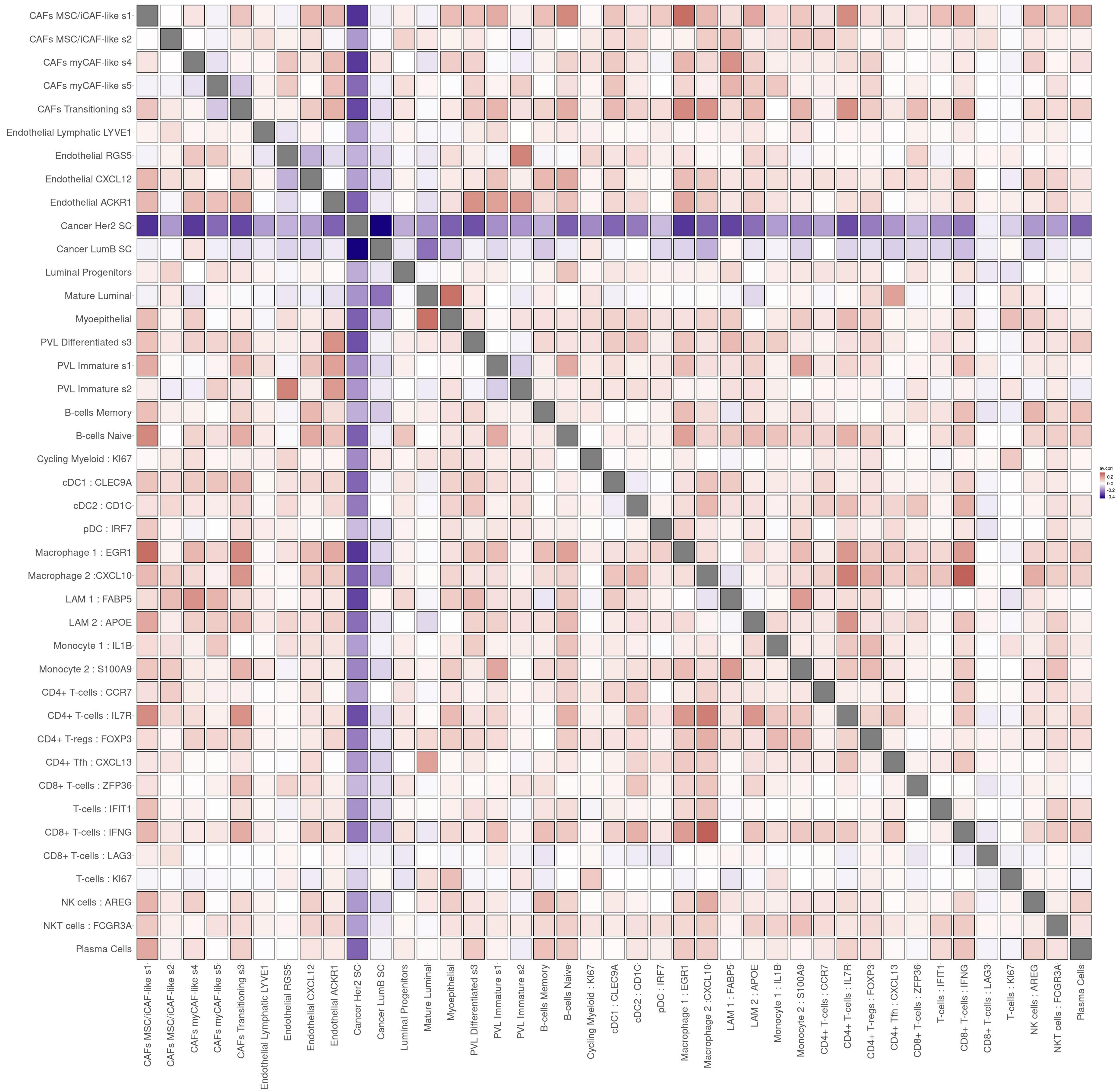
CAFs MSC/iCAF-like s1
CAFs MSC/iCAF-like s2
CAFs myCAF-like s4
CAFs myCAF-like s5
CAFs Transitioning s3
Endothelial Lymphatic LYVE1
Endothelial RGS5
Endothelial CXCL12
Endothelial ACKR1
Cancer Her2 SC
Cancer LumB SC
Luminal Progenitors
Mature Luminal
Myoepithelial
PVL Differentiated s3
PVL Immature s1
PVL Immature s2
B-cells Memory
B-cells Naive
Cycling Myeloid : KI67
cDC1 : CLEC9A
cDC2 : CD1C
pDC : IRF7
Macrophage 1 : EGR1
Macrophage 2 :CXCL10
LAM 1 : FABP5
LAM 2 : APOE
Monocyte 1 : IL1B
Monocyte 2 : S100A9
CD4+ T-cells : CCR7
CD4+ T-cells : IL7R
CD4+ T-regs : FOXP3
CD4+ Tfh : CXCL13
CD8+ T-cells : ZFP36
T-cells : IFIT1
CD8+ T-cells : IFNG
CD8+ T-cells : LAG3
T-cells : KI67
NK cells : AREG
NKT cells : FCGR3A
Plasma Cells
CAFs MSC/iCAF-like s1
CAFs MSC/iCAF-like s2
CAFs myCAF-like s4
CAFs myCAF-like s5
CAFs Transitioning s3
Endothelial Lymphatic LYVE1
Endothelial RGS5
Endothelial CXCL12
Endothelial ACKR1
Cancer Her2 SC
Cancer LumB SC
Luminal Progenitors
Mature Luminal
Myoepithelial
PVL Differentiated s3
PVL Immature s1
PVL Immature s2
B-cells Memory
B-cells Naive
Cycling Myeloid : KI67
cDC1 : CLEC9A
cDC2 : CD1C
pDC : IRF7
Macrophage 1 : EGR1
Macrophage 2 :CXCL10
LAM 1 : FABP5
LAM 2 : APOE
Monocyte 1 : IL1B
Monocyte 2 : S100A9
CD4+ T-cells : CCR7
CD4+ T-cells : IL7R
CD4+ T-regs : FOXP3
CD4+ Tfh : CXCL13
CD8+ T-cells : ZFP36
T-cells : IFIT1
CD8+ T-cells : IFNG
CD8+ T-cells : LAG3
T-cells : KI67
NK cells : AREG
NKT cells : FCGR3A
Plasma Cells
av.corr
0.2
0.0
-0.2
-0.4

subset-H

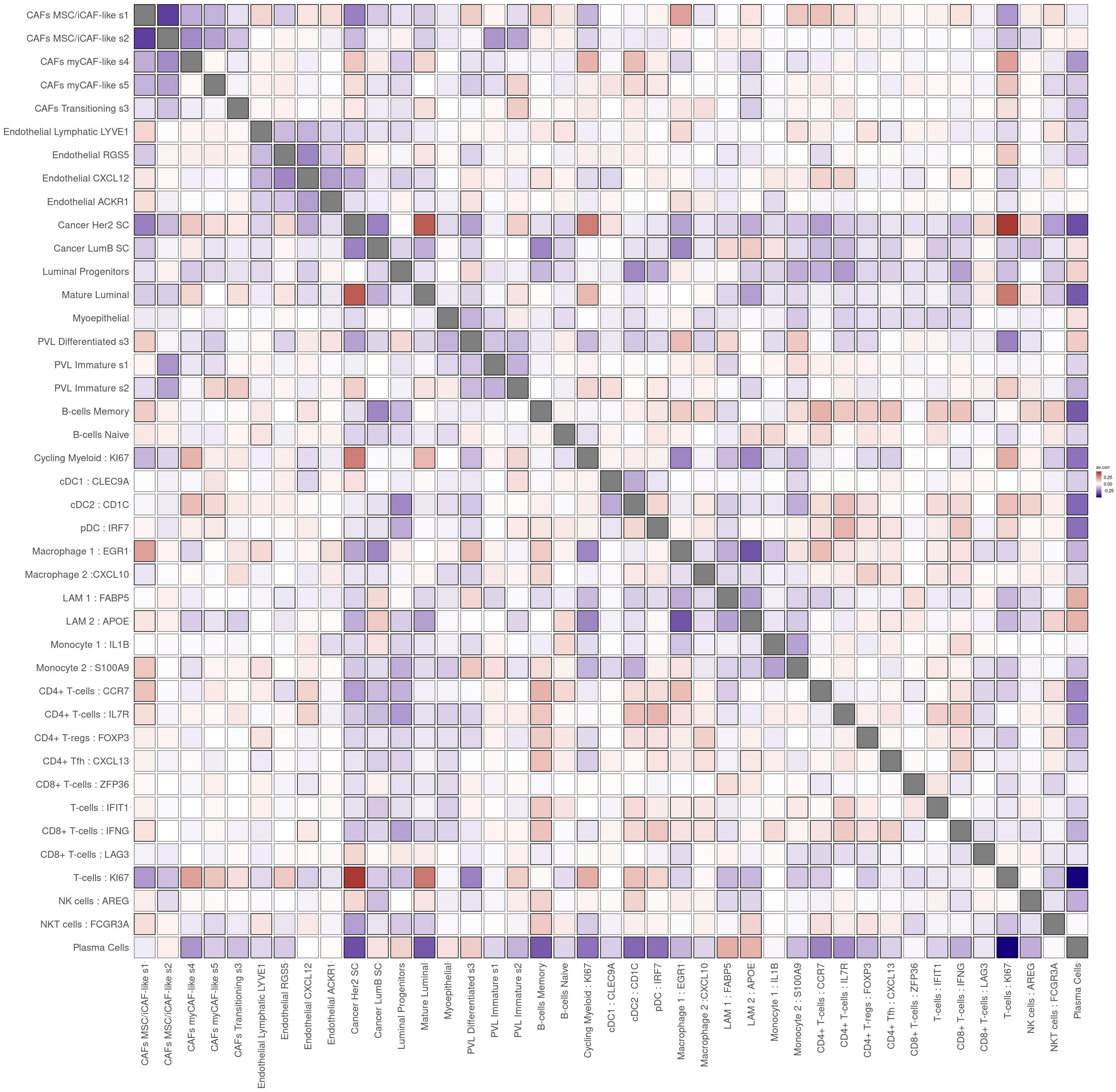

CAFs MSC/iCAF-like s1
CAFs MSC/iCAF-like s2
CAFs myCAF-like s4
CAFs myCAF-like s5
CAFs Transitioning s3
Endothelial Lymphatic LYVE1
Endothelial RGS5
Endothelial CXCL12
Endothelial ACKR1
Cancer Her2 SC
Cancer LumB SC
Luminal Progenitors
Mature Luminal
Myoepithelial
PVL Differentiated s3
PVL Immature s1
PVL Immature s2
B-cells Memory
B-cells Naive
Cycling Myeloid : Kl67
cDC1 : CLEC9A
cDC2 : CD1C
pDC : IRF7
Macrophage 1 : EGR1
Macrophage 2 :CXCL10
LAM 1 : FABP5
LAM 2 : APOE
Monocyte 1 : IL1B
Monocyte 2 : S100A9
CD4+ T-cells : CCR7
CD4+ T-cells : IL7R
CD4+ T-regs : FOXP3
CD4+ Tfh : CXCL13
CD8+ T-cells : ZFP36
T-cells : IFIT1
CD8+ T-cells : IFNG
CD8+ T-cells : LAG3
T-cells : Kl67
NK cells : AREG
NKT cells : FCGR3A
Plasma Cells
av.corr
0.25
0.00
-0.25

subset-all-macro

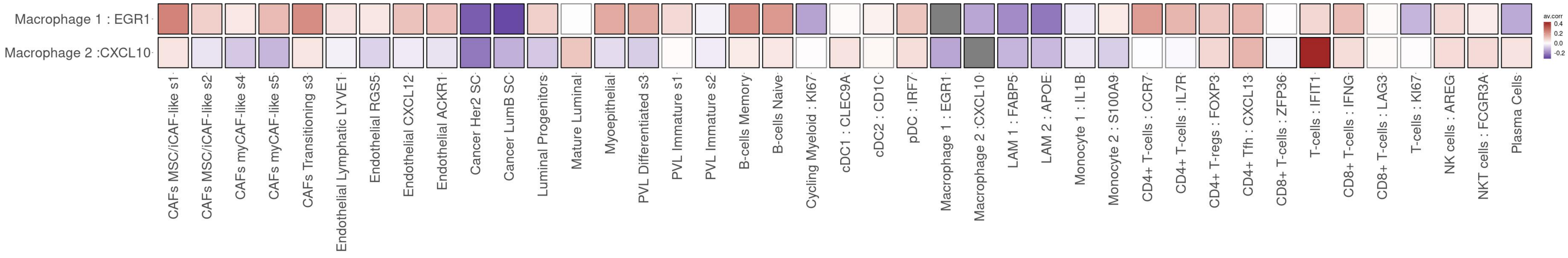

subset-C

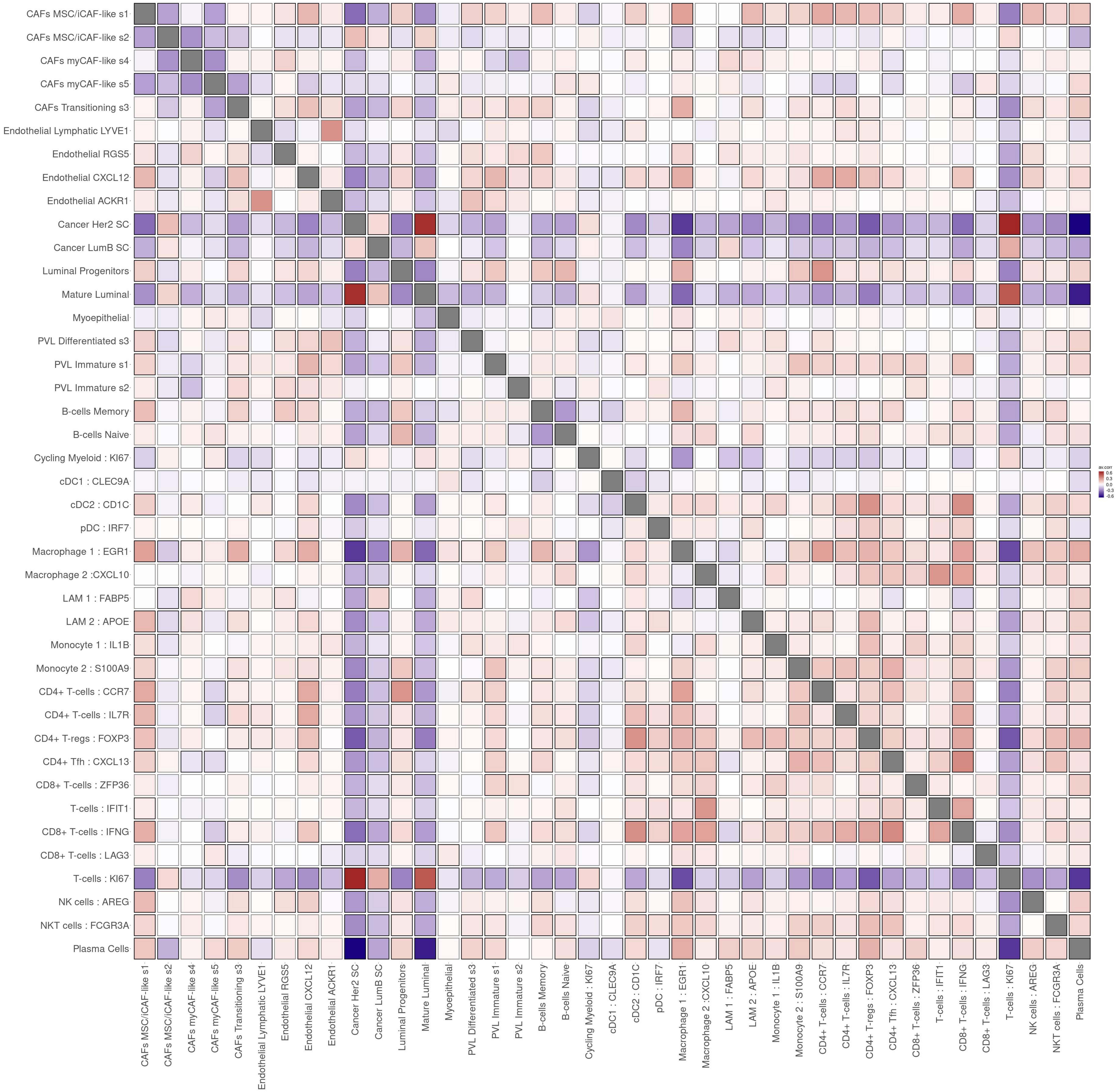
CAFs MSC/iCAF-like s1
CAFs MSC/iCAF-like s2
CAFs myCAF-like s4
CAFs myCAF-like s5
CAFs Transitioning s3
Endothelial Lymphatic LYVE1
Endothelial RGS5
Endothelial CXCL12
Endothelial ACKR1
Cancer Her2 SC
Cancer LumB SC
Luminal Progenitors
Mature Luminal
Myoepithelial
PVL Differentiated s3
PVL Immature s1
PVL Immature s2
B-cells Memory
B-cells Naive
Cycling Myeloid : KI67
cDC1 : CLEC9A
cDC2 : CD1C
pDC : IRF7
Macrophage 1 : EGR1
Macrophage 2 :CXCL10
LAM 1 : FABP5
LAM 2 : APOE
Monocyte 1 : IL1B
Monocyte 2 : S100A9
CD4+ T-cells : CCR7
CD4+ T-cells : IL7R
CD4+ T-regs : FOXP3
CD4+ Tfh : CXCL13
CD8+ T-cells : ZFP36
T-cells : IFIT1
CD8+ T-cells : IFNG
CD8+ T-cells : LAG3
T-cells : KI67
NK cells : AREG
NKT cells : FCGR3A
Plasma Cells
av.corr
0.6
0.3
0.0
-0.3
-0.6

subset-E

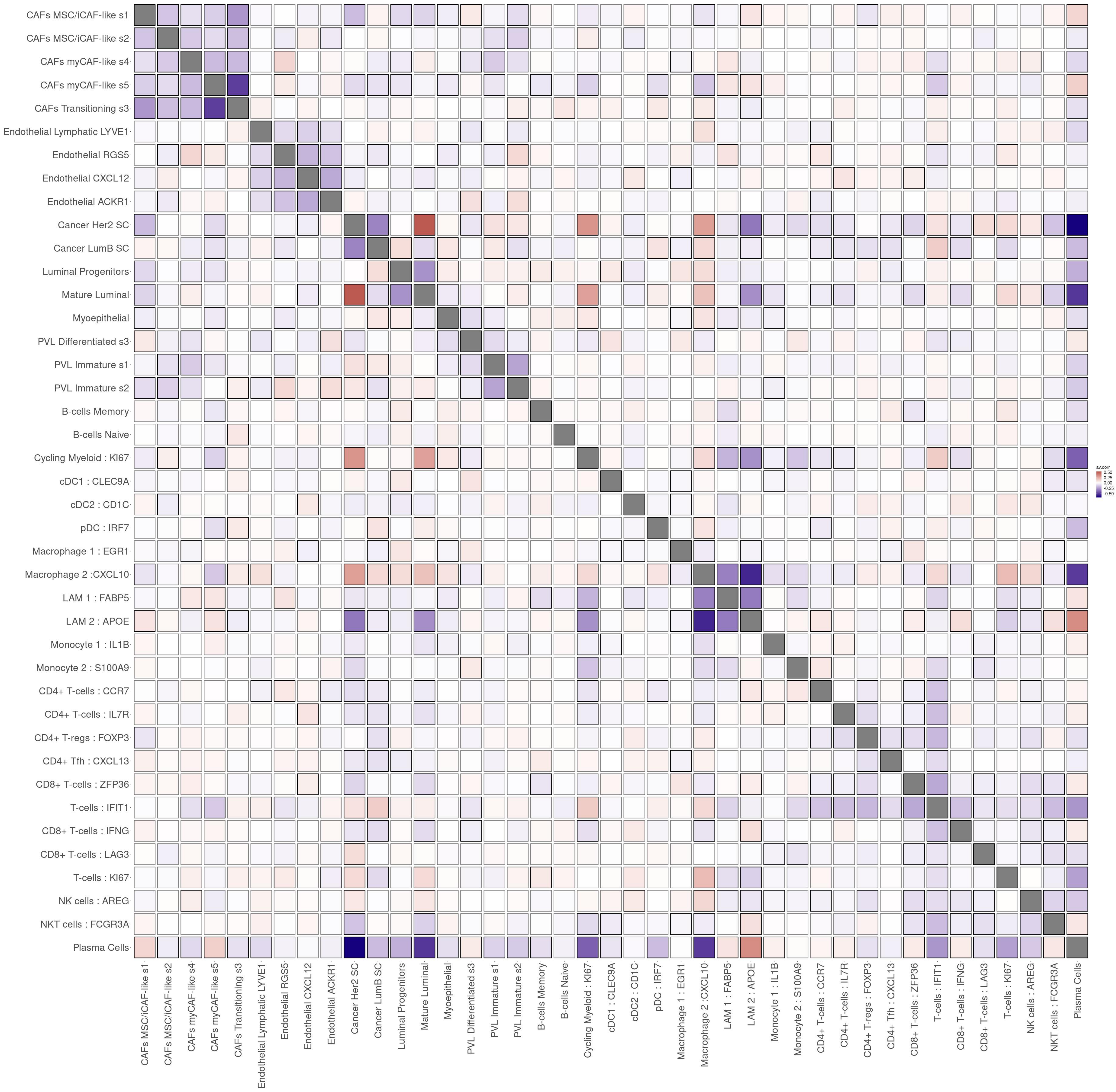
CAFs MSC/iCAF-like s1
CAFs MSC/iCAF-like s2
CAFs myCAF-like s4
CAFs myCAF-like s5
CAFs Transitioning s3
Endothelial Lymphatic LYVE1
Endothelial RGS5
Endothelial CXCL12
Endothelial ACKR1
Cancer Her2 SC
Cancer LumB SC
Luminal Progenitors
Mature Luminal
Myoepithelial
PVL Differentiated s3
PVL Immature s1
PVL Immature s2
B-cells Memory
B-cells Naive
Cycling Myeloid : KI67
cDC1 : CLEC9A
cDC2 : CD1C
pDC : IRF7
Macrophage 1 : EGR1
Macrophage 2 :CXCL10
LAM 1 : FABP5
LAM 2 : APOE
Monocyte 1 : IL1B
Monocyte 2 : S100A9
CD4+ T-cells : CCR7
CD4+ T-cells : IL7R
CD4+ T-regs : FOXP3
CD4+ Tfh : CXCL13
CD8+ T-cells : ZFP36
T-cells : IFIT1
CD8+ T-cells : IFNG
CD8+ T-cells : LAG3
T-cells : KI67
NK cells : AREG
NKT cells : FCGR3A
Plasma Cells
av.corr
0.50
0.25
0.00
-0.25
-0.50

subset-all-t_cell

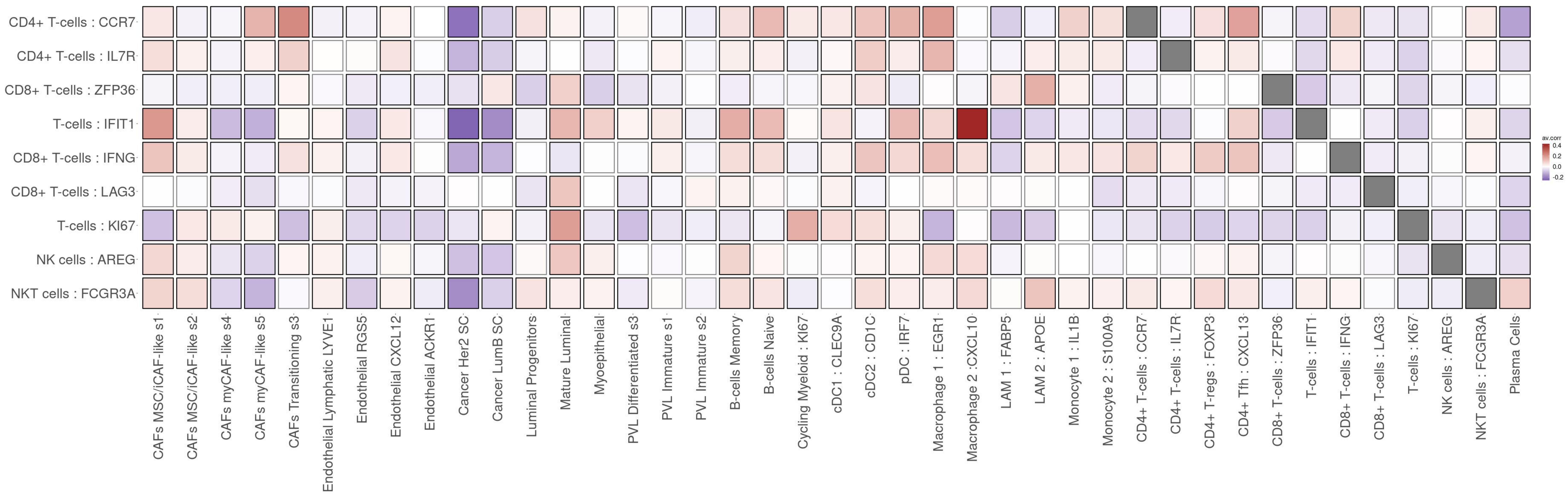

subset-B

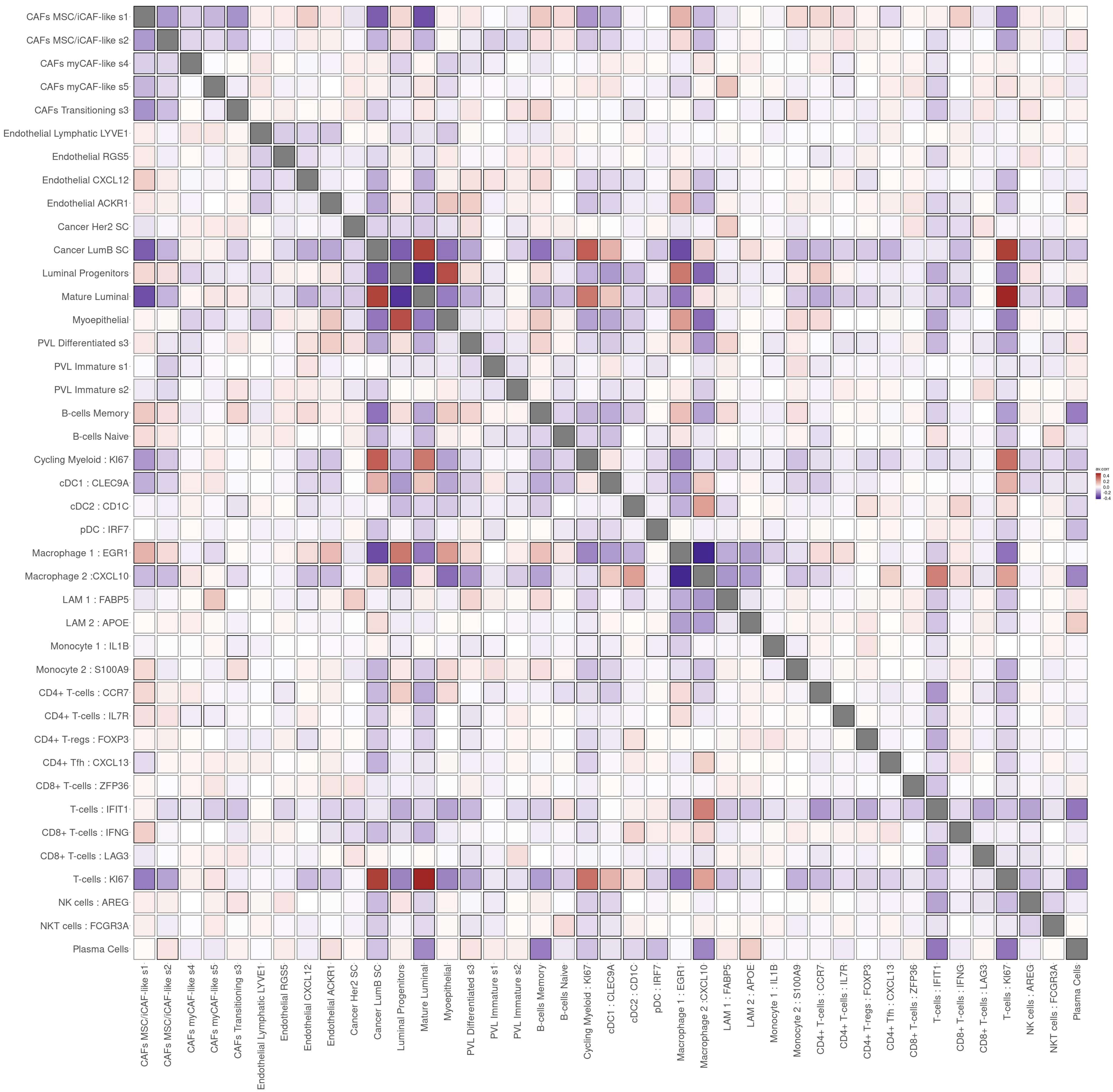
CAFs MSC/iCAF-like s1
CAFs MSC/iCAF-like s2
CAFs myCAF-like s4
CAFs myCAF-like s5
CAFs Transitioning s3
Endothelial Lymphatic LYVE1
Endothelial RGS5
Endothelial CXCL12
Endothelial ACKR1
Cancer Her2 SC
Cancer LumB SC
Luminal Progenitors
Mature Luminal
Myoepithelial
PVL Differentiated s3
PVL Immature s1
PVL Immature s2
B-cells Memory
B-cells Naive
Cycling Myeloid : KI67
cDC1 : CLEC9A
cDC2 : CD1C
pDC : IRF7
Macrophage 1 : EGR1
Macrophage 2 :CXCL10
LAM 1 : FABP5
LAM 2 : APOE
Monocyte 1 : IL1B
Monocyte 2 : S100A9
CD4+ T-cells : CCR7
CD4+ T-cells : IL7R
CD4+ T-regs : FOXP3
CD4+ Tfh : CXCL13
CD8+ T-cells : ZFP36
T-cells : IFIT1
CD8+ T-cells : IFNG
CD8+ T-cells : LAG3
T-cells : KI67
NK cells : AREG
NKT cells : FCGR3A
Plasma Cells
av.corr
0.4
0.2
0.0
-0.2
-0.4

subset-G

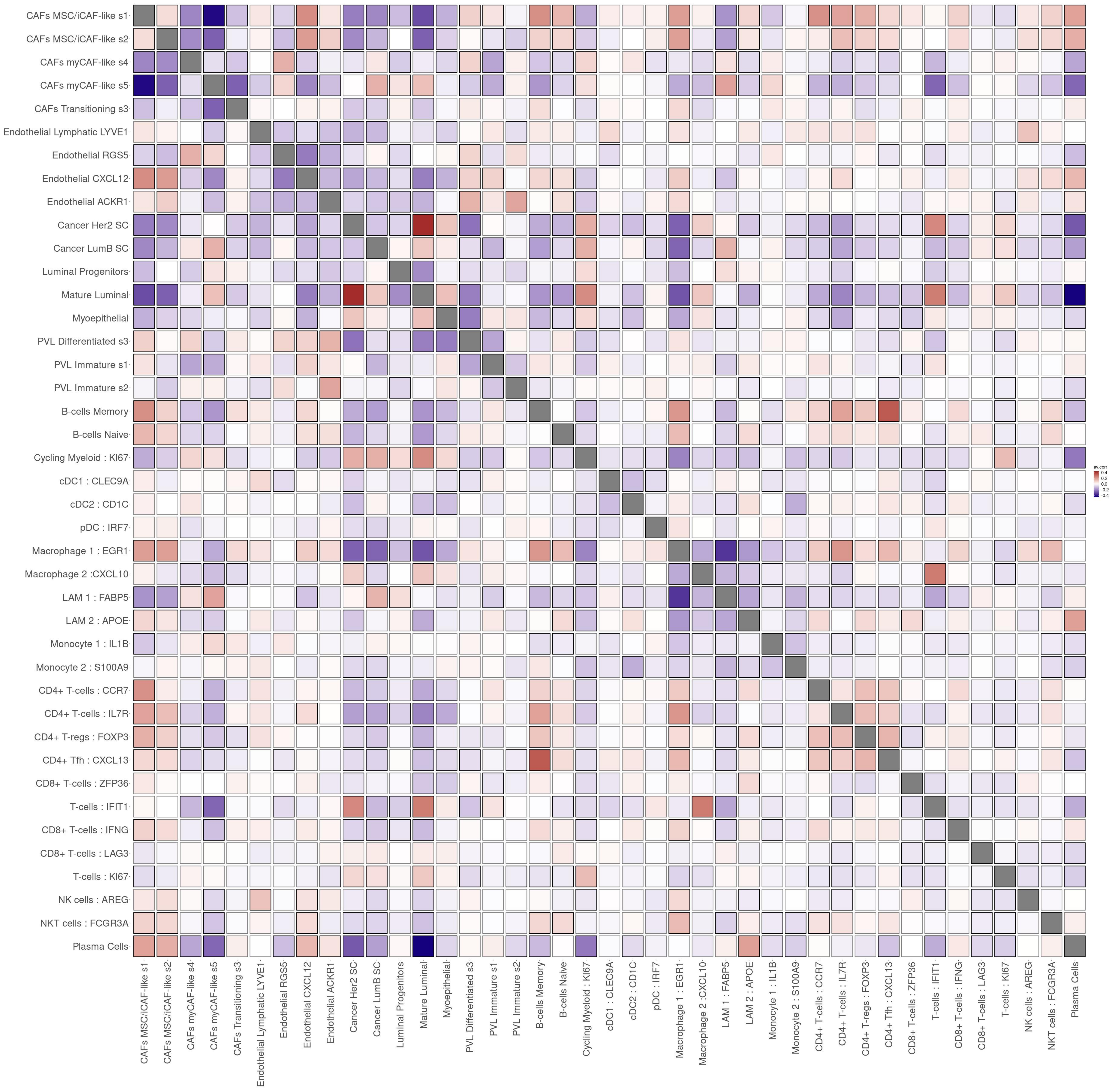

CAFs MSC/iCAF-like s1
CAFs MSC/iCAF-like s2
CAFs myCAF-like s4
CAFs myCAF-like s5
CAFs Transitioning s3
Endothelial Lymphatic LYVE1
Endothelial RGS5
Endothelial CXCL12
Endothelial ACKR1
Cancer Her2 SC
Cancer LumB SC
Luminal Progenitors
Mature Luminal
Myoepithelial
PVL Differentiated s3
PVL Immature s1
PVL Immature s2
B-cells Memory
B-cells Naive
Cycling Myeloid : KI67
cDC1 : CLEC9A
cDC2 : CD1C
pDC : IRF7
Macrophage 1 : EGR1
Macrophage 2 :CXCL10
LAM 1 : FABP5
LAM 2 : APOE
Monocyte 1 : IL1B
Monocyte 2 : S100A9
CD4+ T-cells : CCR7
CD4+ T-cells : IL7R
CD4+ T-regs : FOXP3
CD4+ Tfh : CXCL13
CD8+ T-cells : ZFP36
T-cells : IFIT1
CD8+ T-cells : IFNG
CD8+ T-cells : LAG3
T-cells : KI67
NK cells : AREG
NKT cells : FCGR3A
Plasma Cells
av.corr
0.4
0.2
0.0
-0.2
-0.4

subset-D

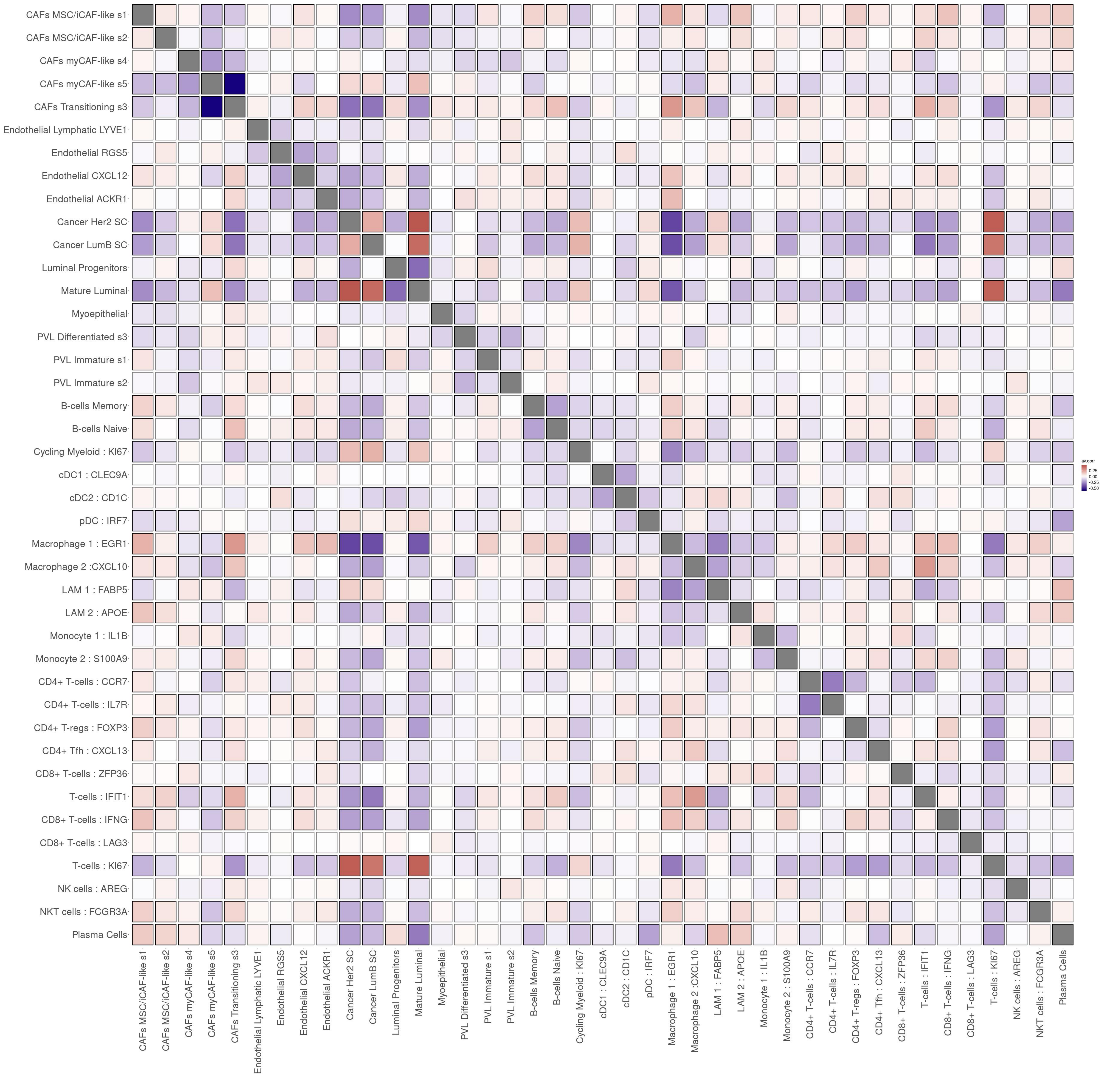
CAFs MSC/iCAF-like s1
CAFs MSC/iCAF-like s2
CAFs myCAF-like s4
CAFs myCAF-like s5
CAFs Transitioning s3
Endothelial Lymphatic LYVE1
Endothelial RGS5
Endothelial CXCL12
Endothelial ACKR1
Cancer Her2 SC
Cancer LumB SC
Luminal Progenitors
Mature Luminal
Myoepithelial
PVL Differentiated s3
PVL Immature s1
PVL Immature s2
B-cells Memory
B-cells Naive
Cycling Myeloid : KI67
cDC1 : CLEC9A
cDC2 : CD1C
pDC : IRF7
Macrophage 1 : EGR1
Macrophage 2 :CXCL10
LAM 1 : FABP5
LAM 2 : APOE
Monocyte 1 : IL1B
Monocyte 2 : S100A9
CD4+ T-cells : CCR7
CD4+ T-cells : IL7R
CD4+ T-regs : FOXP3
CD4+ Tfh : CXCL13
CD8+ T-cells : ZFP36
T-cells : IFIT1
CD8+ T-cells : IFNG
CD8+ T-cells : LAG3
T-cells : KI67
NK cells : AREG
NKT cells : FCGR3A
Plasma Cells
CAFs MSC/iCAF-like s1
CAFs MSC/iCAF-like s2
CAFs myCAF-like s4
CAFs myCAF-like s5
CAFs Transitioning s3
Endothelial Lymphatic LYVE1
Endothelial RGS5
Endothelial CXCL12
Endothelial ACKR1
Cancer Her2 SC
Cancer LumB SC
Luminal Progenitors
Mature Luminal
Myoepithelial
PVL Differentiated s3
PVL Immature s1
PVL Immature s2
B-cells Memory
B-cells Naive
Cycling Myeloid : KI67
cDC1 : CLEC9A
cDC2 : CD1C
pDC : IRF7
Macrophage 1 : EGR1
Macrophage 2 :CXCL10
LAM 1 : FABP5
LAM 2 : APOE
Monocyte 1 : IL1B
Monocyte 2 : S100A9
CD4+ T-cells : CCR7
CD4+ T-cells : IL7R
CD4+ T-regs : FOXP3
CD4+ Tfh : CXCL13
CD8+ T-cells : ZFP36
T-cells : IFIT1
CD8+ T-cells : IFNG
CD8+ T-cells : LAG3
T-cells : KI67
NK cells : AREG
NKT cells : FCGR3A
Plasma Cells
av.corr
0.25
0.00
-0.25
-0.50

subset-all

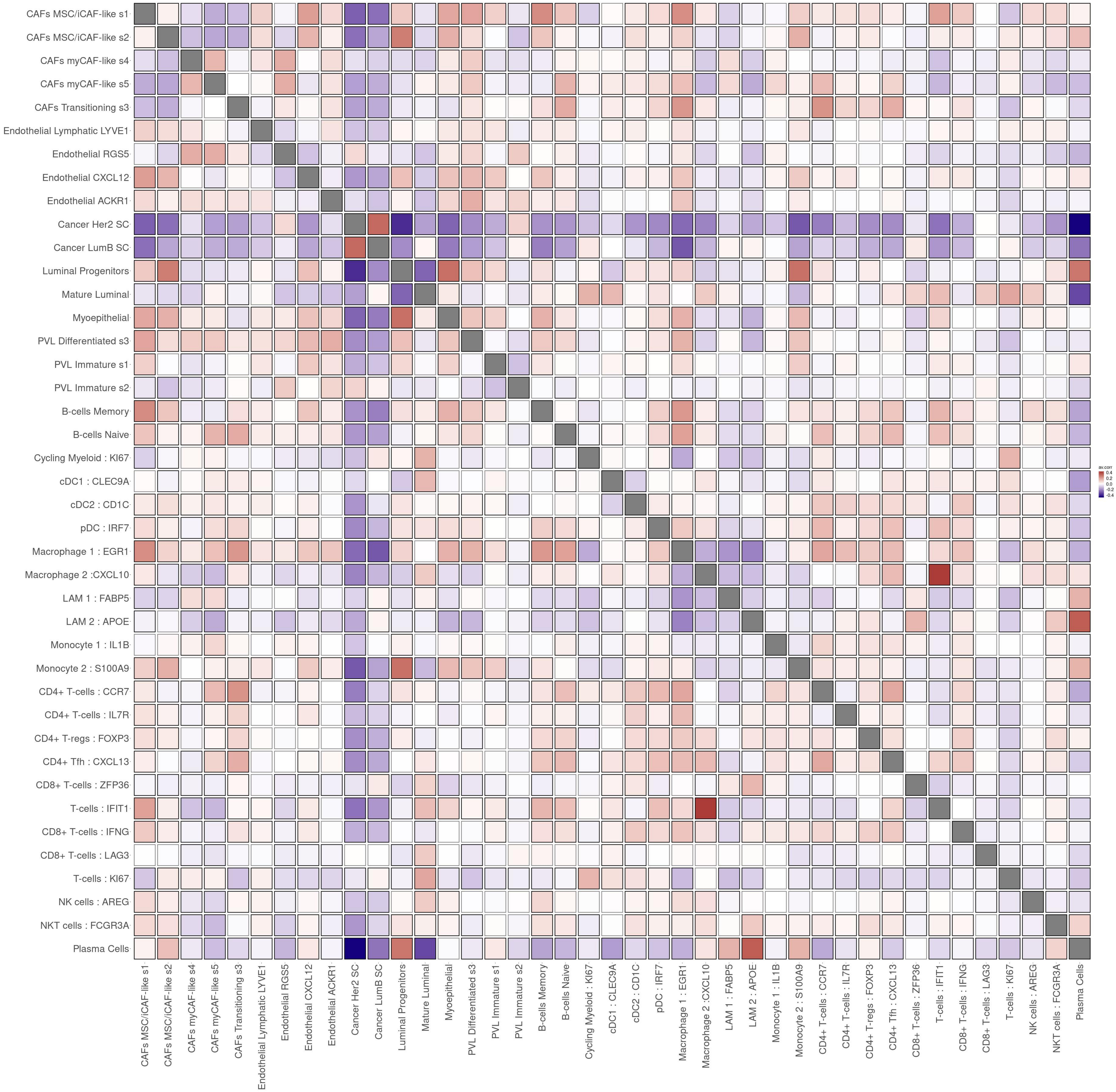
CAFs MSC/iCAF-like s1
CAFs MSC/iCAF-like s2
CAFs myCAF-like s4
CAFs myCAF-like s5
CAFs Transitioning s3
Endothelial Lymphatic LYVE1
Endothelial RGS5
Endothelial CXCL12
Endothelial ACKR1
Cancer Her2 SC
Cancer LumB SC
Luminal Progenitors
Mature Luminal
Myoepithelial
PVL Differentiated s3
PVL Immature s1
PVL Immature s2
B-cells Memory
B-cells Naive
Cycling Myeloid : KI67
cDC1 : CLEC9A
cDC2 : CD1C
pDC : IRF7
Macrophage 1 : EGR1
Macrophage 2 :CXCL10
LAM 1 : FABP5
LAM 2 : APOE
Monocyte 1 : IL1B
Monocyte 2 : S100A9
CD4+ T-cells : CCR7
CD4+ T-cells : IL7R
CD4+ T-regs : FOXP3
CD4+ Tfh : CXCL13
CD8+ T-cells : ZFP36
T-cells : IFIT1
CD8+ T-cells : IFNG
CD8+ T-cells : LAG3
T-cells : KI67
NK cells : AREG
NKT cells : FCGR3A
Plasma Cells
av.corr
0.4
0.2
0.0
-0.2
-0.4

subset-F

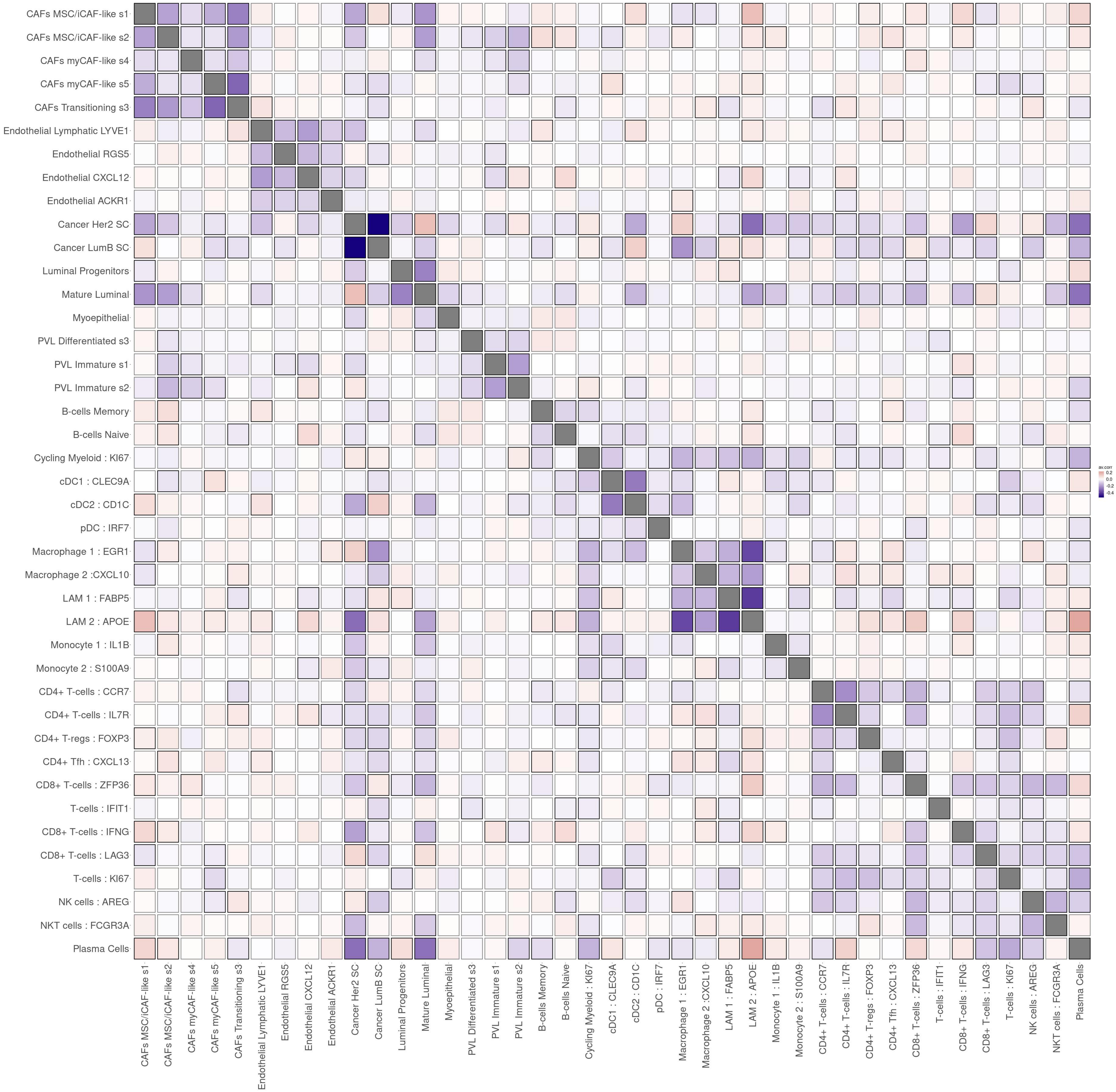

CAFs MSC/iCAF-like s1
CAFs MSC/iCAF-like s2
CAFs myCAF-like s4
CAFs myCAF-like s5
CAFs Transitioning s3
Endothelial Lymphatic LYVE1
Endothelial RGS5
Endothelial CXCL12
Endothelial ACKR1
Cancer Her2 SC
Cancer LumB SC
Luminal Progenitors
Mature Luminal
Myoepithelial
PVL Differentiated s3
PVL Immature s1
PVL Immature s2
B-cells Memory
B-cells Naive
Cycling Myeloid : KI67
cDC1 : CLEC9A
cDC2 : CD1C
pDC : IRF7
Macrophage 1 : EGR1
Macrophage 2 :CXCL10
LAM 1 : FABP5
LAM 2 : APOE
Monocyte 1 : IL1B
Monocyte 2 : S100A9
CD4+ T-cells : CCR7
CD4+ T-cells : IL7R
CD4+ T-regs : FOXP3
CD4+ Tfh : CXCL13
CD8+ T-cells : ZFP36
T-cells : IFIT1
CD8+ T-cells : IFNG
CD8+ T-cells : LAG3
T-cells : KI67
NK cells : AREG
NKT cells : FCGR3A
Plasma Cells
av.corr
0.2
0.0
-0.2
-0.4
