# Supplementary material for: Spatial deconvolution of HER2-positive breast cancer delineates tumor-associated cell type interactions
Source: Nat Commun. 2021 Oct 14;12:6012. doi: 10.1038/s41467-021-26271-2 (PMC8516894; doi:10.1038/s41467-021-26271-2)
Supplement: Supplementary file 17 — Reporting Summary [file 41467_2021_26271_MOESM17_ESM.pdf]

nature research

Corresponding author(s): Alma Andersson & Joakim Lundeberg

Last updated by author(s): Sep 16, 2021

Reporting Summary

Nature Research wishes to improve the reproducibility of the work that we publish. This form provides structure for consistency and transparency in reporting. For further information on Nature Research policies, see our Editorial Policies and the Editorial Policy Checklist.

Statistics

For all statistical analyses, confirm that the following items are present in the figure legend, table legend, main text, or Methods section.

n/a	Confirmed	
[ ]	[x]	The exact sample size (*n*) for each experimental group/condition, given as a discrete number and unit of measurement
[x]	[ ]	A statement on whether measurements were taken from distinct samples or whether the same sample was measured repeatedly
[ ]	[x]	The statistical test(s) used AND whether they are one- or two-sided<br>*Only common tests should be described solely by name; describe more complex techniques in the Methods section.*
[x]	[ ]	A description of all covariates tested
[ ]	[x]	A description of any assumptions or corrections, such as tests of normality and adjustment for multiple comparisons
[ ]	[x]	A full description of the statistical parameters including central tendency (e.g. means) or other basic estimates (e.g. regression coefficient) AND variation (e.g. standard deviation) or associated estimates of uncertainty (e.g. confidence intervals)
[ ]	[x]	For null hypothesis testing, the test statistic (e.g. *F*, *t*, *r*) with confidence intervals, effect sizes, degrees of freedom and *P* value noted<br>*Give P values as exact values whenever suitable.*
[x]	[ ]	For Bayesian analysis, information on the choice of priors and Markov chain Monte Carlo settings
[x]	[ ]	For hierarchical and complex designs, identification of the appropriate level for tests and full reporting of outcomes
[ ]	[x]	Estimates of effect sizes (e.g. Cohen's *d*, Pearson's *r*), indicating how they were calculated

Our web collection on statistics for biologists contains articles on many of the points above.

Software and code

Policy information about availability of computer code

Data collection	No software was used for data collection.
Data analysis	STAR was used to align reads to the reference genome (CRCh38), and used HTSeq-count to generate gene counts based on a Ensembl (v.86) reference file. An updated version of TagGD was used to demultiplex the spatial barcode. ST-pipeline was used to pre-process the spatial data, i.e. spot selection and image alignment .<br><br>Analysis of the spatial data, independent integration of single cell data was mainly performed using STUtility and features from Seurat. The single cell mapping was performed by using stereoscope. The R package shiny was used to create the interactive interface to explore the data. All code associated with the manuscript (i.e., used to generate the presented results) are available at the main github repository, either formatted as R Markdown files or CLI applications. The main repository also lists detailed information regarding what part of the analysis each file/application was designed for. Upon publication, a clone of the GitHub repository have will also be deposited to doi:10.5281/zenodo.3957257.<br><br>Software listing:<br><br>STAR: https://github.com/alexdobin/STAR<br>HTSeq-count: https://github.com/simon-anders/htseq<br>TAgGD: https://github.com/SpatialTranscriptomicsResearch/taggd<br>ST-pipeline: https://github.com/SpatialTranscriptomicsResearch/st_pipeline<br><br><br>STUtility : https://github.com/jbergenstrahle/STUtility

Seurat: https://github.com/satijalab/seurat
stereoscope: https://github.com/almaan/stereoscope
main repository: https://github.com/almaan/her2st/
shiny: https://shiny.rstudio.com/

For manuscripts utilizing custom algorithms or software that are central to the research but not yet described in published literature, software must be made available to editors and reviewers. We strongly encourage code deposition in a community repository (e.g. GitHub). See the Nature Research guidelines for submitting code & software for further information.

Data

Policy information about availability of data

All manuscripts must include a data availability statement. This statement should provide the following information, where applicable:

- Accession codes, unique identifiers, or web links for publicly available datasets
- A list of figures that have associated raw data
- A description of any restrictions on data availability

The raw sequencing files for the Spatial Transcriptomics (ST) data generated in this study are available with restricted access at the European Genome-Phenome Archive (EGA) under the identifier EGAD00001008031, access can be obtained by contacting Åke Borg (ake.borg@med.lu.se). The processed count matrices derived from the raw ST data and the associated brightfield images (HE-images) are available at doi: 10.5281/zenodo.4751624. The public data for the Visium breast cancer sample can be accessed at 10x Genomics's support website (https://support.10xgenomics.com/spatial-gene-expression/datasets, sample: Space Ranger 1.1.0, Human Breast Cancer (Block A Section 1)). The public datasets of developmental heart (time point : 6.5PCW, sample : 4), melanoma (sample id: ST_mel1_rep1), and rheumatoid arthritis (sample id: RA_B_3) can all be accessed via www.spatialresearch.org. The public spatial and single cell data of Human Squamous Cell Carcinoma (SCC) can be accessed at the Gene Expression Omnibus (GEO) under the accession code GSE144240. The public processed single cell data from Wu et al. can be accessed via the Broad Institute Single Cell portal at https://singlecell.broadinstitute.org/single_cell/study/SCP1039. Public raw sequencing data from the Wu et al. study is accessible at EGA under the identifier EGAS00001005173 and access can be obtained by contacting the Data Access Committee. SKCM data from the TCGA database is publicly available and can be accessed from the two R packages RTCGA.clinical and RTCGA.rnaseq (versions 1.60.0). The remaining data are available within the Article or Supplementary Information.

Field-specific reporting

Please select the one below that is the best fit for your research. If you are not sure, read the appropriate sections before making your selection.

- [x] Life sciences
- [ ] Behavioural & social sciences
- [ ] Ecological, evolutionary & environmental sciences

For a reference copy of the document with all sections, see nature.com/documents/nr-reporting-summary-flat.pdf

Life sciences study design

All studies must disclose on these points even when the disclosure is negative.

Sample size	The number of patients included in the study was determined by the number of individuals who approved to donating tissue, i.e., access to samples. When reporting "n-values" for our spatial data, this relates to the number of spots that the tissue section covered, which is solely determined by the shape and character of the tissue piece being investigated
Data exclusions	We did not exclude any data from our analysis
Replication	Replication was not applicable to this study as it is of an exploratory character, not testing any form of intervention, and the sample access was limited.
Randomization	Randomization was not applicable to this study, since we do not look at different groups of individuals subject to different treatment/ intervention regiment.
Blinding	Blinding was not applicable to this study since it is exploratory in character and have no elements that might be influenced by bias from the subject or observer.

Reporting for specific materials, systems and methods

We require information from authors about some types of materials, experimental systems and methods used in many studies. Here, indicate whether each material, system or method listed is relevant to your study. If you are not sure if a list item applies to your research, read the appropriate section before selecting a response.

Materials & experimental systems

n/a	Involved in the study
☐	☒ Antibodies
☒	☐ Eukaryotic cell lines
☒	☐ Palaeontology and archaeology
☒	☐ Animals and other organisms
☐	☒ Human research participants
☒	☐ Clinical data
☒	☐ Dual use research of concern

Methods

n/a	Involved in the study
☒	☐ ChIP-seq
☒	☐ Flow cytometry
☒	☐ MRI-based neuroimaging

Antibodies

Antibodies used

1. Polyclonal Rabbit anti-Human CD3 (Dako, A0452)
2. Anti-human CD20 antibody [L26] mouse monoclonal antibody (Abcam, ab9475)

For (1) the provider supply the following information regarding their validation strategy:
"[The reagent is] Optimized for immunohistochemistry (IHC) with validated protocols"

and

"In Western blotting, the antibody detects bands of the expected molecular weights for CD3 antigens (2).The antibody recognizes CD3e in both a T-cell line (Jurkat) and a natural killer cell line (NK11), but does not react with lysates prepared from several B-cell lines (Raji, Ramos and JY), a myeloid cell line (U937) or a colon carcinoma cell line (Colo-205) (9).In immunoprecipitation from Nonidet P40 lysates of surface-iodated T lymphoblasts, the antibody precipitates the gamma (26kDa), delta (21 kDa) and epsilon (19 kDa) chain of the CD3 molecule, similar to the precipitation pattern seen using the well-characterized monoclonal mouse anti-human CD3, clone UCHT1 (1).In ELISA, the antibody labels the CD3 peptide used as immunogen

For (2) the provider supply the following validation statement (w.r.t. IHC usage):

"We use a variety of methods, including staining multi-normal human tissue microarrays (TMAs), multi-tumor human TMAs, and rat or mouse TMAs during antibody development. These high-throughput arrays allow us to check many tissues at the same time, providing uniformly as all tissues are exposed to the exact same conditions. "

Validation

Both antibodies are commercially available and has been validated by the manufacturer for IHC on human tissue, as indicated on their respective websites. The antibodies were also validated on tonsil tissue before the actual experiment, see main text Methods.

Human research participants

Policy information about studies involving human research participants

Population characteristics

All samples were collected from female primary breast cancer patients in Lund (Sweden) by the Department of Oncology and Pathology at the Department of Clinical Sciences, which belongs to Skåne Oncology Clinic

Recruitment

All patiens with primary breast cancer that was planed to undergo primary surgery could be asked to be recruited. Written information was given by trained health professionals that not themselves were part of the study and all patients provided written informed consent to participate in the study.

Ethics oversight

Samples were collected in concordance with the Declaration of Helsinki and has been approved by the Regional Ethical Review Board of Lund (diary numbers 2009/658).

Note that full information on the approval of the study protocol must also be provided in the manuscript.
